# Data-Driven Prediction of Enantioselectivity for the Sharpless Asymmetric Dihydroxylation: Model Development and Experimental Validation

**DOI:** 10.1021/acscentsci.5c00900

**Published:** 2025-07-29

**Authors:** Blake E. Ocampo, Bilal Altundas, Matthew J. Bock, Sara Feiz, Scott E. Denmark

**Affiliations:** Roger Adams Laboratory, Department of Chemistry, 14589 University of Illinois, Urbana, Illinois 61801, United States

## Abstract

The Sharpless asymmetric dihydroxylation remains a key transformation in chemical synthesis, yet its success hides unexpected cases of lower selectivity. A chemoinformatic workflow was developed to allow data-driven analysis of the reaction. A database of 1007 reactions employing AD-mix α and β was curated from the literature, and an alignment-dependent, fragment-based featurization of alkenes was implemented for modeling. This platform converged on machine learning models capable of predicting the magnitude of enantioselectivity for multiple alkene classes, achieving *Q* ^2^_F3_ values ≥ 0.8, test *r* ^2^ values ≥ 0.7 and mean absolute errors (MAE) ≤ 0.3 kcal/mol. The features of alkenes contributing to model performance were assessed with SHapley Additive exPlanations (SHAP) analysis to gather insight into factors underlying predictions. Experimental validation demonstrated that the models could achieve meaningful predictions on out-of-sample alkenes.

## Graphical abstract

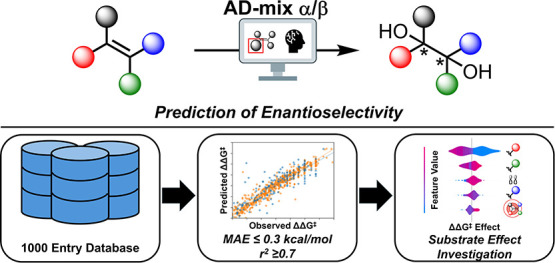

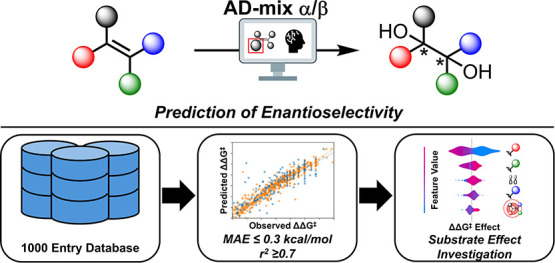

## Introduction

The Sharpless Asymmetric Dihydroxylation (SAD) remains a cornerstone of chemical synthesis in both academic and industrial settings, reliably forming enantio- and diastereomerically enriched vicinal diols from mono-, di-, and trisubstituted alkenes with high functional group tolerance.^1–3^ The proliferation of the SAD was enabled by the commercialization of the original mixture of reagents developed by Sharpless: AD-mix α and β. These mixtures use dimeric ligands derived from dihydroquinine (DHQ) or dihydroquinidine (DHQD).^4–6^

The transformation displays high generality for four alkene classes: monosubstituted, gem-disubstituted, trans-disubstituted, and trisubstituted alkenes, but the SAD continues to suffer from diminished enantioselectivity among some alkene classes (Figure 1A). Low selectivity is well-documented for cis-disubstituted, tetrasubstituted, and short-chain aliphatic monosubstituted alkenes.^1^ However, a number of examples of poor selectivity for other subclasses remain unaddressed, such as diaryl and short-chain aliphatic *gem*-disubstituted alkenes, trisubstituted alkenes containing similar 1,1-substitution, and alkenes in isochromane-derived compounds (Figure 1B).^7–15^

**Figure 1:**
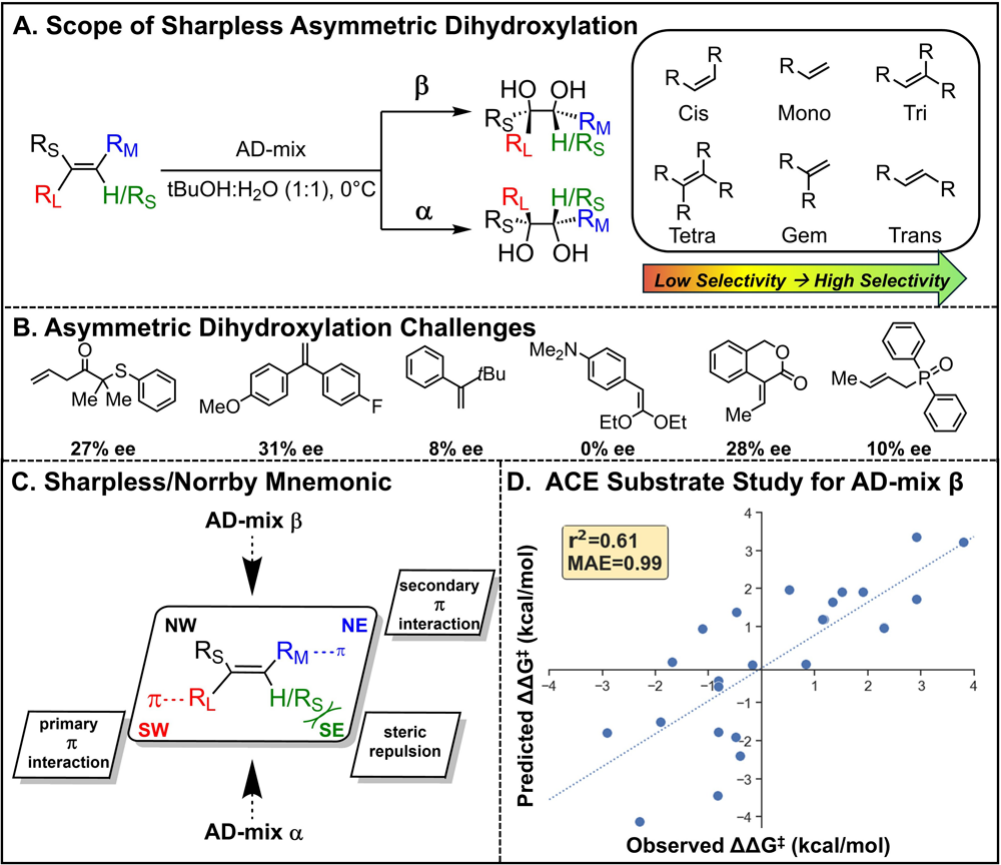
(**A**) Scope of the Sharpless Asymmetric Dihydroxylation. (**B**) Challenges of the Sharpless Asymmetric Dihydroxylation. (**C**) Illustration of Sharpless/Norrby mnemonic.^19,23^ (**D**) Example run of state-of-the-art ACE study predicting substrate selectivity with AD-mix β. Reproduced from ref [^24^]. Copyright © 2020 Springer Nature.

Regardless, designing solutions to problematic substrate classes is possible. Early modifications by Sharpless and Corey, provided selectivity improvements through empirical, hypothesis-driven design,^16–22^ but these designs are not necessarily generalizable. The Corey-Zhang ligand was designed for polyisopropenoid dihydroxylation, whereas the Sharpless ligand, DHQD-IND, could improve selectivity only to above 70% ee for a few cis-disubstituted alkenes.^21^ These solutions were driven by insights into how specific alkene classes interact with the catalyst. Creating a platform toward novel catalyst design could be enabled through a fundamental understanding of how different substrate features impact the selectivity of the SAD. However, the complexity of the mechanistic pathway and ambiguous trends in reactivity among substrates makes rationalizing the impact challenging.^19,21^

Sharpless and Norrby devised a quadrant model to predict the facial selectivity of dihydroxylation with the DHQ and DHQD ligands.^19,23^ The quadrant model rationalized the facial selectivity through π-interactions and steric repulsion of the substrate within the catalyst binding pocket (Figure 1C). This model serves as a reliable mnemonic device for facial selectivity predictions, but can still fail when misassignments of substituents in the mnemonic occur^25,26^ or when the mnemonic fails to capture necessary interactions (e.g., when polarizability is driving selectivity).^7,27^ Another method of rationalizing selectivity is only accessible with the application of hybrid quantum and molecular mechanics that can have lower accuracy.^23,28^

Moitessier and co-workers developed an elegant solution to this problem known as VIRTUAL CHEMIST, which enables the use of the ACE (Asymmetric Catalyst Evaluation) and Q2MM (quantum-guided molecular mechanics) methods, which have been successfully employed in a variety of transition metal-catalyzed, asymmetric transformations.^24,29,30^ However, predictions for the SAD were poor, converging on models with a mean unsigned error (MUE) of 0.96 ± 0.08 kcal/mol in reproducibility tests for substrate selectivity prediction. The main example reported in this work only achieves an *r* ^2^=0.61 with an MUE of 0.99 kcal/mol (Figure 1D). The ACE approach remains the best, general solution for predicting the selectivity of the SAD, but insufficient accuracy in complex cases precludes its use for evaluating broad selectivity trends in substrate performance.

Given the widespread application and data available for the SAD, there is a unique opportunity to apply data science and machine learning (ML) strategies to overcome the deficiencies in both quantum mechanical modeling and empirical solutions. This approach could enable higher predictive power in the magnitude of enantioselectivity for the SAD, while also providing insight into the nature of interactions between diverse substrates depending on the representations employed.

In recent years, chemoinformatics has developed into a burgeoning field, focusing on the application of data-driven methods for the investigation and resolution of chemical problems.^31^ This development has resulted in many successful examples in the application of quantitative structure-selectivity relationships (QSSR) in asymmetric catalysis, such as those by Sigman,^32^ Grzybowski,^33^ Reid,^34^ Sunoj,^35^ Corminboeuf,^36^ Newhouse,^37^ and Hong.^38^

In addition, our group has developed a computer-driven workflow for reaction optimization.^39^ This workflow operates in a “mechanism agnostic” basis to promote generality in application, and demonstrated success for novel catalyst prediction.^40^ However, because this approach focuses on the catalyst as the selectivity-determining component, problems arise when the substrate plays a large role in enantioinduction or if there is a lack of variance in initial training set hits. These failures can impede identification of a meaningful QSSR but have been addressed successfully using alternative algorithmic solutions in two case studies.^41,42^ The SAD instead enables a truly catalyst agnostic investigation into substrate effects on enantioinduction and can overcome the limitation of empirical, hypothesis-driven design.

The work presented herein describes the creation of a chemoinformatic workflow to enable data-driven analysis of the SAD, representing the first ML approach toward successful prediction of enantioselectivity for this reaction. This workflow employs literature data by curating a database of reactions using only AD-mix α and β. Despite the established limitations of literature data in both heterogeneity and positive skew,^43–45^ the SAD reaction poses a unique opportunity for multiple reasons: (1) conditions for optimal reactivity have been established since 1994, making many reports on the transformation very similar, (2) differing ratios of reagents and additives often impact only yield and conversion rather than enantioselectivity, and (3) the two ligands give similar magnitudes of enantioinduction allowing both to be leveraged.^1^ The premise of this work argues that accurate prediction of selectivity for the SAD reaction can be achieved successfully solely on the basis of substrate featurization. Featurization with physically relevant descriptors allows a critical analysis of how the alkene structure impacts enantioselectivity, and predictions from the resulting models are then validated experimentally on substrates not previously reported.

## Methods

### Literature Mining and Database Construction

The literature describing the SAD is immense and widely accessible through the chemical databases Scifinder and Reaxys. We identified serious problems traceable to errors in data transcription from original sources into the database (see Supporting Information). To allow for accurate curation, the decision was made to consult expert encyclopedias and reviews first and then transition into the remaining primary literature using Scifinder when those sources were exhausted.^1–3^ Data collection was enabled by a semiautomatic workflow to minimize errors and heterogeneity in reports scraped from the literature. This workflow involved the creation of ChemDraw files for all reactants and products, conversion to their canonicalized SMILES strings using RDKit,^46^ and subsequent standardization of entries to remove any redundant information.

The data collected for each reaction was reduced to basic factors associated with enantioselectivity, including temperature if reported, oxidant, additive, yield, and alkene class. The data was limited to achiral alkenes to isolate modeling to enantioselectivity measurements. The current database contains 1007 unique entries (Figure 2), comprising 987 diols and 784 alkenes, of which 203 were oxidized with *both* AD-mix α and β. The remaining 20 entries are cases of alkenes with different oxidants, solvents, and temperatures which were not used in modeling. These data illustrate a tendency toward high selectivity, with trans-disubstituted alkenes displaying the highest positive skew (95% ee median). This outcome may be consistent with positive publication bias often seen in chemical data derived from literature data sets.^44^ Nevertheless, the SAD reaction shows a reasonable spread of enantioselectivity within the literature data, especially for a transformation that is considered robust.

**Figure 2:**
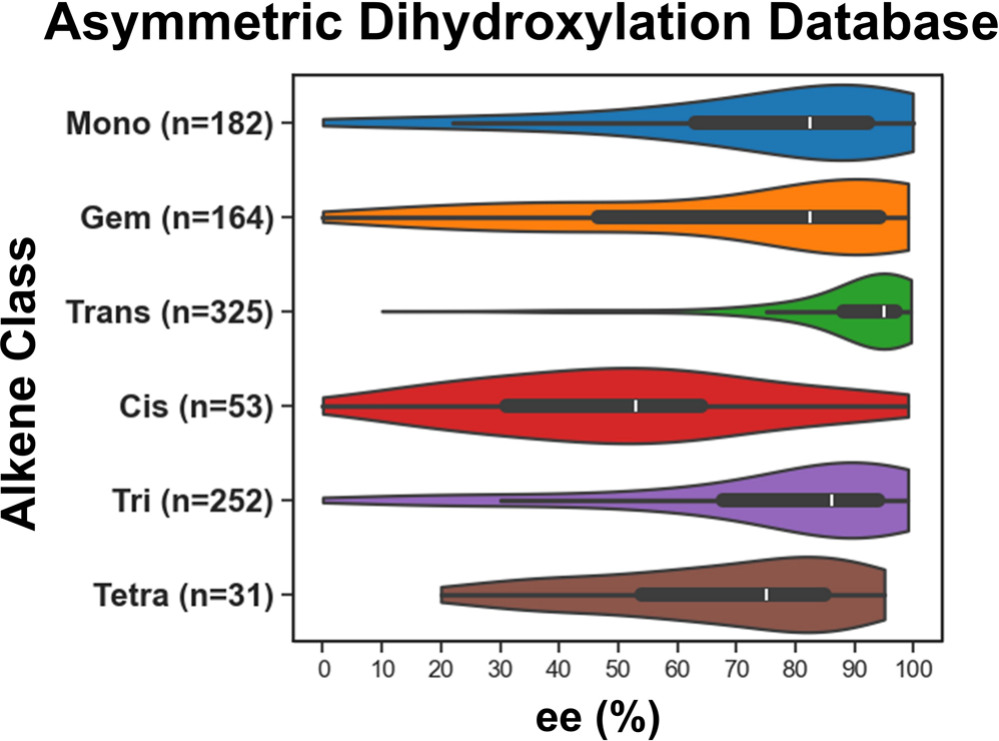
Violin plot of current database organized by % ee.

### Descriptor Implementation

Inspired by the success of the facial selectivity model pioneered by Sharpless and Norrby,^19,23^ a fragment-based descriptor design was implemented to capture effects of individual quadrants. A workflow for alkene substructure identification capable of differentiating highly diverse alkenes was created utilizing RDKit (see Supporting Information) and was subsequently extended to diol structures. Steric features of each quadrant were featurized with Sterimol parameters calculated by the Morfeus implementation of the first three atoms of the substituent,^47–49^ as well as two volume descriptors. One is derived from the full volume of the entire substituent referred to as “Max Volume,”^50^ and the other is derived from the volume as defined by the first three atoms of a breadth-first search (BFS) of the substituent referred to as “3-BFS Volume.” Electronic descriptors ESP_MIN_ and ESP_99_ were employed for each fragment to quantify electronic effects.^40^ These descriptors correlate well with existing Hammett parameters, suggesting they can successfully describe electron-donating and withdrawing properties of the fragments. Full alkene descriptors were also implemented, including the Radial Distribution Function (RDF),^51^ as well as descriptors derived from Natural Bond Orbital (NBO) analysis.^52^ RDF represents the average dispersion calculated at different spheres for various conformers around the alkene atoms. RDF also represents a description of average steric occupancy and conformational flexibility due to its conformer-dependence (see Supporting Information).

All workflows were created with the *molli* python package developed to enable parallelized calculations on libraries of compounds.^53^ (See Supporting Information regarding implementation).

## Results and Discussion

### Alignment Scheme and Descriptor Evaluation

Modeling commenced with substrates and associated enantioselectivity data curated from major review articles. Descriptors were calculated for all alkenes by attempting to automate the Sharpless/Norrby mnemonic using a fragment-based approach. Substituents were placed in the bottom left quadrant (**Q1**) based on priority rules. The rules in decreasing order of priority were:

1. **Q1** has the largest volume substituent
2. **Q1** has the smallest volume substituent cis (**Q2**)
3. **Q1** has the largest ESP_99_
4. **Q1** has the largest ESP_99_ trans (**Q3**)
5. **Q1** has an atom with the largest polarizability connected to the alkene
6. **Q1** has an atom with the largest polarizability trans (**Q3**)

Once **Q1** was decided, the remaining descriptors were concatenated in a counterclockwise orientation around the alkene to allow meaningful comparison of diverse alkenes (Figure 3A). Alkenes were categorized into eight categories on the basis of their substitution pattern: Mono, Gem, Cis, Trans, TriQ2, TriQ3, TriQ4, and Tetra. For trisubstituted alkenes, the Q notation indicates the location of the hydrogen atom (i.e., for TriQ3, the hydrogen is in **Q3**). The full alkene RDF/NBO descriptors were then concatenated, to create a chemical space of 57 features.

**Figure 3:**
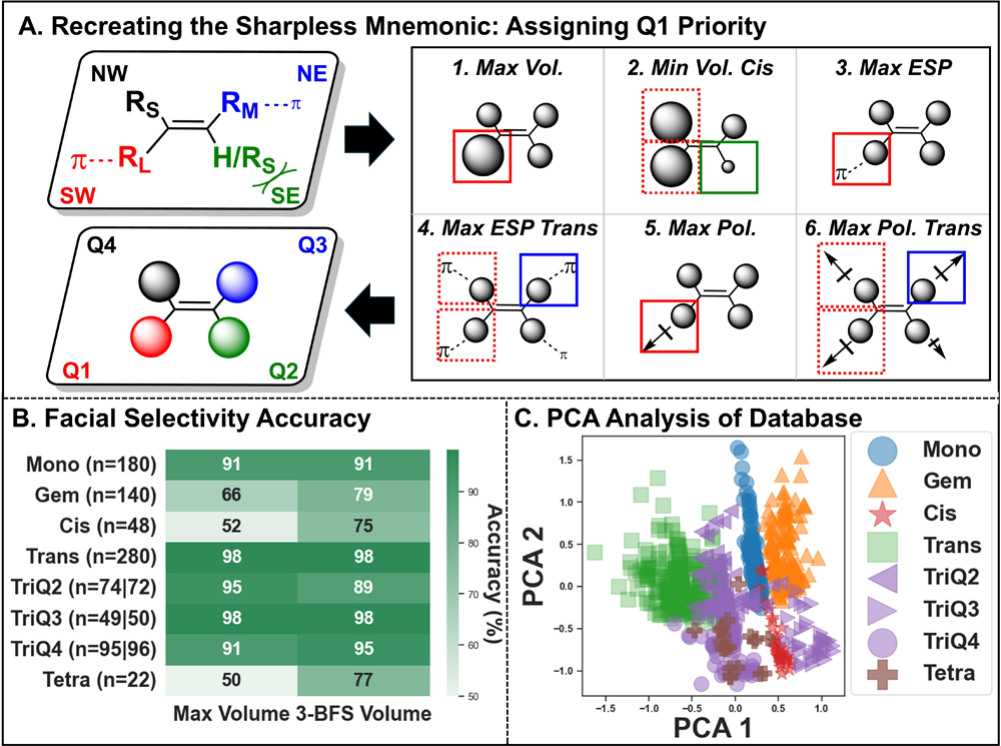
(**A**) Illustration of Sharpless mnemonic being translated to the Q1 priority alignment scheme for all alkene classes. (**B**) Facial selectivity accuracy with different alignment schemes. The vertical bar indicates some alkenes have been recategorized with a different alignment scheme. The number on the left indicates the total number of alkenes with Max Volume, while the number on the right indicates the total number of alkenes with 3-BFS Volume. (**C**) Visualization of the chemical space reduced to the first two dimensions of principal component analysis (PCA).

A system was designed to map the reactant descriptors to their respective diols to validate the mnemonic and alignment scheme. This scheme enabled assignment of top- or bottom-face dihydroxylation of the alkene independent of the Cahn-Ingold-Prelog system, which prevents problems with inconsistent prioritization that can occur in molecules with highly diverse substituents. Of the original 784 alkenes, 705 were mapped to their respective 888 diols and assigned top or bottom face addition according to the above alignment scheme. The entire database was not used owing to limitations in the substructure matching approach (see Supporting Information). Two systems were tested for alignment: (1) the volume being determined by the Max Volume of the substituent or (2) the volume being determined by the 3-BFS Volume. In this approach, accuracy refers to the alignment system giving the expected result of the mnemonic, where AD-mix α gives bottom-face dihydroxylation, and AD-mix β gives top-face dihydroxylation.

These tests showed that the Sharpless mnemonic was maintained to a high degree of accuracy with a Max Volume representation. In addition, the alignment scheme accuracy could be further increased for Cis, Gem, and Tetra alkenes using a 3-BFS Volume criterion (Figure 3B). Since the 3-BFS volume alignment system is more likely to contain multiple quadrants with similar volumes, other features of the alkenes can determine the **Q1** assignment. A higher accuracy should be expected when factors outside of the largest volume are driving the direction of selectivity, such as electronic interactions in the catalyst pocket that may occur in gem-disubstituted alkenes. Inaccuracies of assignment were also associated with known failures of the mnemonic.^27,54–57^ The alignment system functions as an independent, accurate classifier for maintaining the Sharpless mnemonic. The success of this system suggests that the computed descriptors enable a new, quantitative method for interpreting the mnemonic that is not reliant on the subjective assignment of priority.

An unsupervised learning approach was taken to qualitatively evaluate the chemical space in maintaining relevant relationships between alkenes. A score plot was created using the first two components in principal component analysis^58^ (PCA). The score plot illustrates strong segmentation of the alkenes into their individual classes (Figure 3C). The projection suggested that the unified descriptor space effectively captures the differences among the alkene types, which is fundamental with respect to the SAD. This feature space also suggested that relationships between certain alkene classes were maintained such as Cis and TriQ3 being closer in chemical space than Cis and TriQ2.

### Modeling Results

Features were scaled and highly correlated features were removed prior to regression analysis. Because most data points in the library used 0 °C or cited the original Sharpless conditions, temperature was not used as a feature and instead all values for enantioselectivity (% ee) were converted to ΔΔ*G* ^‡^ in kcal/mol at 0 °C. In addition, all ΔΔ*G* ^‡^ values for both AD-mix α and β were averaged and normalized to a positive value. The change to a normalized ΔΔ*G* ^‡^ value focuses the model outputs on the prediction of the magnitude of enantioselectivity rather than the enantiomer produced.

Modeling attempts included PLS Regression,^59^ Support Vector Regression (SVR),^60^ Ridge,^61^ Lasso,^62^ Random Forest (RF) Regression,^63^ Gradient Boosting Regression (GBR),^64^ XGBoost Regression,^65^ *k*-neighbors regression,^66^ and Gaussian process regression (GPR).^67^ Models were constructed with a train/test split of 80/20 with parameters optimized using a 5-fold cross-validation randomized search. These results were then extended using a 5-fold cross-validation Bayesian optimization of model hyperparameters.^68^ Final model architectures for individual alkene classes were then slightly modified on the basis of results from further screening.

Regression analysis was applied to separate substrate classes. Isolating regression relationships to individual substrate classes provides insight into features driving enantioselectivity for different alkene substitution patterns. Nonlinear modeling architectures invariably performed best for all alkene classes. GBR was found to be a top-performer for all alkene classes except Trans and Tetra, which moved forward with RF and GPR respectively. The main metrics used for model evaluation are MAE, *Q* ^2^_F3_, *R* ^2^, and *r* ^2^. Despite their often interchangeable use, *R* ^2^ is not to be confused with *r* ^2^, as these refer to the coefficient of determination and squared correlation coefficient, respectively.^69^ *Q* ^2^_F3_ is used as a key test performance metric, as it has been shown to provide a more robust assessment of quantitative performance (eq 1).^70,71^ PRESS refers to the predicted residual sum of squares, TSS refers to the total sum of squares, *n*EXT refers to the number of instances in the external set, and *n*TR refers to the number of instances in the training set. The *r* ^2^ value is also included to illustrate a more familiar metric associated with the line of best fit often used in QSAR and property modeling (see Supporting Information for further discussion of statistical metrics).

1 $$Q_{F3}^{2}=1-\frac{\mathrm{PRESS}/n_{\mathrm{EXT}}}{\mathrm{TSS}/n_{\mathrm{TR}}}$$

When examining the metrics of all the models together, the results show a train *R* ^2^ of 0.91, train MAE of 0.16 kcal/mol, test *r* ^2^ of 0.72, a *Q* ^2^_F3_ value of 0.93, and MAE of 0.30 kcal/mol. These models display the lowest prediction errors reported to date for broad regression analysis of the SAD.^24^ All individual models are shown in Figure 4. Separate visualization of the train and test is shown in the Supporting Information.

**Figure 4:**
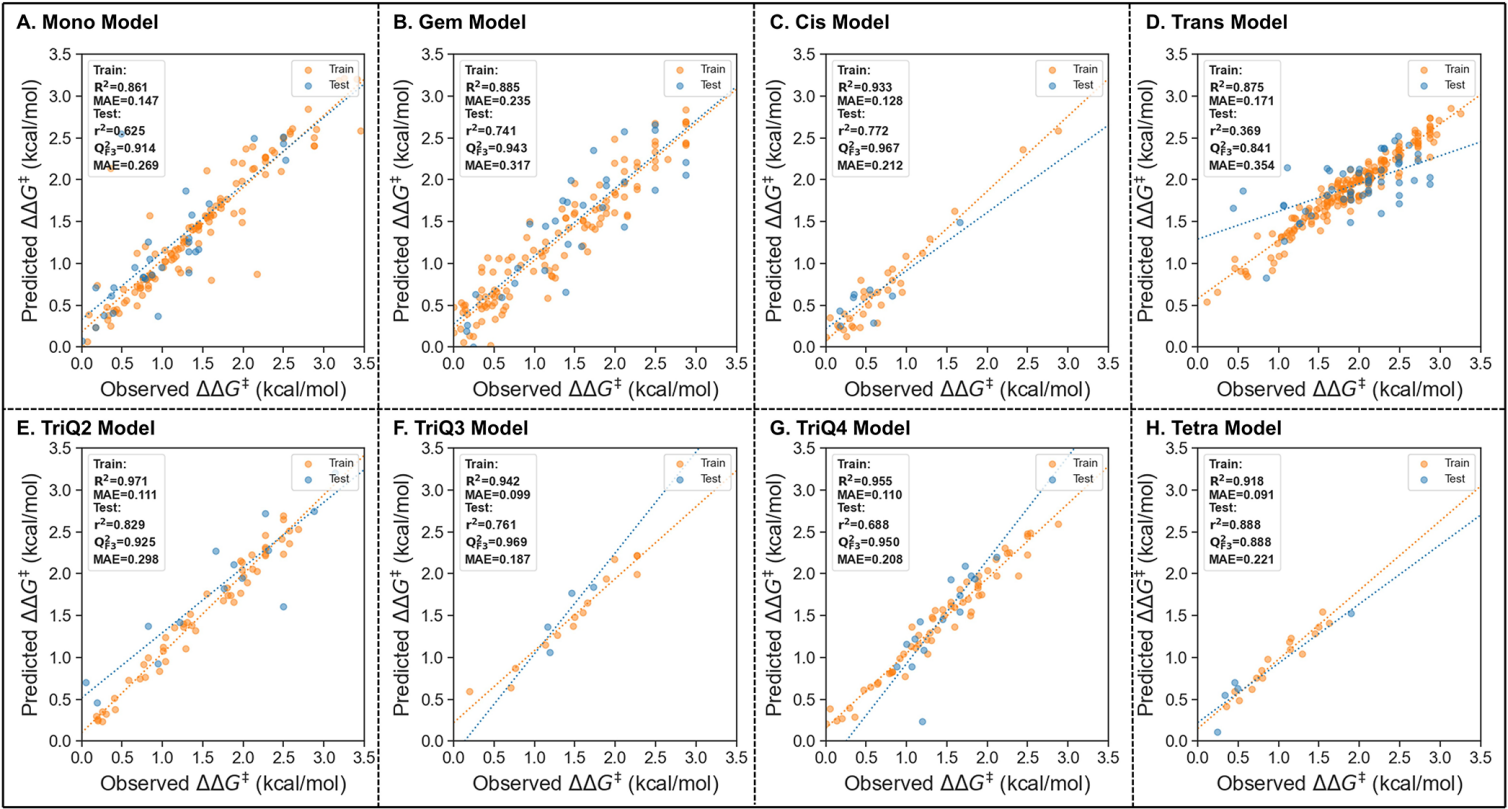
Full modeling results for each alkene class. (**A**) Mono, (**B**) Gem, (**C**) Cis, (**D**) Trans, (**E**) TriQ2, (**F**) TriQ3, (**G**) TriQ4, (**H**) Tetra.

The monosubstituted alkene model shows high efficacy achieving a *Q* ^2^_F3_ of 0.91, an excellent test MAE of 0.27 kcal/mol, and a moderate test *r* ^2^ of 0.63. The reduced *r* ^2^ performance can be attributed to react_242 in the train set (Chart 1), which fell outside of three standard deviations of the mean of the test set. The ΔΔ*G* ^‡^ of this alkene was predicted to be 2.54 kcal/mol but observed to only be 0.48 kcal/mol. The result is unexpected in the SAD, as alkenes containing styrene-like substructures are well-established to be high performing. Removing the outlier from the test set results in the test *r* ^2^ performance increasing to 0.85, and the test MAE decreasing to 0.21 kcal/mol. The monosubstituted alkene model is generally high performing but shows clear limitations on styrene-like compounds showing poor performance.

**Chart 1:**
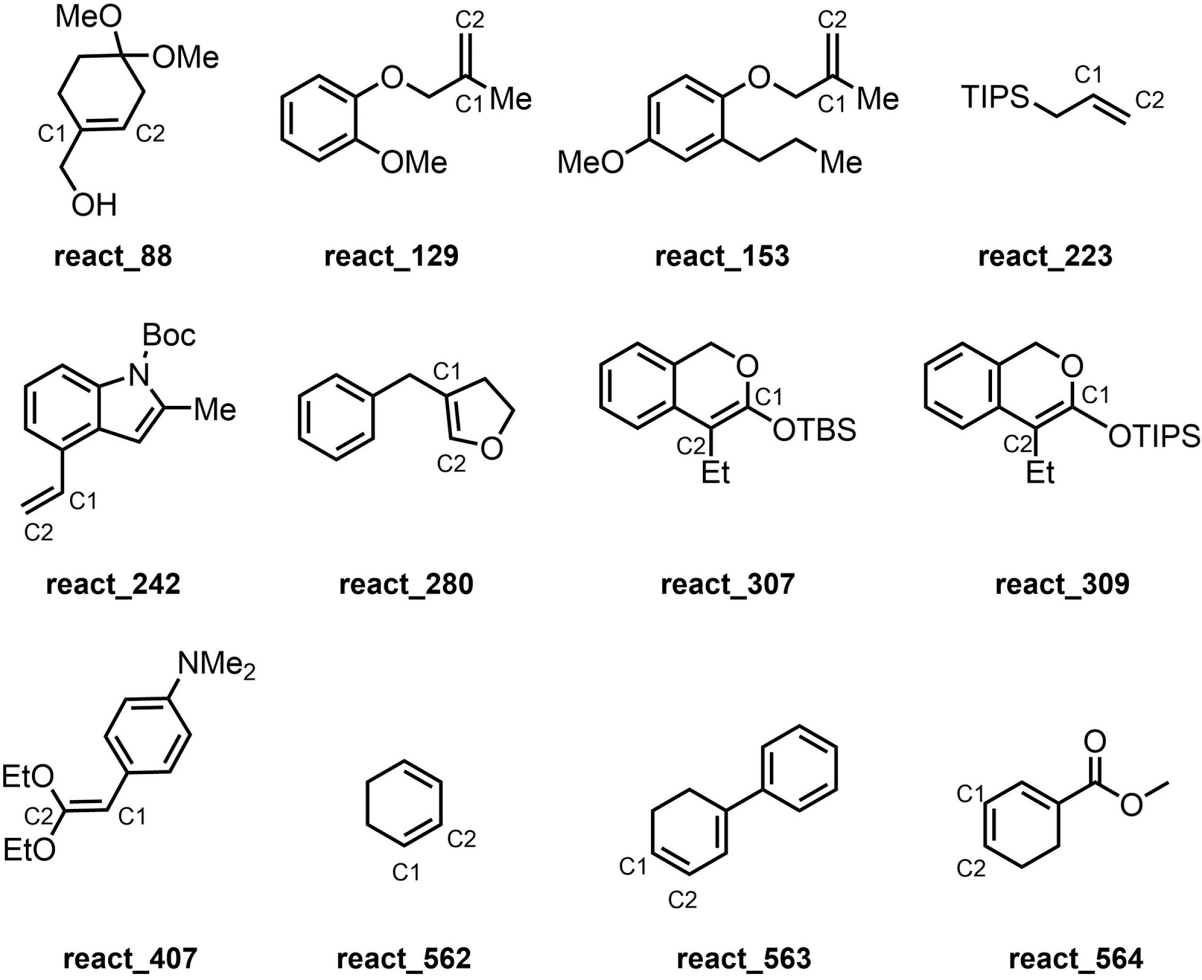
Compounds from the Database Referenced in the Results and Discussion

The Gem alkene model showed similar metrics to the Mono model, with slightly lower performance for regression, achieving a *Q* ^2^_F3_ of 0.94, a good test MAE of 0.32 kcal/mol, and a moderate test *r* ^2^ of 0.74. A higher spread is seen in the residuals, but the model still shows good performance in an out-of-sample test with a large range of enantioselectivity.

Successful prediction of an out-of-sample test was seen in both the cis-disubstituted and tetrasubstituted classes. The Cis alkene model reached a *Q* ^2^_F3_ of 0.97, test MAE of 0.22 kcal/mol, and test *r* ^2^ of 0.74, whereas the tetrasubstituted model reached a *Q* ^2^_F3_ of 0.89, test MAE of 0.22 kcal/mol, and a test *r* ^2^ of 0.89. Although these are excellent metrics, it is important to note that much less data is available for modeling, with only 41 cis-disubstituted and 21 tetrasubstituted alkenes. Both models show poor performance in cross-validation tests, with a *q* ^2^_5‑fold_ at 0.23 for Cis and −0.15 for Tetra, suggesting a lack of stability in the models and a high chance of overfitting. Regardless, performance in out-of-sample tests is still considered a key metric to assess the utility of models,^72^ and further experimental validation will provide a platform for understanding the limitations of these models (see Supporting Information).

All trisubstituted alkene models showed successful performance. TriQ2 achieved a *Q* ^2^_F3_ of 0.93, test MAE of 0.30 kcal/mol, and a test *r* ^2^ of 0.83, TriQ3 achieved a *Q* ^2^_F3_ of 0.97, test MAE of 0.19 kcal/mol, and a test *r* ^2^ of 0.76, and TriQ4 reached a *Q* ^2^_F3_ of 0.95, test MAE of 0.21 kcal/mol, and test *r* ^2^ of 0.69 kcal/mol. Despite reasonable performance with all three alkene subclasses, the split employed had a pronounced effect on the distribution of alkenes available. Instead of having 160 alkenes in the model, there were 67 alkenes in TriQ2, 18 in TriQ3, and 75 in TriQ4. Such a division is expected to reduce the ability to evaluate the TriQ3 model with this approach, as there are only four alkenes available for testing with a traditional 80/20 split. Cross-validation supported this assertion, with the best cross-validation metrics for leave-one-out (LOO) only reaching a *q* ^2^ of 0.29 for the TriQ3. A higher potential of overfitting with lower extrapolative power is expected.

The trans-disubstituted alkene class failed to achieve satisfactory regression. The best model reached a *Q* ^2^_F3_ of 0.84, test MAE of 0.35 kcal/mol, and test *r* ^2^ of 0.37. This class was expected to perform poorly in regression given its high positive skew with a median of ΔΔ*G* ^‡^ = 1.99 kcal/mol (95% ee). It is important to note that this model still performed better than simply predicting the average, which would afford an MAE of 0.47 kcal/mol. A reasonable performance in *Q* ^2^_F3_ further supports that this model can recognize some underperforming alkenes in the test set.

### Feature Importance Analysis

Having identified good regression models with physically meaningful descriptors, we next investigated how individual features impact the predicted enantioselectivity. This approach was taken to provide insight into the molecular interactions pertinent to enantioinduction for the SAD. Feature importance was conducted using SHapely Additive exPlanations (SHAP) analysis.^73^ SHAP is a model-agnostic approach to feature analysis driven by game theory for the evaluation of how features and their associated instances impact the prediction of the dependent variable, which in this case is the predicted ΔΔ*G* ^‡^.^74^ Each feature of an alkene is assigned a SHAP value in kcal/mol, and the sum of SHAP values will represent the predicted ΔΔ*G* ^‡^ for an alkene. A common way of presenting the SHAP analysis of an entire data set is a beeswarm plot, which is shown in Figure 5 for all alkene models.

**Figure 5:**
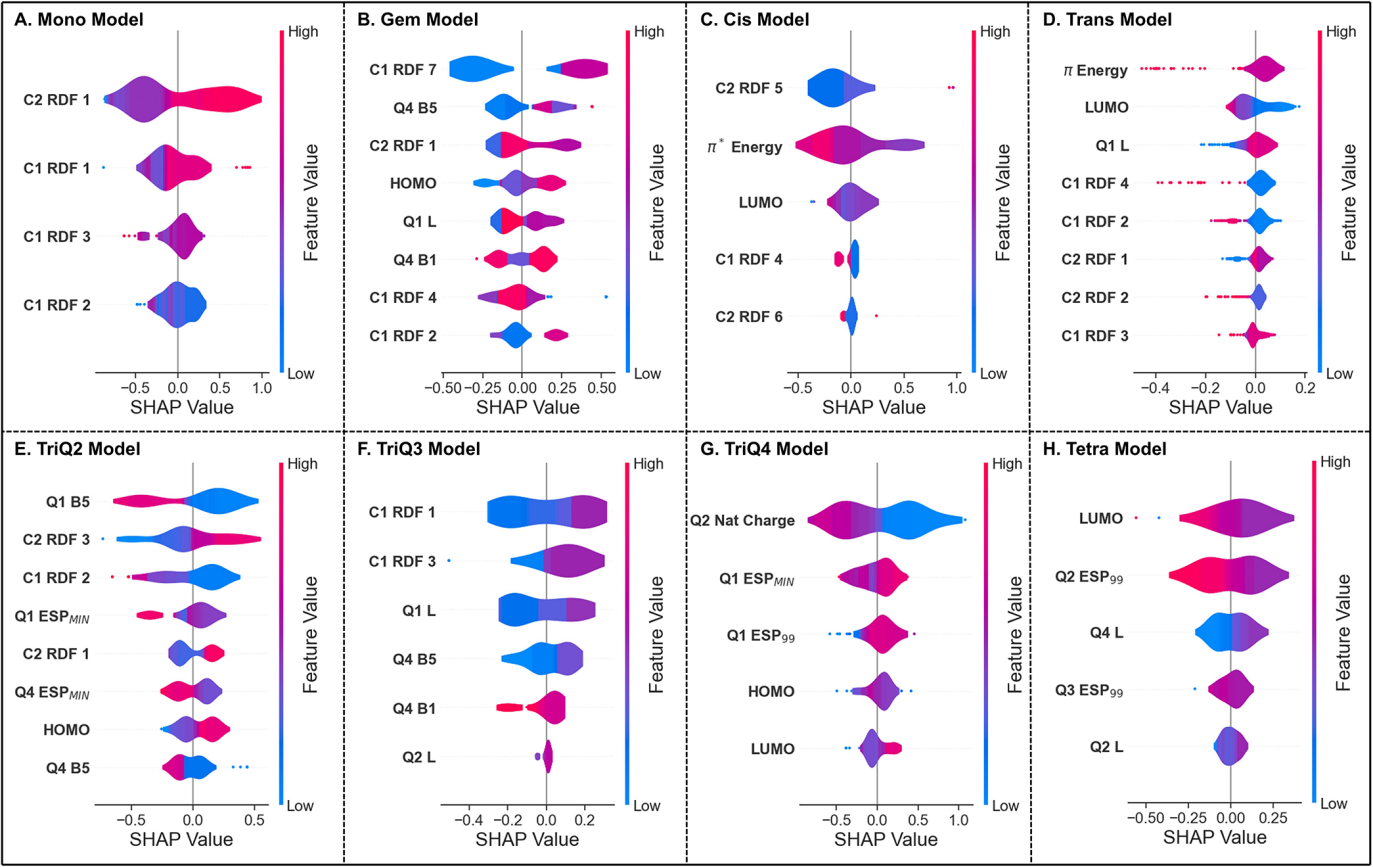
SHAP analysis results for each alkene class as beeswarm plots. Top features for each model are shown with the distribution of SHAP values shown with a violin plot kernel. (**A**) Mono, (**B**) Gem, (**C**) Cis, (**D**) Trans, (**E**) TriQ2, (**F**) TriQ3, (**G**) TriQ4, (**H**) Tetra.

On the *y* axis, the top features for the models are shown, while the calculated SHAP values in kcal/mol are shown on the *x* axis. Negative SHAP values indicate a net decrease in value of the predicted ΔΔ*G* ^‡^, while positive SHAP values indicate an increase. For each feature, a violin plot is constructed to show the distributions of SHAP values for all alkenes with respect to a specific feature. The color of the violin plot indicates the magnitude of the feature itself, with red indicating a high magnitude and blue indicating a low magnitude. A beeswarm plot shows if the feature behaves consistently across different alkenes, such as a high magnitude of a feature being consistently correlated with a higher prediction. SHAP analysis also shows outliers, which suggests that the model is relying heavily on specific alkenes for predictions.

SHAP analysis is inherently correlational and does not imply causation. The work presented herein used SHAP as a quantitative tool to help identify alkenes and features that are important to the model. These cases are then examined to determine whether certain features or alkene substructures display consistent behavior with respect to enantioselectivity. SHAP serves as a method to help generate hypotheses about enantioselectivity in the SAD, which guided further analysis of the existing database and motivated experimental validation.

The monosubstituted alkene model used only four features, all of them being related to the conformer-dependent RDF dispersion descriptor. The most important features with consistent behavior were those centered around the alkene carbon (**C1**) connected to the **Q1** substituent, and the terminal alkene carbon (**C2**) (see Supporting Information Figure S6). Both descriptors showed that larger feature magnitudes are generally correlated to higher predictions of enantioselectivity, and lower magnitudes are correlated to lower predictions. To have high feature values of C2 RDF 1, there must be consistent conformational occupation of the first sphere around the **C2** carbon by multiple atoms. The higher feature values of C2 RDF 1 suggest that the **Q1** substituent is flexible enough to reach the first sphere around the **C2** carbon, whereas the high C1 RDF 1 values suggest there are consistently more atoms in the closest sphere around **C1**. A key example that supports this hypothesis, is react_223 (Chart 1). In this alkene, the silyl substituent has the flexibility to reach C2 RDF 1 which increases the prediction by 0.64 kcal/mol. However, the silyl moiety barely occupies the C1 RDF 1 sphere which decreases the prediction by 0.88 kcal/mol. Thus, the RDF descriptors can differentiate styrene-like from more conformationally flexible structures.

The Gem alkene SHAP analysis revealed an unexpected bimodal distribution for C1 RDF 7 with only 2 key SHAP values being assigned. The model correlates the length required to reach the seventh sphere with higher selectivity. This result could be rationalized as the alkene having a higher likelihood of differentiating between the **Q1** and **Q4** substituents. Additionally, the behavior of Q4 B5 did not offer more than a simplistic differentiation of the width of substituents, with values of phenyl and *tert*-butyl being of similar magnitude. These combined correlations show that the Gem model does not rely on structural differentiation close to **C1**, but rather differences over the full alkene.

In the Cis alkene SHAP analysis, the most notable features are the C2 RDF 5 and the π* energy. For C2 RDF 5, the outlier SHAP values on the high end share a phenyl ring within three atoms of **C1**, such as react_563 (Chart 1), or alkenes containing a halogen or heteroatoms around this sphere. This result suggests that the alkene may bind in the catalyst pocket with the correct conditions met on **Q1** to enable higher enantioselectivity

The Trans alkene SHAP analysis has a distinct dependence on π energy, LUMO, Q1 L, as well as several of the closest spheres of RDF on both **C1** and **C2**. The model puts a higher priority on the lower predicted alkenes as they deviate significantly from the expected values. Interestingly, high π energy outliers included macrocycles and allylsilanes, which would be expected given the distortion possible from strain in macrocycles and impact a silicon moiety would have on the system. Most SHAP outliers have overlap with other features, and consequently, if there are no striking differences in π energy, C1 RDF 4, and LUMO, the models tend to default to a prediction of the expected value or average in the data set.

SHAP analyses for TriQ2, TriQ3, and TriQ4 models all show different behaviors on the chosen features and on general correlations. TriQ2 shows consistent behavior with high values of Q1 B5 leading to lower predictions. This descriptor appears to differentiate methylene groups from aryl substructures by a rotation out of plane that leads to a higher value of Q1 B5, such as the methylene group in react_280 (Chart 1). Although this aspect is a correlative association, it has strong implications in how the subunit generally performs and where higher enantioinduction is expected. The similar magnitude of feature importances prevented further rationalization of the impact of electrostatic features on model prediction.

In the TriQ3 alkene SHAP analysis, the most important features were C1 RDF 1, C1 RDF 3, Q1 L, and Q4 B5 consistently showing that higher magnitudes lead to higher predictions. Styrene substructures are generally recognized with C1 RDF 1, like the Mono alkene model. The outliers of C1 RDF 3 share a similar cyclohexene-derived structure and low reported enantioselectivity that are essential to the existing model, such as react_88 (Chart 1). The reliance of the model on a few key points when there are so few available supports the original hypothesis that this model is not expected to generalize well outside the initial training data. The TriQ4 alkene model is noticeably more dependent on electronic features, with the top eight features all being electrostatic descriptors. The **Q2** natural charge has the highest average SHAP value, nearly twice the closest other descriptor. Many substituents with low Q2 natural charge values were methyl groups which correlated with higher predictions. Cyclic groups with higher **Q2** values consistently led to lower predictions. Larger SHAP values are associated with electron-rich subunits in **Q2** leading to a lower predicted selectivity, such as for react_407 (Chart 1). This result shows that steric effects contribute significantly to model predictions, but electronic effects can override the steric component.

The features in the tetrasubstituted alkene model included LUMO, Q2 ESP_99_, and Q3 ESP_99_. The LUMO generally shows that high values correspond with lower predictions. High values of LUMO are often electron-rich alkenes with aliphatic and π-donating groups. Q2 ESP_99_ plays a major role, but this is inconsistent with the mnemonic, suggesting spurious correlation with respect to this descriptor. However, Q3 ESP_99_ is still an important feature, with larger values being correlated with higher predictions. Thus, secondary π-interactions may be important for higher selectivity in tetrasubstituted alkenes.

The Sharpless/Norrby mnemonic has previously been used solely to understand the facial selectivity preference of the SAD, however this approach affords additional granularity in how those quadrants can drive changes in selectivity. In addition, the correlation analysis suggests that electronic features may be essential to understanding the selectivity of the SAD.

### Experimental Validation Design and Predictions

An experimental campaign was executed to validate the results of modeling. A separate external data set of alkenes was scraped from the literature employing any osmium-catalyzed dihydroxylations of alkenes, regardless of selectivity or ligands, with Scifinder utilizing OpenEye for parsing.^75^ This approach was taken to avoid problems with low yielding transformations and maximize the substrate scope covered. A set of 2,524 unique alkenes was obtained, which also included some alkenes previously collected in the SAD database. These alkenes were then featurized in the same fashion as the database. Fifteen alkenes were chosen for experimental validation through a combination of unsupervised analysis with *k*-means clustering, emphasis to pressure test models, and ease of synthetic accessibility (see Supporting Information). The alkenes chosen for experimental validation have unreported enantioselectivity in the SAD reaction. The selection is not intended to be comprehensive of the entire catalog of osmium-catalyzed dihydroxylations, rather it is meant to provide insight into the performance of the models.

The results of the experimental validation are shown in Figure 6. The model for monosubstituted alkenes showed excellent performance across all three alkenes, predicting within ±7% ee. The high accuracy in prediction with alkene **2** was a notable result, as this aligns with the SHAP analysis for distinguishing a styrene from an allylbenzene. The gem-disubstituted alkene class showed less consistent performance. The model predicted the lower performance of alkene **4** and accurately predicted **5**, however, the models missed other trends in enantioselectivity such as the poor selectivity with alkene **6** and the higher selectivity seen with **7**. The failure to recognize the detrimental impact of the ortho-substituent in **6** and the underprediction of **7** can be rationalized by the SHAP analysis, in which the model fails to recognize differences closer to the alkene. Several compounds with a matching 2-methylallyloxybenzene substructure, including react_129 and react_153 (Chart 1), showed a uniquely low performance of 14% and 24% ee when analyzing the test set. These are likely the main contributors to the low performance in the prediction of **7**, illustrating some sensitivity to the nearest training examples. There may be a limit in the existing model architecture in differentiating phenyl groups from their naphthyl counterparts. Additional literature data may also cause the model to lose accuracy, depending on the bias introduced.

**Figure 6:**
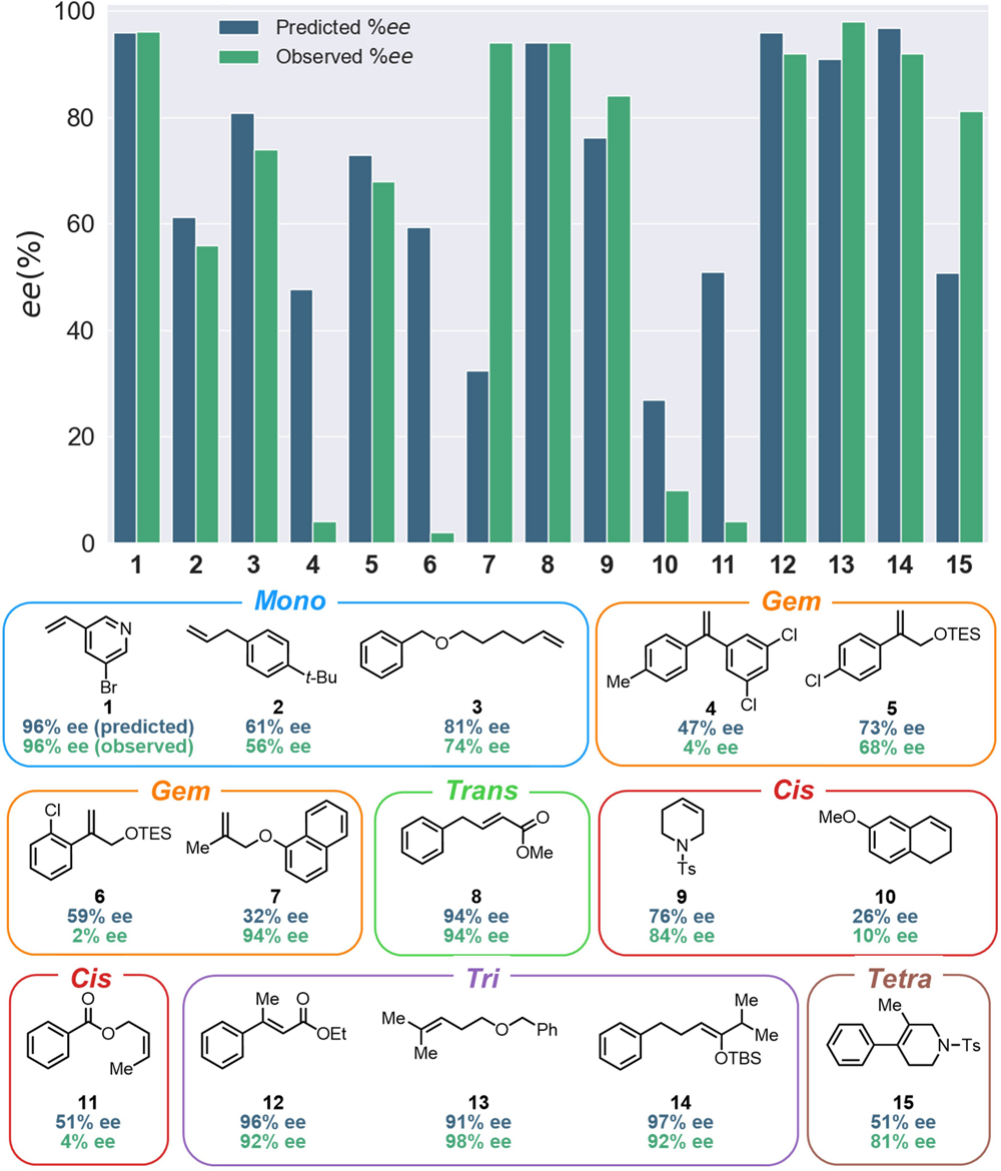
Experimental validation results for all alkene models with predicted shown on top (dark blue) and observed shown below (green).

The model for the trans-disubstituted alkene class showed high accuracy for its prediction despite its low performance on regression. The model tends to predict the expected value of enantioinduction, wherein the trans-disubstituted alkene class is consistently successful in the SAD. The model for the cis-disubstituted alkenes showed high predictive accuracy for substrates **9** and **10**, but not for **11**. Alkene **9** was chosen to test the hypothesis from the SHAP analysis that the alkene could bind within the catalyst structure to engender higher enantioselectivity. The model predicted **9** to be 76% ee, and the experimental result was 84% ee. There was no reason to expect this to perform much higher given that similar compounds in the database were cyclohexadiene analogues of react_562 and react_564 (Chart 1), with reported 37% and 31% ee. Despite potential problems of data limitation, the model still shows value because it is not just predicting the average of the class. Although alkene **11** is predicted to be somewhat less selective, it still shows high inaccuracy, illustrating hard limits on the extrapolative power of this model.

All model predictions for trisubstituted alkenes give experimentally relevant predictions, achieving at most ±7% ee error among all tests. This result supported the expectation that further subcategorization of the trisubstituted classes based on hydrogen location can lead to reasonable modeling. The categorization of alkene **12** was TriQ2, **13** was TriQ4, and **14** could be TriQ2 if aligned by 3-BFS Volume or TriQ3 if aligned by Max Volume. Given the lack of data available for TriQ3, it would be expected that the TriQ2 model would have a more robust prediction less prone to guessing. The TriQ2 model, shown in Figure 6, predicts a 97% ee, however, the TriQ3 model still shows a relatively accurate prediction of 87% ee. Thus, the TriQ3 model can still have reasonable predictive power on additional experimental validation. However, the model most likely will still have limited extrapolative power. The validation of tetrasubstituted alkene **15** gave an unexpected experimental result of 81% ee, whereas the model predicted a 51% ee. Although this is an experimentally relevant prediction, it is still underpredicted by 30% ee. This class also illustrated a similar sensitivity to nearest training examples like the Gem model with alkene **7**. When examining the components of the test set, although there are no alkenes with that same substructure, there are examples with similar connectivity in cyclohexene-derived compound react_307 and react_309 with 40% and 30% ee, respectively (Chart 1).

## Conclusions

In summary, a curated database of 1007 unique SAD reactions was compiled to enable data-driven development of a high-accuracy virtual prediction platform. Feature analysis of each model through SHAP showed the impact of alkene structures on enantioselectivities. Experimental validation was conducted on 15 alkenes previously unreported under SAD conditions to pressure test models and their extrapolative prediction ability. The models showed strong performance on many alkenes, predicting enantioselectivities within ±16% ee for 10 of the alkenes, while also illustrating some direct limitations in differentiating certain alkene substructures. These models predicted unexpected increases in enantioselectivity for cis-disubstituted and tetrasubstituted alkenes, despite poor performance seen in cross-validation.

The work described herein represents a foundational step toward higher accuracy prediction of the SAD, while also retaining a physically relevant representation to enable data-driven analysis of the impact of various features on enantioselectivity. The interactions of various alkene classes with the existing catalyst scaffold can be analyzed and interpreted through the lens of the Sharpless mnemonic originally devised only to rationalize facial selectivity. The consistent success of the alignment scheme with 3-BFS volume suggests the mnemonic could be translated from a qualitative to quantitative analysis for further refinement.

We envision that the alkene descriptor method developed in this work could be extended to additional alkene classes and that this database provides a new platform for researchers to conduct substrate descriptor investigations and development. The database and all specialized descriptor workflow calculations can be found at https://github.com/SEDenmarkLab/SAD. A simplified workflow measuring the similarity with any input alkene to the current database has been made available for researchers to allow quick evaluation as well. The success of this work also overturns a common assumption that literature data is too noisy or too positively biased to achieve meaningful internal and external performance of models. Ultimately, the selectivity principles learned herein could be leveraged to guide efforts toward novel catalyst design.

## Supplementary Material



## Data Availability

Source code for the project, database, and models for the workflows discussed in the manuscript can be downloaded from https://github.com/SEDenmarkLab/SAD.

## Notes

Synthetic protocols and analytical data of alkenes and diols, as well details on the computational methods (PDF)
