# Supplementary material for: Data-Driven Prediction of Enantioselectivity for the Sharpless Asymmetric Dihydroxylation: Model Development and Experimental Validation
Source: ACS Cent Sci. 2025 Jul 29;11(9):1640–50. doi: 10.1021/acscentsci.5c00900 (PMC12464766; doi:10.1021/acscentsci.5c00900)
Supplement: Supplementary file 1 [file oc5c00900_si_001.pdf]

Data-Driven Prediction of Enantioselectivity for the Sharpless Asymmetric Dihydroxylation: Model Development and Experimental Validation

Blake E. Ocampo, Bilal Altundas, Matthew J. Bock, Sara Feiz, and Scott E. Denmark*

Roger Adams Laboratory, University of Illinois, 600 S. Mathews Ave., Urbana, Illinois 61801, USA

SUPPORTING INFORMATION

TABLE OF CONTENTS **PAGE**

General Experimental: **S4**
Alkenes Chosen for Experimental Validations **S7**
Literature Preparation of Known Alkenes: **S8**
Preparation of Novel Alkenes: **S8**
Preparation of 3-Bromo-5-vinylpyridine (1): S8
Preparation of 1,3-Dichloro-5-(1-(4-tolyl)vinyl)benzene (4): S10
Preparation of ((2-(4-Chlorophenyl)allyl)oxy)triethylsilane (5): S12
Preparation of ((2-(2-Chlorophenyl)allyl)oxy)triethylsilane (6): S14
Preparation of (*Z*)-But-2-en-1-yl 3,5-bis(trifluoromethyl)benzoate (11): S16
Preparation of (*Z*)-*tert*-Butyldimethyl((2-methyl-6-phenylhex-3-en-3-yl)oxy)silane (14): S18
Preparation of 4-(4-Chlorophenyl)-5-methyl-1-tosyl-1,2,3,6-tetrahydropyridine (15): S21
Synthesis of Racemic Standards – Racemic Dihydroxylations **S24**
General Procedure for Racemic Dihydroxylations S24
Preparation of *rac*-1-(5-Bromopyridin-3-yl)ethane-1,2-diol (16): S25
Preparation of *rac*-3-(4-(*tert*-Butyl)phenyl)propane-1,2-diol (17): S26
Preparation of *rac*-6-(Benzyloxy)hexane-1,2-diol (18): S27
Preparation of *rac*-1-(3,5-Dichlorophenyl)-1-(4-tolyl)ethane-1,2-diol (19): S28
Preparation of *rac*-2-(4-Chlorophenyl)-3-((triethylsilyl)oxy)propane-1,2-diol (20): S30
Preparation of *rac*-2-(2-Chlorophenyl)-3-((triethylsilyl)oxy)propane-1,2-diol (21): S31
Preparation of *rac*-3-((7-Methoxynaphthalen-1-yl)oxy)-2-methylpropane-1,2-diol (22): S32
Preparation of *rac*-Methyl 2,3-Dihydroxy-4-phenylbutanoate (23): S34
Preparation of *rac*-1-Tosylpiperidine-3,4-diol (24): S35

Preparation of *rac*-7-Methoxy-1,2,3,4-tetrahydronaphthalene-1,2-diol (25): S36
Preparation of *rac*-2,3-Dihydroxybutyl 3,5-bis(trifluoromethyl)benzoate (26): S37
Preparation of *rac*-Ethyl 2,3-Dihydroxy-3-phenylbutanoate (27): S38
Preparation of *rac*-5-(Benzyloxy)-2-methylpentane-2,3-diol (28): S40
Preparation of *rac*-4-Hydroxy-2-methyl-6-phenylhexan-3-one (29): S41
Preparation of *rac*-4-(4-Chlorophenyl)-3-methyl-1-tosylpiperidine-3,4-diol (30): S42
Sharpless Asymmetric Dihydroxylations (SAD) S44
General Procedure for SAD: S44
Preparation of (*R*)-1-(5-Bromopyridin-3-yl)ethane-1,2-diol (16’): S45
Preparation of (*S*)-3-(4-(*tert*-Butyl)phenyl)propane-1,2-diol (17’): S46
Preparation of (*R*)-6-(Benzyloxy)hexane-1,2-diol (18’): S47
Preparation of (*R*)-1-(3,5-Dichlorophenyl)-1-(p-tolyl)ethane-1,2-diol (19’): S48
Preparation of (*S*)-2-(4-Chlorophenyl)-3-((triethylsilyl)oxy)propane-1,2-diol (20’): S49
Preparation of (*S*)-2-(2-Chlorophenyl)-3-((triethylsilyl)oxy)propane-1,2-diol (21’): S50
Preparation of (*S*)-2-Methyl-3-(naphthalen-1-yloxy)propane-1,2-diol (22’): S51
Preparation of methyl (2*S*,3*R*)-2,3-Dihydroxy-4-phenylbutanoate (23’): S52
Preparation of (3*S*,4*R*)-1-Tosylpiperidine-3,4-diol (24’): S53
Preparation of (1*R*,2*S*)-7-Methoxy-1,2,3,4-tetrahydronaphthalene-1,2-diol (25’): S54
Preparation of (2*R*,3*S*)-2,3-Dihydroxybutyl 3,5-bis(trifluoromethyl)benzoate (26’): S55
Preparation of Ethyl (2*S*,3*R*)-2,3-Dihydroxy-3-phenylbutanoate (27’): S56
Preparation of (*R*)-5-(Benzyloxy)-2-methylpentane-2,3-diol (28’): S57
Preparation of (S)-4-Hydroxy-2-methyl-6-phenylhexan-3-one (29’): S58
Preparation of (3*R*,4*R*)-4-(4-Chlorophenyl)-3-methyl-1-tosylpiperidine-3,4-diol (30’): S59
Computational Methods S60
Semi-Automatic Workflow for Database Construction S60
Alkene Descriptor Workflow S62
Step 1: 2D Alkene Identification S62
Step 2-4: 3D Structure Creation, Canonicalization, and Alkene Identification S63
Step 5: Alkene Fragmentation and Sterimol/Volume Calculation S64
Step 6: ESP, NBO, and RDF Descriptor Calculation S66
Diol Alignment for Facial Selectivity Assignment S70
Steps 1-4: 2D Diol Identification, 3D Structure Creation, and 3D Diol Identification S70
Steps 5-6: Mapping Alkene Descriptors to Diols and Facial Selectivity Assignment S72

Experimental Validation Selection S83
Model Parameters S87
Statistical Analysis S90
Compiled Metrics *S90*
Model Validation Tests *S91*
References S96
^{1}H, ^{13}C NMR Data and HPLC Traces S103

General Experimental:

General Procedures: All reactions were performed in oven- (110 °C) and/or flame-dried glassware under an atmosphere of dry nitrogen, unless otherwise indicated. Room temperature (rt) was approximately 20 °C, unless otherwise stated. "Brine" refers to a saturated solution of sodium chloride in H_2O. General procedures for racemic and asymmetric dihydroxylations are outlined in the relevant sections.

NMR Spectroscopy: ^{1}H, ^{13}C, and 19F NMR spectra for characterization and quantitative NMR were recorded on a Bruker 600 MHz (600 MHz, ^{1}H; 151 MHz, ^{13}C; 565 MHz, 19 F) or 500 MHz spectrometer (500 MHz, ^{1}H; 126 MHz, ^{13}C) at 21 °C unless otherwise noted. Dimethyl sulfone (Sigma Aldrich, 99.51%) was used the internal standard for quantitative NMR experiments. ^{1}H and ^{13}C NMR spectra are referenced to residual chloroform (δ = 7.26 ppm, ^{1}H; 77.16 ppm, ^{13}C). Chemical shifts are reported in parts per million. NMR values are reported as follows: chemical shift, multiplicity (s = singlet, br s = broad singlet, d = doublet, dd = doublet of doublets, ddd = doublet of doublet of doublets, dt = doublet of triplets, t = triplet, q = quartet, pent = pentet, hept = heptet, m = multiplet), coupling constant (Hz), integration, and signal assignment. Assignments were obtained by reference to APT, COSY, HSQC, and HMBC correlations.

Infrared Spectroscopy: Infrared spectra (IR) were recorded neat on a PerkinElmer FT-IR system and peaks were reported in cm^{-1} with indicated relative intensities: s (strong, 0-33% T); m (medium, 34-66% T); w (weak, 67-100% T).

Mass Spectrometry: Mass spectrometry (MS) was performed by the University of Illinois Mass Spectrometry Laboratory. Electron Impact (EI) spectra were performed on a VG 70-VSE (A) spectrometer (extended geometry) or a Waters GCT Premier (oa-TOF). Electrospray Ionization (ESI) and Atmospheric Solids Analysis Probe (ASAP) spectra were performed on a Waters Synapt G2-Si spectrometer (Q-TOF). Data are reported in the form of m/z (intensity relative to the base peak = 100).

Elemental Analysis: Elemental analysis was performed by the University of Illinois Microanalysis Laboratory. Reported data is the average of at least 2 runs.

Melting Points: Melting points (m.p.) were determined on a MPA 160 Digimelt capillary melting point apparatus in vacuum sealed tubes and are corrected.

Chromatography: Analytical thin-layer chromatography was performed on Merck silica gel 60 F254 plates. TLC plates were visualized by exposure to ultraviolet (UV) light and aqueous KMnO4. Retention factor (R_f) values reported were measured using a 10 x 2 cm (length x width) TLC plate in a developing chamber containing the solvent system described. Manual flash column chromatography was performed using Silicycle SiliaFlash®P60 (40-63 µm particle size, 230-400 mesh) (SiO_2). Automated column chromatography was performed using a Biotage Selekt with Sfär Silica HC Duo (20 µm, 50 Å) columns. Unless otherwise specified, "silica" refers to P60 grade silica gel.

Solvents: Reaction solvent tetrahydrofuran (THF) (Fisher Scientific, not stabilized, HPLC grade,) was dried by percolation through two columns packed with neutral alumina under positive pressure of argon. Reaction solvent toluene (Fisher Scientific, ACS grade) was dried by percolation through a column packed with neutral alumina and a column packed with Q5 reactant (supported copper catalyst for scavenging oxygen) under a positive pressure of argon. Reaction solvent diethyl ether (Et_2O) (Fisher Scientific, BHT stabilized, ACS Grade) was dried by percolation through two columns packed with neutral alumina under positive pressure of argon. Reaction solvent dichloromethane (CH_2Cl_2) (Millipore Sigma, amylene-stabilized, HPLC) was dried by percolation through two columns packed with neutral alumina under positive pressure of argon. Reaction solvent *tert*-butanol (Fisher, ACS grade) was used as received. Reaction solvent water (H_2O) (Sigma Aldrich, HPLC Grade) was used as received. Reaction solvent dimethylformamide (DMF) (Fisher Scientific, ACS Grade) was dried by percolation through two columns packed with 4Å MS under positive pressure of argon. Unless noted, solvents for filtration, transfers and post-reaction workup were dichloromethane (CH_2Cl_2) (Fisher Scientific, amylene stabilized, ACS grade), ethyl acetate (EtOAc) (Fisher Scientific, ACS grade), diethyl ether (Et_2O) (Fisher Scientific, BHT stabilized, ACS grade), pentane (Fisher Scientific, HPLC grade). Solvents used for chromatography and recrystallizations were ethyl acetate (Fisher Scientific, ACS grade), hexanes (Sigma Aldrich, HPLC grade), diethyl ether (Fisher Scientific, BHT stabilized, ACS grade), pentane (Fisher Scientific, HPLC grade) and dichloromethane (CH_2Cl_2) (Fisher Scientific, amylene stabilized, ACS grade). Solvents used for chiral separations were hexanes (Sigma Aldrich, HPLC grade), 2-propanol (Sigma Aldrich, HPLC grade), water (Sigma Aldrich, HPLC grade) and acetonitrile (MeCN) (Sigma Aldrich, HPLC grade).

Commercial Reagents: Unless noted, the following commercial reagents were used as received without further purification: Sodium hexamethyldisilazide (NaHMDS) (Sigma Aldrich), 5-bromonicotinaldehyde (Ambeed), Trimethylphosphonium bromide (Oakwood Chemical), 4-bromotoluene (Sigma Aldrich), magnesium turnings (Fisher Scientific), iodine (Sigma Aldrich), 3,5-dichlorophenylethan-1-one (Aaron Chemicals), p-toluenesulfonic acid dihydrate (*p*-TsOH•$2H_2O$) (Fisher Scientific), 4-chlorobromobenzene (Sigma Aldrich), copper iodide (Strem Chemicals), propargyl alcohol (Oakwood Chemical), imidazole (Aldrich), *N*,*N*-4-dimethylaminopyridine (DMAP) (Sigma Aldrich), chlorotriethylsilane (TESCl) (Oakwood Chemical), sodium chloride (NaCl) (Fisher Scientific), ammonium chloride (NH_4Cl) (Fisher Scientific), potassium carbonate (K_2CO_3) (Fisher Scientific), sodium sulfate (Na_2SO_4) (Fisher Scientific), 2-chloroiodobenzene (Sigma Aldrich), 3,5-bistrifluoromethyl benzoic acid (Sigma Aldrich), thionyl chloride ($SOCl_2$) (Sigma Aldrich), 2-butyn-1-ol (Ambeed), triethylamine (NEt_3) (Sigma Aldrich), Lindlar's catalyst (Strem Chemicals), quinoline (Sigma Aldrich), hydrogen gas (Air Gas), 2-bromopropane (Fisher Scientific), pyridinium chlorochromate (PCC) (Sigma Aldrich), Lithium hexamethyldisilazide (1.0 M in ethylbenzene:THF) (LiHMDS) (Thermofisher Scientific Chemicals), *tert*-butyldimethylsilyl triflate (TBSOTf) (Oakwood Chemical), benzoic acid (Sigma Aldrich), 4-(phenylazo)diphenylamine (Sigma Aldrich), Celite (Fisher Scientific), 1-bromo-4-chlorobenzene (Sigma Aldrich), *tert*-butyl 3-methyl-4-oxopiperidine-1-carboxylate (Ambeed), concentrated hydrochloric acid (HCl, 37%, Fisher Scientific), *p*-toluenesulfonyl chloride (Sigma Aldrich), 1,4-diazabicyclo[2.2.2]octane (DABCO) (Sigma Aldrich), AD-mix β (Sigma Aldrich), potassium ferricyanide (K_3FeCN_6) (Sigma Aldrich), methanesulfonamide (Sigma Aldrich).

Alkenes Chosen for Experimental Validations

ALKENE SUBSTITUTION CLASSIFICATIONS

mono	geminal	trans
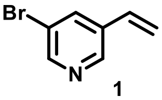 **1**	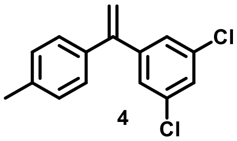 **4**	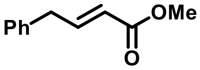 **8**
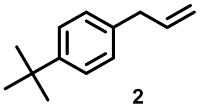 **2**	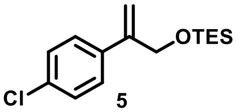 **5**	
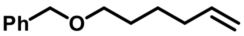 **3**	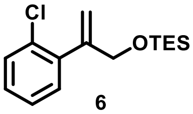 **6**	
	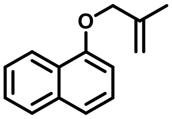 **7**	

ALKENE SUBSTITUTION CLASSIFICATIONS

cis	tri	tetra
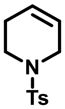 **9**	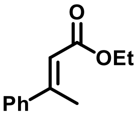 **12**	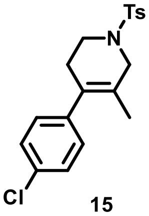 **15**
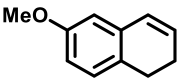 **10**	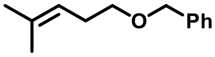 **13**	
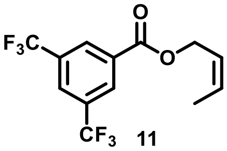 **11**	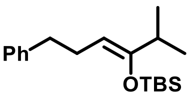 **14**	

Literature Preparation of Known Alkenes:

1-allyl-4-(tert-butyl)benzene (**2**)[1], ((hex-5-en-1-yloxy)methyl)benzene (**3**)[2], 1-((2-methylallyl)oxy)naphthalene (**7**)[3], methyl (E)-4-phenylbut-2-enoate (**8**)[4], 1-tosyl-1,2,3,6-tetrahydropyridine (**9**)[5], 6-methoxy-1,2-dihydronaphthalene (**10**)[6], ethyl (E)-3-phenylbut-2-enoate (**12**)[7], and (((4-methylpent-3-en-1-yl)oxy)methyl)benzene (**13**)[8] were prepared according to previously published protocols. For all novel alkenes **1**, **4**, **5**, **6**, **11**, **14**, **15**, the relevant characterization data as well as their preparation are described below.

Preparation of Novel Alkenes:

<u>Preparation of 3-Bromo-5-vinylpyridine (1):</u>

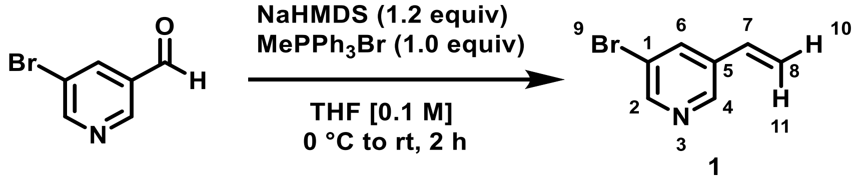

A 50-mL, flame-dried, Schlenk flask equipped with an egg-shaped magnetic stirring bar (3/4 inch x 3/8 inch, length x diameter) and sealed with a rubber septum was brought into a nitrogen-filled glovebox. NaHMDS (1.183 g, 6.45 mmol, 1.20 equiv) was added, the flask re-sealed with a rubber septum, brought out of the glovebox and placed under a nitrogen atmosphere. Then, anhydrous THF (30 mL) was added and the solution cooled to 0 °C in an ice bath. Trimethylphosphonium bromide (1.92 g, 5.38 mmol, 1.00 equiv) was added as a solid in one portion under a positive pressure of nitrogen. Upon addition of the phosphonium bromide, the solution turned into a bright yellow suspension and was allowed to stir for 1 h at 0 °C. Then, an anhydrous THF solution (24 mL) of 5-bromonicotinaldehyde (1.00 g, 5.38 mmol) was added dropwise over 5 min. After complete addition, the ice bath was removed, the mixture allowed to warm to rt (20 °C) over 15 min and stirred for an additional 45 min then quenched with saturated aqueous NH_4Cl (60 mL). The mixture was transferred to a 250-mL, separatory funnel with the assistance of Et_2O (20 mL), the phases were shaken, separated and the ether phase collected. The aqueous phase was further extracted with Et_2O (2 x 20 mL), the ether phases collected, combined and dried over Na_2SO_4 (5 g). The solution was filtered and concentrated under reduced pressure (7.5 mmHg, 34 °C) to give a crude oil (2.82 g).

The oil was then dissolved in CH_2Cl_2 (10 mL) and dry loaded onto Celite (4 g) by concentrating under reduced pressure (7.5 mmHg, 34 °C). The crude material was then purified via automated silica gel flash chromatography loading onto a pre-equilibrated silica column (Biotage Sfär HC Duo 50 g silica column, 22-mL fractions, 120 mL/min flow rate), eluting with hexanes/EtOAc (100:0) to hexanes/EtOAc (95:5) over 5 min to afford **1** (690 mg, 70% yield) as a colorless oil.

Data for 3-Bromo-5-vinylpyridine (**1**):

^{1}H NMR: (600 MHz, $CDCl_3$)
8.53 (dd, *J* = 18.6, 2.0 Hz, 2H, HC(2), HC(4)), 7.86 (t, *J* = 2.0 Hz, 1H, HC(6)), 6.64 (dd, *J* = 17.6, 11.0 Hz, 1H, HC(7)), 5.84 (d, *J* = 17.6 Hz, 1H, HC(11)), 5.44 (d, *J* = 11.0 Hz, 1H, HC(10)).

^{13}C NMR: (151 MHz, $CDCl_3$)
149.91 (C2), 146.45 (C4), 135.28 (C5), 134.83 (C7), 132.25 (C6), 121.02 (C1), 117.99 (C8).

IR: (neat)
3090 (w), 3036 (w), 3010 (w), 2988 (w), 1629 (w), 1575 (w), 1547 (w), 1439 (w), 1427 (m), 1400 (m), 1313 (w), 1295 (w), 1229 (m), 1205 (w), 1168 (w), 1147 (w), 1137 (w), 1095 (m), 1039 (w), 1018 (s), 985 (m), 917 (s), 882 (s), 842 (s), 718 (m), 691 (w), 662 (s), 597 (w), 594 (w), 566 (w).

HRMS: TOF MS ES+ m/z [M+H$^+$] calculated for C_7H_7NBr 183.9762; found 183.9762

TLC: R_f = 0.16, (hexanes/EtOAc, 95:5, UV/$KMnO_4$)

Purity: 97.5%, Q-NMR (Dimethyl sulfone used as the internal standard)

Preparation of 1,3-Dichloro-5-(1-(4-tolyl)vinyl)benzene (4):

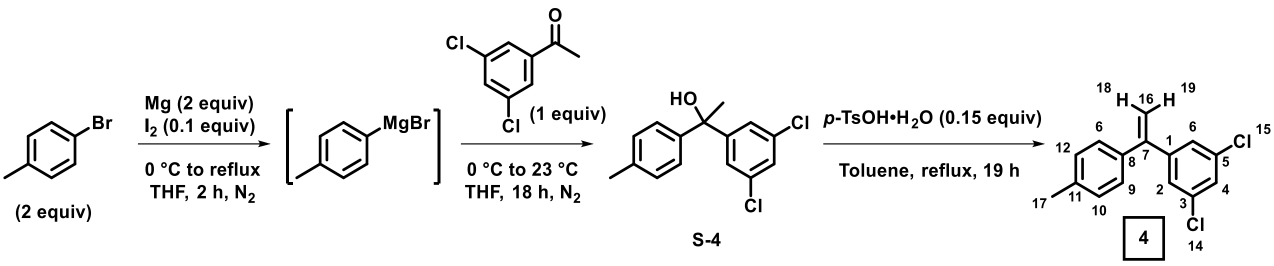

Step 1: To a 100-mL, flame-dried, Schlenk flask equipped with a stir bar (3/4 inch x 3/8 inch, length x diameter) under nitrogen atmosphere was added Mg turnings (386 mg, 15.9 mmol, 2.00 equiv) and iodine (201 mg, 0.79 mmol, 0.10 equiv). Then, stirring was initiated followed by the addition of anhydrous THF (30 mL). The mixture was cooled to 0 °C in an ice-bath and neat 4-bromotoluene (1.46 mL, 11.9 mmol, 1.50 equiv) was added dropwise. The ice-bath was removed, a reflux condenser was attached to the flask and the mixture heated to reflux for 2 h (after 2 h at reflux, the reaction mixture was a homogeneous, yellow solution). Then, the flask was cooled to 0 °C by submerging into an ice-bath and stirred for 15 min followed by dropwise addition of an anhydrous THF solution (10 mL) of 1-(3,5-dichlorophenyl)ethan-1-one (1.15 mL, 7.94 mmol). After the addition of the ketone, the ice-bath was removed and the reaction stirred at room temperature (approximately 20 °C) for 18 h. Upon completion, the reaction was quenched with 1 M HCl (25 mL) and transferred to a 125-mL, separatory funnel using Et_2O (25 mL). The phases were shaken, separated and the organic phase collected. The aqueous phase was further extracted with Et_2O (2 x 25 mL), the organic phases collected, combined, dried over Na_2SO_4 (10 g), and concentrated under reduced pressure (7.5 mmHg, 34 °C) to give a viscous, yellow oil.

The oil was dissolved in CH_2Cl_2 (20 mL), loaded onto Celite (1.3 g) and purified via automated silica gel flash chromatography on a pre-equilibrated silica gel column (Biotage Sfär HC Duo 25 g silica column, 22 mL fractions, 80 mL/min flow rate), eluting with hexanes/EtOAc (100:0) to hexanes/EtOAc (90:10) over 5 min to afford **S-4** (1.99 g, 89%, colorless oil).

Step 2: To a 250-mL, round-bottomed flask equipped with a stir bar (3/4 inch x 3/8 inch, length x diameter) under nitrogen atmosphere was added **S-4** (1.99 g, 7.07 mmol) followed by toluene (anhydrous, 16 mL). Then, *p*-TsOH•H_2O (202 mg, 1.06 mmol, 0.15 equiv) was added and the flask placed in a sand bath and heated to reflux for 19 h. The reaction was then cooled to room temperature (approximately 20 °C) and the solvent removed under rotary evaporation (7.5 mmHg,

34 ºC). The crude mixture was then dissolved in EtOAc (15 mL), transferred to a 125-mL, separatory funnel and the flask further rinsed with EtOAc (3 x 10 mL) for quantitative transfer. Brine (50 mL) was added, the phases were shaken, separated and the organic layer collected. The aqueous phase was further extracted with EtOAc (3 x 30 mL), the organic phases collected, combined, dried over Na_2SO_4 (10 g), filtered and concentrated to give a crude oil.

The crude oil was dissolved in CH_2Cl_2 (10 mL), loaded onto silica gel (5 g) and purified via flash column chromatography on a 10 cm x 3 cm (length x width) silica gel column collecting 25-mL fractions and eluting with hexanes to afford **4** (1.66 g, 89% yield) as a colorless oil.

Data for **4**:

^{1}H NMR: (600 MHz, $CDCl_3$)
7.31 (t, *J* = 1.9, 1.9 Hz, 1H, HC(4)), 7.22 (d, *J* = 1.9 Hz, 2H, HC(2), HC(6)), 7.20 – 7.15 (m, 4H, HC(9, 10, 12, 13), 5.49 (d, *J* = 0.9 Hz, 1H, HC(18)), 5.42 (d, *J* = 0.9 Hz, 1H, HC(19)), 2.38 (s, 3H, H_3C(17)).

^{13}C NMR: (151 MHz, $CDCl_3$)
147.92 (C7), 144.94 (C1), 138.30 (C11), 137.39 (C8), 134.87 (C3 and C5), 129.29 (C10 and C12), 128.15 (C9 and C13), 127.77 (C4), 126.88 (C2 and C6), 115.64 (C16), 21.34 (C17).

IR: (neat)
3087 (w), 3049 (w), 3025 (w), 2993 (w), 2921 (w), 2857 (w), 1609 (w), 1583 (m), 1557 (s), 1511 (m), 1435 (w), 1410 (w), 1380 (w), 1320 (m), 1305 (w), 1276 (w), 1213 (w), 1185 (w), 1163 (w), 1115 (w), 1102 (w), 1082 (w), 1060 (w), 1038 (w), 1020 (w), 995 (w), 905 (m), 883 (m), 857 (m), 824 (s), 800 (s), 737 (w), 718 (m), 678 (w), 652 (w), 630 (w), 588 (m), 568 (w).

HRMS: (EI+) m/z [M^+] calculated for $C_{15}H_{12}Cl_2$ 262.03161; found: 262.03217

TLC: R_f = 0.64, (hexanes/EtOAc, 95:5, UV/$KMnO_4$)

Purity: 99.02% (Q-NMR, Dimethyl sulfone used as the internal standard)

Preparation of ((2-(4-Chlorophenyl)allyl)oxy)triethylsilane (5):

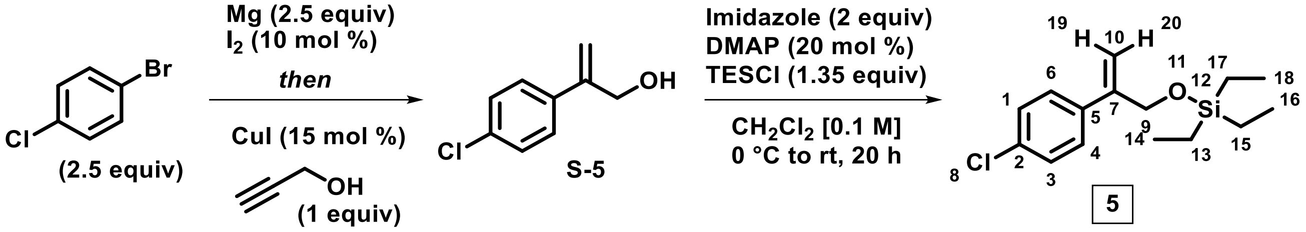

Step 1: To a 50-mL, flame-dried, Schlenk flask equipped with a stir bar (3/4 inch x 3/8 inch, length x diameter) under nitrogen atmosphere was added magnesium turnings (244 mg, 10 mmol, 2.5 equiv) and iodine (102 mg, 0.10 equiv) followed by anhydrous Et_2O (10 mL). Then, the flask was submerged into an ice bath and cooled to 0 °C and after 5 min, 4-chlorobromobenzene (1.92 g, 10 mmol, 2.5 equiv) was added in one portion under a positive pressure of nitrogen. The ice bath was removed and the reaction mixture heated to reflux and stirred for 2 h. The heating bath was removed, the reaction cooled to rt (20 °C) and then CuI (110 mg, 0.6 mmol, 0.15 equiv) was added in one portion under a positive pressure of nitrogen. The reaction mixture was stirred for 30 min at rt (20 °C) followed by dropwise addition of an anhydrous Et_2O solution (4 mL) of propargyl alcohol (230 mg, 4.00 mmol), heated to reflux, and stirred for 24 h. The reaction was then cooled to rt (20 °C) and carefully quenched with a saturated aqueous NH_4Cl solution (50 mL). The mixture was transferred to a 250-mL, separatory funnel with the assistance of Et_2O (3 x 15 mL rinses), the phases were shaken, separated and the ether phase collected. The aqueous phase was further extracted with Et_2O (2 x 50 mL), the ether phases were collected, combined and washed with brine (100 mL). The organic layer was collected, dried over Na_2SO_4 (10 g), filtered, and concentrated to give a crude yellow-green oil (800 mg).

The crude material was dissolved in CH_2Cl_2 (10 mL), loaded onto Celite (3 g) and purified via flash column chromatography on silica gel (column dimensions, 3 cm x 10 cm, width x length) collecting 25-mL fractions to give **S-5** as a light-yellow oil (510 mg, 75%) exhibiting spectral data identical to that previously reported.[9]

Step 2: To a 200-mL, round-bottomed flask equipped with a stir bar (3/4 inch x 3/8 inch, length x diameter) under ambient conditions (20 °C, open to air) **S-5** (510 mg, 3.02 mmol) was added followed by CH_2Cl_2 (20 mL). The reaction mixture was then cooled to 0 °C and stirred for 5 min. Then, DMAP (146 mg, 1.21 mmol, 0.40 equiv) and imidazole (824 mg, 12.10 mmol, 4.00 equiv) were added. The mixture was allowed to stir for 5 min then TESCl (1.38 mL, 8.12 mmol,

2.70 equiv) was added dropwise over 2 min and the reaction allowed to stir for 5 min at 0 °C followed by removal of the ice bath and warming to rt (20 °C) and stirred for 20 h. Then, brine (50 mL) was added and the mixture transferred to a 125-mL, separatory funnel with the assistance of CH_2Cl_2 (3 x 10 mL rinses), the phases shaken, separated and the organic layer was collected. The aqueous phase was further washed with CH_2Cl_2 (2 x 20 mL), the organic phases collected, combined, dried over Na_2SO_4 (10 g), filtered and concentrated to give a crude oil.

The crude material was dissolved in CH_2Cl_2 (20 mL), loaded onto Celite (2 g), and purified via automated silica gel flash chromatography on a pre-equilibrated silica gel column (Biotage Sfär HC Duo 25 g silica column, 9-mL fractions, 80 mL/min flow rate), eluting with hexanes over 2 min to afford **5** (800 g, 94% yield) as a colorless oil.

Data for **5**:

^{1}H NMR: (600 MHz, $CDCl_3$)
7.35 (dt, J = 9.0, 2.5, 2.5 Hz, 2H, HC(1, 3)), 7.29 (dt, J = 9.0, 2.5, 2.5 Hz, 2H, HC(4, 6)), 5.41 (dq, J = 6.2, 1.5 Hz, 2H, HC(19, 20)), 4.49 (t, J = 1.5 Hz, 2H, H_2C(9), 0.97 (t, J = 7.9 Hz, 9H, H_3C(14, 16, 18), 0.64 (q, J = 7.9 Hz, 6H, H_2C(13, 15, 17)).

^{13}C NMR: (151 MHz, $CDCl_3$)
146.07 (C7), 137.66 (C5), 133.57 (C2), 128.57 (C4) and (C6), 127.52 (C1) and (C3), 112.39 (C10), 64.53 (C9), 6.92 (C13, C15, and C17), 4.64 (C14, C16, and C18).

IR: (neat)
2955 (w), 2938 (w), 2911 (w), 2876 (w), 1635 (w), 1595 (w), 1493 (m), 1458 (w), 1413 (w), 1379 (w), 1314 (w), 1301 (w), 1239 (w), 1183 (w), 1129 (m), 1092 (s), 1075 (s), 1012 (s), 974 (w), 962 (w), 946 (w), 905 (m), 835 (s), 819 (m), 774 (m), 767 (w), 726 (s), 690 (m), 647 (w), 634 (w), 593 (w), 556 (w), 534 (w).

HRMS: (CI+) m/z $[M+H]^+$ calculated for $C_{15}H_{24}ClOSi$ 283.12904; found 283.12796

TLC: R_f = 0.58, (hexanes/EtOAc, 95:5, UV/KMnO4)

Purity: 96.9%, (Q-NMR, Dimethyl sulfone used as the internal standard)

Preparation of ((2-(2-Chlorophenyl)allyl)oxy)triethylsilane (6):

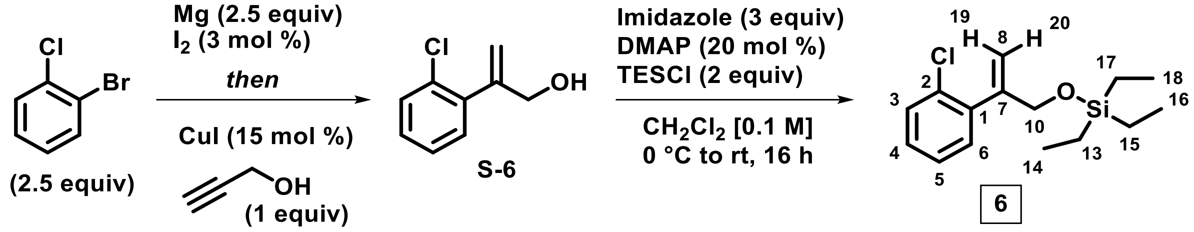

Step 1: To a 50-mL, flame-dried, Schlenk flask equipped with a stir bar (3/4 inch x 3/8 inch, length x diameter) under nitrogen atmosphere was added magnesium turnings (542 mg, 22.3 mmol, 2.5 equiv) and iodine (60 mg, 0.24 mmol, 0.03 equiv) followed by anhydrous Et_2O (24 mL). Then, the flask was submerged into an ice bath and cooled to 0 °C and after 5 min, neat 2-chloroiodobenzene (2.72 mL, 22.3 mmol, 2.5 equiv) was added dropwise over 5 min (vigorous refluxing is observed if added too quickly). The ice bath was removed, a reflux condenser was attached, and the reaction mixture heated to reflux and stirred for 1 h. The heating bath was removed, the reaction cooled to rt (20 °C) and then CuI (258 mg, 1.35 mmol, 0.15 equiv) was added in one portion under a positive pressure of nitrogen. The reaction mixture was stirred for 30 min at rt (20 °C) followed by dropwise addition of an anhydrous Et_2O solution (10 mL) of propargyl alcohol (0.51 mL, 8.92 mmol), heated to reflux, and stirred for 13 h. The reaction was then cooled to rt (20 °C) and carefully quenched with a saturated aqueous NH_4Cl solution (50 mL). The mixture was transferred to a 250-mL, separatory funnel with the assistance of EtOAc (5 x 10 mL rinses), the phases were shaken, separated and the ether phase collected. The aqueous phase was further extracted with EtOAc (2 x 30 mL), the organic phases were collected, combined and washed with brine (100 mL). The organic layer was collected, dried over Na_2SO_4 (10 g), filtered, and concentrated to give a crude, yellow oil.

The crude material was dissolved in CH_2Cl_2 (10 mL), loaded onto silica gel (10 g) and purified via automated silica gel flash chromatography on a pre-equilibrated silica gel column (100 g silica column, 22-mL fractions, 120 mL/min flow rate), eluting with hexanes/EtOAc (100:0) to hexanes/EtOAc (80:20) over 16 min to afford **S-6** (576 mg, 38% yield) as a yellow oil exhibiting spectral data identical to that previously reported.[10]

Step 2: To a 200-mL, round-bottomed, flask equipped with a stir bar (3/4 inch x 3/8 inch, length x diameter) under ambient conditions (20 °C, open to air) **S-6** (235 mg, 1.39 mmol) was added followed by CH_2Cl_2 (14 mL). The reaction mixture was then cooled to 0 °C and stirred for

5 min. Then, DMAP (34 mg, 0.28 mmol, 0.2 equiv) and imidazole (285 mg, 4.18 mmol, 3 equiv) were added. The mixture was allowed to stir for 5 min then TESCl (0.47 mL, 2.79 mmol, 2.0 equiv) was added dropwise over 2 min and the reaction allowed to stir for 5 min at 0 °C followed by removal of the ice bath and warming to rt (20 °C) and stirred for 16 h. Then, brine (50 mL) was added and the mixture transferred to a 125-mL, separatory funnel with the assistance of CH_2Cl_2 (5 x 5 mL rinses), the phases shaken, separated and the organic layer was collected. The aqueous phase was further washed with CH_2Cl_2 (3 x 20 mL), the organic phases collected, combined, dried over Na_2SO_4 (5 g), filtered and concentrated to give an oil.

The crude material was dissolved in CH_2Cl_2 (10 mL), loaded onto Celite (2 g), and purified via automated silica gel flash chromatography on a pre-equilibrated silica gel column (25 g silica column, 9-mL fractions, 80 mL/min flow rate), eluting with hexanes/EtOAc (100:0) to hexanes/EtOAc (90:10) over 6 min to afford **6** (301 mg, 76% yield) as a colorless oil.

Data for **6**:

^{1}H NMR: (600 MHz, $CDCl_3$)
7.38 – 7.33 (m, 1H, HC(6)), 7.24 – 7.19 (m, 3H, HC(4, 5, 6), 5.58 (dd, *J* = 1.7, 1.7 Hz, 1H, HC(19)), 5.11 (dd, *J* = 1.7, 1.7 Hz, 1H, HC(20)), 4.38 (dd, *J* = 1.7, 1.7 Hz, 2H, H_2C(10)), 0.96 (t, *J* = 8.0 Hz, 9H, H_3C(14, 16, 18), 0.63 (q, *J* = 8.0 Hz, 6H, H_2C(13, 15, 17)).

^{13}C NMR: (151 MHz, $CDCl_3$)
147.53 (C7), 139.52 (C1), 132.67 (C2), 131.09 (C6), 129.58 (C3), 128.79 (C4), 126.68 (C5), 64.86 (C10), 6.90 (C13, C15, and C17), 4.61 (C14, C16, and C18).

IR: (neat)
2954 (w), 2937 (w), 2911 (w), 2876 (w), 1465 (w), 1458 (w), 1439 (w), 1429 (w), 1414 (w), 1378 (w), 1263 (w), 1239 (w), 1133 (m), 1087 (s), 1045 (w), 1035 (w), 1005 (m), 974 (w), 945 (w), 910 (m), 864 (w), 824 (m), 775 (m), 735 (s), 728 (s), 701 (m), 689 (m), 671 (m), 619 (w), 605 (w), 564 (w), 551 (w), 535 (w).

HRMS: (CI+) m/z $[M+H]^+$ calculated for $C_{15}H_{24}ClOSi$ 283.12850; found 283.12860

TLC: R_f = 0.52, (hexanes/EtOAc, 95:5, UV/$KMnO_4$)

Purity: 98.12%, (Q-NMR, Dimethyl sulfone used as the internal standard)

Preparation of (*Z*)-But-2-en-1-yl 3,5-bis(trifluoromethyl)benzoate (11):

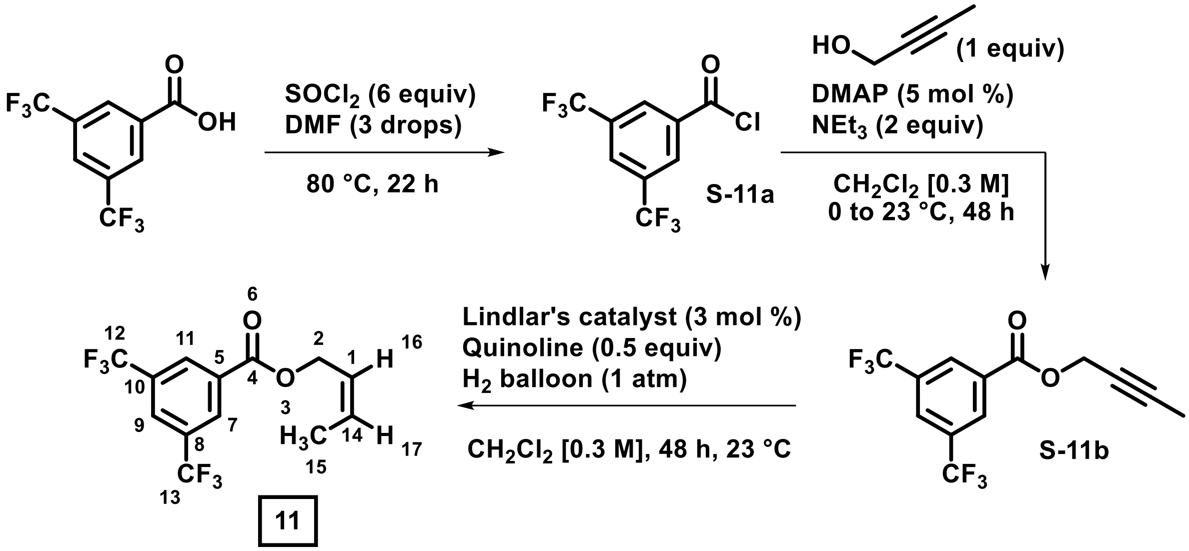

Step 1: To a 250-mL, flame-dried, 2-necked flask equipped with an egg-shaped stir bar (3/4 inch x 3/8 inch, length x diameter) under nitrogen atmosphere was added 3,5-bistrifluoromethylbenzoic acid (3.0 g, 11.62 mmol) followed by thionyl chloride (5 mL). Then, anhydrous DMF (3 drops) was added and the reaction brought to reflux temperature (80 °C) and allowed to stir for 22 h. Then, the thionyl chloride was removed under reduced pressure (22 °C, 22.5 mmHg) and the resulting crude yellow oil **S-11a** (3.20 g) was used as is in the subsequent esterification.

Step 2: To a 50-mL, flame-dried, Schlenk flask equipped with an egg-shaped stir bar (3/4 inch x 3/8 inch, length x diameter) was added **S-11a** (3.20 g, 11.6 mmol, 1.05 equiv). The flask was then evacuated and backfilled with nitrogen atmosphere (3x). Then, anhydrous CH_2Cl_2 (38 mL) was added and the solution cooled to 0 °C in an ice bath. Then, NEt_3 (3.1 mL, 22.2 mmol, 2.00 equiv) was added and the mixture stirred for 1 min followed by dropwise addition of 2-butyn-1-ol (0.83 mL, 11.00 mmol). Then, DMAP (67 mg, 0.55 mmol, 0.05 equiv) was added under a positive pressure of nitrogen and the reaction allowed to warm to rt (20 °C) by removal of the ice bath and stirred for 24 h. The reaction was quenched with a saturated aqueous solution Na_2CO_3 (50 mL). The reaction mixture was transferred to a 500-mL separatory funnel with the assistance of Et_2O (4 x 50 mL rinses), shaken, separated, and the organic layer collected. The aqueous phase was further extracted with Et_2O (3 x 100 mL) and the organic phases collected, combined, dried over Na_2SO_4 (10 g), filtered and concentrated to give a light-yellow oil (3.73 g).

The crude material was dissolved in CH_2Cl_2 (20 mL), loaded onto silica gel (5 g), and purified by flash column chromatography on silica gel (column dimensions, 6 cm x 24 cm, width x length) with hexanes (200 mL) and hexanes/EtOAc (95:5) (1 L) collecting 45-mL fractions to give **S-11b** (3.40 g, 99% yield) as a colorless oil.

Step 3: To a 100-mL, round-bottomed flask equipped with a stir bar (3/4 inch x 3/8 inch, length x diameter) was added **S-11b** (1.8 g, 5.8 mmol) and dissolved in anhydrous CH_2Cl_2 (20 mL). Then, quinoline (0.34 mL, 2.9 mmol, 0.5 equiv) and Lindlar's catalyst (5% w/w Pd mixed with $CaCO_3$ and doped with Pb, 4.08 g) were added. The flask was stoppered with a rubber septum and then sparged with a hydrogen balloon (1 atm) for 10 min making sure that the hydrogen bubbled through the heterogeneous mixture. Once sparging was complete, the hydrogen balloon was replenished, and the reaction was allowed to stir at rt (20 °C) for 48 h. Then, the reaction was carefully sparged with nitrogen for 10 min and reaction mixture concentrated under reduced pressure (7.5 mmHg, 34 °C). The crude material was diluted with hexanes (20 mL) and passed through a pad of silica gel (3 cm x 7 cm, width x length) using hexanes/EtOAc (95:5) (1 L). The material was concentrated under reduced pressure (7.5 mmHg, 34 °C) to give a light-yellow oil (1.58 g).

The oil was dissolved with CH_2Cl_2 (10 mL), loaded onto Celite (5 g), and purified via automated silica gel flash chromatography on a pre-equilibrated silica gel column (100 g silica column, 22-mL fractions, 120 mL/min flow rate), eluting with hexanes/CH_2Cl_2 (100:0) to hexanes/CH_2Cl_2 (95:5) over 20 min to afford **11** (1.30 g, 72% yield) as a colorless oil.

NOTE: *The material was found to degrade readily upon storage and is best used immediately after isolation.*

Data for **11**:

^{1}H NMR: (600 MHz, $CDCl_3$)
8.49 (s, 2H, HC(7, 11)), 8.06 (s, 1H, HC(9)), 5.84 (dqt, J = 10.6, 7.0, 7.0, 7.0, 1.6, 1.6 Hz, 1H, HC(17)), 5.70 (dtq, J = 10.6, 7.0, 7.0, 1.6, 1.6, 1.6 Hz, 1H, HC(16)), 4.97 (dt, J = 7.0, 1.6, 1.6 Hz, 2H, $H_2C(2)$), 1.79 (ddd, J = 6.9, 1.6, 0.7, 0.7 Hz, 3H, $H_3C(15)$).

^{13}C NMR: (151 MHz, $CDCl_3$)
164.04 (C4), 132.68 (C5), 132.31 (q, J = 34.2 Hz, C8 and C10), 131.05 (C14),

129.93 (q, *J* = 3.8 Hz, C7 and C11), 126.43 (hept, *J* = 3.8 Hz, C9), 123.50 (C1), 123.04 (q, *J* = 272.9 Hz, C12 and C13), 61.83 (C2), 13.39 (C15).

IR: (neat)
2981 (w), 2972 (w), 1731 (m), 1622 (w), 1458 (w), 1390 (w), 1378 (w), 1347 (w), 1277 (s), 1242 (s), 1175 (s), 1129 (s), 1016 (w), 1003 (w), 991 (w), 944 (m), 912 (m), 844 (w), 770 (m), 716 (w), 699 (m), 681 (s), 579 (w).

HRMS: EI+ m/z [M^+] calculated for $C_{13}H_{10}O_2F_6$ 312.05848; found 312.05734

TLC: R_f = 0.46, (hexanes/EtOAc, 95:5, UV/$KMnO_4$)

Purity: 95.92%, (Q-NMR, Dimethyl sulfone used as the internal standard)

Preparation of (*Z*)-*tert*-Butyldimethyl((2-methyl-6-phenylhex-3-en-3-yl)oxy)silane (14):

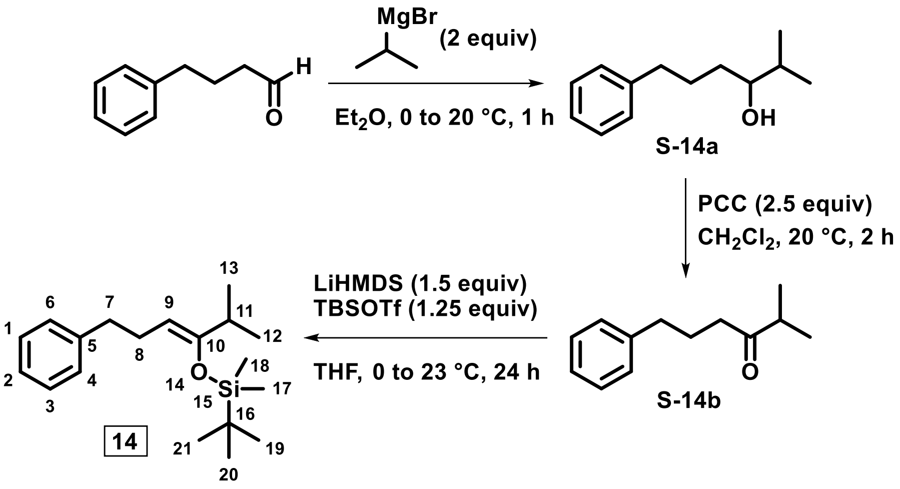

Preparation of isopropylmagnesium bromide stock solution: To a 250-mL, flame-dried, Schlenk flask equipped with a stir bar (3/4 inch x 3/8 inch, length x diameter) under nitrogen atmosphere was added magnesium turnings (2.22 g, 89 mmol, 1.10 equiv) and iodine (220 mg, 0.81 mmol, 1 mol %) followed by anhydrous Et_2O (86 mL). The mixture was stirred until the purple-pink color dissipated and then cooled to 0 °C by submerging the flask into an ice bath. After stirring for 10 min, 2-bromopropane (7.6 mL, 81.00 mmol, 1.00 equiv) was added dropwise over 2 min, the reaction warmed to rt (20 °C) and stirred for 2 h. The stock solution was titrated against

benzoic acid (24 mg) with 4-(phenylazo)diphenylamine as the indicator and was found to be 0.434 M

Step 1: To a 250-mL, flame-dried, 2-necked flask stoppered with a rubber septum, a gas-flow adapter and equipped with a stir bar (3/4 inch x 3/8 inch, length x diameter) under nitrogen atmosphere was added a freshly prepared isopropylmagnesium bromide solution (62 mL, 0.434 M, 26.9 mmol, 2.00 equiv). The solution was cooled to 0 °C in an ice bath and stirred for 15 min. Separately, in a 20-mL, flame-dried, scintillation vial under nitrogen atmosphere was prepared an anhydrous diethyl ether solution (20-mL) of 4-phenylbutanal (1.98 g, 13.4 mmol). The aldehyde solution was then added dropwise over 5 min to the reaction flask. The vial containing the aldehyde was rinsed with additional diethyl ether (2 x 5 mL) and also added to the reaction mixture at 0 °C. The ice-bath was then removed, the reaction allowed to warm to rt (20 °C) and stirred for 1 h. Then, the reaction was quenched by the dropwise addition of 1 M aqueous HCl (100-mL) at 0 °C and the pH adjusted to 1 (gelation occurs if insufficient HCl is added). The mixture was transferred to a 250-mL, separatory funnel with the assistance of EtOAc (5 x 5 mL rinses) and then the phases were shaken, separated and the aqueous layer further extracted with EtOAc (3 x 20 mL). The organic layers were combined, washed with brine (50 mL), dried over Na_2SO_4 (10 g), filtered, and concentrated under reduced pressure (7.5 mmHg, 34 °C) to give a clear, light-yellow oil.

The crude material was dissolved in CH_2Cl_2 (20 mL), loaded onto silica gel (5 g), and purified via flash column chromatography on silica gel (column dimensions, 6 cm x 16 cm, width x length) with hexanes (200 mL) and hexanes/EtOAc (95:5) (1 L) collecting 20-mL fractions to give **S-14a** (2.03 g, 79% yield) as a colorless oil.

Step 2: To a 200-mL, round-bottomed flask equipped with a stir bar (3/4 inch x 3/8 inch, length x diameter) under ambient atmosphere was added **S-14a** (2.03 g, 10.6 mmol) followed by CH_2Cl_2 (70 mL) and Celite (10 g). Then, pyridinium chlorochromate (5.77 g, 26.8 mmol, 2.5 equiv) was added portion-wise under moderate stirring (400~500 rpm) and stirred at rt (20 °C) for 2 h. Then, the reaction mixture was filtered through a short plug of silica gel (7 cm x 5 cm, width x length). The filter cake was further washed with CH_2Cl_2 (1 L) and the filtrate concentrated under reduced pressure (7.5 mmHg, 34 °C) to afford **S-14b** (1.92 g, 95.6% yield) as a clear light-yellow oil exhibiting spectral data identical to that previously reported.[11]

Step 3: To a 50-mL, flame-dried, Schlenk flask equipped with a stir bar (3/4 inch x 3/8 inch, length x diameter) under nitrogen atmosphere was added a solution of LiHMDS (7.10 mL, 1

M in ethylbenzene/THF, 7.10 mmol, 1.50 equiv). The reaction flask was submerged into an ice bath, cooled to a 0 °C and stirred for 15 min. Separately, in a 20-mL, flame-dried, scintillation vial under nitrogen atmosphere was prepared an anhydrous THF solution (14 mL) of ketone **S-14b** (900 mg, 4.73 mmol). The solution of **S-14b** was then added dropwise over 5 min to the reaction flask. The vial containing was rinsed with additional anhydrous THF (2 mL) and added to the reaction mixture. The reaction was kept at 0 °C for 2 h and then neat TBSOTf (1.36 mL, 5.91 mmol, 1.25 equiv) was added dropwise over 2 min. The reaction was kept at 0 °C for 10 h and then allowed to warm to 15 °C over 12 h. The reaction was quenched by the addition of a saturated aqueous solution of $NaHCO_3$ (30 mL) and extracted with EtOAc (3 x 20 mL). The organic phases were combined, washed with brine (50 mL), dried over Na_2SO_4 (10 g), filtered, and concentrated under reduced pressure (7.5 mmHg, 34 °C) to give a crude brown oil (1.7 g).

The crude material was dissolved in CH_2Cl_2 (20 mL), loaded onto Celite (7.5 g), and purified via automated silica gel flash chromatography on neutralized silica gel (75 g silica column pre-treated with 10% NEt_3/hexanes, 9-mL fractions, 60 mL/min flow rate), eluting with hexanes over 15 min to afford **14** (1.26 g, 88% yield, 10:1 *Z*/*E*) as a colorless oil.

Data for **14** (*Z*-isomer):

^{1}H NMR: (500 MHz, $CDCl_3$)
7.28 (m, 2H, HC(4, 6)), 7.23 – 7.14 (m, 3H, HC(1, 2, 3)), 4.48 (tdd, *J* = 6.9, 2.6, 1.0 Hz, 1H, HC(9)), 2.63 (td, *J* = 8.4, 6.8, 2.6 Hz, 2H, $H_2C(7)$), 2.34 (qdd, *J* = 6.8, 2.6, 1.1 Hz, 2H, $H_2C(8)$), 2.14 (dtdd, *J* = 13.5, 9.3, 4.5, 2.1 Hz, 1H, HC(11)), 1.04 (dd, *J* = 6.7, 2.6 Hz, 6H, $H_2C(12, 13)$), 0.97 (d, *J* = 2.5 Hz, 9H, $H_3C(19, 20, 21)$), 0.12 (d, *J* = 2.6 Hz, 6H, $H_2C(17, 18)$).

^{13}C NMR: (126 MHz, $CDCl_3$)
156.58 (C10), 142.70 (C5), 128.61 (C1 and C3), 128.32 (C4 and C6), 125.71 (C2), 104.24 (C9), 36.43 (C7), 34.26 (C11), 27.26 (C8), 26.08 (C19, C20, and C21), 21.07 (C12 and C13), 18.56 (C16), -3.81 (C17 and C18).

IR: (neat)
3028 (w), 2959 (m), 2929 (m), 2896 (w), 2886 (w), 2858 (w), 1668 (w), 1605 (w), 1496 (w), 1472 (w), 1462 (w), 1454 (w), 1407 (w), 1382 (w), 1369 (w), 1361 (w), 1354 (w), 1335 (w), 1303 (w), 1253 (m), 1228 (w), 1207 (w), 1133 (s), 1102 (m),

1077 (w), 1044 (w), 1030 (w), 1019 (w), 1005 (w), 981 (w), 938 (w), 857 (m), 835 (s), 804 (m), 776 (s), 746 (m), 697 (s), 672 (w), 648 (w), 592 (w), 566 (w), 534 (w).

HRMS: ES+ m/z [M+H$^+$] calculated for $C_{19}H_{33}OSi$ 305.2301; found 305.2298

TLC: R_f = 0.11 (hexanes, UV/$KMnO_4$)

Purity: 97.29% (Q-NMR, Dimethyl sulfone used as the internal standard)

Preparation of 4-(4-Chlorophenyl)-5-methyl-1-tosyl-1,2,3,6-tetrahydropyridine (15):

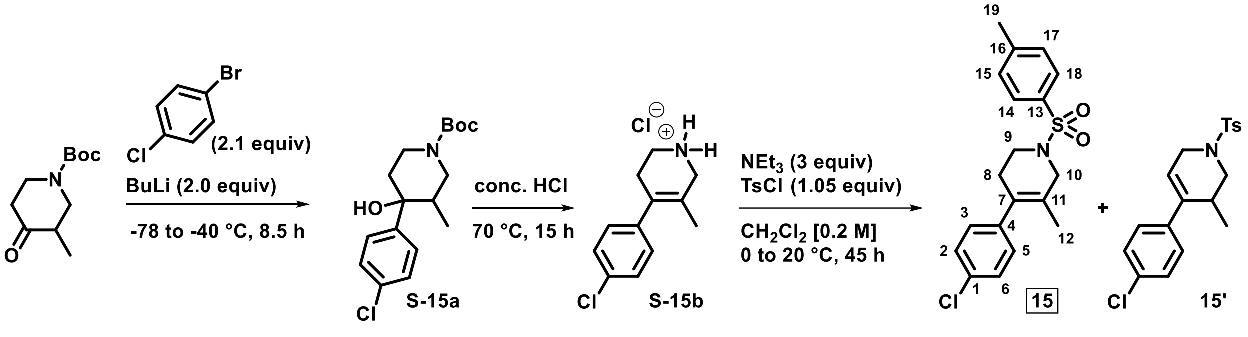

Step 1: To a 100-mL, flame-dried, Schlenk flask equipped with a stir bar (3/4 inch x 3/8 inch, length x diameter) under nitrogen atmosphere was added 1-bromo-4-chlorobenzene (3.80 g, 20 mmol, 2.10 equiv) followed by anhydrous THF (20 mL). The flask was then submerged into a dry ice-acetone bath and cooled to -78 °C. After stirring for 5 min, freshly titrated BuLi (12 mL, 1.55 M in hexanes) was added dropwise to the reaction mixture and stirred for 90 min. Then, a solution of *tert*-butyl 3-methyl-4-oxopiperidine-1-carboxylate (2.0 g, 9.4 mmol) in anhydrous THF (13 mL) was added dropwise and the reaction stirred at -78 °C for 2 h, then allowed to slowly warm to -40 °C (over 5 h) and quenched with a saturated NH_4Cl solution (50 mL). The mixture was transferred to a 250-mL, separatory funnel with the assistance of Et_2O (50 mL). The layers were shaken, separated, and the organic phase collected. The aqueous phase was further extracted with Et_2O (3 x 30 mL), the organic layers were collected, dried over Na_2SO_4 (10 g), filtered, and concentrated under reduced pressure (7.5 mmHg, 34 °C) to give a colorless oil.

The oil was then treated with hexanes (100 mL) and stirred for 5 min. A seed crystal was then added to induce precipitation and the mixture stored in a -20 °C freezer for 36 h which resulted in the precipitation of a white solid. The solids were then filtered through a fritted medium porosity

Buchner funnel, the solid further washed with cold hexanes (3 x 30 mL) and dried under air. Then, the solid was transferred to a 20-mL scintillation vial and dried further under vacuum (0.1 mmHg, 20 °C) which gave **S-15a** (1.69 g) as a white solid. The mother liquor from the recrystallization was loaded onto Celite (5 g) and purified via automated silica gel flash chromatography (25 g silica column, 22-mL fractions, 80 mL/min flow rate), eluting with a hexanes/EtOAc (100:0) to hexanes/EtOAc (80:20) gradient over 12 min to afford additional **S-15a** (810 mg). In total, **15** was obtained as a white solid (2.50 g, 82% yield) exhibiting spectra identical to that of the previously published material.[12]

Step 2: To a 50-mL, round-bottomed flask equipped with a stir bar (3/4 inch x 3/8 inch, length x diameter) was added **S-15a** (1.20 g, 3.68 mmol) and then concentrated HCl (aqueous 35-37% reagent grade HCl, 6.8 mL) was added carefully. Vigorous bubbling was observed at 20 °C, the reaction mixture was stirred for 5 min until the solution turned mostly homogeneous and the bubbling ceased. Then, the reaction mixture was placed into a heating bath and heated to 70 °C and stirred for 15 h. Then, the aqueous HCl was removed under reduced pressure (7.5 mmHg, 60 °C). The crude solid was further rinsed with toluene (2 x 10 mL) and the solvent evaporated under reduced pressure (7.5 mmHg, 60 °C) to afford a **S-15b** as a light-brown solid (900 mg). The material was used without further purification in the subsequent step.

Step 3: To a 100-mL, flame-dried, Schlenk flask equipped with a stir bar (3/4 inch x 3/8 inch, length x diameter) under nitrogen atmosphere was added **S-15b** (756 mg, 3.10 mmol) followed by anhydrous CH_2Cl_2 (31 mL). Then, anhydrous NEt_3 (1.29 mL, 12.10 mmol, 3.00 equiv) was added to the reaction mixture and the reaction was cooled to 0 °C in an ice bath and stirred for 5 min. Then, 4-toluenesulfonyl chloride (618 mg, 3.24 mmol, 1.05 equiv) was added as a solid in one portion under a positive pressure of nitrogen. After stirring for 5 min at 0 °C, the ice bath was removed, the reaction allowed to warm to 20 °C and stirred for 24 h. Then, the reaction was washed with a saturated, aqueous $NaHCO_3$ solution (20 mL), transferred to a 125-mL, separatory funnel with the assistance of CH_2Cl_2 (5 x 10 mL rinses). The phases were shaken, separated and the organic layer collected. The aqueous layer was further extracted with CH_2Cl_2 (3 x 30 mL), the phases were separated, the organic layers collected and combined, dried over Na_2SO_4 (5 g), filtered and concentrated under reduced pressure (7.5 mmHg, 34 °C) to give an oil containing a 4:1 mixture of tetrasubstituted/trisubstituted alkene (**15**:**15’**). The trisubstituted alkene can be purged and the desired tetrasubstituted alkene isolated as described below.

The oil was triturated by dissolving in CH_2Cl_2 (2 mL) followed by the addition of pentane (8 mL) which resulted in the precipitation of an off-white solid (564 mg) enriched in the trisubstituted alkene (1.85:1 ratio of **15**:**15'**). The mother liquor was collected, loaded onto Celite (2 g), and purified via automated silica gel flash chromatography (50 g silica column, 9-mL fractions, 120 mL/min flow rate), eluting with a hexanes/EtOAc (100:0) to hexanes/EtOAc (80:20) gradient over 10 min to afford **15** (400 mg) as a white solid. Additional material was obtained by subjecting the initial off-white solid precipitate to three iterative recrystallizations with Pentane/CH_2Cl_2, 4:1 followed by automated column chromatography using the same conditions as described above to afford more of **15** (240 mg). In total, **15** was obtained as a white solid (640 mg, 57% yield).

Data for **15**:

^{1}H NMR: (600 MHz, $CDCl_3$)
7.72 (d, J = 8.1 Hz, 2H, HC(14, 18), 7.35 (d, J = 8.1 Hz, 2H, HC(15, 17), 7.27 (d, J = 8.4 Hz, 2H, HC(3, 5), 6.96 (d, J = 8.4 Hz, 2H, HC(2, 6), 3.55 (t, J = 2.5 Hz, 2H, H_2C(10)), 3.27 (t, J = 5.8 Hz, 2H, H_2C(9)), 2.44 (s, 3H, H_3C(19)), 2.40 (tq, J = 5.8, 2.5 Hz, 2H, H_2C(8)), 1.53 (s, 3H, H_3C(12)).

^{13}C NMR: (151 MHz, $CDCl_3$)
143.79 (C16), 140.03 (C13), 133.35 (C4), 132.72 (C1), 129.98 (C7), 129.82 (C15 and C17), 129.65 (C2 and C6), 128.59 (C3 and C5), 127.93 (C14 and C18), 126.02 (C11), 49.17 (C10), 43.48 (C9), 31.30 (C8), 21.68 (C19), 17.78 (C12).

IR: (neat)
1341 (m), 1165 (s), 1089 (w), 1027 (w), 1015 (w), 760 (w), 665 (m), 587 (m), 550 (w).

HRMS: ES+ m/z [M+H$^+$] calculated for $C_{19}H_{21}NO_2SCl$ 362.0982; found 362.0979

TLC: R_f = 0.44, (hexanes/EtOAc, 80:20, UV/$KMnO_4$)

m.p: 143.2 °C – 143.8 °C (sealed tube)

Purity: 97.58% (Q-NMR, Dimethyl sulfone used as the internal standard)

Synthesis of Racemic Standards – Racemic Dihydroxylations

<u>General Procedure for Racemic Dihydroxylations</u>

All racemic dihydroxylations were conducted on a 1.00 mmol scale

General Procedure A – For Liquid Alkenes:

A 20-mL, scintillation vial equipped with a cylindrical stir bar (2 cm x 1 cm, length x diameter) at 20 °C and under ambient atmosphere was charged with K_3FeCN_6 (988 mg, 3.00 mmol, 3.00 equiv), K_2CO_3 (414 mg, 3.00 mmol, 3.00 equiv), DABCO (56 mg, 0.50 mmol, 0.50 equiv), K_2OsO_4•$2H_2O$ (18.4 mg, 0.05 mmol, 0.05 equiv). Then, *t*-BuOH (4.0 mL, ACS grade) and H_2O (5 mL, HPLC grade) were added and the vial sealed with a green Teflon-lined septum cap. The mixture was stirred vigorously at 700 rpm on a magnetic stir plate until all the solids were dissolved to afford a light orange solution. To a separate 2-dram vial was charged the alkene (1.00 mmol) and *t*-BuOH (0.50 mL, ACS grade). The solution of alkene was then added to the scintillation vial, the 2-dram vial rinsed with a further 0.50 mL of *t*-BuOH, added to the scintillation vial and sealed with a green Teflon-lined cap. The reactions were stirred until completion and monitored via TLC. Upon completion, sodium sulfite (1.50 g) was added as a solid, the mixture stirred for 1 h and then transferred to a 125-mL, separatory funnel with the assistance of EtOAc or CH_2Cl_2 (5 x 5 mL rinses). Brine (25 mL) was added, the phases were shaken, separated, and the organic layer collected. The aqueous layer was further extracted with EtOAc or CH_2Cl_2 (4 x 20 mL), the organic phases collected, combined, dried over Na_2SO_4 (5 g), filtered, and concentrated under reduced pressure (7.5 mmHg, 34 °C).

The crude material was dissolved in CH_2Cl_2 (10 mL), loaded onto Celite (1 g) and purified via automated silica gel flash chromatography on a pre-equilibrated Biotage silica gel column.

General Procedure B – for solid alkenes:

If the alkene was a solid, a *t*-BuOH solution of the alkene was not prepared but rather directly added to the 20-mL scintillation vial in one portion. In these cases, *t*-BuOH (5 mL) was added to the scintillation vial to prepare the homogeneous solution containing K_3FeCN_6, K_2CO_3, DABCO, and K_2OsO_4•$2H_2O$.

Preparation of *rac*-1-(5-Bromopyridin-3-yl)ethane-1,2-diol (16):

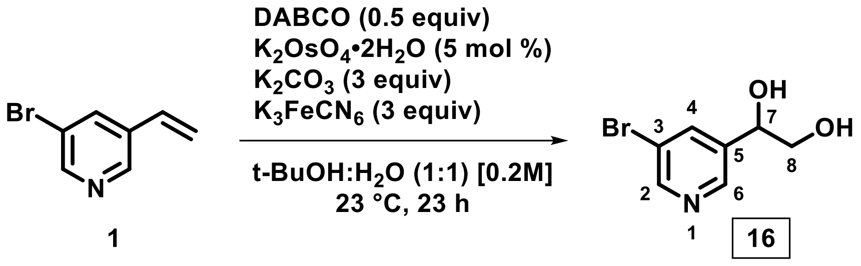

Following **General Procedure A**, 3-bromo-5-vinylpyridine (**1**) (184 mg, 1.00 mmol) was dihydroxylated after stirring at 20 °C for 23 h. Upon completion, **16** was isolated via automated silica gel flash chromatography on a pre-equilibrated silica gel column (Biotage Sfär HC Duo 10 g silica column, 9-mL fractions, 40 mL/min flow rate), eluting with EtOAc to afford **16** (150.5 mg, 69% yield) as a white solid.

***NOTE**: Compound **16** was not very soluble in $CDCl_3$, however due to overlapping of signals in other deuterated solvents the NMR data was best recorded in $CDCl_3$.*

Data for **16**:

^{1}H NMR: (600 MHz, $CDCl_3$)
8.61 (d, J = 2.2 Hz, 1H, HC(2)), 8.51 (d, J = 2.2 Hz, 1H, HC(6)), 7.92 (t, J = 2.2 Hz, 1H, HC(4)), 4.87 (dd, J = 7.9, 3.5 Hz, 1H, HC(7)), 3.84 (dd, J = 11.2, 3.5 Hz, 1H, H_2C(8a)), 3.67 (dd, J = 11.2, 7.9 Hz, 1H, H_2C(8b)).

^{13}C NMR: (151 MHz, $CDCl_3$)
150.54 (C2), 146.10 (C6), 137.95 (C5), 136.76 (C4), 121.10 (C3), 71.84 (C7), 67.70 (C8).

IR: (neat)
3309 (w), 3125 (w), 3085 (w), 3053 (w), 2923 (w), 2866 (w), 2828 (w), 1453 (w), 1413 (m), 1370 (w), 1327 (w), 1303 (w), 1225 (w), 1204 (w), 1095 (w), 1077 (m), 1056 (s), 1030 (s), 1024 (s), 926 (w), 909 (s), 884 (m), 869 (w), 744 (m), 697 (s), 668 (w), 646 (m), 596 (m), 549 (m), 519 (s).

HRMS: TOF MS ES+ m/z [M+H$^+$] calculated for $C_7H_9BrNO_2$ 217.9817; found 217.9815.

TLC: R_f = 0.11 (EtOAc, UV/$KMnO_4$)

m.p: 91.5 °C - 92.3 °C (sealed tube)

Analysis: $C_7H_9BrNO_2$ (216.97)

Calcd: C, 38.56%; H, 3.70%; N, 6.42%

Found: C, 38.61%; H, 3.72%; N, 6.30%

HPLC: t_R = 17.626 min and t_R = 21.186 min (Supelco Astec, hexanes/*i*-PrOH, 90:10, 1.0 mL/min; 220 nm; 24 °C).

Preparation of *rac*-3-(4-(*tert*-Butyl)phenyl)propane-1,2-diol (17):

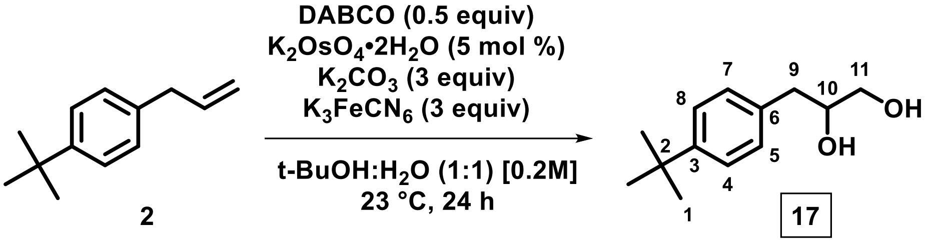

Following **General Procedure A**, 1-allyl-4-(*tert*-butyl)benzene (**2**) (174 mg, 1.00 mmol) was dihydroxylated after stirring at 20 °C for 24 h. Upon completion, **16** was isolated via automated silica gel flash chromatography on a pre-equilibrated silica gel column (Biotage Sfär HC Duo 10 g silica column, 9-mL fractions, 40 mL/min flow rate), eluting with hexanes/EtOAc (100:0) to hexanes/EtOAc (25:75) over 10 min to afford **17** (186.6 mg, 90%) as a colorless oil.

Data for **17**:

^{1}H NMR: (500 MHz, $CDCl_3$)

7.34 (d, J = 8.0 Hz, 2H, HC(4, 8)), 7.16 (d, J = 8.0 Hz, 2H, HC(5, 7)), 3.95 (dtd, J = 11.0, 7.0, 3.1 Hz, 1H, HC(10)), 3.70 (dd, J = 11.0, 3.1 Hz, 1H, HC(11)), 3.53 (dd, J = 11.0, 7.0 Hz, 1H, HC(11)), 2.75 (qd, J = 13.7, 7.0 Hz, 2H, $H_2C(9)$), 1.99 (br s, 2H, OH), 1.31 (s, 9H, $H_3C(1)$).

^{13}C NMR: (126 MHz, $CDCl_3$)

149.68 (C3), 134.64 (C6), 129.14 (C5 and C7), 125.74 (C4 and C8), 73.16 (C11), 66.28 (C10), 39.42 (C9), 34.57 (C2), 31.50 (C1).

IR: (neat)

3351 (m), 3343 (m), 3093 (w), 3056 (w), 3026 (w), 2957 (m), 2905 (m), 2867 (m), 1513 (m), 1474 (w), 1460 (w), 1447 (w), 1410 (w), 1394 (w), 1363 (m), 1269 (m), 1237 (w), 1202 (w), 1120 (w), 1074 (s), 1021 (s), 922 (w), 896 (w), 865 (w), 843 (m), 826 (m), 809 (m), 786 (w), 738 (w), 706 (w), 677 (w), 644 (m), 626 (m), 579 (s), 570 (s), 546 (m), 534 (m).

HRMS: EI+ m/z [M^+] calculated for $C_{13}H_{20}O_2$: 208.14633; found: 208.14587.

TLC: R_f = 0.04 (EtOAc/hexanes, 75:25, UV/$KMnO_4$)

Purity: 90.33% (Q-NMR, Dimethyl sulfone used as the internal standard)

RP-HPLC: t_R = 3.973 min and t_R = 5.574 min (IF3, MeCN/H_2O, 40:60, 1.0 mL/min; 220 nm; 24 °C).

Preparation of *rac*-6-(Benzyloxy)hexane-1,2-diol (18):

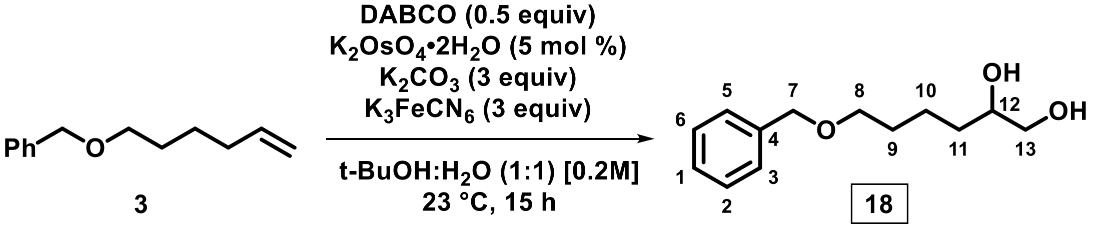

Following **General Procedure A**, ((hex-5-en-1-yloxy)methyl)benzene (**3**) (191 mg, 1.00 mmol) was dihydroxylated after stirring at 20 °C for 15 h. Upon completion, **18** was isolated via automated silica gel flash chromatography on a pre-equilibrated silica gel column (Biotage Sfär HC Duo 25 g silica column, 9-mL fractions, 80 mL/min flow rate), eluting with hexanes/EtOAc (25:75) to hexanes/EtOAc (0:100) over 10 min to afford **18** (202.7 mg, 90%) as a colorless oil.

Data for **18**:

^{1}H NMR: (600 MHz, $CDCl_3$)

7.37 – 7.32 (m, 4H, HC(2, 3, 4, 6)), 7.30 – 7.26 (m, 1H, HC(1)), 4.50 (s, 2H, H_2C(7)), 3.75 – 3.68 (m, 1H, HC(12)), 3.64 (dtd, *J* = 11.1, 2.1, 1.0 Hz, 1H, H_2C(13a)), 3.49 (td, *J* = 6.4, 1.0 Hz, 2H, H_2C(8)), 3.42 (ddd, *J* = 11.1, 7.6, 1.0 Hz,

1H, $H_2C(13b)$), 2.05 (s, 2H, OH), 1.73 – 1.60 (m, 2H, $H_2C(9)$), 1.59 – 1.50 (m, 1H, $H_2C(11a)$), 1.50 – 1.40 (m, 3H, $H_2C(10)$ and $H_2C(11b)$.

^{13}C NMR: (151 MHz, $CDCl_3$)
138.63 (C4), 128.53 (C2 and C6), 127.83 (C3 and C5), 127.72 (C1), 73.11 (C7), 72.26 (C12), 70.31 (C8), 66.93 (C13), 33.03 (C10), 29.74 (C9), 22.42 (C11).

IR: (neat)
3374 (m), 2936 (m), 2861 (m), 1496 (w), 1454 (m), 1434 (w), 1410 (w), 1363 (m), 1330 (w), 1309 (w), 1257 (w), 1232 (w), 1205 (w), 1178 (w), 1098 (s), 1075 (s), 1028 (m), 931 (w), 913 (w), 864 (w), 735 (s), 698 (s), 679 (w), 652 (w), 612 (w), 576 (w), 543 (w), 537 (w), 531 (w).

HRMS: TOF MS ES^+ m/z $[M+Na^+]$ calculated for $C_{13}H_{20}O_3Na$: 247.1310; found: 247.1312

TLC: R_f = 0.24 (EtOAc/hexanes, 50:50, UV/$KMnO_4$)

Purity: 97.43% (Q-NMR, Dimethyl sulfone used as the internal standard)

HPLC: t_R = 15.651 min and t_R = 18.234 min (Supelco Astec, hexanes/*i*-PrOH, 90:10, 1.0 mL/min; 210 nm; 24 °C).

Preparation of *rac*-1-(3,5-Dichlorophenyl)-1-(4-tolyl)ethane-1,2-diol (19):

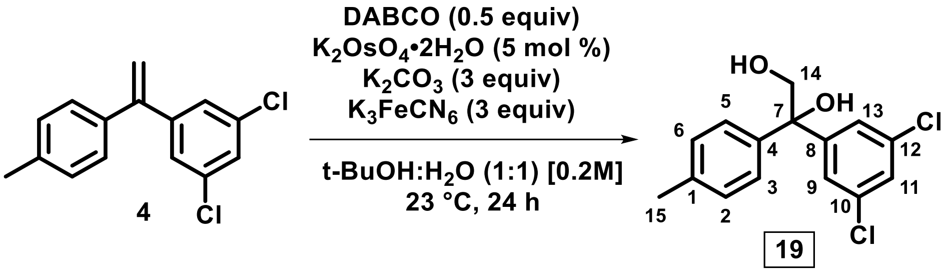

Following **General Procedure A**, 1,3-dichloro-5-(1-(4-tolyl)vinyl)benzene (**4**) (263 mg, 1.00 mmol) was dihydroxylated after stirring at 20 °C for 24 h. Upon completion, **19** was isolated via automated silica gel flash chromatography on a pre-equilibrated silica gel column (Biotage Sfär HC Duo 10 g silica column, 9-mL fractions, 40 mL/min flow rate), eluting with

hexanes/EtOAc (100:0) to hexanes/EtOAc (0:100) over 11 min to afford **19** (148.5 mg, 50%) as a white solid.

Data for **19**:

^{1}H NMR: (600 MHz, $CDCl_3$)
7.32 (d, J = 1.9 Hz, 2H, HC(9, 13)), 7.31 – 7.27 (m, 2H, HC(3, 5)), 7.25 (t, J = 1.9 Hz, 1H, HC(11)), 7.18 (d, J = 7.8 Hz, 1H, HC(2, 6)), 4.14 (d, J = 11.4 Hz, 1H, H_2C(14a)), 4.03 (d, J = 11.4 Hz, 1H, H_2C(14b)), 3.24 (s, 1H, OH), 2.34 (s, 3H, H_3C(15)), 1.97 (s, 1H, OH).

^{13}C NMR: (151 MHz, $CDCl_3$)
147.79 (C8), 139.76 (C4), 137.99 (C1), 135.11 (C10 and C12), 129.61 (C2 and C6), 127.65 (C11), 126.24 (C3 and C5), 125.22 (C9 and C13), 78.02 (C7), 69.10 (C14), 21.17 (C15).

IR: (neat)
3351 (w), 3269 (w), 3262 (w), 3088 (w), 3059 (w), 3026 (w), 2997 (w), 2975 (w), 2950 (w), 2921 (w), 2869 (w), 1611 (w), 1587 (w), 1563 (m), 1510 (w), 1467 (w), 1417 (s), 1377 (m), 1343 (w), 1316 (w), 1297 (w), 1263 (w), 1244 (w), 1206 (m), 1192 (w), 1180 (w), 1130 (w), 1109 (m), 1081 (m), 1031 (s), 997 (w), 969 (w), 953 (w), 942 (w), 912 (w), 886 (w), 853 (s), 839 (w), 818 (s), 796 (s), 776 (s), 738 (m), 722 (m), 683 (s), 647 (w), 622 (m), 589 (m), 571 (m), 537 (w).

HRMS: TOF MS ES^- m/z [$M-H^-$] calculated for $C_{15}H_{13}O_2Cl_2$: 295.0293; found: 295.0291

TLC: R_f = 0.81, (EtOAc, UV/$KMnO_4$)

m.p: 106.0 °C – 106.8 °C (sealed tube)

Analysis: $C_{15}H_{14}O_2Cl_2$ (296.04)
Calcd: C, 60.62%; H, 4.75%
Found: C, 60.69%; H, 4.85%

RP-HPLC: t_R = 20.280 min and t_R = 28.298 min (ADR-H, MeCN/H_2O, 40:60, 1.0 mL/min; 210 nm; 29.5 °C).

Preparation of *rac*-2-(4-Chlorophenyl)-3-((triethylsilyl)oxy)propane-1,2-diol (20):

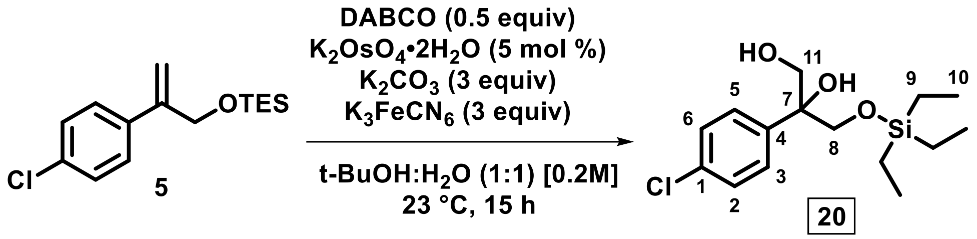

Following **General Procedure A**, ((2-(4-chlorophenyl)allyl)oxy)triethylsilane (**5**) (283 mg, 1.00 mmol) was dihydroxylated after stirring at 20 °C for 15 h. Upon completion, **20** was isolated via automated silica gel flash chromatography on a pre-equilibrated silica gel column (Biotage Sfär HC Duo 10 g silica column, 9-mL fractions, 40 mL/min flow rate), eluting with hexanes/EtOAc (100:0) to hexanes/EtOAc (80:20) over 14 min to afford **20** (286.8 mg, 91%) as a colorless oil.

Data for **20**:

^{1}H NMR: (500 MHz, $CDCl_3$)
7.39 (d, *J* = 8.5 Hz, 2H, HC(2, 6), 7.32 (d, *J* = 8.5 Hz, 2H, HC(3, 5)), 3.95 (d, *J* = 10.0 Hz, 1H, H_2C(8a)), 3.90 (d, *J* = 11.4 Hz, 1H, H_2C(11a)), 3.72 (d, *J* = 10.0 Hz, 1H, H_2C(H8b)), 3.67 (d, *J* = 11.4 Hz, 1H, H_2C(H11b)), 3.44 (s, 1H, OH), 2.46 (s, 1H, OH), 0.92 (t, *J* = 7.7 Hz, 9H, H_3C(10)), 0.58 (q, *J* = 7.7 Hz, 6H, H_2C(9)).

^{13}C NMR: (126 MHz, $CDCl_3$)
140.34 (C4), 133.47 (C1), 128.54 (C2 and C6), 126.98 (C3 and C5), 75.77 (C7), 68.80 (C8), 68.23 (C11), 6.74 (C10), 4.32 (C9).

IR: (neat)
3437 (w), 2954 (w), 2938 (w), 2912 (w), 2876 (w), 1491 (w), 1458 (w), 1412 (w), 1349 (w), 1334 (w), 1288 (w), 1273 (w), 1239 (w), 1195 (w), 1091 (s), 1031 (m), 1012 (s), 974 (w), 941 (w), 900 (w), 870 (w), 807 (s), 741 (s), 727 (s), 676 (w), 646 (w), 633 (w), 609 (w), 557 (w), 507 (m).

HRMS: TOF MS ES$^+$ m/z [M+Na$^+$] calculated for $C_{15}H_{25}O_3NaClSi$: 339.1159; found: 339.1157

TLC: R_f = 0.33, (hexanes/EtOAc, 80:20, UV/$KMnO_4$)

Purity: >99% (Q-NMR, Dimethyl sulfone used as the internal standard)

HPLC: t_R = 4.702 min and t_R = 6.106 min (Whelk, hexanes/*i*-PrOH, 90:10, 1.0 mL/min; 215 nm; 24 °C).

Preparation of *rac*-2-(2-Chlorophenyl)-3-((triethylsilyl)oxy)propane-1,2-diol (21):

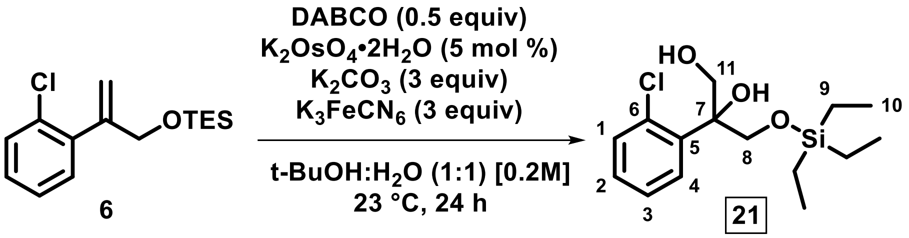

Following **General Procedure A**, ((2-(2-chlorophenyl)allyl)oxy)triethylsilane (**6**) (283 mg, 1.00 mmol) was dihydroxylated after stirring at 20 °C for 24 h. Upon completion, **21** was isolated via automated silica gel flash chromatography on a pre-equilibrated silica gel column (Biotage Sfär HC Duo 25 g silica column, 9-mL fractions, 80 mL/min flow rate), eluting with hexanes/EtOAc (100:0) to hexanes/EtOAc (80:20) over 10 min to afford **21** (283.3 mg, 89%) as a colorless oil.

Data for **21**:

^{1}H NMR: (600 MHz, $CDCl_3$)
7.87 (dd, J = 7.9, 1.7 Hz, 1H, HC(1)), 7.33 (dd, J = 7.9, 1.3 Hz, 1H, HC(4)), 7.31 – 7.27 (m, 1H, HC(3)), 7.21 (td, J = 7.9, 1.7 Hz, 1H, HC(2)), 4.33 (dd, J = 11.3, 4.2 Hz, 1H, H_2C(8a)), 4.26 (dd, J = 10.0, 1.7 Hz, 1H, H_2C(11a)), 4.13 (dd, J = 10.0, 1.7 Hz, 1H, H_2C(11b)), 3.97 (br s, 1H, OH), 3.90 (dd, J = 11.3, 4.2 Hz, 1H, H_2C(8b)), 2.47 (br s, 1H, OH), 0.88 (t, J = 8.0 Hz, 9H, H_3C(10)), 0.54 (q, J = 8.0 Hz, 6H, H_2C(9)).

^{13}C NMR: (151 MHz, $CDCl_3$)
139.08 (C5), 131.21 (C4), 130.65 (C6), 129.75 (C1), 129.10 (C2), 127.07 (C3), 76.99 (C7), 65.54 (C8), 65.52 (C11), 6.67 (C10), 4.32 (C9).

<u>IR:</u> (neat)

3471 (w), 2954 (w), 2938 (w), 2912 (w), 2876 (w), 1465 (w), 1430 (w), 1413 (w), 1381 (w), 1337 (w), 1287 (w), 1239 (w), 1185 (w), 1142 (w), 1077 (m), 1034 (m), 1004 (s), 973 (w), 898 (w), 803 (m), 743 (s), 725 (s), 686 (m), 673 (m), 630 (w), 594 (w), 566 (w), 507 (w).

<u>HRMS:</u> TOF MS ES$^+$ m/z [M+Na$^+$] calculated for $C_{15}H_{25}O_3NaClSi$: 339.1159; found: 339.1159

<u>TLC:</u> R_f = 0.52, (hexanes/EtOAc, 70:30, UV/$KMnO_4$)

<u>Purity:</u> 94.68% (Q-NMR, Dimethyl sulfone used as the internal standard)

<u>HPLC:</u> t_R = 8.969 min and t_R = 10.305 min (Whelk, hexanes/*i*-PrOH, 97:03, 1.0 mL/min; 220 nm; 24 °C).

<u>Preparation of *rac*-3-((7-Methoxynaphthalen-1-yl)oxy)-2-methylpropane-1,2-diol (22):</u>

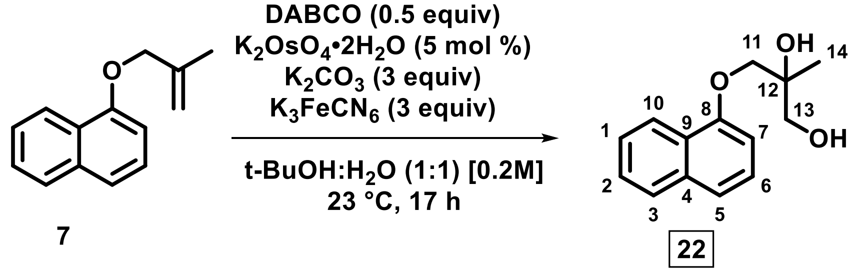

Following **General Procedure A**, 7-methoxy-1-((2-methylallyl)oxy)naphthalene (**7**) (200 mg, 1.00 mmol) was dihydroxylated after stirring at 20 °C for 17 h. Upon completion, **22** was isolated via automated silica gel flash chromatography on a pre-equilibrated silica gel column (Biotage Sfär HC Duo 25 g silica column, 9-mL fractions, 80 mL/min flow rate), eluting with hexanes/EtOAc (100:0) to hexanes/EtOAc (0:100) over 10 min to afford **22** (182.1 mg, 78%) as a white solid.

<u>Data for **22**:</u>

^{1}H NMR: (500 MHz, $CDCl_3$)

8.20 (dd, J = 7.2, 2.4 Hz, 1H, HC(10)), 7.82 (dd, J = 7.2, 2.4 Hz, 1H, HC(3)), 7.54

– 7.47 (m, 2H, HC(1, 2)), 7.46 (d, *J* = 7.9 Hz, 1H, HC(5)), 7.38 (t, *J* = 7.9 Hz, 1H, HC(6)), 6.85 (d, *J* = 7.9 Hz, 1H, H7), 4.11 (dd, *J* = 15.5, 9.0 Hz, 2H, $H_2C(11)$), 3.86 (d, *J* = 11.1 Hz, 1H, $H_2C(13a)$), 3.70 (d, *J* = 11.1 Hz, 1H, $H_2C(13b)$), 2.71 (s, 1H, OH), 2.13 (s, 1H, OH), 1.42 (s, 3H, $H_3C(14)$).

^{13}C NMR: (126 MHz, $CDCl_3$)
154.21 (C8), 134.65 (C9), 127.82 (C3), 126.66 (C2), 125.98 (C1), 125.58 (C6), 125.55 (C4), 121.64 (C10), 121.05 (C5), 105.21 (C7), 72.65 (C12), 72.47 (C11), 67.82 (C13), 21.91 (C14).

IR: (neat)
3290 (w), 3054 (w), 2978 (w), 2932 (w), 2875 (w), 1595 (w), 1579 (m), 1509 (w), 1470 (w), 1459 (w), 1438 (w), 1427 (w), 1402 (m), 1395 (m), 1376 (w), 1363 (w), 1316 (w), 1268 (m), 1242 (m), 1215 (w), 1179 (w), 1140 (m), 1103 (s), 1068 (m), 1053 (m), 1021 (m), 1006 (m), 980 (w), 965 (w), 918 (w), 874 (w), 791 (s), 770 (s), 730 (m), 697 (w), 690 (w), 683 (w), 673 (w), 667 (w), 662 (w), 634 (w), 615 (m), 573 (m), 550 (w), 521 (w), 510 (w).

HRMS: TOF MS ES^+ m/z $[M+Na^+]$ calculated for $C_{14}H_{16}O_3Na$: 255.0997; found: 255.1004

TLC: R_f = 0.75, (EtOAc, UV/$KMnO_4$)

m.p: 77.9 °C - 81.0 °C (sealed tube)

Purity: >99% (Q-NMR, Dimethyl sulfone as the internal standard)

HPLC: t_R = 11.494 min and t_R = 14.877 min (Supelco Astec, hexanes/*i*-PrOH, 80:20, 1.0 mL/min; 215 nm; 24 °C).

Preparation of *rac*-Methyl 2,3-Dihydroxy-4-phenylbutanoate (23):

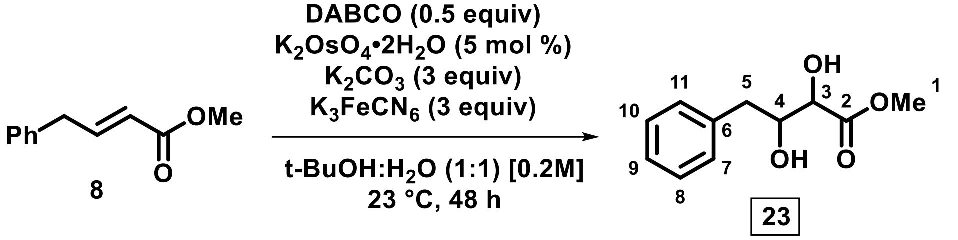

Following **General Procedure A**, methyl (*E*)-4-phenylbut-2-enoate (**8**) (176 mg, 1.00 mmol) was dihydroxylated after stirring at 20 °C for 48 h. Upon completion, **23** was isolated via automated silica gel flash chromatography on a pre-equilibrated silica gel column (Biotage Sfär HC Duo 25 g silica column, 9-mL fractions, 80 mL/min flow rate), eluting with hexanes/EtOAc (100:0) to hexanes/EtOAc (0:100) over 10 min to afford **23** (99.1 mg, 47%) as a white solid.

Data for **23**:

^{1}H NMR: (600 MHz, $CDCl_3$)
7.34 (m, 2H, HC(8, 10)), 7.31 – 7.23 (m, 3H, HC(7, 9, 11)), 4.18 (qd, *J* = 8.0, 1.8 Hz, 1H, HC(4)), 4.10 (dd, *J* = 5.6, 1.8 Hz, 1H, HC(3)), 3.81 (s, 3H, $H_3C(1)$), 3.15 (s, 1H, OH), 2.97 (ddd, *J* = 20.2, 13.2, 8.0 Hz, 2H, $H_2C(5)$), 2.13 (s, 1H, OH).

^{13}C NMR: (151 MHz, $CDCl_3$)
174.10 (C2), 137.52 (C6), 129.54 (C7 and C11), 128.82 (C8 and C10), 126.86 (C9), 73.59 (C4), 71.97 (C3), 52.97 (C1), 40.24 (C5).

IR: (neat)
3450 (w), 3028 (w), 2954 (w), 2928 (w), 1735 (s), 1604 (w), 1495 (w), 1454 (m), 1439 (m), 1397 (w), 1365 (w), 1354 (w), 1262 (s), 1216 (s), 1172 (m), 1156 (w), 1112 (s), 1077 (m), 1049 (s), 1033 (m), 1004 (w), 984 (m), 950 (w), 925 (w), 851 (w), 831 (w), 787 (w), 746 (m), 701 (s), 672 (w), 623 (w), 567 (w), 515 (m).

HRMS: TOF MS ES^+ m/z [$M+Na^+$] calculated for $C_{11}H_{14}O_4Na$: 233.0790; found: 233.0791

TLC: R_f = 0.50, (EtOAc, UV/$KMnO_4$)

m.p: 80.0 °C – 80.9 °C (sealed tube)

Purity: 98.94% (Q-NMR, Dimethyl sulfone used as the internal standard)

HPLC: t_R = 19.676 min and t_R = 25.919 min (ADH, hexanes/*i*-PrOH, 90:10, 1.0 mL/min; 215 nm; 24 °C).

Preparation of *rac*-1-Tosylpiperidine-3,4-diol (24):

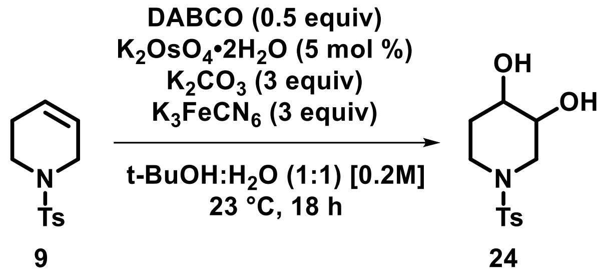

Following **General Procedure B**, 1-tosyl-1,2,3,6-tetrahydropyridine (**9**) (239 mg, 1.00 mmol) was dihydroxylated after stirring at 20 °C for 18 h. Upon completion, **24** was isolated via trituration from hexanes/CH_2Cl_2 (80:20, 50 mL) and the resulting white precipitate filtered to afford **24** (243.8 mg, 89%) as a white solid with spectral data identical to that previously reported.[13]

Data for **24**:

HRMS: TOF MS ES$^+$ m/z [M+Na$^+$] calculated for $C_{11}H_{15}N_5NaS$: 272.0946; found: 272.0956

TLC: R_f = 0.26, (EtOAc, UV/$KMnO_4$)

HPLC: t_R = 14.236 min and t_R = 18.435 min (OJH, hexanes/*i*-PrOH, 85:15, 2.0 mL/min; 240 nm; 24 °C).

Preparation of *rac*-7-Methoxy-1,2,3,4-tetrahydronaphthalene-1,2-diol (25):

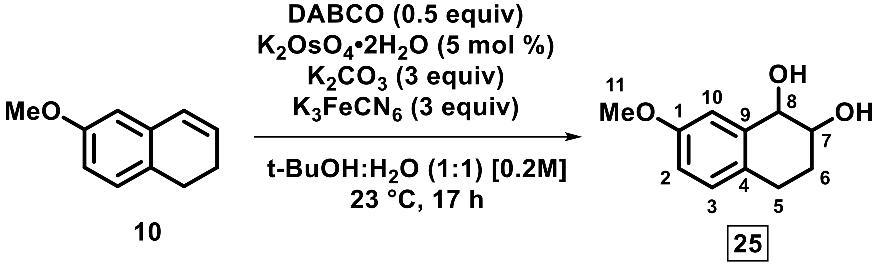

Following **General Procedure A**, 6-methoxy-1,2-dihydronaphthalene (**10**) (160 mg, 1.00 mmol) was dihydroxylated after stirring at 20 °C for 17 h. Upon completion, **25** was isolated via automated silica gel flash chromatography on a pre-equilibrated silica gel column (Biotage Sfär HC Duo 25 g silica column, 9-mL fractions, 80 mL/min flow rate), eluting with EtOAc over 10 min to afford **25** (143.3 mg, 74%) as a white solid.

Data for **25**:

^{1}H NMR: (600 MHz, $CDCl_3$)
7.04 (d, J = 8.4 Hz, 1H, HC(3)), 6.98 (d, J = 2.7 Hz, 1H, HC(10)), 6.81 (dd, J = 8.4, 2.7 Hz, 1H, HC(2)), 4.65 (dd, J = 6.2, 3.8 Hz, 1H, HC(8)), 4.02 (dq, J = 9.8, 6.2, 3.8 Hz, 1H, HC(7)), 3.80 (s, 3H, $H_3C(11)$), 2.89 (dt, J = 16.7, 5.6 Hz, 1H, $H_2C(5a)$), 2.72 (ddd, J = 16.7, 9.3, 5.6 Hz, 1H, $H_2C(5b)$), 2.42 (d, J = 6.5 Hz, 1H, OH), 2.36 (d, J = 6.5 Hz, 1H, OH), 2.02 (dtd, J = 13.0, 9.3, 5.7 Hz, 1H, $H_2C(6a)$), 1.94 – 1.87 (m, 1H, $H_2C(6b)$).

^{13}C NMR: (151 MHz, $CDCl_3$)
158.20 (C1), 137.41 (C4), 129.63 (C3), 128.12 (C9), 115.06 (C2), 113.74 (C10), 70.17 (C7), 69.55 (C8), 55.38 (C11), 26.57 (C6), 25.88 (C5).

IR: (neat)
3418 (w), 3389 (w), 3373 (w), 3363 (w), 3350 (w), 3332 (w), 3325 (w), 2937 (w), 1613 (w), 1502 (s), 1460 (w), 1433 (w), 1318 (w), 1261 (m), 1248 (m), 1205 (w), 1152 (w), 1116 (w), 1067 (w), 1056 (w), 1035 (s), 964 (w), 836 (w), 785 (w).

HRMS: TOF MS ES$^+$ m/z [M+Na$^+$] calculated for $C_{11}H_{14}O_3Na$: 217.0841; found: 217.0842

TLC: R_f = 0.33, (EtOAc, UV/$KMnO_4$)

m.p: 125.5°C – 126.0 °C (sealed tube)

Purity: >99% (Q-NMR, Dimethyl sulfone used as the internal standard)

HPLC: t_R = 17.881 min and t_R = 21.250 min (OJH, hexanes/*i*-PrOH, 90:10, 1.0 mL/min; 215 nm; 24 °C).

Preparation of *rac*-2,3-Dihydroxybutyl 3,5-bis(trifluoromethyl)benzoate (26):

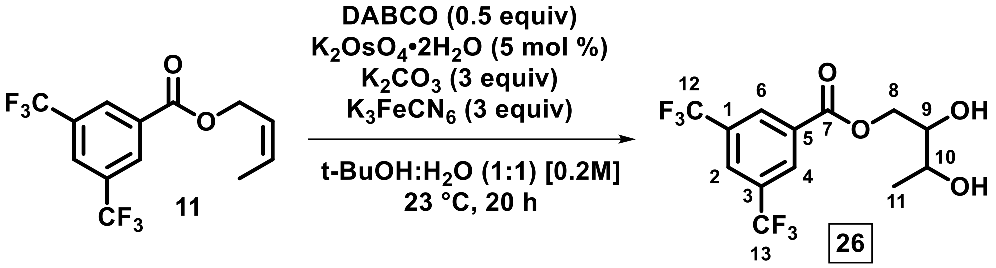

Following **General Procedure A**, (*Z*)-but-2-en-1-yl 3,5-bis(trifluoromethyl)benzoate (**11**) (312 mg, 1.00 mmol) was dihydroxylated after stirring at 20 °C for 20 h. Upon completion, **26** was isolated via automated silica gel flash chromatography on a pre-equilibrated silica gel column (Biotage Sfär HC Duo 10 g silica column, 9-mL fractions, 40 mL/min flow rate), eluting with hexanes/Et_2O (100:0) to hexanes/Et_2O (25:75) over 10 min to afford **26** (80 mg, 23%) as a white solid.

***NOTE**: **26** was observed to undergo acyl transfer leading to by-products upon extended periods of storage, even at -20 °C.*

Data for **26**:

^{1}H NMR: (600 MHz, $CDCl_3$)
8.50 (s, 2H, HC(4, 6)), 8.09 (s, 1H, HC(2)), 4.55 (qd, J = 11.8, 4.9 Hz, 2H, $H_2C(8)$), 4.01 – 3.92 (m, 2H, **overlapping peaks**, HC(9/10)), 2.45 (s, 1H, OH), 2.03 (s, 1H, OH), 1.31 (d, J = 6.1 Hz, 3H, $H_3C(11)$).

^{13}C NMR: (151 MHz, $CDCl_3$)

164.45 (C7), 132.33 (q, *J* = 34.1 Hz, C1 and C3), 131.96 (C5), 129.86 (q, *J* = 3.9 Hz, C4 and C6), 126.66 (hept, *J* = 3.9 Hz, C2), 122.81 (q, *J* = 272.9 Hz, C12 and C13), 73.57 (C9), 68.40 (C10), 67.01 (C8), 18.34 (C11).

IR: (neat)
3402 (w), 3394 (w), 3386 (w), 2979 (w), 2973 (w), 2923 (w), 1728 (m), 1624 (w), 1457 (w), 1375 (w), 1324 (w), 1276 (s), 1246 (s), 1176 (m), 1128 (s), 1072 (m), 1010 (w), 975 (m), 913 (m), 893 (w), 865 (w), 845 (m), 770 (m), 735 (w), 700 (m), 681 (s), 652 (w), 617 (w), 580 (w), 538 (w), 529 (w).

HRMS: TOF MS ES^- m/z [$M-H^-$] calculated for $C_{13}H_{11}O_4F_6$: 345.0562; found: 345.0570

TLC: R_f = 0.53, (EtOAc, UV/$KMnO_4$)

m.p: 76.5°C - 77.3 °C (sealed tube)

Purity: >99% (Q-NMR, Dimethyl sulfone used as the internal standard)

HPLC: t_R = 22.464 min and t_R = 26.647 min (Supelco Astec, hexanes/*i*-PrOH, 95:5, 1.0 mL/min; 220 nm; 24 °C).

Preparation of *rac*-Ethyl 2,3-Dihydroxy-3-phenylbutanoate (27):

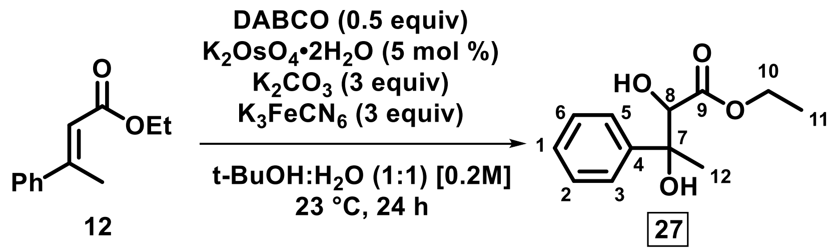

Following **General Procedure A**, ethyl (*E*)-3-phenylbut-2-enoate (**12**) (190 mg, 1.00 mmol) was dihydroxylated after stirring at 20 °C for 24 h. Upon completion, **27** was isolated via automated silica gel flash chromatography on a pre-equilibrated silica gel column (Biotage Sfär HC Duo 10 g silica column, 9-mL fractions, 40 mL/min flow rate), eluting with EtOAc over 5 min to afford **27** (154.6 mg, 69 %) as a colorless oil.

Data for **27**:

^{1}H NMR: (600 MHz, $CDCl_3$)
7.49 – 7.46 (m, 2H, HC(3, 5)), 7.37 – 7.33 (m, 2H, HC(2, 6)), 7.29 – 7.25 (m, 1H, HC(1)), 4.35 (d, J = 6.4 Hz, 1H, HC(8)), 4.22 – 4.11 (m, 2H, $H_2C(10)$), 3.27 (s, 1H, OH-C(8)), 3.11 (d, J = 6.4 Hz, 1H, OH-C(7)), 1.62 (s, 3H, $H_3C(12)$), 1.17 (t, J = 7.1 Hz, 3H, $H_3C(11)$).

^{13}C NMR: (151 MHz, $CDCl_3$)
172.78 (C9), 143.91 (C4), 128.29 (C3 and C5), 127.52 (C1), 125.34 (C2 and C6), 77.78 (C8), 75.80 (C7), 62.20 (C10), 25.95 (C12), 14.13 (C11).

IR: (neat)
3473 (w), 3464 (w), 2982 (w), 2938 (w), 1726 (s), 1495 (w), 1464 (w), 1447 (m), 1369 (m), 1343 (w), 1265 (m), 1199 (s), 1158 (w), 1144 (w), 1095 (s), 1069 (m), 1055 (s), 1026 (s), 937 (w), 913 (w), 863 (w), 833 (w), 785 (w), 764 (m), 761 (m), 730 (w), 700 (s), 623 (w), 569 (m), 546 (m), 520 (w), 513 (w), 501 (m).

HRMS: TOF MS ES^+ m/z [$M+Na^+$] calculated for $C_{12}H_{16}O_4Na$: 247.0946; found: 247.0945

TLC: R_f = 0.65, (EtOAc, UV/$KMnO_4$)

Purity: 99.42% (Q-NMR, Dimethyl sulfone used as the internal standard)

Analysis: $C_{12}H_{16}O_4$ (224.10)
Calcd: C, 64.27 %; H, 7.19 %
Found: C, 64.12 %; H, 7.00 %

HPLC: t_R = 11.467 min and t_R = 13.391 min (Supelco Astec, hexanes/*i*-PrOH, 90:10, 1.0 mL/min; 220 nm; 24 °C).

Preparation of *rac*-5-(Benzyloxy)-2-methylpentane-2,3-diol (28):

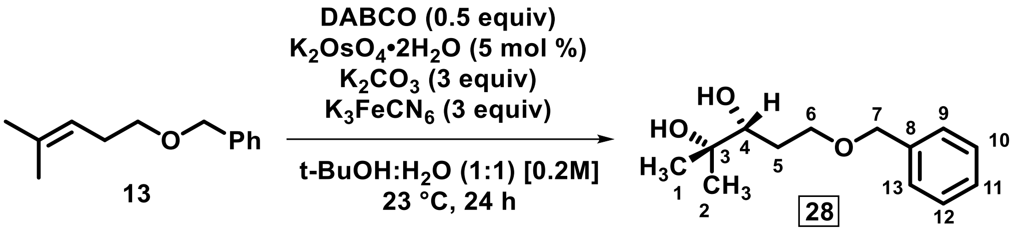

Following **General Procedure A**, (((4-methylpent-3-en-1-yl)oxy)methyl)benzene (**13**) (190 mg, 1.00 mmol) was dihydroxylated after stirring at 20 °C for 24 h. Upon completion, **28** was isolated via automated silica gel flash chromatography on a pre-equilibrated silica gel column (Biotage Sfär HC Duo 25 g silica column, 9-mL fractions, 80 mL/min flow rate), eluting with hexanes/EtOAc (100:0) to hexanes/EtOAc (0:100) over 10 min to afford **28** (167.3 mg, 75%) as a colorless oil.

Data for **28**:

^{1}H NMR: (600 MHz, $CDCl_3$)
7.40 – 7.28 (m, 5H, HC(9, 10, 11, 12, 13)), 4.54 (AB q, $\Delta\upsilon$ = 5.7 Hz, *J* = 11.9 Hz, 2H, $H_2C(7)$), 3.76 (dt, *J* = 9.5, 4.9 Hz, 1H, $H_2C(6a)$), 3.72 – 3.67 (m, 1H, $H_2C(6b)$), 3.61 (dd, *J* = 4.7 Hz 1H, HC(4)), 3.36 (s, 1H, OH), 2.37 (s, 1H, OH), 1.83 – 1.72 (m, 2H, $H_2C(5)$), 1.21 (s, 3H, $H_3C(1)$), 1.16 (s, 3H, $H_3C(2)$).

^{13}C NMR: (151 MHz, $CDCl_3$)
137.84 (C8), 128.66 (C10 and C12), 128.00 (C11), 127.88 (C9 and C13), 78.08 (C4), 73.59 (C7), 72.52 (C3), 69.66 (C6), 31.11 (C5), 26.34 (C1), 24.14 (C2).

IR: (neat)
3415 (w), 2972 (w), 2931 (w), 2865 (w), 1454 (w), 1365 (m), 1310 (w), 1289 (w), 1207 (w), 1165 (w), 1077 (s), 1028 (w), 994 (w), 966 (w), 950 (w), 737 (m), 698 (m), 616 (w), 609 (w), 581 (w), 569 (w), 565 (w), 561 (w), 553 (w), 545 (w), 542 (w), 537 (w), 534 (w), 525 (w), 521 (w).

HRMS: TOF MS ES^+ m/z $[M+Na^+]$ calculated for $C_{13}H_{20}O_3Na$: 247.1310; found: 247.1308

TLC: R_f = 0.22 (hexanes/EtOAc, 50:50, UV/$KMnO_4$)

Purity: 97.27% (Q-NMR, Dimethyl sulfone used as the internal standard)

HPLC: t_R = 37.577 min and t_R = 42.890 min (OJH, hexanes/*i*-PrOH, 99:01, 2.0 mL/min; 205 nm; 24 °C).

Preparation of *rac*-4-Hydroxy-2-methyl-6-phenylhexan-3-one (29):

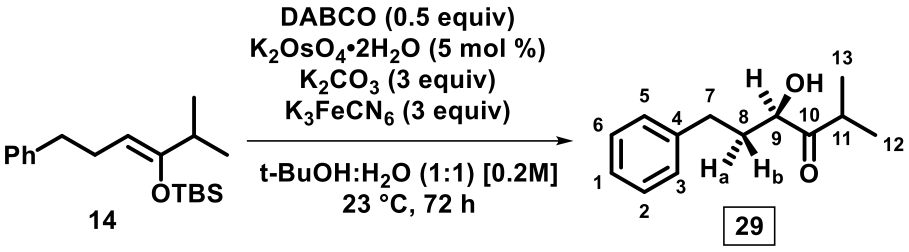

Following **General Procedure A**, (*Z*)-*tert*-butyldimethyl((2-methyl-6-phenylhex-3-en-3-yl)oxy)silane (**14**) (305 mg, 1.00 mmol) was dihydroxylated after stirring at 20 °C for 72 h. Upon completion, **29** was isolated via automated silica gel flash chromatography on a pre-equilibrated silica gel column (Biotage Sfär HC Duo 25 g silica column, 9-mL fractions, 80 mL/min flow rate), eluting with hexanes/EtOAc (90:10) over 5 min to afford **29** (156.5 mg, 76%) as a colorless oil.

Data for **29**:

^{1}H NMR: (600 MHz, $CDCl_3$)
7.33 – 7.27 (m, 2H, HC(3, 5)), 7.24 – 7.18 (m, 3H, HC(1, 2, 6)), 4.31 (dt, *J* = 7.2, 3.3 Hz, 1H, HC(9)), 3.56 (d, *J* = 5.0 Hz, 1H, OH), 2.86 – 2.69 (m, 3H, **overlapping peaks**, H_2C(7a, 7b) and HC(11)), 2.14 (dddd, *J* = 13.5, 10.2, 7.2, 3.3 Hz, 1H, H_2C(8a)), 1.78 (dtd, *J* = 13.5, 9.0, 5.0 Hz, 1H, H_2C(8b)), 1.11 (d, *J* = 2.9 Hz, 3H, H_3C(12)), 1.09 (d, *J* = 2.3 Hz, 3H, H_3C(13)).

^{13}C NMR: (151 MHz, $CDCl_3$)
216.18 (C10), 141.29 (C4), 128.70 (C2 and C6), 128.66 (C3 and C5), 126.28 (C1), 74.34 (C9), 36.08 (C11), 35.78 (C8), 31.55 (C7), 19.65 (C12), 17.69 (C13).

IR: (neat)
3472 (w), 2972 (w), 2933 (w), 2874 (w), 1708 (s), 1497 (w), 1467 (w), 1455 (m), 1384 (w), 1365 (w), 1347 (w), 1261 (w), 1227 (w), 1178 (w), 1119 (w), 1100 (w),

1072 (w), 1058 (w), 1020 (s), 928 (w), 751 (w), 722 (w), 700 (s), 505 (w).

HRMS: EI$^+$ m/z [M$^+$] calculated for $C_{13}H_{18}O_2$: 206.13068; found: 206.13083.

TLC: R_f = 0.23 (hexanes/EtOAc, 90:10, UV/$KMnO_4$)

Purity: 97.29% (Q-NMR, Dimethyl sulfone as the internal standard)

HPLC: t_R = 10.097 min and t_R = 10.938 min (OJH, hexanes/*i*-PrOH, 95:5, 1.0 mL/min; 220 nm; 24 °C).

Preparation of *rac*-4-(4-Chlorophenyl)-3-methyl-1-tosylpiperidine-3,4-diol (30):

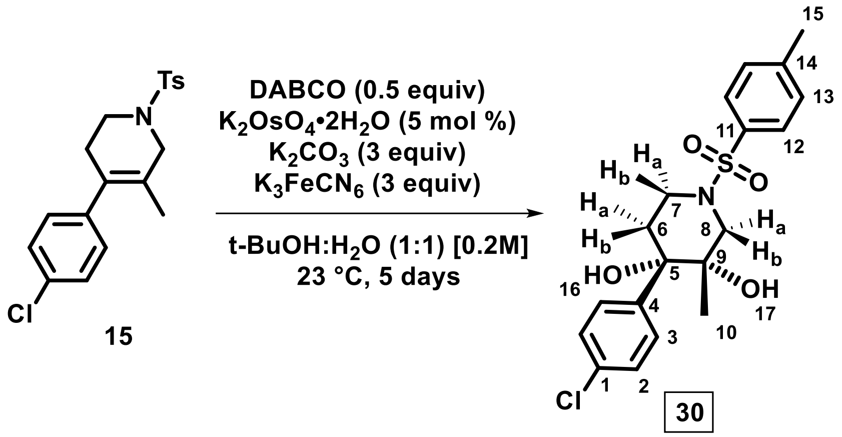

Following **General Procedure B**, 4-(4-chlorophenyl)-5-methyl-1-tosyl-1,2,3,6-tetrahydropyridine (**15**) (362 mg, 1.00 mmol) was dihydroxylated after stirring at 20 °C for 5 days. Upon completion, **30** was isolated via trituration with hexanes/CH_2Cl_2 (1:5, 30 mL) resulting in a white precipitate that was filtered and dried under vacuum (0.5 mmmHg, 20 °C) to afford **30** (330 mg, 83%, white solid)

Data for **30**:

^{1}H NMR: (600 MHz, $CDCl_3$)

7.67 (d, J = 8.2 Hz, 2H, HC(12)), 7.38 – 7.31 (m, 6H, **overlapping peaks**, HC(2/3/13), 3.73 (ddt, J = 11.2, 4.6, 2.1 Hz, 1H, H_2C(8a)), 3.47 (dd, J = 10.9, 2.1 Hz, 1H, H_2C(7a)), 2.77 (td, J = 12.3, 2.5 Hz, 1H, H_2C(8)), 2.67 (d, J = 10.9 Hz, 1H, H_2C(7b)), 2.63 (d, J = 1.2 Hz, 1H, OH-C(16)), 2.59 (dddd, J = 14.0, 12.3, 5.0, 1.2

Hz, 1H, $H_2C(6a)$), 2.45 (s, 3H, $H_3C(15)$), 1.94 (s, 1H, OH-C(17)), 1.82 (dt, J = 14.0, 2.5 Hz, 1H, $H_2C(6b)$), 1.20 (s, 3H, $H_3C(10)$).

^{13}C NMR: (151 MHz, $CDCl_3$)
143.88 (C14), 140.79 (C4), 134.02 (C1), 133.35 (C11), 129.93 (C13), 128.29 (C2 and C3), 127.78 (C12), 75.36 (C5), 73.22 (C9), 53.26 (C7), 42.05 (C8), 33.59 (C6), 23.05 (C10), 21.72 (C15).

IR: (neat)
3483 (w), 1706 (w), 1597 (w), 1493 (w), 1465 (w), 1398 (w), 1347 (m), 1330 (m), 1306 (w), 1226 (w), 1185 (w), 1161 (s), 1121 (w), 1091 (s), 1040 (w), 1017 (w), 997 (w), 975 (w), 958 (w), 925 (w), 848 (w), 813 (m), 756 (w), 729 (w), 662 (m), 640 (w), 598 (w), 574 (m), 551 (m), 520 (w), 502 (w).

HRMS: TOF MS ES^+ m/z [M+H^+] calculated for $C_{19}H_{23}NO_4SCl$: 396.1036; found: 396.1031

TLC: R_f = 0.18 (hexanes/EtOAc, 70:30, UV/$KMnO_4$)

m.p: 222.1 °C – 223.1 °C (sealed tube)

Purity: >99% (Q-NMR, Dimethyl sulfone used as the internal standard)

HPLC: t_R = 13.463 min and t_R = 15.526 min (Supelco Astec, hexanes/*i*-PrOH, 85:15, 1.5 mL/min; 230 nm; 24 °C).

Sharpless Asymmetric Dihydroxylations (SAD)

All asymmetric dihydroxylations were performed according to the Sharpless protocol on a minimum of 0.10 mmol scale or higher. The relevant solvent quantities, AD-mix β and additive amounts are detailed in the individual procedures for each alkene. The general procedure outlined below describes the general protocol employed.

General Procedure for SAD:

A 20-mL scintillation vial equipped with a cylindrical stir bar (2 cm x 1 cm, length x diameter) at rt (20 °C) and under ambient atmosphere was charged with AD-mix β (154 mg, 0.10 mmol) and methanesulfonamide (1.00 equiv)*. Then, *t*-BuOH and H_2O were added and the vial capped with a green Teflon-lined septum cap. The mixture was stirred vigorously at 700 rpm via a magnetic stir plate until all the solids were dissolved to afford a light orange solution. Then, the vial was placed into a 0 °C bath equipped with a cryocool and stirred for 10 min. To a separate 2-dram vial was charged the alkene and *t*-BuOH. The solution of alkene was then added to the scintillation vial, the 2-dram vial rinsed with a further amount of *t*-BuOH and added to the scintillation vial and capped with a green Teflon-lined septum cap. The reactions were stirred and maintained at 0 °C until completion and monitored via TLC.

If the alkene was a solid, a solution of the alkene was not prepared but rather directly added to the 20-mL scintillation vial in one portion. In these cases, the required amount of *t*-BuOH was added to the scintillation vial to prepare the homogeneous solution containing AD-mix β and methanesulfonamide.

*Methanesulfonamide was added in all cases except for monosubstituted alkenes[14]

Preparation of (*R*)-1-(5-Bromopyridin-3-yl)ethane-1,2-diol (16'):

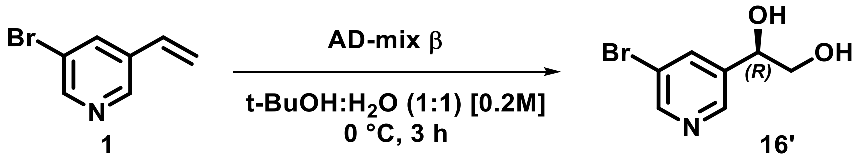

Following **General Procedure for SAD**, AD-mix β (230 mg, 0.29 mmol, 1.8 equiv), *t*-BuOH (0.37 mL) and H_2O (0.82 mL) were added to the scintillation vial, stirred at 20 °C until homogeneous and then cooled to 0 °C. In a separate 2-dram vial, a *t*-BuOH solution (0.15 mL) of **1** (30 mg, 0.16 mmol) was prepared then added in one portion to the reaction vial. The 2-dram vial was rinsed with *t*-BuOH (0.15 mL) and then added in one portion to the reaction vial. The reaction was allowed to stir for 3 h and monitored for completion via TLC. Upon completion, sodium sulfite (240 mg) was added as a solid at 0 °C, the mixture stirred for 1 h and then transferred to a 125-mL, separatory funnel with the assistance of EtOAc (5 x 5 mL rinses). Brine (25 mL) was added and the phases were shaken, separated, and the organic layer collected. The aqueous layer was further extracted with EtOAc (4 x 10 mL), the organic phases collected, combined, dried over Na_2SO_4 (5 g), filtered, and concentrated under reduced pressure (7.5 mmHg, 34 °C) to give a thin film of solid **16'** (18 mg, 51% yield) which was sufficiently pure exhibiting spectral data identical to the racemate (**16**) and directly analyzed by CSP-HPLC.

Data for **16'**:

HPLC: t_R = 16.552 min and t_R = 20.877 min (Supelco Astec, hexanes/*i*-PrOH, 90:10, 1.0 mL/min; 220 nm; 24 °C).

e.r and % ee: 98:2, 96% ee

Opt. Rot.: $[\alpha]_D^{24}$ -19.4 (*c* = 0.92 in DMSO)

Preparation of (*S*)-3-(4-(*tert*-Butyl)phenyl)propane-1,2-diol (17'):

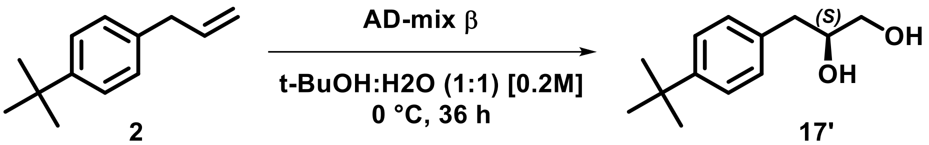

2 17'

Following **General Procedure B**, AD-mix β (240 mg, 0.31 mmol, 1.80 equiv), *t*-BuOH (0.54 mL) and H_2O (0.86 mL) were added to the scintillation vial, stirred at 20 °C until homogeneous and then cooled to 0 °C. In a separate 2-dram vial, a *t*-BuOH solution (0.16 mL) of **2** (30 mg, 0.17 mmol) was prepared then added in one portion to the reaction vial. The 2-dram vial was rinsed with *t*-BuOH (0.16 mL) and then added in one portion to the reaction vial. The reaction was allowed to stir for 36 h and monitored for completion via TLC. Upon completion, sodium sulfite (240 mg) was added as a solid at 0 °C, the mixture stirred for 1 h and then transferred to a 125-mL, separatory funnel with the assistance of CH_2Cl_2 (5 x 5 mL rinses). Brine (25 mL) was added, and the phases were shaken, separated, and the organic layer collected. The aqueous layer was further extracted with CH_2Cl_2 (5 x 10 mL), the organic phases collected, combined, dried over Na_2SO_4 (5 g), filtered, and concentrated under reduced pressure (7.5 mmHg, 34 °C) to give **17'** as an oil (27 mg, 75% yield) which was sufficiently pure exhibiting spectral data identical to the racemate (**17**) and directly analyzed by CSP-HPLC.

Data for **17'**:

HPLC: t_R = 3.653 min and t_R = 5.321 min (IF3, MeCN/H_2O, 40:60, 1.0 mL/min; 220 nm; 24 °C).

e.r and % ee: 78:22, 56% ee

Opt. Rot.: $[\alpha]_D^{24}$ +0.59 (c = 1.21 in $CHCl_3$)

Preparation of (*R*)-6-(Benzyloxy)hexane-1,2-diol (18'):

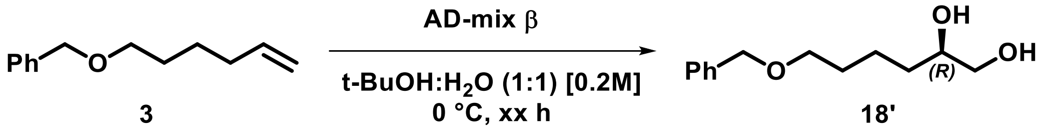

3 18'

Following **General Procedure for SAD**, AD-mix β (736 mg, 0.94 mmol, 1.80 equiv), *t*-BuOH (2.03 mL) and H_2O (2.63 mL) were added to the scintillation vial, stirred at 20 °C until homogeneous and then cooled to 0 °C. In a separate 2-dram vial, a *t*-BuOH solution (0.30 mL) of **3** (100 mg, 0.526 mmol) was prepared then added in one portion to the reaction vial. The 2-dram vial was rinsed with *t*-BuOH (0.30 mL) and then added in one portion to the reaction vial. The reaction was allowed to stir for 24 h and monitored for completion via TLC. Upon completion, sodium sulfite (762 mg) was added as a solid at 0 °C, the mixture stirred for 1 h and then transferred to a 125-mL, separatory funnel with the assistance of CH_2Cl_2 (5 x 5 mL rinses). Brine (25 mL) was added and the phases were shaken, separated, and the organic layer collected. The aqueous layer was further extracted with CH_2Cl_2 (5 x 10 mL), the organic phases collected, combined, dried over Na_2SO_4 (5 g), filtered, and concentrated under reduced pressure (7.5 mmHg, 34 °C) to give an oil.

The oil was dissolved in CH_2Cl_2 (10 mL), loaded onto Celite (1 g) and purified via automated silica gel flash chromatography on a pre-equilibrated silica gel column (Biotage Sfär HC Duo 10 g silica column, 9-mL fractions, 40 mL/min flow rate), eluting with hexanes/EtOAc (100:0) to hexanes/EtOAc (0:100) over 7 min to afford **18'** (78.9 mg, 67% yield).

Data for **18'**:

HPLC: t_R = 15.321 min and t_R = 18.036 min (Supelco Astec, hexanes/*i*-PrOH, 90:10, 1.0 mL/min; 215 nm; 24 °C).

e.r and % ee: 88:12, 76% ee

Opt. Rot.: $[\alpha]_D^{24}$ +0.14, $[\alpha]_{365}^{24}$ +1.24 (c = 4.64 in $CHCl_3$)

Preparation of (*R*)-1-(3,5-Dichlorophenyl)-1-(p-tolyl)ethane-1,2-diol (19'):

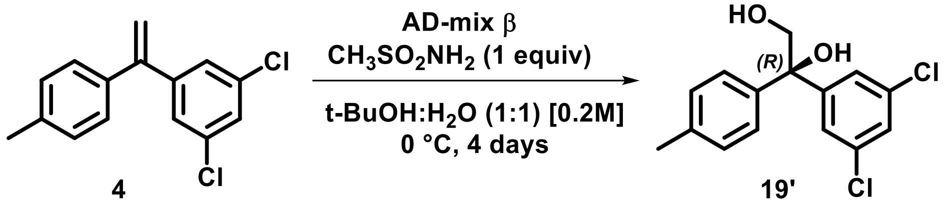

Following **General Procedure for SAD**, AD-mix β (800 mg, 1.03 mmol, 1.80 equiv), methanesulfonamide (54.2 mg, 0.57 mmol, 1.00 equiv), *t*-BuOH (2.25 mL) and H_2O (2.85 mL) were added to the scintillation vial, stirred at 20 °C until homogeneous and then cooled to 0 °C. In a separate 2-dram vial, a *t*-BuOH solution (0.30 mL) of **4** (150 mg, 0.57 mmol) was prepared then added in one portion to the reaction vial. The 2-dram vial was rinsed with *t*-BuOH (0.30 mL) and then added in one portion to the reaction vial. The reaction was allowed to stir for 4 days and monitored for completion via TLC. Upon completion, sodium sulfite (826 mg) was added as a solid at 0 °C, the mixture stirred for 1 h and then transferred to a 125-mL, separatory funnel with the assistance of CH_2Cl_2 (5 x 5 mL rinses). Brine (25 mL) was added and the phases were shaken, separated, and the organic layer collected. The aqueous layer was further extracted with CH_2Cl_2 (5 x 10 mL), the organic phases collected, combined, dried over Na_2SO_4 (5 g), filtered, and concentrated under reduced pressure (7.5 mmHg, 34 °C) to give an oil.

The oil was dissolved in CH_2Cl_2 (10 mL), loaded onto Celite (1 g) and purified via automated silica gel flash chromatography on a pre-equilibrated silica gel column (Biotage Sfär HC Duo 10 g silica column, 9-mL fractions, 40 mL/min flow rate), eluting with hexanes/EtOAc (100:0) to hexanes/EtOAc (70:30) over 9 min to afford **19'** (145 mg, 86% yield) exhibiting spectral data identical to the racemate (**19**).

Data for **19'**:

HPLC: t_R = 20.114 min and t_R = 28.013 min (ADR-H, MeCN/H_2O, 40:60, 1.0 mL/min; 210 nm; 29.5 °C).

e.r and % ee: 48:52, 4% ee

Preparation of (*S*)-2-(4-Chlorophenyl)-3-((triethylsilyl)oxy)propane-1,2-diol (20'):

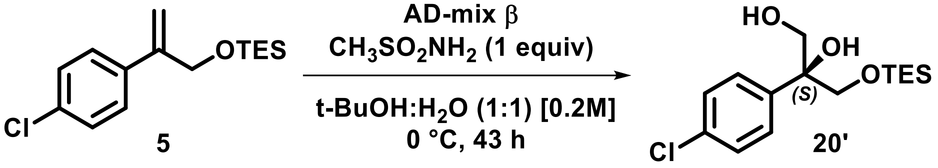

Following **General Procedure for SAD**, AD-mix β (496 mg, 0.64 mmol, 1.80 equiv), methanesulfonamide (33.6 mg, 0.354 mmol, 1.00 equiv), *t*-BuOH (1.17 mL) and H_2O (1.77 mL) were added to the scintillation vial, stirred at 20 °C until homogeneous and then cooled to 0 °C. In a separate 2-dram vial, a *t*-BuOH solution (0.30 mL) of **5** (100 mg, 0.354 mmol) was prepared then added in one portion to the reaction vial. The 2-dram vial was rinsed with *t*-BuOH (0.30 mL) and then added in one portion to the reaction vial. The reaction was allowed to stir for 43 h and monitored for completion via TLC. Upon completion, sodium sulfite (513 mg) was added as a solid at 0 °C, the mixture stirred for 1 h and then transferred to a 125-mL, separatory funnel with the assistance of CH_2Cl_2 (5 x 5 mL rinses). Brine (25 mL) was added and the phases were shaken, separated, and the organic layer collected. The aqueous layer was further extracted with CH_2Cl_2 (5 x 10 mL), the organic phases collected, combined, dried over Na_2SO_4 (5 g), filtered, and concentrated under reduced pressure (7.5 mmHg, 34 °C) to give an oil.

The oil was dissolved in CH_2Cl_2 (10 mL), loaded onto Celite (750 mg) and purified via automated silica gel flash chromatography on a pre-equilibrated silica gel column (Biotage Sfär HC Duo 10 g silica column, 9-mL fractions, 40 mL/min flow rate), eluting with hexanes/EtOAc (100:0) to hexanes/EtOAc (80:20) over 12 min to afford **20'** (90 mg, 80 % yield) exhibiting spectral data identical to the racemate (**20**).

Data for **20'**:

HPLC: t_R = 4.769 min and t_R = 6.157 min (Whelk, hexanes/*i*-PrOH, 90:10, 1.0 mL/min; 215 nm; 24 °C).

e.r and % ee: 16:84, 68% ee

Opt. Rot.: $[\alpha]_D^{24}$ -0.73 (c = 5.29 in $CHCl_3$)

Preparation of (*S*)-2-(2-Chlorophenyl)-3-((triethylsilyl)oxy)propane-1,2-diol (21'):

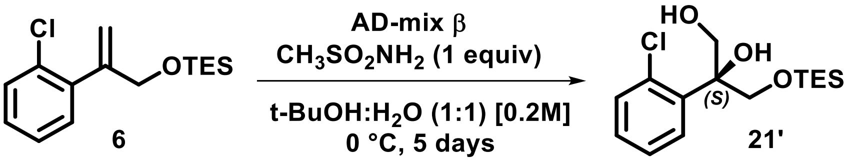

Following **General Procedure for SAD**, AD-mix β (500 mg, 0.64 mmol, 1.80 equiv), methanesulfonamide (17 mg, 0.18 mmol, 1.00 equiv) *t*-BuOH (1.20 mL) and H_2O (1.80 mL) were added to the scintillation vial, stirred at 20 °C until homogeneous and then cooled to 0 °C. In a separate 2-dram vial, a *t*-BuOH solution (0.30 mL) of **6** (50 mg, 0.18 mmol) was prepared then added in one portion to the reaction vial. The 2-dram vial was rinsed with *t*-BuOH (0.30 mL) and then added in one portion to the reaction vial. The reaction was allowed to stir for 96 h and an additional solution of AD-mix β (500 mg, 0.64 mmol, 1.80 equiv) in *t*-BuOH:H_2O (0.88 mL:0.88 mL) was added. The reaction was stirred for an additional 20 h. Upon completion, sodium sulfite (261 mg) was added as a solid at 0 °C, the mixture stirred for 1 h and then transferred to a 125-mL, separatory funnel with the assistance of CH_2Cl_2 (5 x 5 mL rinses). Brine (25 mL) was added, and the phases were shaken, separated, and the organic layer collected. The aqueous layer was further extracted with CH_2Cl_2 (3 x 15 mL), the organic phases collected, combined, dried over Na_2SO_4 (5 g), filtered, and concentrated under reduced pressure (7.5 mmHg, 34 °C) to give an oil.

The oil was dissolved in CH_2Cl_2 (10 mL), loaded onto Celite (500 mg) and purified via automated silica gel flash chromatography on a pre-equilibrated silica gel column (Biotage Sfär HC Duo 5 g silica column, 9-mL fractions, 18 mL/min flow rate), eluting with hexanes/EtOAc (90:10) over 8 min to afford **21'** (50 mg, 89% yield) exhibiting spectral data identical to the racemate (**21**).

Data for **21'**:

HPLC: t_R = 8.838 min and t_R = 10.128 min (Whelk, hexanes/*i*-PrOH, 97:03, 1.0 mL/min; 220 nm; 24 °C).

e.r and % ee: 49:51, 2% ee

Preparation of (*S*)-2-Methyl-3-(naphthalen-1-yloxy)propane-1,2-diol (22'):

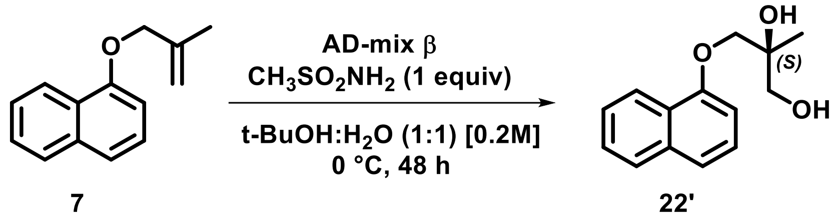

Following **General Procedure for SAD**, AD-mix β (707 mg, 0.91 mmol 1.80 equiv), methanesulfonamide (48 mg. 0.504 mmol, 1.00 equiv) *t*-BuOH (1.92 mL) and H_2O (2.52 mL) were added to the scintillation vial, stirred at 20 °C until homogeneous and then cooled to 0 °C. In a separate 2-dram vial, a *t*-BuOH solution (0.30 mL) of **7** (100 mg, 0.504 mmol) was prepared then added in one portion to the reaction vial. The 2-dram vial was rinsed with *t*-BuOH (0.30 mL) and then added in one portion to the reaction vial. The reaction was allowed to stir for 48 h and monitored for completion via TLC. Upon completion, sodium sulfite (717 mg) was added as a solid at 0 °C, the mixture stirred for 1 h and then transferred to a 125-mL, separatory funnel with the assistance of CH_2Cl_2 (5 x 5 mL rinses). Brine (25 mL) was added and the phases were shaken, separated, and the organic layer collected. The aqueous layer was further extracted with CH_2Cl_2 (5 x 10 mL), the organic phases collected, combined, dried over Na_2SO_4 (5 g), filtered, and concentrated under reduced pressure (7.5 mmHg, 34 °C) to give an oil.

The oil was dissolved in CH_2Cl_2 (10 mL), loaded onto Celite (1 g) and purified via automated silica gel flash chromatography on a pre-equilibrated silica gel column (Biotage Sfär HC Duo 10 g silica column, 9-mL fractions, 40 mL/min flow rate), eluting with hexanes/EtOAc (100:0) to hexanes/EtOAc (0:100) over 10 min to afford **22'** (104.7 mg, 89% yield) exhibiting spectral data identical to the racemate (**22**).

Data for **22'**:

HPLC: t_R = 11.491 min and t_R = 14.711 min (Supelco Astec, hexanes/*i*-PrOH, 80:20, 1.0 mL/min; 215 nm; 24 °C).

e.r and % ee: 3:97, 94% ee

Opt. Rot.: $[\alpha]_D^{24}$ -1.78 (c = 0.97 in EtOH)

Preparation of methyl (2*S*,3*R*)-2,3-Dihydroxy-4-phenylbutanoate (23'):

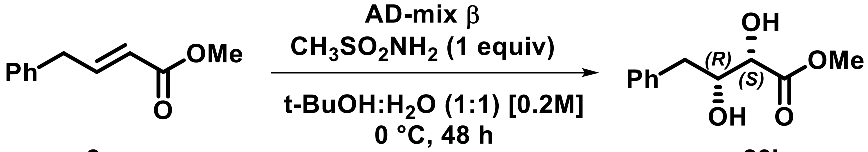

Following **General Procedure for SAD**, AD-mix β (796 mg, 1.02 mmol, 1.80 equiv), methanesulfonamide (54 mg, 0.568 mmol, 1.00 equiv), *t*-BuOH (1.84 mL) and H_2O (2.84 mL) were added to the scintillation vial, stirred at 20 °C until homogeneous and then cooled to 0 °C. In a separate 2-dram vial, a *t*-BuOH solution (0.50 mL) of **8** (100 mg, 0.568 mmol) was prepared then added in one portion to the reaction vial. The 2-dram vial was rinsed with *t*-BuOH (0.50 mL) and then added in one portion to the reaction vial. The reaction was allowed to stir for 48 h and monitored for completion via TLC. Upon completion, sodium sulfite (870 mg) was added as a solid at 0 °C, the mixture stirred for 1 h and then transferred to a 125-mL, separatory funnel with the assistance of CH_2Cl_2 (5 x 5 mL rinses). Brine (25 mL) was added and the phases were shaken, separated, and the organic layer collected. The aqueous layer was further extracted with CH_2Cl_2 (5 x 10 mL), the organic phases collected, combined, dried over Na_2SO_4 (5 g), filtered, and concentrated under reduced pressure (7.5 mmHg, 34 °C) to give an oil.

The oil was dissolved in CH_2Cl_2 (10 mL), loaded onto Celite (800 mg) and purified via automated silica gel flash chromatography on a pre-equilibrated silica gel column (Biotage Sfär HC Duo 10 g silica column, 9-mL fractions, 40 mL/min flow rate), eluting with hexanes/EtOAc (100:0) to hexanes/EtOAc (0:100) over 10 min to afford **23'** (95.6 mg, 80% yield) exhibiting spectral data identical to the racemate (**23**).

Data for **23'**:

HPLC: t_R = 19.290 min and t_R = 25.478 min (ADH, hexanes/*i*-PrOH, 90:10, 1.0 mL/min; 215 nm; 24 °C).

e.r and % ee: 97:3, 94% ee

Opt. Rot.: $[\alpha]_D^{24}$ +36.0 (*c* = 5.62 in $CHCl_3$)

Preparation of (3*S*,4*R*)-1-Tosylpiperidine-3,4-diol (24'):

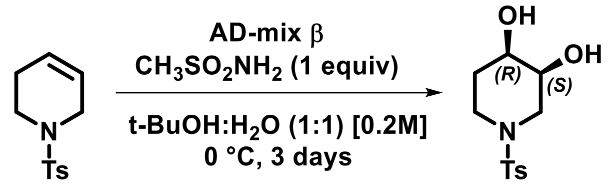

9 **24'**

Following **General Procedure for SAD**, AD-mix β (591 mg, 0.76 mmol, 1.80 equiv), methanesulfonamide (43 mg, 0.45 mmol, 1.10 equiv), *t*-BuOH (2.11 mL) and H_2O (2.11 mL) were added to the scintillation vial, stirred at 20 °C until homogeneous and then cooled to 0 °C. Then, **9** (100 mg, 0.42 mmol) was added in one portion to the reaction vial. The reaction was allowed to stir for 3 days and monitored for completion via TLC. Upon completion, sodium sulfite (632 mg) was added as a solid at 0 °C, the mixture stirred for 1 h and then transferred to a 125-mL, separatory funnel with the assistance of CH_2Cl_2 (5 x 5 mL rinses). Brine (25 mL) was added and the phases were shaken, separated, and the organic layer collected. The aqueous layer was further extracted with CH_2Cl_2 (4 x 10 mL), the organic phases collected, combined, dried over Na_2SO_4 (5 g), filtered, and concentrated under reduced pressure (7.5 mmHg, 34 °C) to give a white solid.

The solid was dissolved in CH_2Cl_2 (2 mL) with gentle heating. Then, hexanes (10 mL) was added and a white solid precipitated. The solid was filtered through a medium porosity fritted Buchner funnel and washed with pentane (20 mL) then the solid was dried under reduced pressure (0.1 mmHg, 23 °C) to give **24'** (102 mg, 89% yield) of a white solid exhibiting spectral data identical to the racemate (**24**).

Data for **24'**:

HPLC: t_R = 14.442 min and t_R = 18.565 min (OJH, hexanes/*i*-PrOH, 85:15, 2.0 mL/min; 240 nm; 24 °C).

e.r and % ee: 8:92, 84% ee

Opt. Rot.: $[\alpha]_D^{24}$ -21.6 (c = 2.43 in $CHCl_3$)

Preparation of (1*R*,2*S*)-7-Methoxy-1,2,3,4-tetrahydronaphthalene-1,2-diol (25'):

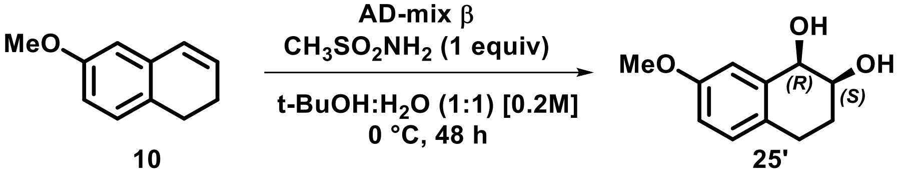

Following **General Procedure for SAD**, AD-mix β (840 mg, 1.08 mmol, 1.80 equiv), methanesulfonamide (57 mg, 0.60 mmol, 1.00 equiv), *t*-BuOH (3 mL) and H_2O (3 mL) were added to the scintillation vial, stirred at 20 °C until homogeneous and then cooled to 0 °C. Then, **10** (96 mg, 0.60 mmol) was added in one portion to the reaction vial. The reaction was allowed to stir for 48 h and monitored for completion via TLC. Upon completion, sodium sulfite (870 mg) was added as a solid at 0 °C, the mixture stirred for 1 h and then transferred to a 125-mL, separatory funnel with the assistance of CH_2Cl_2 (5 x 5 mL rinses). Brine (25 mL) was added and the phases were shaken, separated, and the organic layer collected. The aqueous layer was further extracted with CH_2Cl_2 (5 x 10 mL), the organic phases collected, combined, dried over Na_2SO_4 (5 g), filtered, and concentrated under reduced pressure (7.5 mmHg, 34 °C) to give an oil.

The oil was dissolved in CH_2Cl_2 (10 mL), loaded onto Celite (800 mg) and purified via automated silica gel flash chromatography on a pre-equilibrated silica gel column (Biotage Sfär HC Duo 10 g silica column, 9-mL fractions, 40 mL/min flow rate), eluting with hexanes/EtOAc (100:0) to hexanes/EtOAc (0:100) over 10 min to afford **25'** (143.3 mg, 74% yield) exhibiting spectral data identical to the racemate (**25**).

Data for **25'**:

HPLC: t_R = 17.436 min and t_R = 20.434 min (OJH, hexanes/*i*-PrOH, 90:10, 1.0 mL/min; 215 nm; 24 °C).

e.r and % ee: 45:55, 10% ee

Preparation of (2*R*,3*S*)-2,3-Dihydroxybutyl 3,5-bis(trifluoromethyl)benzoate (26'):

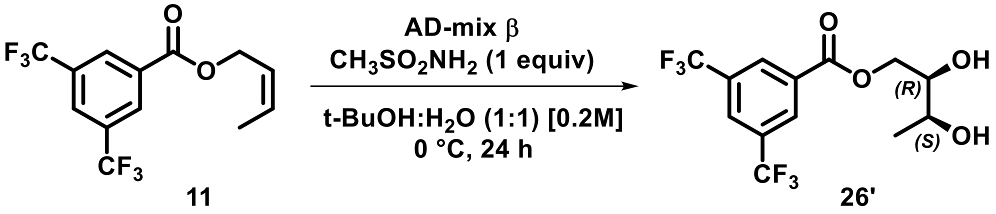

Following **General Procedure for SAD**, AD-mix β (160 mg, 0.21 mmol, 1.80 equiv), methanesulfonamide (11 mg, 0.11 mmol, 1.00 equiv), *t*-BuOH (0.36 mL) and H_2O (0.56 mL) were added to the scintillation vial, stirred at 20 °C until homogeneous and then cooled to 0 °C. In a separate 2-dram vial, a *t*-BuOH solution (0.10 mL) of **11** (35 mg, 0.11 mmol) was prepared then added in one portion to the reaction vial. The 2-dram vial was rinsed with *t*-BuOH (0.10 mL) and then added in one portion to the reaction vial. The reaction was allowed to stir for 24 h and monitored for completion via TLC. Upon completion, sodium sulfite (160 mg) was added as a solid at 0 °C, the mixture stirred for 1 h and then transferred to a 125-mL, separatory funnel with the assistance of CH_2Cl_2 (5 x 5 mL rinses). Brine (25 mL) was added and the phases were shaken, separated, and the organic layer collected. The aqueous layer was further extracted with CH_2Cl_2 (5 x 10 mL), the organic phases collected, combined, dried over Na_2SO_4 (5 g), filtered, and concentrated under reduced pressure (7.5 mmHg, 34 °C) to give an oil.

The oil was dissolved in CH_2Cl_2 (10 mL), loaded onto Celite (500 mg) and purified via automated silica gel flash chromatography on a pre-equilibrated silica gel column (Biotage Sfär HC Duo 5 g silica column, 9-mL fractions, 18 mL/min flow rate), eluting with hexanes/Et_2O (100:0) to hexanes/Et_2O (0:100) over 13 min to afford **26'** (14 mg, 36% yield) exhibiting spectral data identical to the racemate (**26**).

Data for **26'**:

HPLC: t_R = 7.506 min and t_R = 8.316 min (Supelco Astec, hexanes/*i*-PrOH, 90:10, 1.0 mL/min; 215 nm; 24 °C).

e.r and % ee: 48:52, 4% ee

Preparation of Ethyl (2*S*,3*R*)-2,3-Dihydroxy-3-phenylbutanoate (27'):

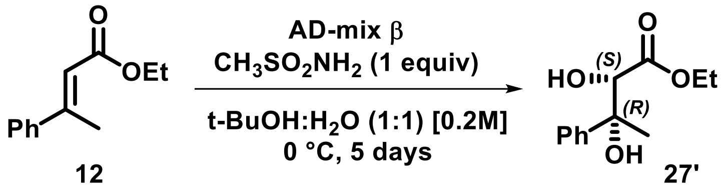

Following **General Procedure for SAD**, AD-mix β (370 mg, 0.47 mmol, 1.80 equiv), methanesulfonamide (25 mg, 0.26 mmol, 1.00 equiv), *t*-BuOH (0.70 mL) and H_2O (1.30 mL) were added to the scintillation vial, stirred at 20 °C until homogeneous and then cooled to 0 °C. In a separate 2-dram vial, a *t*-BuOH solution (0.30 mL) of **12** (50 mg, 0.26 mmol) was prepared then added in one portion to the reaction vial. The 2-dram vial was rinsed with *t*-BuOH (0.30 mL) and then added in one portion to the reaction vial. The reaction was allowed to stir for 5 days and monitored for completion via TLC. Upon completion, sodium sulfite (377 mg) was added as a solid at 0 °C, the mixture stirred for 1 h and then transferred to a 125-mL, separatory funnel with the assistance of CH_2Cl_2 (3 x 5 mL rinses). Brine (25 mL) was added and the phases were shaken, separated, and the organic layer collected. The aqueous layer was further extracted with CH_2Cl_2 (4 x 15 mL), the organic phases collected, combined, dried over Na_2SO_4 (5 g), filtered, and concentrated under reduced pressure (7.5 mmHg, 34 °C) to give an oil.

The oil was dissolved in CH_2Cl_2 (10 mL), loaded onto Celite (500 mg) and purified via automated silica gel flash chromatography on a pre-equilibrated silica gel column (Biotage Sfär HC Duo 5 g silica column, 9-mL fractions, 18 mL/min flow rate), eluting with hexanes/EtOAc (70:30) over 3 min to afford **27'** (44 mg, 75% yield) exhibiting spectral data identical to the racemate (**27**).

Data for **27'**:

HPLC: t_R = 11.011 min and t_R = 13.284 min (Supelco Astec, hexanes/*i*-PrOH, 90:10, 1.0 mL/min; 220 nm; 24 °C).

e.r and % ee: 96:4, 92% ee

Opt. Rot.: $[\alpha]_D^{24}$ +14.2 (c = 1.45 in $CHCl_3$)

Preparation of (*R*)-5-(Benzyloxy)-2-methylpentane-2,3-diol (28'):

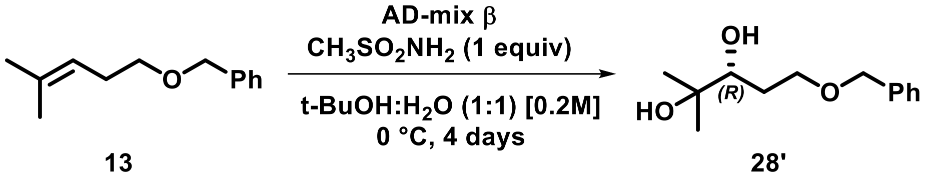

13 28'

Following **General Procedure for SAD**, AD-mix β (220 mg, 0.28 mmol, 1.80 equiv), methanesulfonamide (15 mg, 0.16 mmol, 1.00 equiv), *t*-BuOH (0.49 mL) and H_2O (0.79 mL) were added to the scintillation vial, stirred at 20 °C until homogeneous and then cooled to 0 °C. In a separate 2-dram vial, a *t*-BuOH solution (0.15 mL) of **13** (30 mg, 0.16 mmol) was prepared then added in one portion to the reaction vial. The 2-dram vial was rinsed with *t*-BuOH (0.15 mL) and then added in one portion to the reaction vial. The reaction was allowed to stir for 4 days and monitored for completion via TLC. Upon completion, sodium sulfite (240 mg) was added as a solid at 0 °C, the mixture stirred for 1 h and then transferred to a 125-mL, separatory funnel with the assistance of CH_2Cl_2 (5 x 5 mL rinses). Brine (25 mL) was added and the phases were shaken, separated, and the organic layer collected. The aqueous layer was further extracted with CH_2Cl_2 (4 x 10 mL), the organic phases collected, combined, dried over Na_2SO_4 (5 g), filtered, and concentrated under reduced pressure (7.5 mmHg, 34 °C) to give an oil.

The oil was dissolved in CH_2Cl_2 (10 mL), loaded onto Celite (500 mg) and purified via automated silica gel flash chromatography on a pre-equilibrated silica gel column (Biotage Sfär HC Duo 5 g silica column, 9-mL fractions, 18 mL/min flow rate), eluting with hexanes/EtOAc (100:0) to hexanes/EtOAc (25:75) over 12 min to afford **28'** (28.8 mg, 81% yield) exhibiting spectral data identical to the racemate (**28**).

Data for **28'**:

HPLC: t_R = 38.027 min (OJH, hexanes/*i*-PrOH, 99:01, 2.0 mL/min; 205 nm; 24 °C).

e.r and % ee: >99:1, >99% ee

Opt. Rot.: $[\alpha]_D^{24}$ -0.52 (c = 11.9 in $CHCl_3$)

Preparation of (S)-4-Hydroxy-2-methyl-6-phenylhexan-3-one (29'):

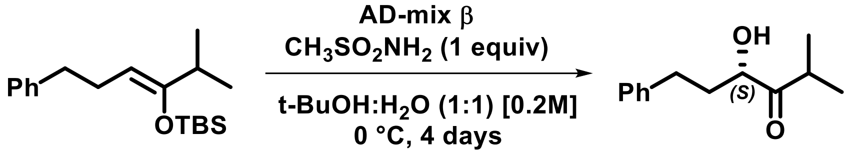

Following **General Procedure for SAD**, AD-mix β (180 mg, 0.23 mmol, 1.80 equiv), methanesulfonamide (12 mg, 0.13 mmol, 1.00 equiv), *t*-BuOH (0.46 mL) and H_2O (0.66 mL) were added to the scintillation vial, stirred at 20 °C until homogeneous and then cooled to 0 °C. In a separate 2-dram vial, a *t*-BuOH solution (0.10 mL) of **14** (40 mg, 0.13 mmol) was prepared then added in one portion to the reaction vial. The 2-dram vial was rinsed with *t*-BuOH (0.10 mL) and then added in one portion to the reaction vial. The reaction was allowed to stir for 4 days and monitored for completion via TLC. Upon completion, sodium sulfite (196 mg) was added as a solid at 0 °C, the mixture stirred for 1 h and then transferred to a 125-mL, separatory funnel with the assistance of CH_2Cl_2 (3 x 5 mL rinses). Brine (15 mL) was added and the phases were shaken, separated, and the organic layer collected. The aqueous layer was further extracted with CH_2Cl_2 (2 x 15 mL), the organic phases collected, combined, dried over Na_2SO_4 (5 g), filtered, and concentrated under reduced pressure (7.5 mmHg, 34 °C) to give an oil.

The oil was dissolved in CH_2Cl_2 (10 mL), loaded onto Celite (500 mg) and purified via automated silica gel flash chromatography on a pre-equilibrated silica gel column (Biotage Sfär HC Duo 5 g silica column, 9-mL fractions, 18 mL/min flow rate), eluting with hexanes/EtOAc (100:0) to hexanes/EtOAc (90:10) over 7 min to afford **29'** (16.6 mg, 61% yield) exhibiting spectral data identical to the racemate (**29**).

Data for **29'**:

HPLC: t_R = 10.641 min and t_R = 11.551 min (OJH, hexanes/*i*-PrOH, 95:5, 1.0 mL/min; 220 nm; 24 °C).

e.r and % ee: 96:4, 92% ee

Opt. Rot.: $[\alpha]_D^{24}$ -64.3 (c = 2.18 in $CHCl_3$)

Preparation of (3*R*,4*R*)-4-(4-Chlorophenyl)-3-methyl-1-tosylpiperidine-3,4-diol (30'):

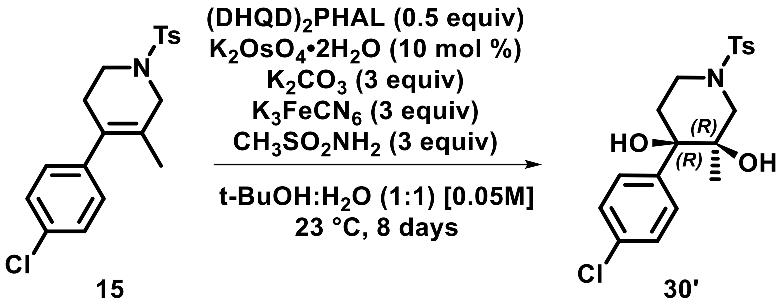

A 20-mL scintillation vial equipped with a cylindrical stir bar (2 cm x 1 cm, length x diameter) at 20 °C and under ambient atmosphere was charged with K_3FeCN_6 (145 mg, 0.44 mmol, 3.20 equiv), K_2CO_3 (57 mg, 0.41 mmol, 3.00 equiv), $(DHQD)_2PHAL$ (55 mg, 0.07 mmol, 0.50 equiv), K_2OsO_4•$2H_2O$ (5.3 mg, 0.014 mmol, 0.10 equiv), and methanesulfonamide (41 mg, 0.43 mmol, 3.10 equiv). Then, *t*-BuOH (1.38 mL) and H_2O (1.38 mL) were added and the vial capped with a green Teflon-lined septum cap. The mixture was stirred vigorously (700 rpm) at 20 °C on a magnetic stir plate until all the solids were dissolved to afford a light orange solution. Then, **15** (50 mg, 0.14 mmol) was added in one portion to the reaction vial and the mixture stirred for 8 days monitoring for completion every 24 h. Upon completion, sodium sulfite (210 mg) was added as a solid, the mixture stirred for 1 h and then transferred to a 125-mL, separatory funnel with the assistance of CH_2Cl_2 (5 x 5 mL rinses). Brine (25 mL) was added and the phases were shaken, separated, and the organic layer collected. The aqueous layer was further extracted with CH_2Cl_2 (4 x 15 mL), the organic phases collected, combined, dried over Na_2SO_4 (5 g), filtered, and concentrated under reduced pressure (7.5 mmHg, 34 °C) to give an oil.

The oil was dissolved in CH_2Cl_2 (10 mL), loaded onto Celite (2 g) and purified via automated silica gel flash chromatography on a pre-equilibrated silica gel column (Biotage Sfär HC Duo 25 g silica column, 9-mL fractions, 80 mL/min flow rate), eluting with hexanes/EtOAc (100:0) to hexanes/EtOAc (60:40) over 8 min to afford **30'** (42.8 mg, 78%) exhibiting spectral data identical to the racemate (**30**).

Data for **30'**:

HPLC: t_R = 13.692 min and t_R = 15.510 min (Supelco Astec, hexanes/*i*-PrOH, 85:15, 1.5mL/min; 230 nm; 24 °C).

e.r and % ee: 11:89, 78% ee

Opt. Rot.: $[\alpha]_D^{24}$ +9.7 (c = 2.12 in $CHCl_3$)

Computational Methods

All the chemical data presented in the database has been previously published, and the reader is referred to the original papers referenced in the database. All code, outputs, and files associated with the report can be found on the Github repository: https://github.com/SEDenmarkLab/SAD. All workflows were created with the *molli* python package developed to enable parallelized calculations on libraries of compounds.[15]

Semi-Automatic Workflow for Database Construction

Initial attempts to curate the SAD database included information on catalyst loading, substrate-catalyst ratio, concentration, equivalents of oxidant, conversion, and other essential components of the reaction. Unfortunately, problems arose with inconsistent reporting of the reagents used. Some groups reported the SAD reaction as "AD-mix α," which could be assumed to be the commercial mixture from Sigma-Aldrich™ containing 1.6 mmol $(DHQ)_2PHAL$, 0.7 mmol $K_2[OsO_2(OH)_4]$, 498.8 mmol K_2CO_3, and 498.8 mmol $K_3[Fe(CN)_6]$. Other groups would report using "AD-mix α," but then give direct quantities of each reagent added that would not match the commercial mixture or the reported Sharpless conditions.[14] The current database operates with key assumptions about the transformation by limiting the amount of additional data about the catalyst and conditions to promote homogeneity in entries, as well as lower the necessary time for data collection and accurate curation significantly.

Additional problems arose with the initial database curation. Multiple examples within preliminary searches documented both AD-mix α and β giving the same stereo-defined product, which were shown to be errors in tabulation by going to the primary source. Scifinder™ also failed to correctly assign the *E*/*Z* geometry of alkenes, even when directly enforced with their existing tools. These challenges also do not account for the chemical expertise necessary to remove non-representative examples, such as enantioselectivity being reported after crystallization, kinetic resolution being operative, or when essential reaction details are missing.

The semi-automatic workflow used for database construction is built to normalize all entries in this database. The database utilizes the YAML format, storing the IUPAC names of the library, RDKit[16] canonicalized SMILES[17] string, assignable nicknames for certain structures, and an assigned ID number for the various components. The numbered components are reactants,

products, oxidants, solvents, and additives. The workflow uses a series of questions associated with the SAD reaction for tabulation. An example literature entry used for this database, responses for construction of the entry, and the created entry are shown in Figure S1.[18] The current database stores the following outputs: reactant ID, reactant SMILES, product ID, product SMILES, catalyst ID, catalyst designation, whether it is a commercial/standard mixture (i.e. it may use just osmium(VIII) tetroxide instead of potassium osmate), mixture change notes, solvent 1 ID, solvent 1 SMILES, solvent 2 ID, solvent 2 SMILES, solvent 1 to solvent 2 ratio, oxidant ID, oxidant SMILES, oxidant solvent ID, oxidant solvent concentration, additive ID, additive SMILES, temperature (°C), time (hours), percent yield, %*ee*, er, $\Delta\Delta G^{\ddagger}$ (kcal/mol), multiple stereocenters, %*de*, dr, $\Delta\Delta G^{\ddagger}$ dr (kcal/mol), alkene type (Mono, Gem, etc.), DOI, and any additional notes. SMILES strings were generated for alkenes and diols with ChemDraw™, and SMILES strings were canonicalized with RDKit.

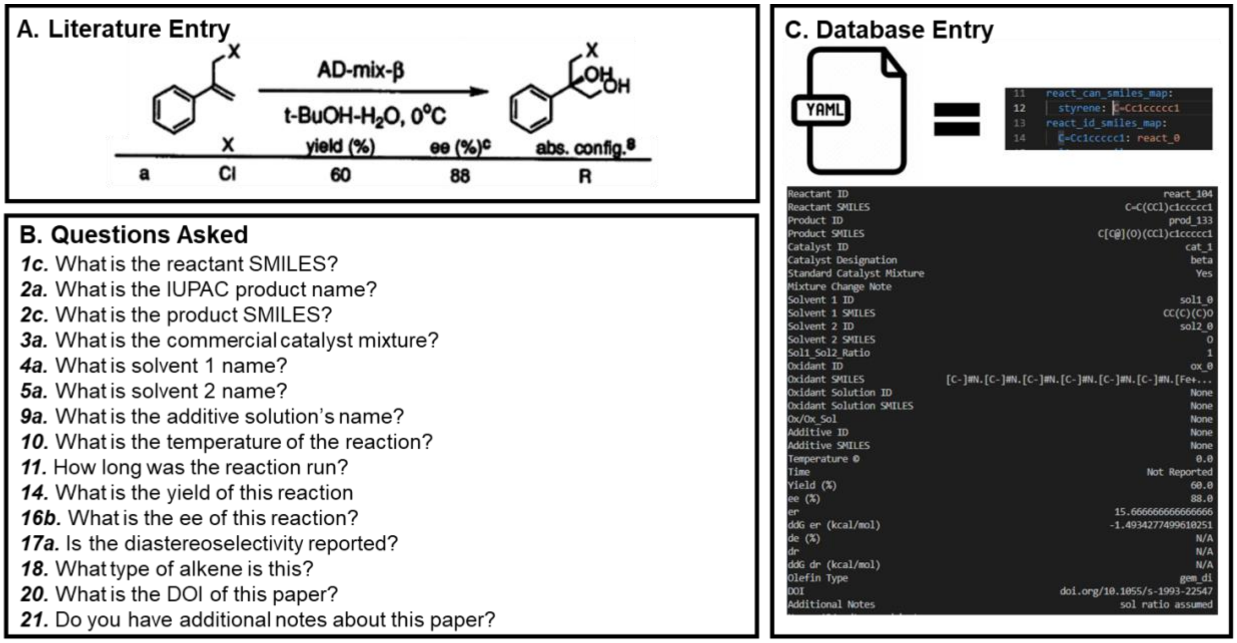

Figure S1. (**A**) Example literature entry.[18] (**B**) Questions asked to create the entry. (**C**) Final entry for the database and illustration of YAML format.

There is no information occupying the diastereoselectivity columns for this work, as this work focus on achiral alkenes, but this option is available for elaboration in future (formation of single diastereomers by stereospecific *syn* addition is assumed). In the case that temperature, time, and yield are not reported, the workflow script constructing the database automatically fills in "Not Reported" to those columns. This script takes the %*ee* and calculates the enantiomeric ratio (er)

and $\Delta\Delta G^{\ddagger}$ if the temperature is reported for the reaction. Internal checks are implemented in this workflow that will prevent errors in database compiling, such as ensuring values can be converted to floats and that entries cannot be repeated. The workflow was implemented with an internal check associated with the canonical SMILES of both reactants and products. The literature curated, and hence the current database, maintained that AD-mix α and β result in different diol enantiomers. The workflow is flexible to any potential changes in tabulation and can be adapted for use by a non-chemical expert for the creation of a chemically accurate and homogeneous database. The final database iteration can be found on the Github repository in the *db_tools* folder.

Alkene Descriptor Workflow

There are six steps associated with descriptor calculation: (1) 2D alkene identification, (2) 3D structure creation and force-field optimization, (3) canonicalization of RDKit and molli objects, (4) 3D alkene identification, (5) density functional theory (DFT) geometry optimization and initial alignment of alkenes by volume, (6) remaining descriptor calculation and realignment of alkenes. These steps can be found on the Github repository under *descriptor_workflow*.

Step 1: 2D Alkene Identification

Step 1 uses the inherent properties assigned from the molecular objects of RDKit and returns the location of the two carbon atoms of the alkene. An example using (*E*)-(2-methoxyvinyl)benzene is shown in Figure S2A. In this example, the RDKit molecule can be envisioned as a connectivity matrix made up of the SMILES string CO/C=C/c1ccccc1. The two alkene atoms, ***2*** and ***3***, are isolated by asking about the properties of the individual atoms that differentiate them: Which atoms are carbons, sp^2, and not aromatic?" The function then returns a string of "0011000000", or a True and False for all atoms. The identification may seem trivial with the availability of flexible querying architectures such as SMARTS,[19] but the complexity of the alkene dihydroxylations in the SAD database resulted in cases that could not be differentiated without various complex SMARTS strings. The alkene identification script was enabled with a combination of SMARTS and other more interpretable methods of atom filtration through the RDQueries module of RDKit.

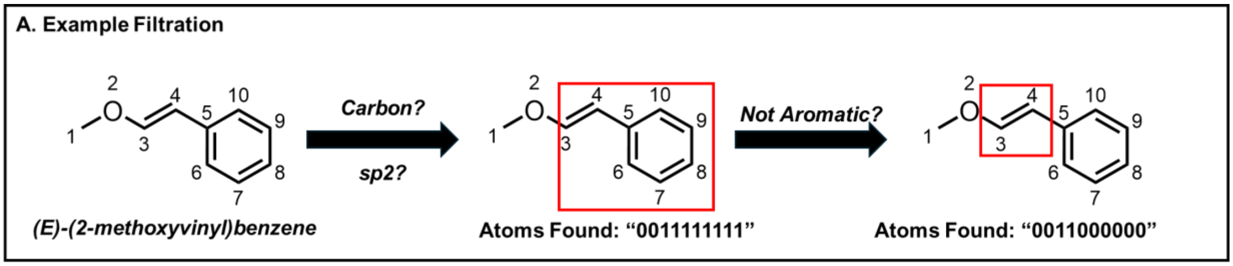

B. Atom Filters for Alkene Atoms

1. sp^2, carbon
2. sp^2, carbon, in one ring, no heteroatom neighbors, not aromatic
3. sp^2, carbon, no heteroatom neighbor, not aromatic
4. sp^2, carbon, one heteroatom neighbor, not in one ring, not aromatic, not C=O SMARTS query
5. sp^2, carbon, one heteroatom neighbor, in one ring, not aromatic, not C=O SMARTS query
6. sp^2, carbon, not three heteroatom neighbors, in one ring, not aromatic, not C=O SMARTS query
7. sp^2, carbon, not three heteroatom neighbors, not in two rings, not aromatic, not C=O SMARTS query
8. sp^2, carbon, not three heteroatom neighbors, not in two rings, not aromatic, in ring size 5, not C=O SMARTS query, not c1cscn1 SMARTS query
9. sp^2, carbon, not three heteroatom neighbors, not in two rings, not C=O SMARTS query, [NX3][CX3]=[CX3] SMARTS query
10. sp^2, carbon, [nX3H][cX3]([CX4])[cX3]([CX4]) SMARTS query
11. sp^2, carbon, not C=CC=C SMARTS query, [CX3]=[CX3][CX4][OH] SMARTS query
12. sp^2, carbon, not three heteroatom neighbors, c1[nH]c(=O)oc1 SMARTS query
13. sp^2, carbon, not two heteroatom neighbors, O=c(o)cc SMARTS query
14. sp^2, carbon, not aromatic
15. sp^2, carbon, in two rings, cc[n,o]c=O SMARTS query, not c1ccccc1 SMARTS query

Figure S2. (**A**) Visualization of how atoms are filtered to identify an alkene. (**B**) Filters used to isolate the alkenes in the SAD database.

The original atom filtration system implemented in the molli python package was extended to enable this workflow, allowing visualization of alkenes and checking connectivity of the atoms found.[15] There were fifteen sequential atom filters applied to the 784 alkenes in the SAD database (Figure S2B), and all associated alkenes were visualized to confirm the expected behavior. All remaining polyenes used in the database were manually assigned values by visualization of the alkene and reported diol with RDKit. The Boolean array was then stored as a string of 1 or 0 as a property of the RDKit object for retrieval in later steps.

<u>Step 2-4: 3D Structure Creation, Canonicalization, and Alkene Identification</u>

Step 2 matched the RDKit object to the database and assigned the alkene type. Hydrogens were added to the RDKit object, 3D coordinates were generated, and the structures were optimized using the implementation of MMFF94 in RDKit.[20] The RDKit object was then loaded into molli as a 3D object and serialized as a *MoleculeLibrary*. Step 3 canonicalized the SMILES of the RDKit objects containing hydrogens and translated these to their respective 3D objects in molli. The molli python package uses an algorithm to reorder the list of atoms, bonds, and cartesian coordinates of molli objects to ensure the RDKit object and molli *Molecule* object can be used interchangeably. Step 4 then uses the same workflow described in Step 1 and extends this to the new RDKit objects

with hydrogens for identification of the alkene carbons associated with the 3D coordinates.

Step 5: Alkene Fragmentation and Sterimol/Volume Calculation

Step 5 consists of the following substeps: (1) density functional theory geometry optimization of the alkenes utilizing ORCA, (2) Sterimol and volume calculation of individual alkene quadrants, and (3) initial alignment of alkenes by volume of the substituent. All DFT calculations were performed with Orca 5.0.3[21] and NBO 7.0[22] with both geometry optimizations and population analysis realized at the B3LYP/def2-SVP level of theory.[23–25] Fragment-based descriptors are well-defined for branched alkenes; however, endocyclic and exocyclic alkenes must be fragmented on both sides of the alkene to create the unique fragment. For non-symmetrical alkenes, such as cyclic enol ethers, fragment-based descriptors must be capable of differentiating the side of attachment on the alkene. A breadth-first search was employed to identify the four unique substructures of the alkene substituents while maintaining the alkene carbon associated with the fragment as a handle. A visualization of the fragmentation for branched, exocyclic, and endocyclic alkenes is shown in Figure S3.

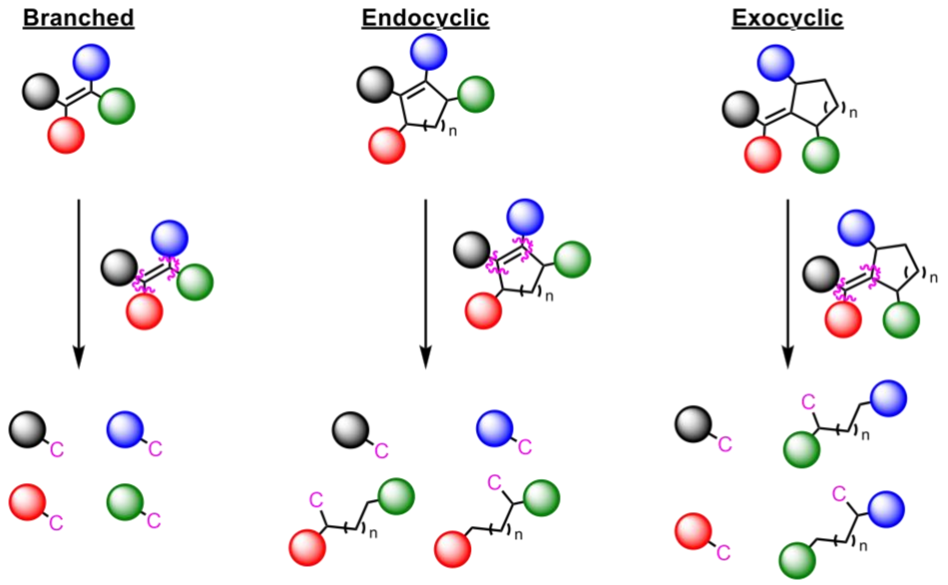

Figure S3. Visualization of alkene fragmentation into branched, exocyclic, or endocyclic constitution.

Sterimol parameters were calculated for each isolated substructure using the Morfeus implementation.[26–28] Verloop developed the method for this parameterization, which breaks down the dimensional properties of a molecule into three sub-parameters, B1, B5, and L. If looking at

the two-dimensional projection of the molecule down the axis to which the substituent is attached, B1 is the smallest length to the edge of the van der Waals radius, while B5 is the largest value to the edge of the van der Waals radius. L represents the longest length to the van der Waals radius projected to the axis to which the substituent is attached to, culminating in a low-dimensional description of a molecule that captures aspects of the volume in space that the substituent occupies. A visualization of the three parameters is shown in Figure S4A.[29]

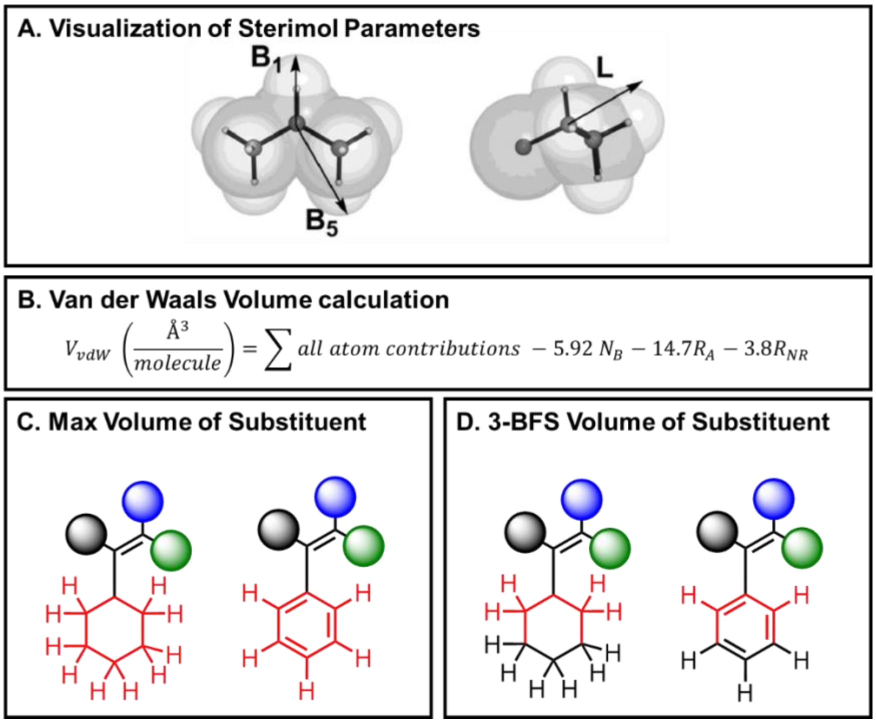

Figure S4.(**A**) Visualization of sterimol parameters. Reproduced from [29]. Available under a CC-BY license. Copyright © 2019 American Chemical Society. (**B**) Equation used for to calculate the volume of a substituent[30] (**C**) Visualization of the entire substituent being utilized for the Max Volume calculation (**D**) Visualization of the first three atoms of the substituent being utilized for the 3-BFS Volume calculation.

In addition, the atomic and bond contributions of van der Waals (vdW) volume (VABC) of the substituent was calculated as described by Zhao and co-workers.[30] The VABC method is an estimating equation (Figure S4B) derived from the contributions of atom vdW contributions and corrections derived from the number of bonds (N_B), the number of aromatic rings (R_A), and the number of non-aromatic rings (R_{NA}). The VABC method has high accuracy with respect to a wide range of organic vDW volumes. This work uses the reports from Truhlar on consistent vdW radii for individual atom vdW contributions.[31] Sterimol and the VABC method were used on both the entire substituents, referred to as Max Volume (Figure S4C), and a shortened substituent limited

to a three-atom breadth-first search. The three-atom breadth-first search variant of the volume is referred to as the "3-BFS Volume" (Figure S4D). Alkenes were then aligned by locating the substituent with the largest volume (**Q1**), and the remaining substituents were ordered **Q2**, **Q3**, and **Q4** in a counterclockwise orientation from the highest priority substituent. Two separate approaches are used, including the 3-BFS Volume and the Max Volume approach. This step does not account for potential symmetry and is meant as an initial alignment method.

Step 6: ESP, NBO, and RDF Descriptor Calculation

Step 6 consists of the remaining descriptor calculation: (1) separate molecule generation of a tetramethylammonium species for individual alkene substituents, (2) ESP_{MIN}, ESP_{MAX}, and ESP_{99} calculation,[32] (3) GFN2-XTB atom property calculation,[33] (4) CREST conformer generation, screening, and alignment,[34] (5) NBO calculation,[22] (6) merging of all descriptors into one structure, (7) final realignment of alkene structures with new descriptors, (8) final realignment of CREST conformers for RDF calculation. The same breadth-first search approach employed in alkene fragmentation was applied to generate structures that were then appended with a tetramethylammonium ion. This step was then followed by calculation of an electrostatic potential with a grid-spacing of 0.025 nm, range of 0.2 nm, and probe radius of 0.1 nm at the B3LYP level of theory with a 6-31G* basis set with no additional geometry optimization using the molli python package (Figure S5A). Electrostatic potentials were calculated utilizing NWChem 7.0.0.[35] Two unique problems were uncovered in this approach: iodides that were not parameterized with the 6-31G* basis set, and structures derived from alkynes ran into problems with undefined dihedral angles with the generation of arbitrary coordinates. These were redone manually with a 6-311G* and through disabling internal coordinates respectively. The calculated electrostatic potential represented by a grid of points was then parsed to retrieve the ESP_{MIN} and ESP_{MAX} which represents the minimum and the maximum of the grid.

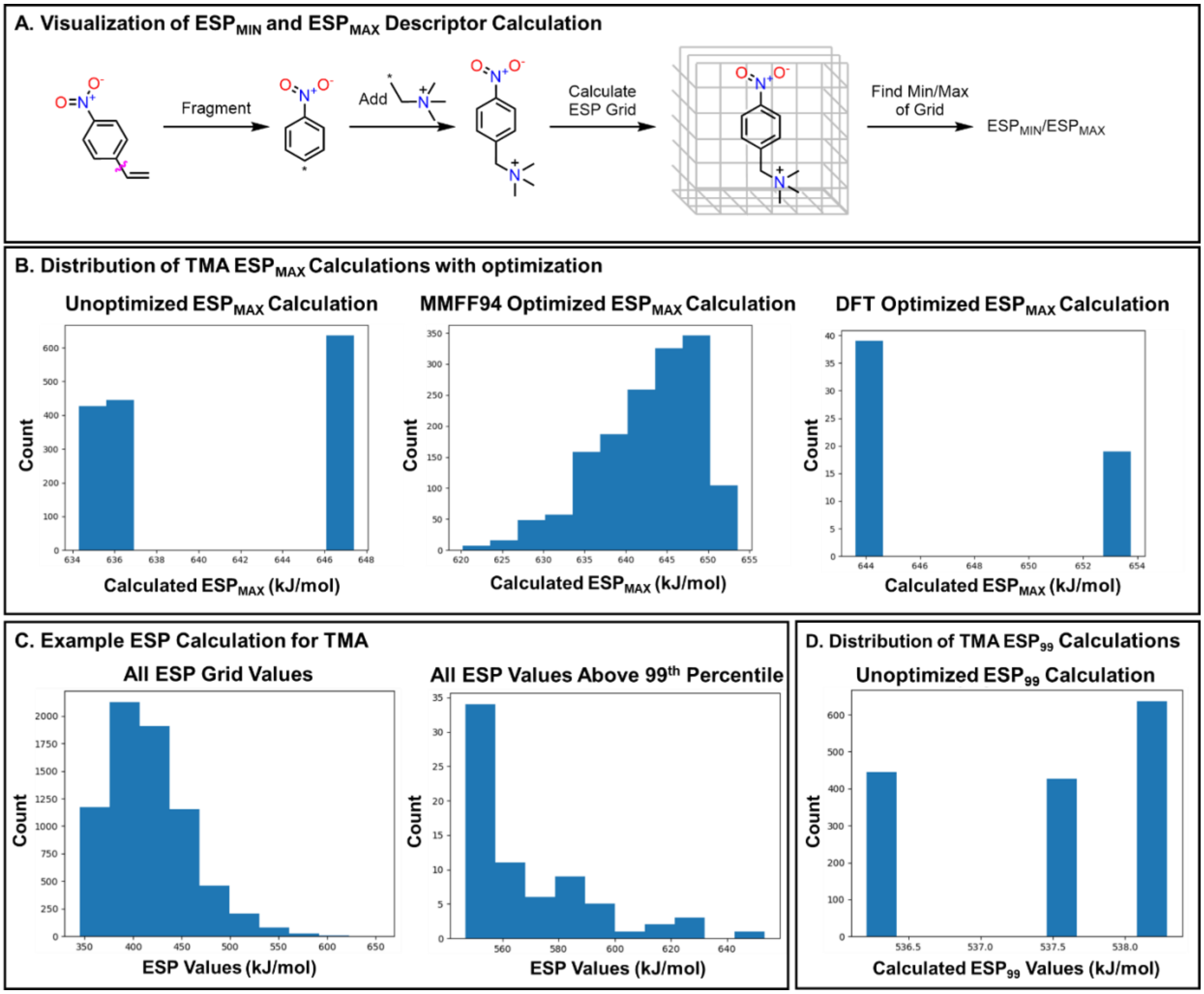

Figure S5.(**A**) Visualization of the ESPMIN/ESPMAX descriptor calculation for an alkene (**B**) Distribution of various ESPMAX values calculated for tetramethylammonium (TMA) with no optimization (left), MMFF94 optimization (middle), and DFT optimization (right) (**C**) Distribution of single TMA ESP calculation grid point values (left) and distribution of single TMA ESP calculation gird point values above the 99th percentile (right) (**D**) Distribution of various TMA ESP99 calculations.

An important problem encountered in control studies was the inconsistent calculations of the tetramethylammonium ion potentials in the ESPMAX calculation, varying by up to 13 kJ/mol. Additional investigations revealed that the calculation could vary up to 30 kJ/mol with MMFF94 optimized structures and by 10 kJ/mol with DFT optimized structures. (Figure S5B). Further investigation showed that although the structures were the same, the problem appeared because of errors in the grid of the electrostatic potential between hydrogen interactions in a tetramethylammonium structure. There was a large spread of electrostatic values within the top

99th percentile of calculated grid values were found when compared to the rest of the grid values (Figure S5C). This discrepancy was expected to result in inconsistent behaviors of the ESP_{MAX} descriptor between structures. In reproducibility studies, taking the 99th percentile value instead of the maximum consistently gave values within 1 kJ of each other for repeated tetramethylammonium electrostatic potential calculation, so the 99th percentile in the grid replaced the ESP_{MAX} descriptor, and it is referred to as ESP_{99} (Figure S5D).

Calculation of various atom properties, including charge, covalent coordination number,[36] Fukui indices,[37] Wiberg bond order[38], and polarizabilities.[33, 39] were done with GFN2-XTB version 6.6.1 on optimized structures from Step 5. Conformers were generated with CREST version 2.12 at the GFN-FF level of theory with a set temperature of 298.15 °C, energy window of 15.0 kcal/mol, and a topology check. This effect was then further evaulated at the GFN2-XTB level of theory with the same parameters as the search. These conformers were then aligned such that the **C1** alkene carbon was at the origin, **Q1** was in the XY plane, and the alkene atoms are along the X axis.

NBO[22] calculations were done with version 7.0 on the DFT-optimized structures from Step 5. NBO Analysis was employed to find the following descriptors: (1) DFT-level HOMO/LUMO energies, (2) natural charge of alkene carbon 1 and 2, (3) top 3 second order perturbation energies seen from bonds associated with the alkene, (4) π and π* energies of the alkene. All descriptors were then merged into a combined *MoleculeLibrary*. In addition, the ESP descriptors for quadrants containing only hydrogen (i.e. the ESP descriptor was calculated with only a tetramethylammonium ion) were updated to the median values over every calculation in the library. The median value for ESP_{MIN} was 342.28 kJ/mol, ESP_{MAX} was 636.33 kJ/mol, and ESP_{99} was 537.54 kJ/mol.

As described in the **Results and Discussion**, alkenes were aligned on the basis of six rules for both Max Volume and 3-BFS Volume. The workflow resulted in the true **C1** and true **Q1** being reassigned, and the remaining quadrants were then reassigned based on the new order. Alkenes were then realigned such that **C1** was at the origin, the alkene atoms were placed along the x axis, the molecule was rotated into the XY plane, and the alkenes were corrected to make sure **Q1** was placed consistently. Alkene types were then updated on the basis of these final assignments, including hydrogen location for the trisubstituted alkenes. After this update, the new alignment scheme was transferred to the *ConformerLibrary* for RDF calculation. RDF was calculated using

the previously calculated dispersion atomic properties for all alkenes at the **C1** and **C2** alkene carbons with an incremental length of 0.9 Å and no radial scaling. RDF bins atoms into 10 concentric spheres around each carbon atom of the alkene for each conformer, calculates the dispersion interaction energies[40] for atoms in each bin, sums their values, and averages it over the set of conformers to provide additional description of noncovalent interactions relevant to each carbon of the alkene (Figure S6). It is important to note that despite being represented by a non-covalent, dispersion phenomenon, this descriptor also inherently represents a description of the average steric occupancy and conformational flexibility owing to its conformer dependence. Smaller average values for the spheres can either indicate a lower occupation of the sphere because of atoms of the conformer rotating out of the sphere, or it can be indicative of atoms with a lower dispersion value occupying the sphere.

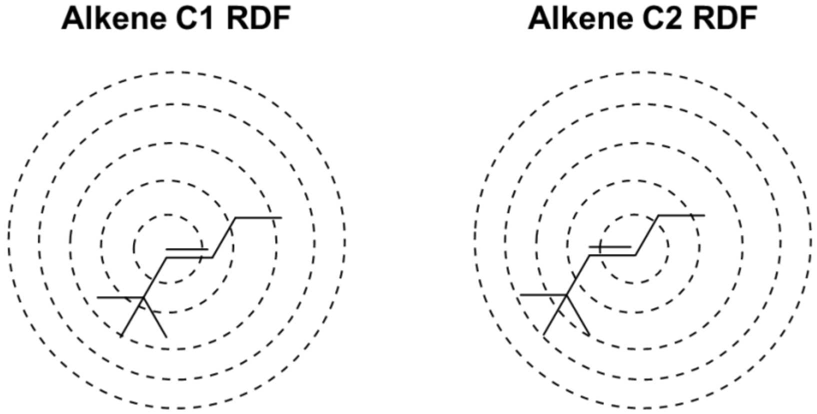

Figure S6. Visualization of the Alkene **C1** and **C2** RDF descriptors.

All descriptors were then concatenated to create a chemical space of 57 features. Descriptor matrix creation for individual alkene classes removed the fragments associated with a hydrogen atom. For example, the Mono alkene descriptor matrix would only have the 7 descriptors associated with **Q1** and then the remaining full alkene descriptors. The monosubstituted model had 36 features, the disubstituted models had 43 features, the trisubstituted models had 50 features, and the tetrasubstituted model had 57 features before any additional preprocessing or feature selection. The same approach was taken for the featurization of the experimental validation dataset and the external dataset.

Diol Alignment for Facial Selectivity Assignment

Six steps are implemented for diol descriptor matching and alignment: (1) 2D diol identification, (2) 3D structure creation, force-field optimization, and CIP assignment (3) canonicalization of RDKit and molli objects, (4) 3D diol identification, (5) alkene to diol mapping with descriptors and alignment, (6) assignment of top or bottom face dihydroxylation. These steps can be found on the Github repository under *descriptor_workflow*.

Descriptor calculation and alignment of diols are notably more challenging than the alkene case. Alkenes are geometrically rigid, but the corresponding diols are not. The product of the SAD is traditionally drawn with both hydroxyl groups on the same side because the SAD is a *syn*-stereospecific dihydroxylation. However, instantiation and subsequent geometry optimization of the 3D structures from the 2D diols can alter the orientation of hydroxyl groups owing to potential bond rotation around the carbon-carbon bond of the diol. The free rotation of this bond also makes the assignment of **Q1**/**Q2**/**Q3**/**Q4** and **C1**/**C2** for the alkene more complicated. In addition, there was no direct mapping between the 784 alkenes and 987 diols in SAD database curation, as the structures were transcribed with ChemDraw with independent atom ordering to maintain accurate chirality. The consequence of having no available mapping means that even if descriptors are calculated for the diols, they cannot be automatically assigned to the respective quadrants calculated on the alkene. The goal of this workflow is to enable geometrical assignment of top or bottom-face dihydroxylation rather than relying on the Cahn-Ingold-Prelog (CIP) rules. Many diols are not consistent with *R* and *S* assignments owing to inconsistent prioritization that can occur in molecules with highly diverse substituents and an inconsistent number of stereocenters upon dihydroxylation.

Steps 1-4: 2D Diol Identification, 3D Structure Creation, and 3D Diol Identification

These steps follow the same workflow as described in Steps 1-4 of the **Alkene Descriptor Workflow** to identify the diols and create canonicalized 3D structures. The main difference with this workflow and the previous workflow is that only six, atom filtration questions were necessary to isolate the diols in the SAD database. The sequential questions used to identify the diols are shown in Scheme S1.

1. Check 1
    - ❖ sp^3
    - ❖ carbon
    - ❖ CO SMARTS query
2. Check 2
    - ❖ sp^3
    - ❖ carbon
    - ❖ in one ring
    - ❖ one heteroatom neighbor
    - ❖ not [OR1X2][CR1X4]*[NR1X3][CR1X3](=O) SMARTS query
    - ❖ not COC SMARTS query
    - ❖ not cOC SMARTS query
    - ❖ not OCC*N SMARTS query
    - ❖ not OCCN SMARTS query
3. Check 3
    - ❖ sp^3
    - ❖ carbon
    - ❖ [OHX2][CH2][CX4][OHX2] SMARTS query
    - ❖ not [OHX2][CH2][CX4](C[OH])[OHX2] SMARTS query
    - ❖ not [OR1X2][CR1X4]*[NR1X3][CR1X3](=O) SMARTS query
    - ❖ not COC SMARTS query
    - ❖ not cOC SMARTS query
    - ❖ not OCC*N SMARTS query
    - ❖ not OCCN SMARTS query
4. Check 4
    - ❖ sp^3
    - ❖ carbon
    - ❖ [OHX2][CH2][CX4][OHX2] SMARTS query
    - ❖ not [OHX2][CX4][CX4](C[OH])[OHX2]
    - ❖ not [OHX2][CX4](C[OH])[CX4](C[OH])[OHX2] SMARTS query
    - ❖ not [OHX2][CX4](C[OH])[CX4][OHX2] SMARTS query
    - ❖ not [OR1X2][CR1X4]*[NR1X3][CR1X3](=O) SMARTS query
    - ❖ not COC SMARTS query
    - ❖ not cOC SMARTS query
    - ❖ not OCC*N SMARTS query
    - ❖ not OCCN SMARTS query
5. Check 5
    - ❖ sp^3
    - ❖ carbon
    - ❖ has a chiral tag
6. Check 6
    - ❖ sp^3
    - ❖ carbon
    - ❖ has a chiral tag OR [OHX2][CX4][CX4][OHX2] SMARTS query

Scheme S1. Sequential questions asked for diol identification. Each bullet point refers to an additional filter to isolate atoms.

Steps 5-6: Mapping Alkene Descriptors to Diols and Facial Selectivity Assignment

Step 5 creates a map from the alkene to the diol for geometrical assignment of top (α) or bottom (β)-face addition by identifying the substructures associated with the quadrants **Q1** and **Q4**. The alkene workflow prioritized alignment based on **Q1**, so this half of the molecule was chosen as the side for assignment of facial selectivity. The Step 5 workflow is as follows: (1) creation of **Q1Q4** and **Q1** substructures from the alkene (Figure S7A), (2) identification of the **Q1Q4** and **Q1** substructures on the diol (Figure S7B), and (3) alignment of the diol to match the **Q1Q4** orientation of the alkene (Figure S7C and Figure S7D).

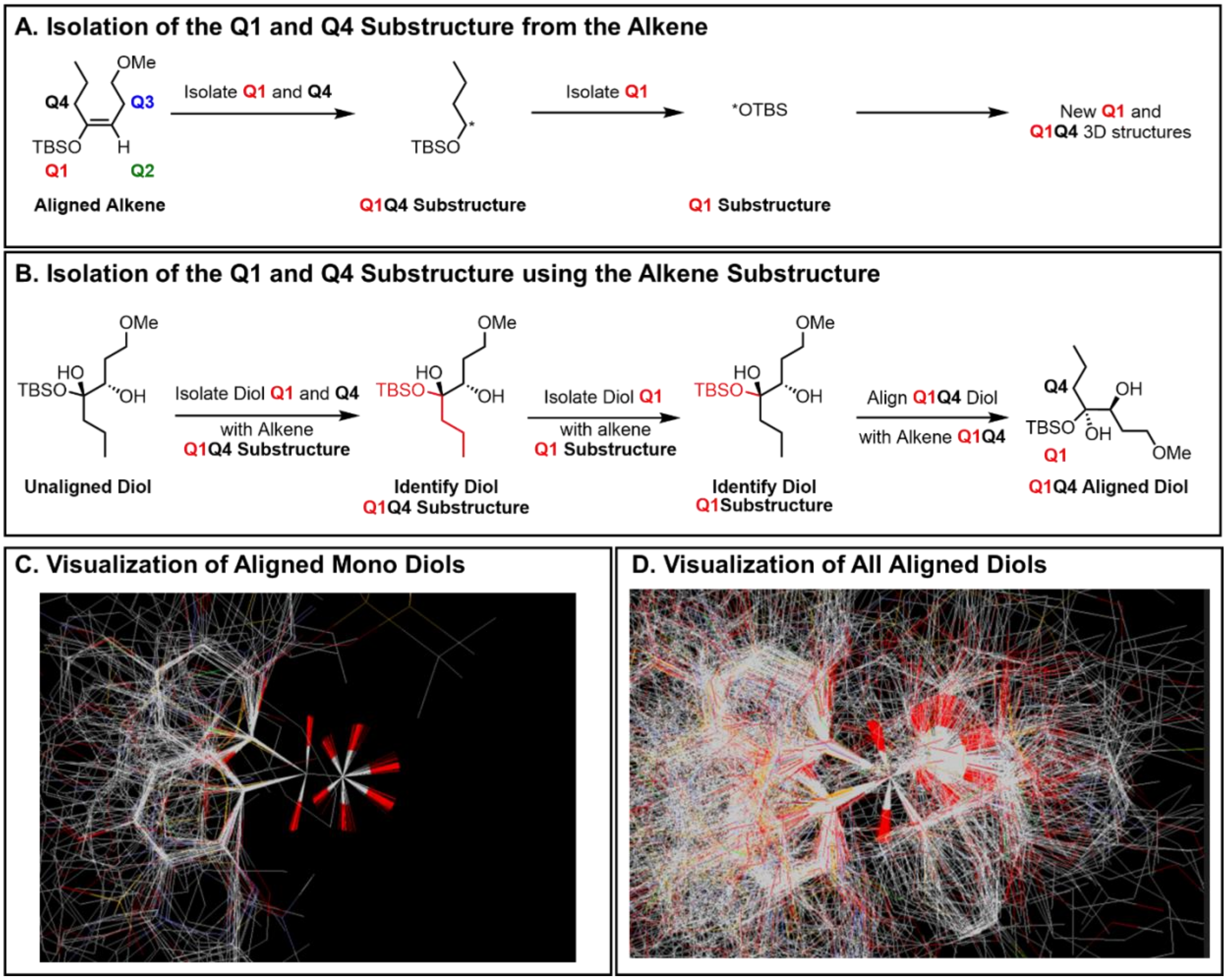

Figure S7. (**A**) Method for creation of **Q1** and **Q1Q4** substructure from the aligned alkene (**B**) Method for identification of **Q1** on the diol from the alkene substructure and subsequent alignment of the diol to match the alkene. (**C**) Visualization of diols aligned to their respective monosubstituted alkenes with the molli python package. (**D**) Visualization of all diols aligned to their respective alkenes with the molli python package.

Fragmentation of the alkene to create the **Q1Q4** and **Q1** substructures takes the same approach as shown in Step 5 of the **Alkene Descriptor Workflow** using molli *Molecule* objects to generate the fragmented 3D structures. The isolated substructures were transformed into NetworkX[41] graphs, and diol substructures were identified through iteration of subgraph isomorphisms with a max iteration of 10,000. A limit on subgraph matching was created because certain structures did not converge in a reasonable time. In addition, other structures ran into problems because of symmetrical substituents on the alkene. The Max Volume **Q1** substructure identification had a 4.6% failure rate over the 987 diols, while the 3-BFS Volume **Q1** substructure identification had a 10% failure rate. The worse performance of the 3-BFS Volume substructure identification is attributed to substituents being less differentiable in a lower number of subgraph isomorphism iterations. The **Q1** structures are more prone to being differentiated on electronic factors, which is not accounted for in the NetworkX graph construction. The ranges are

A. Performance of Substructure Matching for Max Volume Aligned Alkenes

Alkene Type	Alkenes Possible (Number)	Max Volume Alkenes Identified (Number)	Max Volume Alkene Failure Rate (%)	Diols Possible (Number)	Max Volume Diols Identified (Number)	Max Volume Diol Failure Rate (%)
Mono	148	148	0	180	180	0
Gem	148	142	4.1	161	154	4.3
Cis	41	40	2.4	49	48	2.0
Trans	250	231	7.6	316	295	6.6
TriQ2	62	58	6.4	83	76	7.0
TriQ3	37	36	2.7	51	50	2.0
TriQ4	77	75	2.6	117	114	2.6
Tetra	21	18	14.3	30	25	16.7
**Total**	784	748	4.6	987	942	4.6

B. Performance of Substructure Matching for 3-BFS Volume Aligned Alkenes

Alkene Type	Alkenes Possible (Number)	BFS Volume Alkenes Identified (Number)	BFS Volume Failure Rate (%)	Diols Possible (Number)	BFS Volume Diols Identified (Number)	Max Volume Diol Failure Rate (%)
Mono	148	148	0	180	180	0
Gem	148	128	13.5	161	140	13
Cis	41	40	2.5	49	48	2.5
Trans	250	218	12.8	316	280	11.3
TriQ2	71	56	0	96	72	0
TriQ3	38	35	0	54	50	0
TriQ4	67	64	0	101	96	0
Tetra	21	16	27.3	30	22	29
**Total**	784	705	10.1	987	888	10.0

Table S1. (**A**) Performance of substructure matching to diols from Max Volume aligned alkenes (**B**) Performance of substructure matching to diols from 3-BFS Volume aligned alkenes

summarized in Table S1A and Table S1B. Other subgraph matching platforms, including RDKit SMARTS substructure querying and enumeration of stereoisomers with subsequent canonicalization to match RDKit SMILES failed to give consistent performance.

Step 6 calculates the geometric assignment by comparing the direction of the normal vector of the alkene defined by **Q1**/**C1**/**C2** to the direction of the C-O bond defined in the hydroxyl group. The sign of the dot product of the two vectors indicates the direction of facial selectivity. If the vectors are in the same direction (i.e. the dot product is positive), this indicates top-face dihydroxylation. If the vectors are in the opposite direction (i.e. the dot product is negative), this indicates bottom-face dihydroxylation (Figure S8).

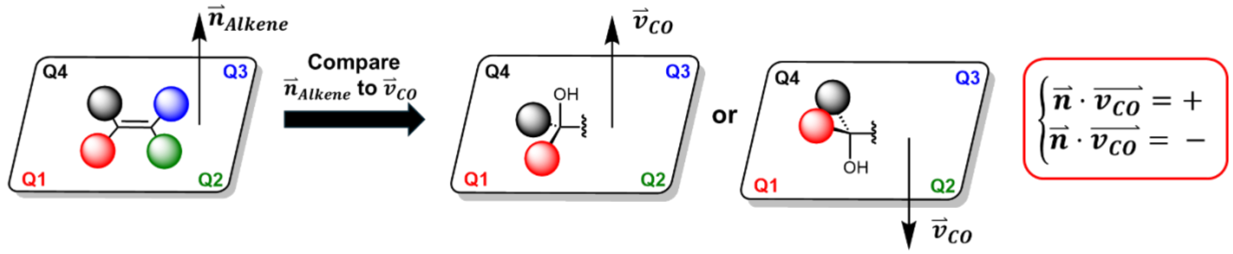

Figure S8 Illustration of how the dot product is taken between normal vector to the plane of the alkene and the C-O bond of the diol to identify if the dihydroxylation took place on the top face or bottom face with respect to **Q1**.

The compiled results for the Max Volume and 3-BFS Volume are shown in Figure S9. This figure is organized by alkene type, with blue representing top face dihydroxylation and orange representing bottom face dihydroxylation. In addition, the counts for each AD-mixture are separated with α on the left and β on the right. Although there were 54 more diols available with the Max Volume substructure recognition for assessment, the results presented herein instead focus on the intersection of the 888 diols recognized through the 3-BFS Volume alignment to allow for meaningful comparison in the limitations of both methods. The Max Volume alignment scheme shows strong performance on most classes, lining up with the Sharpless mnemonic, where AD-mix α gives bottom-face dihydroxylation and AD-mix β gives top-face dihydroxylation. The Max Volume alignment scheme fails to consistently match with the Sharpless mnemonic on the Cis and Tetra classes; however, these failures can be corrected with 3-BFS Volume alignment.

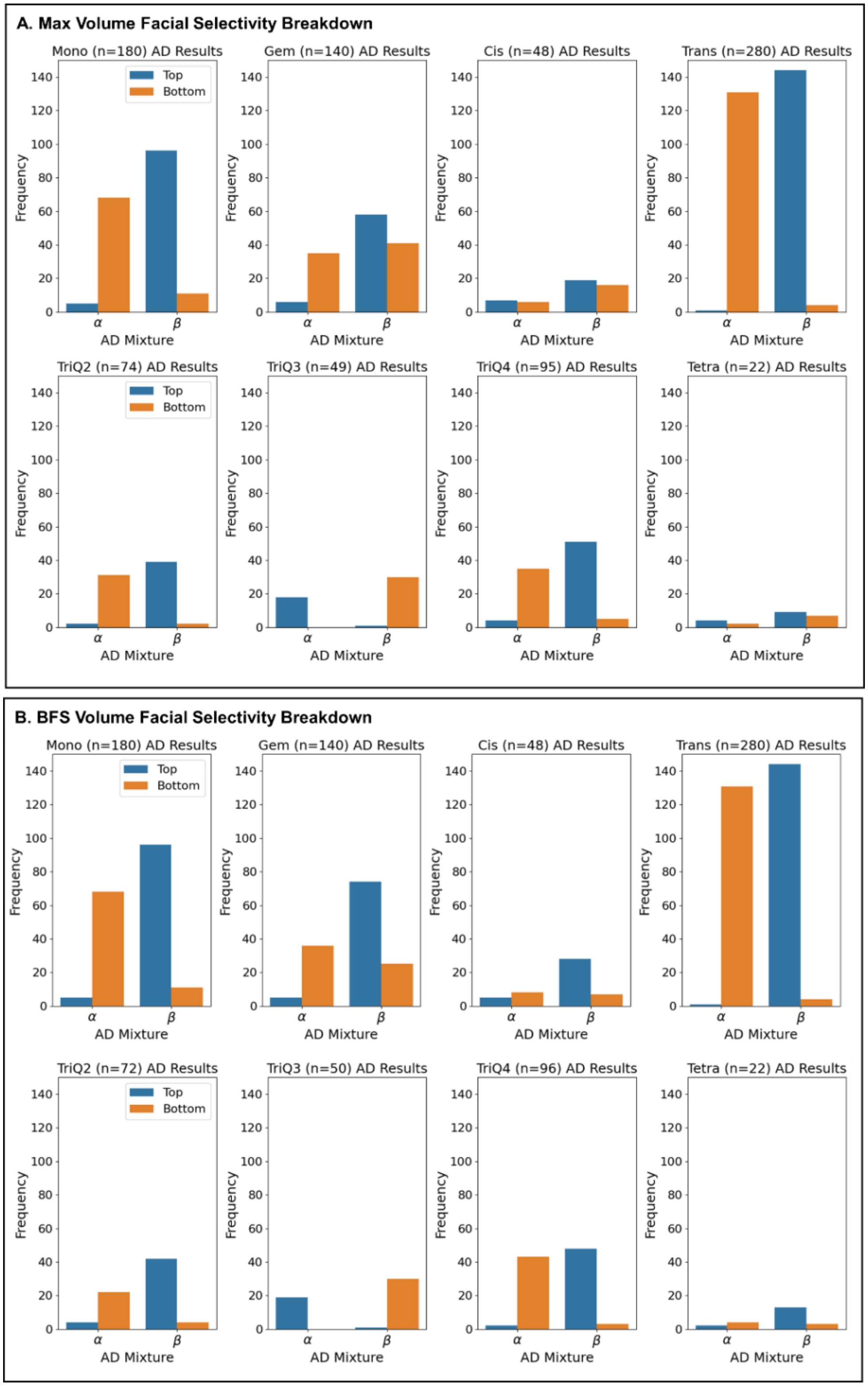

Figure S9. (**A**) Count plots of facial selectivity ordered by catalyst structure with a Max Volume alignment. Blue represents top-face dihydroxylation, while Orange represents bottom-face dihydroxylation. AD-mix α is shown on the left and AD-mix β is shown on the right. (**B**) Count plots of facial selectivity ordered by catalyst structure with a Max Volume alignment.

Notably, for both Max Volume and 3-BFS Volume alignment, the TriQ3 facial selectivity is consistently inverted compared to the other alkene classes. Although this appears to be inconsistent with the mnemonic, the flip is a natural consequence of the current alignment scheme. The traditional Sharpless/Norrby mnemonic states that AD-mix α adds from the bottom face of the alkene while AD-mix β adds from the top. The mnemonic is hypothesized to be driven by a strong, repulsive steric interaction in the **Q2** quadrant. In contrast, the current alignment scheme prioritizes the **Q1** assignment, which automatically locks the orientation of the other quadrants.

For TriQ3 alkenes, the smallest substituent is the hydrogen placed in **Q3**. To use the traditional mnemonic, the alkene would need to be geometrically flipped so the hydrogen in **Q3** would be placed in **Q2**. Since the orientation of the alkene is locked, the TriQ3 alkenes appear to show dihydroxylation on the incorrect face of the alkene. However, the dihydroxylation still maintains the correct facial selectivity, and the observed inversion of the TriQ3 facial selectivity can be rationalized by flipping the alkene to match the expected catalyst pocket or simply flipping the mnemonic itself. The TriQ3 inversion and the full alignment scheme appears to capture the necessary interactions of the individual quadrants, while remaining consistent with mnemonic. The TriQ3 mnemonic flip is visualized in Figure S10.

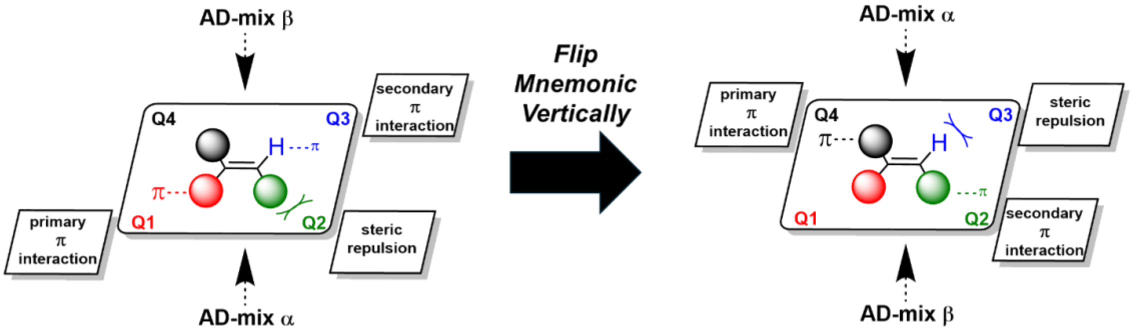

Figure S10. Representation of the TriQ3 mnemonic flip that gives inverted facial selectivity.

Chemical Space Visualization and Analysis

The SAD database chemical space can be divided into two different representations based on either the Max Volume alignment scheme, or the 3-BFS alignment scheme. Both are visualized as their respective score plots using the first two components of principal component analysis (PCA) in Figure S11.[42] The explained variance of the Max Volume space was 0.37, while the explained variance of the 3-BFS Volume space was 0.39. Both alignment schemes show significant

segmentation of the individual alkene classes relevant to the transformation. In the Max Volume aligned space (Figure S11A), TriQ3 alkenes are more closely related to the Cis alkene class, while TriQ2 is more closely related to the Trans alkene class, which is to be expected. The 3-BFS Volume score plot (Figure S11B) shows stronger segmentation of the individual classes, but less clear relationships between the individual classes in this space. E3FP fingerprints were also calculated, but they lost the differentiation among alkene classes and interpretability, so they were not considered as an optimal platform for further investigation.[43]

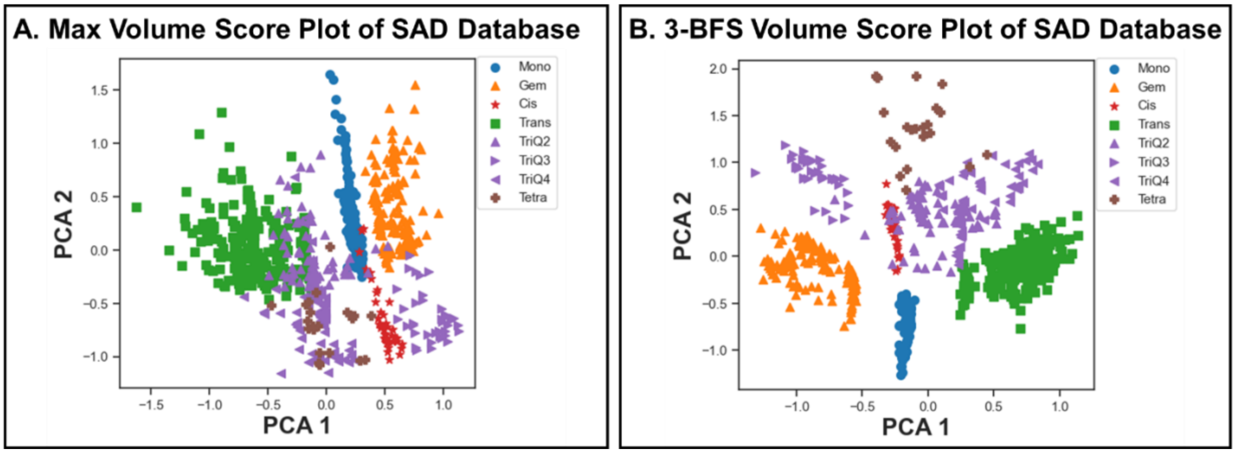

Figure S11. (**A**) Score plot of the SAD Database when aligned by the Max Volume (**B**) Score plot of the SAD Database when aligned by the 3-BFS Volume.

An external dataset of 2,524 alkenes was compiled with Scifinder^{TM} and parsed with OpenEye^{TM}.[44] The dataset was compiled using a substructure search of all alkene substitution patterns and isolated to any osmium-catalyzed dihydroxylation. A key difference with the creation of the external dataset over the original SAD Database was the use of the substructure search, whereas the SAD database functioned directly with established encyclopedias and keyword searches from Scifinder^{TM}. The same workflow was applied for alkene descriptor calculation, however some molecules failed at the descriptor calculation stage for problems in ESP calculation and conformer generation, resulting in 272 alkenes being skipped for the purposes of substrate scope evaluation. The PCA score plot of the external alkene dataset is shown in Figure S12. There is still significant segmentation of the various alkene classes with the external dataset, but it is less visible in the two-dimensional score plot due to the projection.

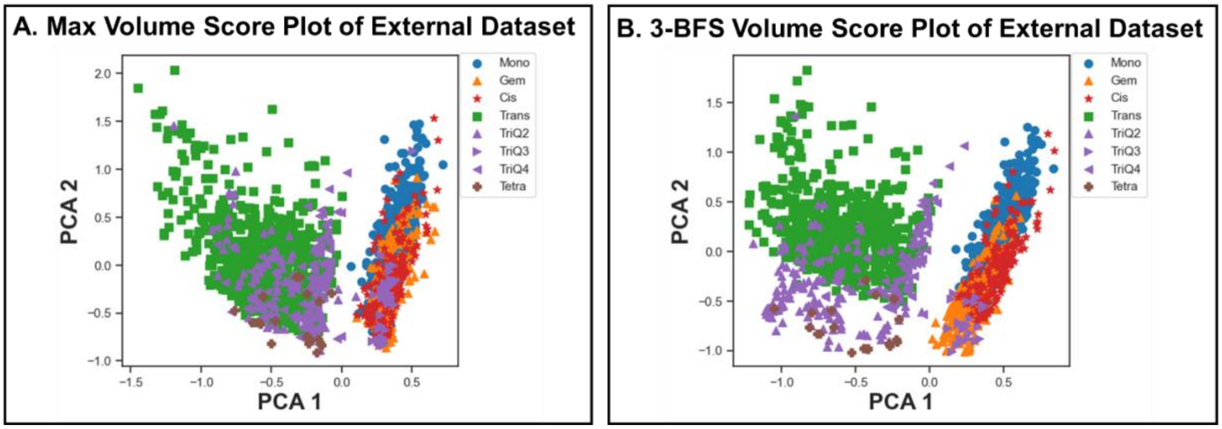

Figure S12. (**A**) Score plot of the external dataset of alkenes when aligned by the Max Volume (**B**) Score plot of the external dataset of alkenes when aligned by the 3-BFS Volume.

The explained variance of the external dataset with the Max Volume space is 0.41, whereas the explained variance of the 3-BFS Volume space is 0.42. The magnitude of the explained variance in the external dataset is similar to the SAD database, suggesting that the SAD database covers a similar amount of chemical space as all osmium-catalyzed dihydroxylations despite having fewer alkenes (784 alkenes in the SAD database compared to 2,252 alkenes in the external dataset). The SAD database coverage of the chemical space can also be qualitatively seen in Figure S13. The external dataset does not contain every alkene in the SAD database, with only 343 alkenes of the 784 being present. The missing alkenes suggests that there is a significant difference when utilizing a substructure search as done with the external dataset in comparison to the key-word search and encyclopedia tabulation done for the original SAD database curation. The 3-BFS score plot of the combined SAD database and external dataset appears to have inverted the PCA 2 axis upon its combination (Figure S13B), but the overall space is consistent when the datasets are combined.

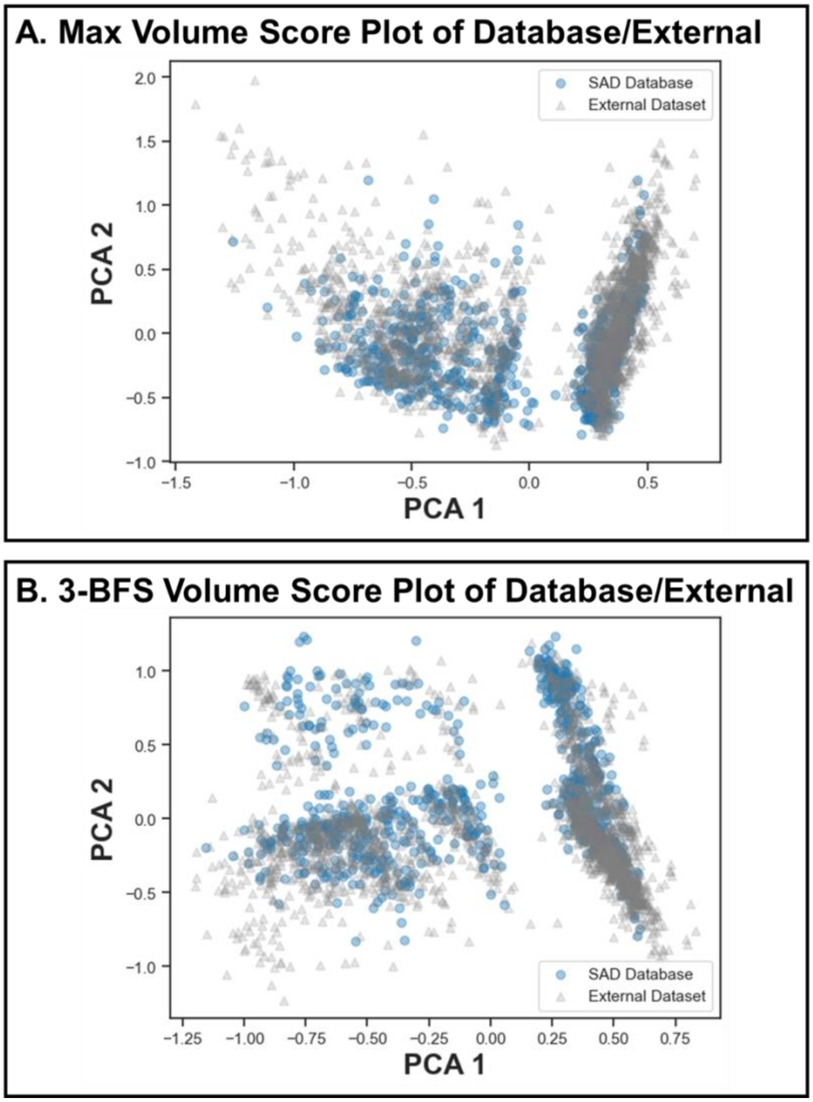

Figure S13. (**A**) Score plot of the combined SAD Database and external dataset of alkenes when aligned by the Max Volume (**B**) Score plot of the combined SAD Database and external dataset of alkenes when aligned by the 3-BFS Volume.

The location of the experimental validation in the chemical space is shown in Figure S14. Qualitative analysis of the experimental validation set suggests the experimental validation covers a large range of experimental space, with the 3-BFS volume alignment (Figure S14B) showing wider coverage than the Max Volume alignment space (Figure S14A).

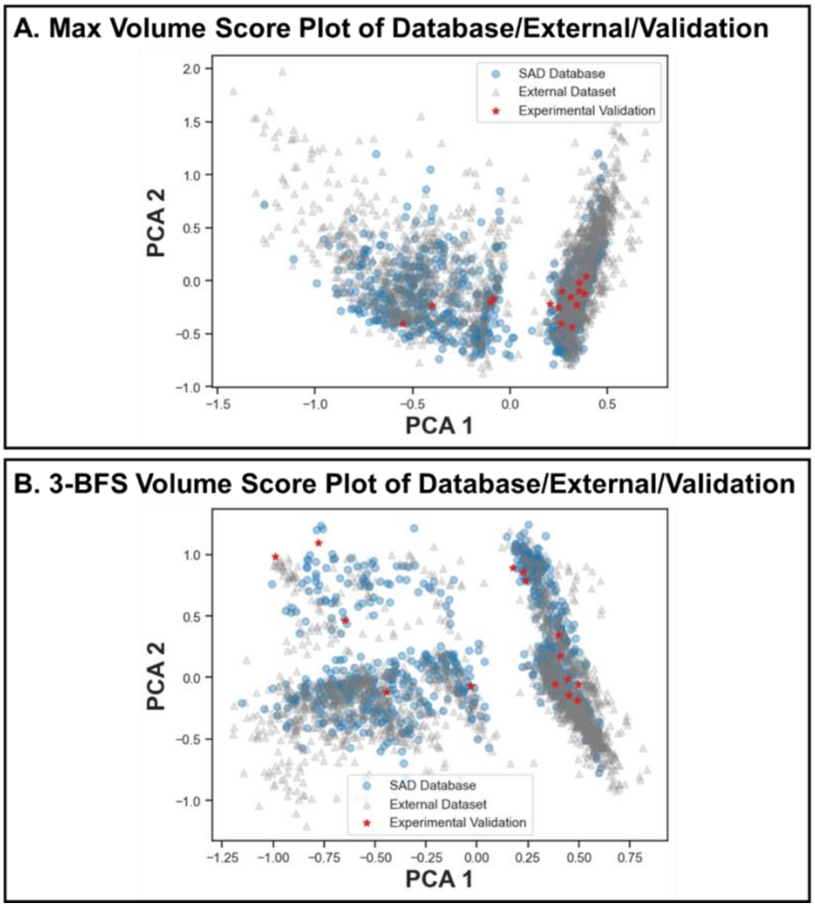

Figure S14. (**A**) Score plot of the combined SAD Database, external dataset of alkenes, and experimental validation descriptors when aligned by the Max Volume (**B**) Score plot of the combined SAD Database, external dataset of alkenes, and experimental validation descriptors when aligned by the 3-BFS Volume

Additional analysis of the pairwise similarities was conducted between the training set and test set of the individual models. These were done with Morgan Fingerprints with a radius of 3 and a fingerprint size of 2048.[45] To evaluate similarity between the fingerprints, the Tanimoto index was chosen due to its success in fingerprint-based similarity calculations.[46] The results are shown in Figure S15.

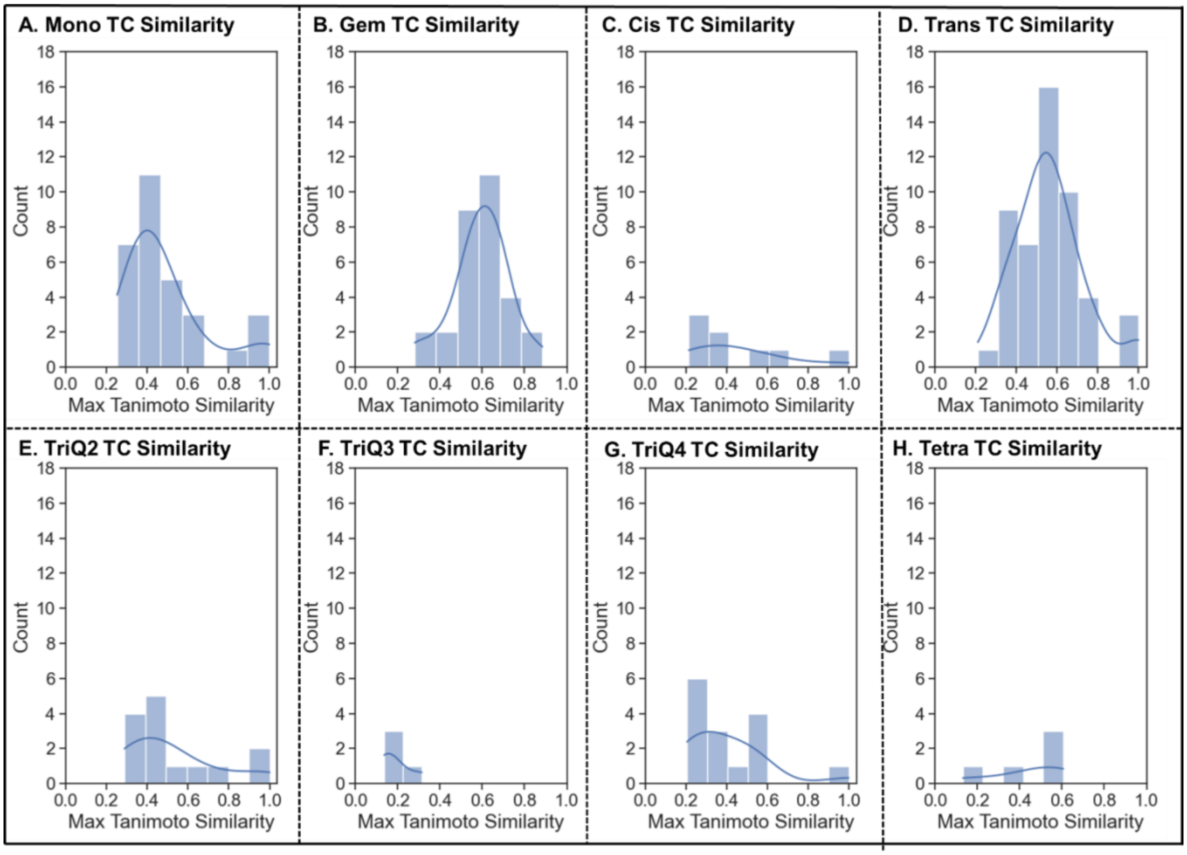

Figure S15. Histogram of maximum Tanimoto coefficient (TC) similarity values of the test set with respect to the training set for each alkene classes (**A**) Mono (**B**) Gem (**C**) Cis (**D**) Trans (**E**) TriQ2 (**F**) TriQ3 (**G**) TriQ4 (**H**) Tetra.

These results illustrate there is consistently a range of similarities within the test set when evaluating its similarity to the training set. Hence, prediction on the test set would provide a range of predictions that mix from easier to more challenging when modeling. The visualization of pairwise similarities was then extended to identify alkenes within a minimum Euclidean distance of the feature space. A standard scaling transform was applied to the train and test sets, the minimum pairwise distance in this representation was then calculated, and the closest alkene was identified in Euclidean distance. Although the distances are somewhat arbitrary in this representation, it functionally allows some understanding of the pairwise similarities in the current feature space. The distribution is shown below in Figure S16.

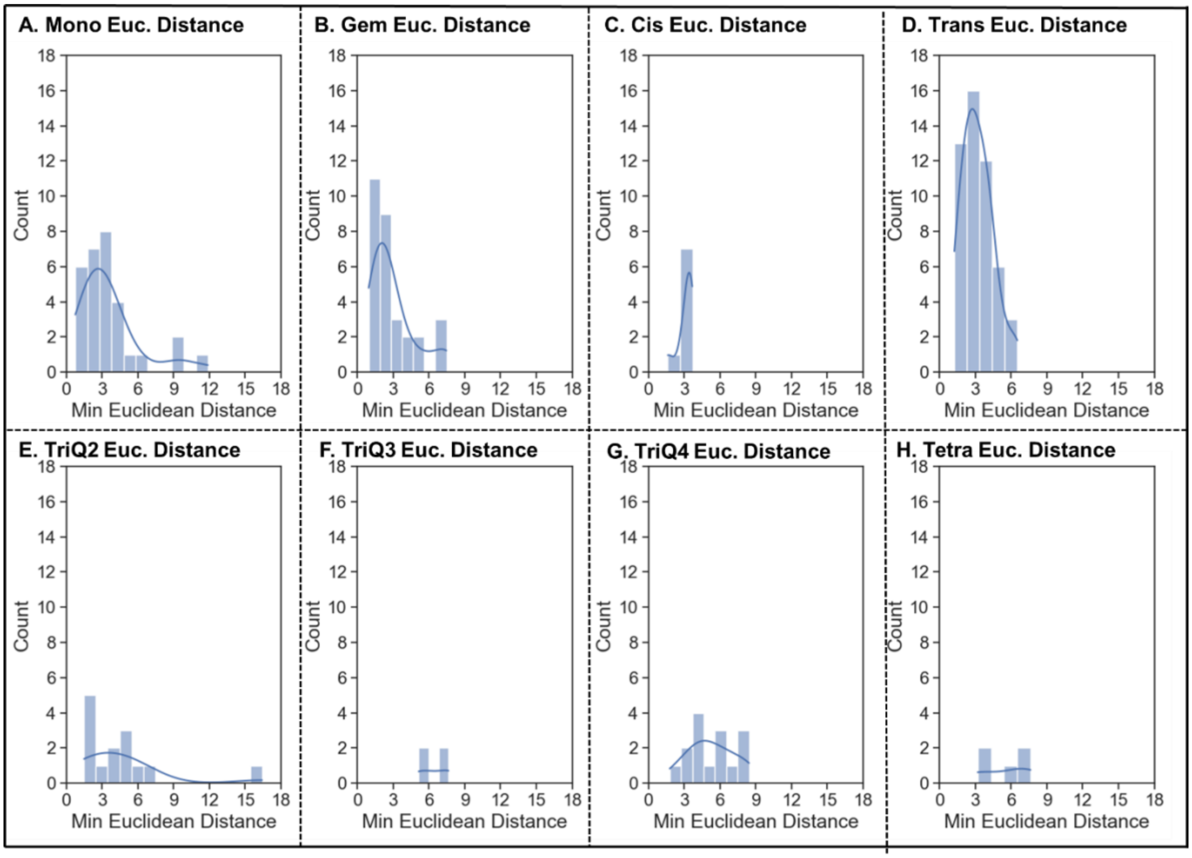

Figure S16. Histogram of minimum Euclidean (Euc.) distance of the test set with respect to the training set for each alkene classes (**A**) Mono (**B**) Gem (**C**) Cis (**D**) Trans (**E**) TriQ2 (**F**) TriQ3 (**G**) TriQ4 (**H**) Tetra.

In addition to the train/test similarity value visualization, the values for the experimental validation have been tabulated for the Tanimoto coefficients and minimum Euclidean distance for the experimental validation using the same method described above. These are shown in Table S2. Most compounds in the experimental validation show below a 0.5 Tanimoto Coefficient derived similarity. The table also illustrates that the embeddings provided from the fingerprint representation do not necessarily represent the same embeddings and distances as provided by the feature space.

Alkene Label	Maximum Tanimoto Similarity	Closest Alkene by Tanimoto	Observed ee (%) by Tanimoto	Minimum Euclidean Distance	Closest Alkene by Euclidean	Observed ee (%) by Euclidean
1	0.324	react_471	98.0	4.001	react_85	96.0
2	0.429	react_225	98.0	3.055	react_14	82.0
3	0.500	react_213	17.0	2.947	react_25	89.0
4	0.319	react_781	25.0	5.072	react_151	31.0
5	0.411	react_166	98.0	3.842	react_160	78.0
6	0.254	react_175	91.0	4.704	react_174	86.0
7	0.412	react_129	24.0	2.426	react_153	14.0
8	0.532	react_719	96.0	2.512	react_344	98.0
9	0.128	react_554	56.0	7.518	react_566	41.0
10	0.375	react_70	15.0	3.671	react_70	15.0
11	0.651	react_73	64.0	4.830	react_73	64.0
12	0.364	react_550	98.0	5.550	react_414	99.0
13	0.309	react_408	93.5	2.203	react_575	92.0
14 (TriQ2)	0.414	react_408	93.5	6.845	react_336	97.0
14 (TriQ3)	0.333	react_336	97.0	5.113	react_334	87.5
15	0.236	react_296	84.0	32.017	react_510	88.0

Table S2. Representation of maximum Tanimoto Coefficient found within the combined train/test set, as well as the minimum Euclidean distance provided by the specialized features. The closest alkene as defined by the Tanimoto Coefficient and the minimum Euclidean distance are provided.

Experimental Validation Selection

The selection of alkenes for experimental validation employed the combined SAD database and external alkene dataset as a platform for both selection and suggestion of potential ideas. Clustering analysis was done utilizing *k*-means[47] for each unique alkene class. The "Kneed" algorithm[48] was then implemented to evaluate the "elbow" defined by the distortion of these clusters and assign an optimal number of clusters to represent the space. Each cluster was visualized with RDKit to qualitatively evaluate the different alkene structures available in each class. The knee plots and subsequent *k*-means clustering analysis for Max Volume are shown below in Figure S17 and Figure S18. The knee plots and *k*-means clustering analysis for 3-BFS Volume are shown below in Figure S19 and Figure S20.

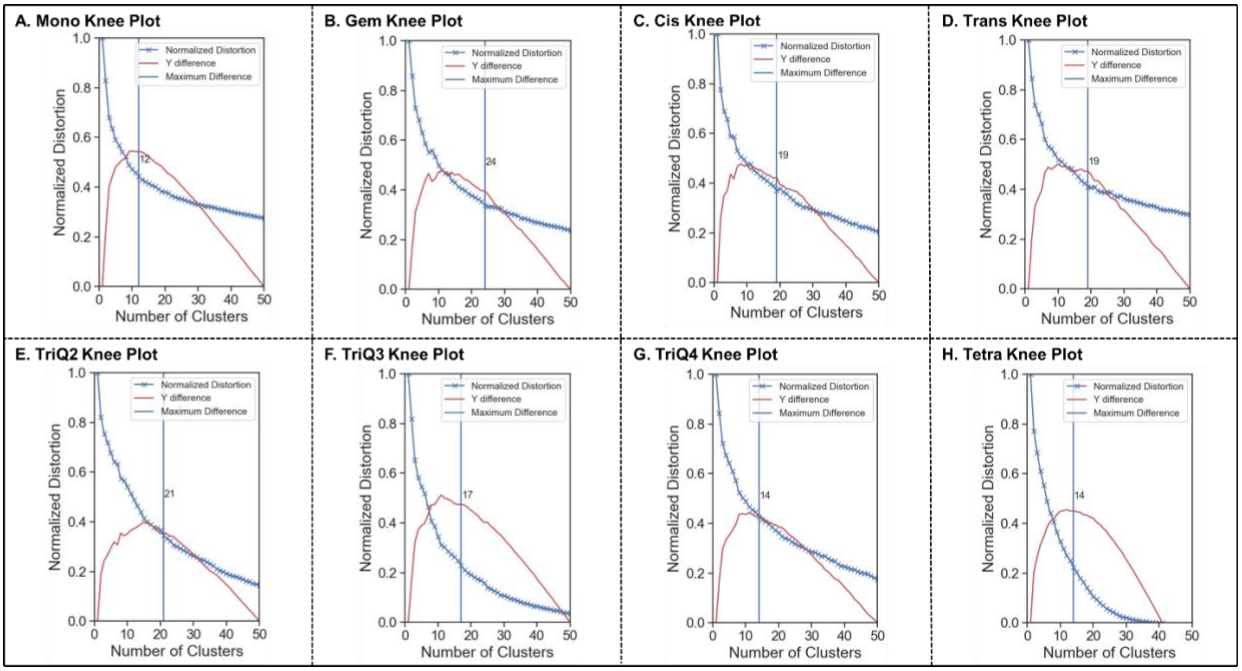

Figure S17. The knee plots for all alkene classes in the combined SAD database and external dataset when alkenes are aligned by Max Volume. The vertical line represents the location of the "knee," the decreasing blue line represents the normalized distortion of the *k*-means clustering analysis, and the red-line represents the difference in normalized distortion between one cluster and the previous cluster. (**A**) Mono (**B**) Gem (**C**) Cis (**D**) Trans (**E**) TriQ2 (**F**) TriQ3 (**G**) TriQ4 (**H**) Tetra.

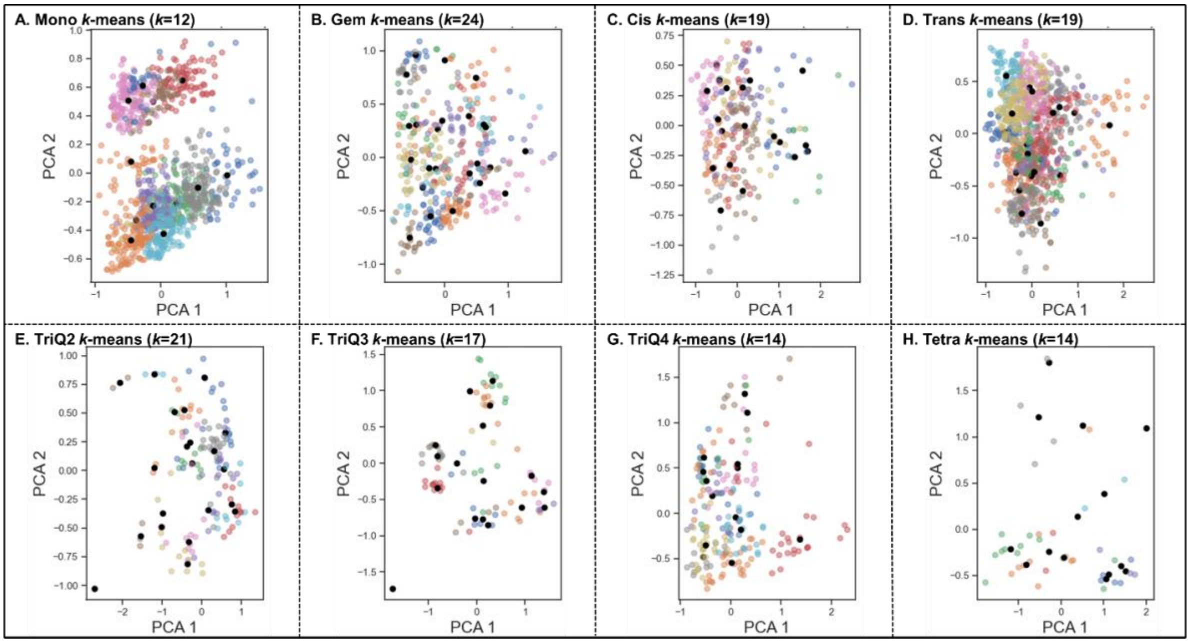

Figure S18. The *k*-means clustering analysis for all alkene classes in the combined SAD database and external dataset when alkenes are aligned by Max Volume, shown as a score plot with PCA analysis. Each color represents a different cluster, and each black dot represents a centroid. (**A**) Mono (**B**) Gem (**C**) Cis (**D**) Trans (**E**) TriQ2 (**F**) TriQ3 (**G**) TriQ4 (**H**) Tetra.

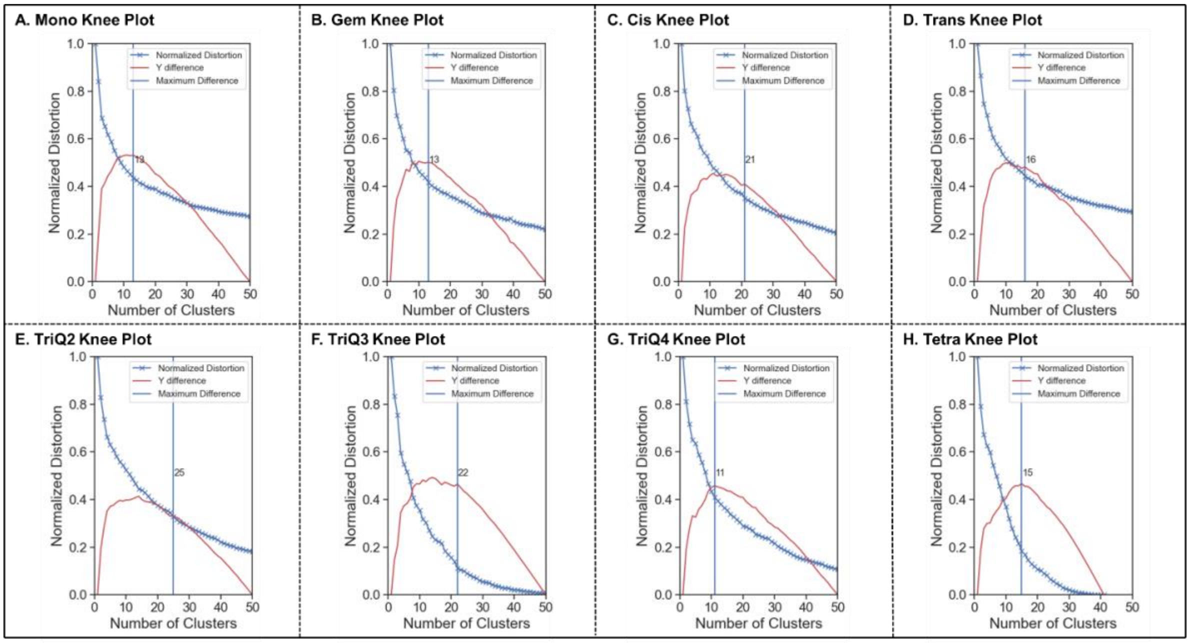

Figure S19. The knee plots for all alkene classes in the combined SAD database and external dataset when alkenes are aligned by 3-BFS Volume. The vertical line represents the location of the "knee," the decreasing blue line represents the normalized distortion of the *k*-means clustering analysis, and the red-line represents the difference in normalized distortion between a cluster and the previous cluster. (**A**) Mono (**B**) Gem (**C**) Cis (**D**) Trans (**E**) TriQ2 (**F**) TriQ3 (**G**) TriQ4 (**H**) Tetra.

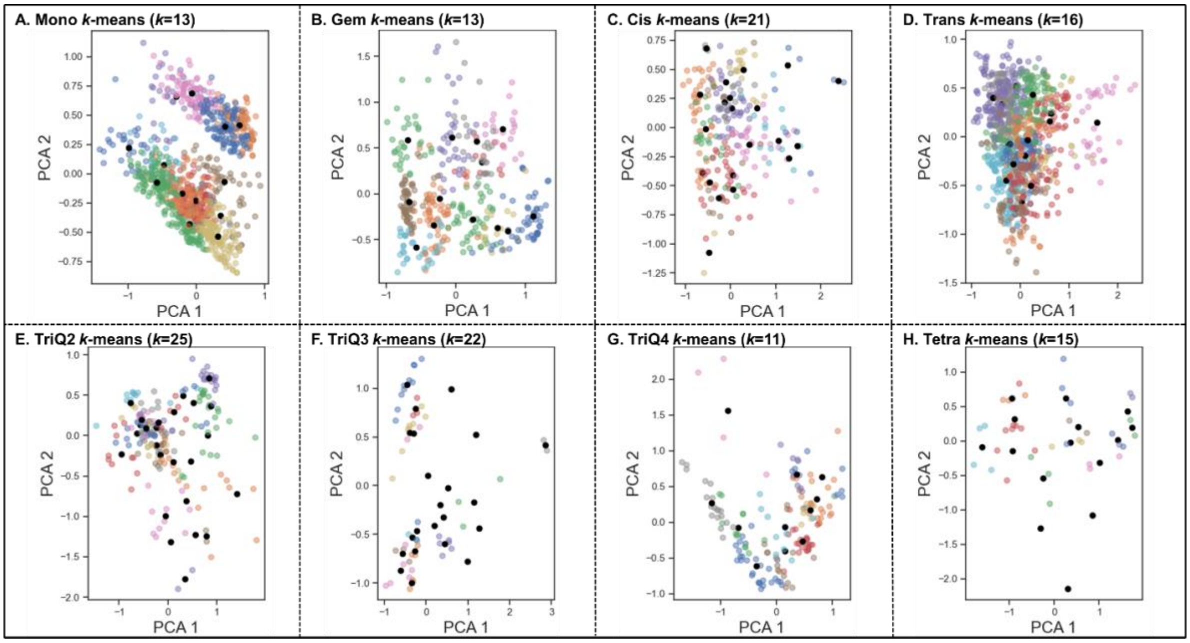

Figure S20. The *k*-means clustering analysis for all alkene classes in the combined SAD database and external dataset when alkenes are aligned by 3-BFS Volume, shown as a score plot with PCA analysis. Each color represents a different cluster, and each black dot represents a centroid. (**A**) Mono (**B**) Gem (**C**) Cis (**D**) Trans (**E**) TriQ2 (**F**) TriQ3 (**G**) TriQ4 (**H**) Tetra.

A few different approaches to selecting alkenes were attempted. The first method was strict algorithmic selection, in which alkenes were identified to find examples that maximize diversity, but the approach suffered from high synthetic complexity of the chosen alkenes. A combined algorithmic and chemical intuition approach was attempted to filter potential selected alkenes, but this approach gave alkenes whose synthesis would be prohibitively lengthy or costly from commercial sources. The final approach used the cluster and visualization platform to group alkene subclasses together to create new substrate structures for alkene tests. Structures were either taken directly from the chemical space, modified to be synthetically tractable, or designed on the basis of key representations or hypotheses of predicted performance of the model. Although the approach necessarily lowers the amount of chemical space covered, the more subjective nature of the approach enables flexibility in the selection of alkenes to directly stress-test the model. For example, alkene **2** (1-allyl-4-*tert*-butylbenzene) was chosen to test if the Mono model could overcome the high representation of styrene-derived structures in the database to recognize the lower enantioselectivity expected when the alkene is homologated by a single carbon. The experimental validation consisted of 15 alkenes, with different examples for each alkene class. The selection is not intended to be comprehensive of the entire catalog of osmium-catalyzed

dihydroxylations, rather it is meant to provide insight into the performance of the models. The Tetra alkene class was limited to a single alkene due to poor conversion preventing adequate enantioselectivity measurements. All alkenes chosen for experimental validation have never been represented in the database or the literature in an enantioselective fashion at the time of this work.

Model Parameters

A evaluation platform was developed using version 1.5.2 of scikit-learn.[49] that screened a variety of scalers, correlated feature removal, feature selection methods, and modeling architectures. No additional dimensionality reduction methods were evaulated in initial preprocessing to maintain the interpretability of the modeling. Modeling attempts included PLS Regression,[50] Support Vector Regression (SVR),[51] Ridge,[52] Lasso,[53] Random Forest (RF) Regression,[54] Gradient Boosting Regression (GBR),[55] XGBoost Regression,[56] *k*-neighbors regression,[57] and Gaussian process regression (GPR).[58] Models were constructed with a train/test split of 80/20 with parameters optimized using a 5-fold cross-validation randomized search. These results were then extended using a 5-fold cross-validation Bayesian optimization of model hyperparameters using version 0.10.2 of scikit-optimize.[59] The evaluation platform also attempted to create models associated with both Max Volume alignment and 3-BFS Volume alignment, as it was unclear if the volume type would have any impact on modeling. The results of all model evaluations were compiled, and the architecture with the most successful model and subset of features was then optimized further. The preprocessing and modeling steps used are compiled in Table S3.

Alkene Type	Alignment Method	Scaling Method	Threshold for Correlation Removal	Feature Selection Method	Model Type
Mono	MaxVol	***MinMaxScaler***	0.806	***SelectPercentile***<br>• percentile=14<br>• score_func=mutual_info_regression	***GBR***<br>• learning_rate=0.77<br>• loss=absolute_error<br>• max_depth=2<br>• n_estimators=53<br>• subsample=0.91
Gem	BFSVol	***StandardScaler***	0.809	***SelectPercentile***<br>• percentile=55<br>• score_func=f_regression	***GBR***<br>• learning_rate=0.61<br>• max_depth=1<br>• n_estimators=49<br>• subsample=0.71
Cis	BFSVol	***StandardScaler***	1.00	***SelectKBest***<br>• k=5<br>• score_func=f_regression	***GBR***<br>• learning_rate=0.71<br>• max_depth=1<br>• n_estimators=31<br>• subsample=0.71
Trans	MaxVol	***MinMaxScaler***	0.940	***SelectKBest***<br>• k=32<br>• score_func=f_regression	***RF***<br>• ccp_alpha=1e-05<br>• criterion='poisson'<br>• max_depth=10,<br>• n_estimators=199
TriQ2	BFSVol	***StandardScaler***	0.950	***SelectKBest***<br>• k=37<br>• score_func=f_regression	***GBR***<br>• learning_rate=0.51<br>• max_depth=2<br>• n_estimators=18<br>• subsample=0.66
TriQ3	MaxVol	***RobustScaler***	0.75	***SelectKBest***<br>• k=6<br>• score_func=mutual_info_regression	***GBR***<br>• learning_rate=0.21<br>• loss=absolute_error<br>• max_depth=1<br>• n_estimators=39<br>• subsample=0.86
TriQ4	MaxVol	***StandardScaler***	None	***SelectKBest***<br>• k=12<br>• score_func=mutual_info_regression	***GBR***<br>• learning_rate=0.46<br>• max_depth=2<br>• n_estimators=24<br>• subsample=0.56
Tetra	MaxVol	***RobustScaler***	0.75	***SelectPercentile***<br>• percentile=18<br>• score_func=f_regression	**GP**<br>• alpha=0.1

Table S3. Breakdown of the alkene models, alignment schemes, and final pipelines used for the final models. GBR represents gradient boosting regression, RF represents random forest regression, and GP represents gaussian process regression.

The models shown in the main text are shown as a parity plot with both the training and test sets combined. They have been split apart to make results clearer in Figure S21 and Figure S22

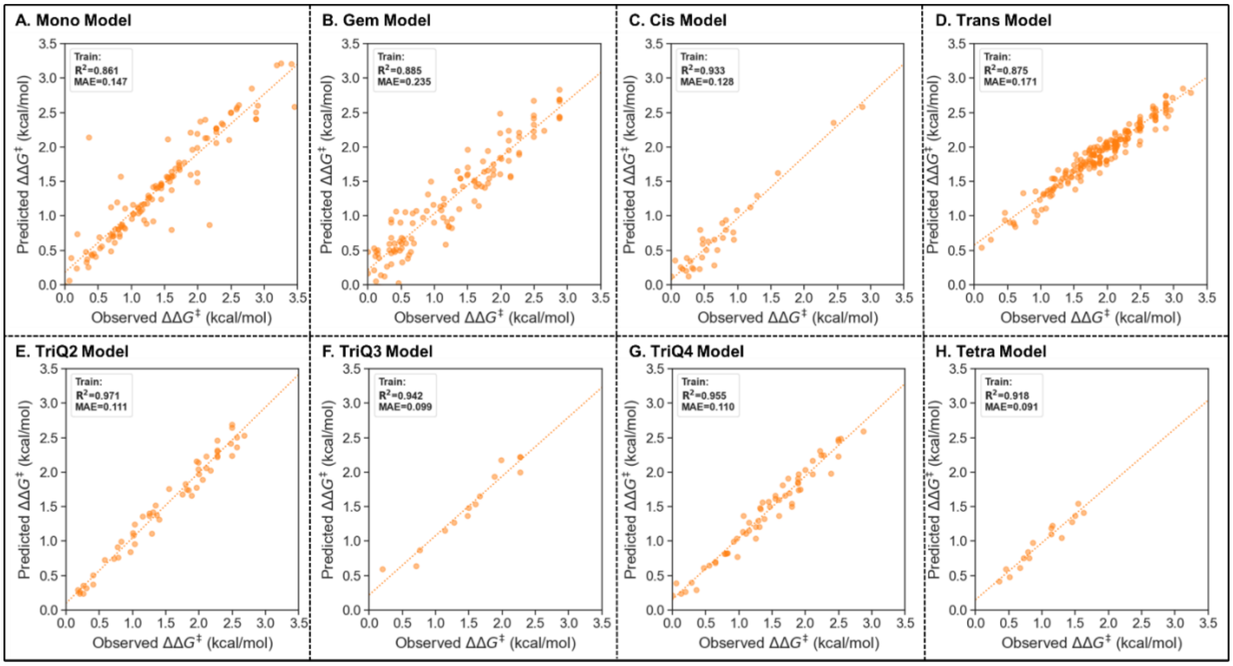

Figure S21 Modeling results for the training set of each alkene classes (**A**) Mono (**B**) Gem (**C**) Cis (**D**) Trans (**E**) TriQ2 (**F**) TriQ3 (**G**) TriQ4 (**H**) Tetra.

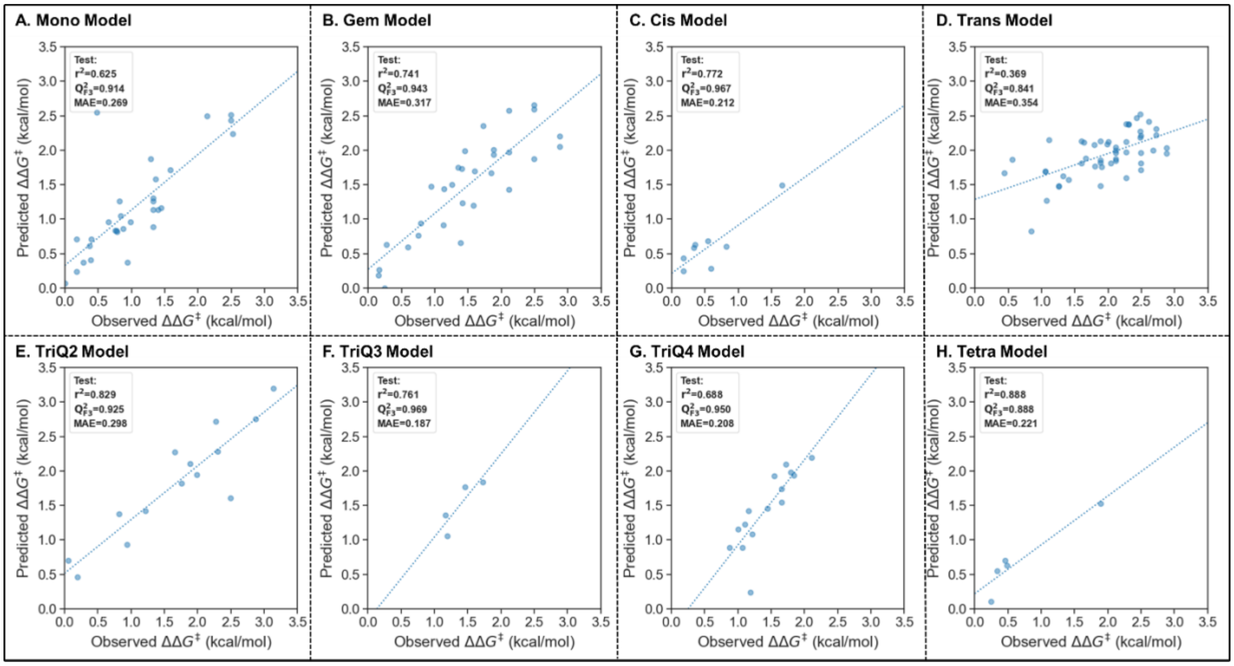

Figure S22 Modeling results for the test set of each alkene classes (**A**) Mono (**B**) Gem (**C**) Cis (**D**) Trans (**E**) TriQ2 (**F**) TriQ3 (**G**) TriQ4 (**H**) Tetra.

The results for the prediction of the experimental validation are also shown with respect to $\Delta\Delta G^{\ddagger}$ in Figure S23.

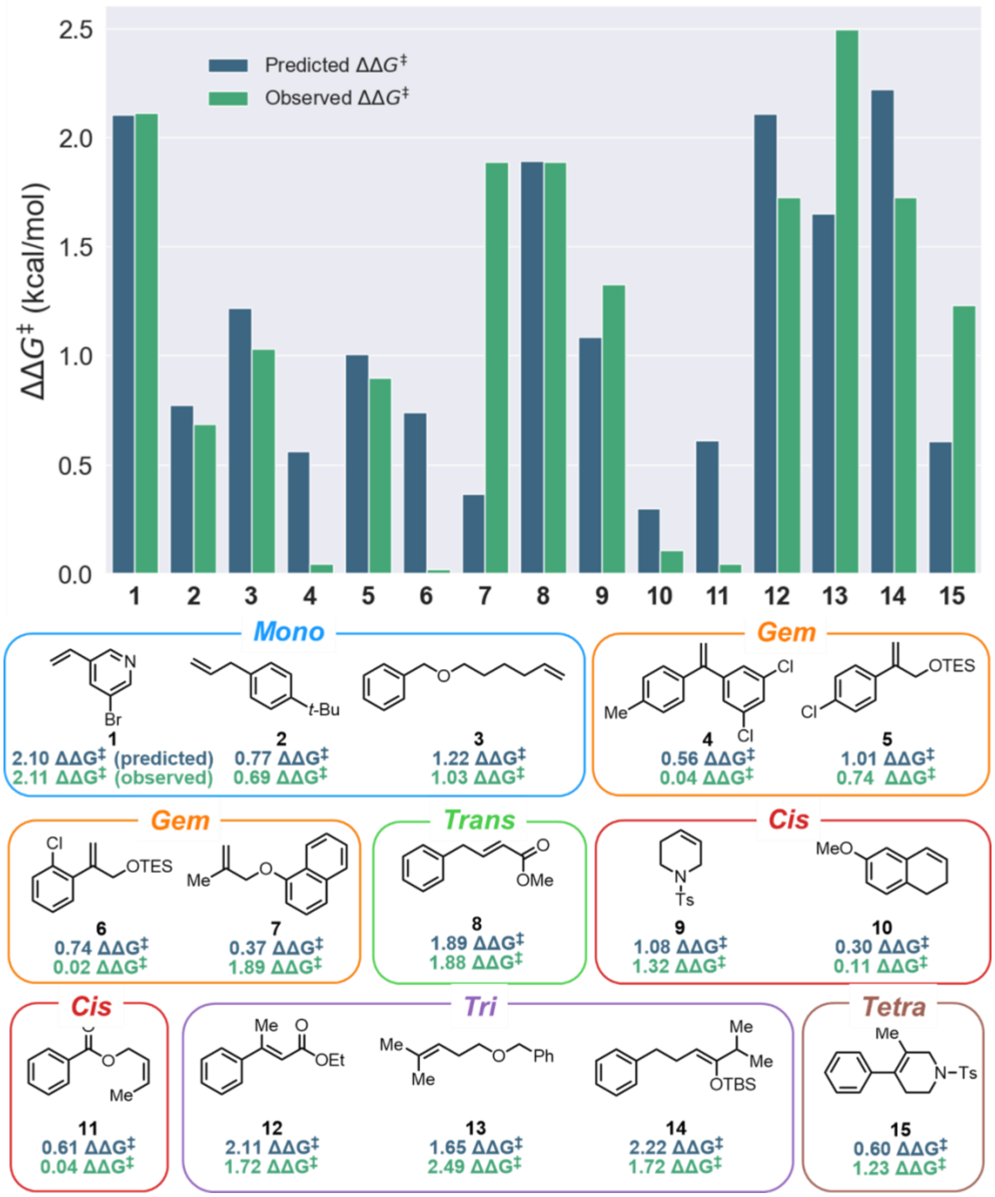

Figure S23 Experimental Validation results given in ΔΔG‡ for all alkene models with predicted shown on top (dark blue) and observed shown below (green). Statistical Analysis

Compiled Metrics

The metrics used to evaluate this work were train R^2, train mean absolute error (MAE), Q^2_{F3}, test r^2, and test mean absolute error (MAE). In this context, R^2 is not to be confused with r^2. Despite their often interchangeable use, R^2 refers to the coefficient of determination, and r^2 refers to the squared correlation coefficient.[60] These metrics are often close but not necessarily the same since they are not calculated in the same manner. The distinction of R^2 vs. r^2 is important to recognize, as this work utilizes non-linear modeling architecture. The r^2 does not necessarily

provide an accurate description of the true fit of the model. The problem with traditional test R^2 value as a metric is that it is only representative of a single split, and the test R^2 value is dependent on the distribution of the data in the test split. Quantitative structure activity relationship (QSAR) modeling has uncovered that Q^2_{F3} is a more robust assessment for quantitative performance analysis owing to its independence of external object distribution when compared to the tradition test R^2, or Q^2_{F2}, analysis.[61, 62] The test r^2 was included as it is a more familiar metric. The compiled metrics for all models are shown in Table S4. Baseline MAE refers to the MAE if only the average value of the training data is used to predict the train data.

Alkene Type	Model Type	Train R^2	Train MAE (kcal/mol)	Test r^2	Test Q^2_{F3}	Test MAE (kcal/mol)	Baseline MAE (kcal/mol)
Mono	GBR	0.86	0.15	0.63	0.91	0.27	0.64
Gem	GBR	0.89	0.24	0.74	0.94	0.32	0.72
Cis	GBR	0.93	0.13	0.77	0.97	0.22	0.43
Trans	RF	0.88	0.17	0.37	0.84	0.35	0.47
TriQ2	GBR	0.97	0.11	0.83	0.93	0.3	0.67
TriQ3	GBR	0.94	0.1	0.76	0.97	0.19	0.49
TriQ4	GBR	0.96	0.11	0.69	0.95	0.21	0.56
Tetra	GP	0.92	0.1	0.89	0.89	0.22	0.35

Table S4. Breakdown of the alkene models and combined metrics for train and test.

Model Validation Tests

The first control test conducted was a one-hot encoding test, which replaces the real feature matrix with a one-hot encoding matrix. The results are shown in Figure S24. Although some models show what appears to be reasonable metrics for training, when the predictions are visualized, the models clearly demonstrate poor performance, only successfully training on a few points. Most points are assigned an average prediction for the dataset. The failure in prediction extends to the test set, with no meaningful regression occurring. All eight alkene models failed strongly with one-hot encoding, confirming that the model is not solely memorizing the index of the alkenes.

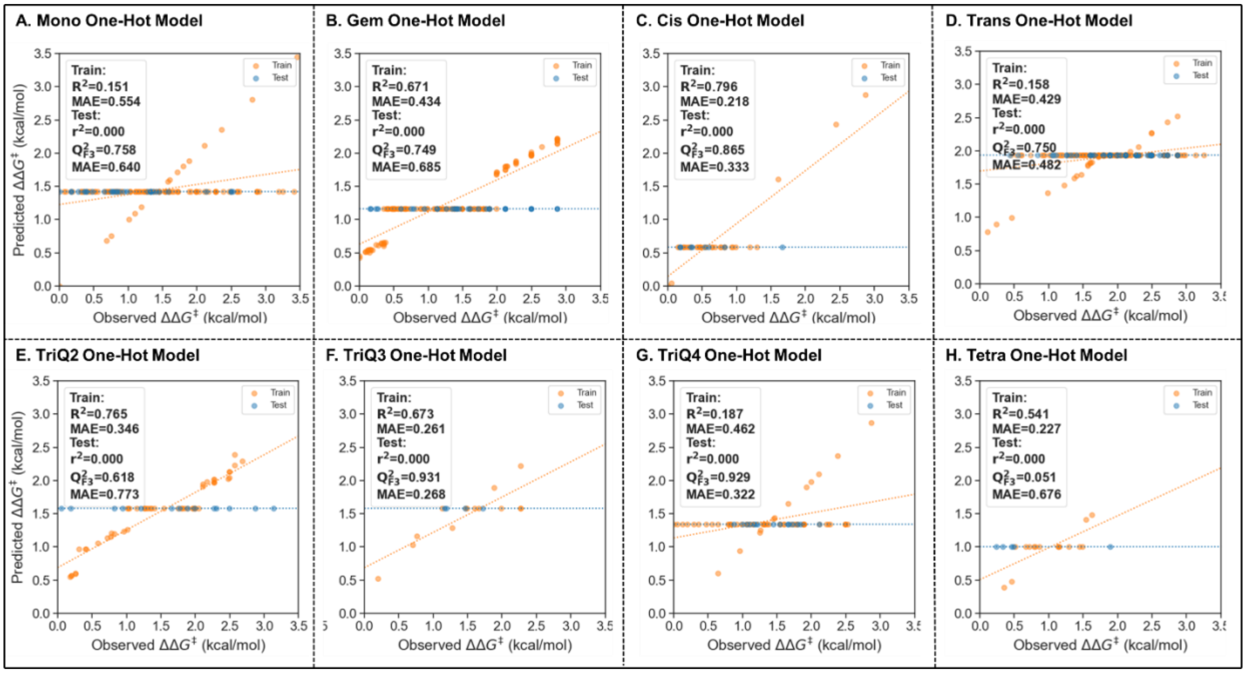

Figure S24. The results for each model with one-hot encoding of the alkenes. Orange represents the training set, while blue represents the test set. (**A**) Mono (**B**) Gem (**C**) Cis (**D**) Trans (**E**) TriQ2 (**F**) TriQ3 (**G**) TriQ4 (**H**) Tetra.

An additional control test was training with random features and attempting to predict the test set. The results of this are shown in Figure S25. The models have a clear propensity to fit strongly to the training set, reaching an average train R^2 0.90 and train MAE of 0.133 kcal/mol. However, the good performance does not carry forward to performance on the test set, reaching an average test r^2 of 0.09, average Q^2_{F3} of 0.46, and test MAE of 0.666 kcal/mol. The poor performance of the test set confirms that the current feature space provides more than just randomly assigning features.

It is important to note that despite poor regression on all alkene classes, the TriQ3 model shows reasonable predictive power with respect to the mean absolute error, achieving an MAE of 0.188 kcal/mol. This result is on par with the performance of the model with real features in which the MAE was 0.187 kcal/mol, suggesting that the current TriQ3 model is most likely overfit and was not an adequate test of the predictive power of the model in initial screening. The features also cannot be confirmed to have any meaning beyond spurious correlation. This was not unexpected, as the TriQ3 class only contains 18 alkenes for train/test splitting, which severely limits the ability to train a strong model.

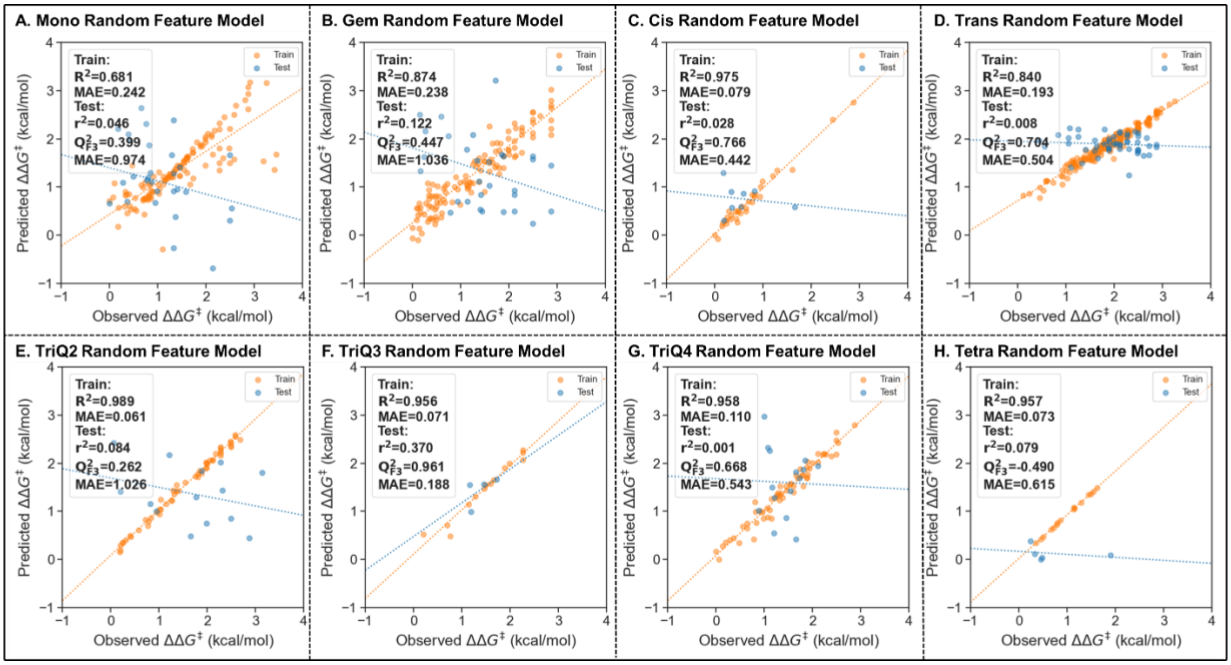

Figure S25. The results for each model with one-hot encoding of the alkenes. Orange represents the training set, while blue represents the test set. (**A**) Mono (**B**) Gem (**C**) Cis (**D**) Trans (**E**) TriQ2 (**F**) TriQ3 (**G**) TriQ4 (**H**) Tetra.

The next validation test conducted was Y-randomization. The goal of this technique is not to estimate errors of prediction, but rather estimate the reliability of the metrics through statistical significance.[63, 64] Y-randomization, also known as Y-scrambling, takes the y values (i.e. $\Delta\Delta G^{\ddagger}$), and shuffles them randomly. The shuffled y values are then regressed on with the same feature array. Statistical significance is then quantified via a Z-test, which estimates if the metrics of the model falls in the tail end of the distribution of metrics.[65] The equation for a Z-test is shown below in Scheme S2.

$$Z = \frac{metric - \mu}{\sigma}$$

Scheme S2. Equation for calculation of the Z score.

In this equation, *metric* refers to the error of the original model, μ refers to the average value of the metric estimates of the y-shuffled models, and σ refers to the standard deviation of the error estimates for all permutations. A p value can then be calculated using a normal distribution, and if the p value is <0.05, the estimated error is statistically significant. The y values were shuffled 100 times and retrained with the same train/test split as the original models. The test MAE and test

r^2 were tested and the results are shown in Table S5. The calculated Z score must have an absolute value of 1.645 to be considered statistically significant. The MAE has a negative Z score because lower values are considered better. For all models, the test MAE converged on is statistically significant. All models except TriQ3 also show statistical significance with respect to the test r^2 value. The p value is <0.08, suggesting that if the performance of the model was statistically equivalent to the random model, there is still an 8% chance one could observe a deviation in r^2 performance this large. Given the good performance of random features in modeling the test set, it is unlikely the TriQ3 model can be claimed as more than random chance. As discussed in the random feature models, TriQ3 may show this poor result because of the smaller number of values available to train (i.e. only 18 alkenes).

Alkene Type	Test MAE Z Score	Test MAE p value	Statistically Significant	Test r^2 Z Score	Test r^2 p value	Statistically Signifcant
Mono	-5.255	<0.01	Yes	11.487	<0.01	Yes
Gem	-5.204	<0.01	Yes	18.398	<0.01	Yes
Cis	-1.935	0.0265	Yes	4.031	<0.01	Yes
Trans	-2.748	<0.01	Yes	10.980	<0.01	Yes
TriQ2	-3.670	<0.01	Yes	9.144	<0.01	Yes
TriQ3	-1.974	0.0242	Yes	1.413	0.0788	No
TriQ4	-4.083	<0.01	Yes	7.217	<0.01	Yes
Tetra	-1.894	0.0291	Yes	2.699	<0.01	Yes

Table S5. Z scores and p values for all alkene models.

An additional validation test conducted was q^2 testing, focusing on the internal validation of the model. The q^2 metric is not to be confused with Q^2_{F3} metric, which is specifically a metric of the external test set. The q^2 value represents the cross-validated coefficient of determination.[66] This metric attempts to quantify the stability and predictive power of the model, and its equation is shown in Scheme S3.

$$q^2 = 1 - \frac{PRESS}{TSS}$$

Scheme S3. Equation representing the q^2 metric for cross-validation.

The *PRESS* refers to the predicted residual sum of squares, and the *TSS* refers to the total sum of squares. Cross-validation involves splitting the training set into separate partitions called

folds that consist of a smaller training set and a validation set. The model is retrained on the training set, the validation set is predicted, and this process is repeated over all the folds until all the original training set has been tested in the validation set. The sum of all validation set residuals is summed and used to calculate the final q^2. The cross-validation metrics for all available data are shown with 3-fold, 5-fold, 10-fold, and leave-one-out (LOO) cross-validation in Table S6.

**Alkene Type**	$q^2_{3\text{-fold}}$	$q^2_{5\text{-fold}}$	$q^2_{10\text{-fold}}$	q^2_{LOO}
Mono	0.41	0.49	0.55	0.52
Gem	0.40	0.44	0.40	0.52
Cis	-1.14	0.23	-0.17	-0.64
Trans	0.20	0.25	0.22	0.23
TriQ2	0.27	-0.06	0.19	0.14
TriQ3	-0.11	-0.10	0.01	0.29
TriQ4	-0.29	0.21	0.15	0.02
Tetra	-0.38	-0.15	0.01	0.02

Table S6. Cross-validation q^2 metrics with various folds for all alkene models.

For a model to be considered predictive and stable, the general goal is for the q^2 to be greater than the 0.5.[66] Because of the small data set on which the models were derived, it would be expected that the largest number of alkenes would be necessary for training, making the LOO metric the most representative category of q^2 for this work. The only models that reach strong q^2 metrics are the Mono and Gem models. This result would suggest the models would be expected to have poor predictive ability. However, the q^2 metric is fundamentally a test of internal validation, and as such, there exists no correlation between LOO cross-validated q^2 and metrics for an external test set.[67] In fact, a high q^2 metric does not necessarily indicate proof that a model has high predictive ability, rather, it is a way of evaluating the existing architecture for predicted extrapolative power and potential for overfitting.[68] The true test to establish a reliable QSAR model is through external validation. All the current alkene models showed statistically significant predictions and meaningful correlations associated from the feature analysis with varying substructures. The poor performance in cross-validation suggests the presence of some overfitting and instability in the models, as well as potential limitations in the extrapolative power of the model.

References

[1] Jin, Y.; Jing, Y.; Li, C.; Li, M.; Wu, W.; Ke, Z.; Jiang, H. Palladium-Catalysed Selective Oxidative Amination of Olefins with Lewis Basic Amines. *Nat. Chem.*, **2022**, *14* (10), 1118–1125. https://doi.org/10.1038/s41557-022-01023-x.

[2] Li, X.; He, S.; Song, Q. Diethylzinc-Mediated Radical 1,2-Addition of Alkenes and Alkynes. *Org. Lett.*, **2021**, *23* (8), 2994–2999. https://doi.org/10.1021/acs.orglett.1c00669.

[3] Kongkathip, N.; Kongkathip, B.; Siripong, P.; Sangma, C.; Luangkamin, S.; Niyomdecha, M.; Pattanapa, S.; Piyaviriyagul, S.; Kongsaeree, P. Potent Antitumor Activity of Synthetic 1,2-Naphthoquinones and 1,4-Naphthoquinones. *Bioorg. Med. Chem.*, **2003**, *11* (14), 3179–3191. https://doi.org/10.1016/S0968-0896(03)00226-8.

[4] Sato, S.; Tetsuhashi, M.; Sekine, K.; Miyachi, H.; Naito, M.; Hashimoto, Y.; Aoyama, H. Degradation-Promoters of Cellular Inhibitor of Apoptosis Protein 1 Based on Bestatin and Actinonin. *Bioorg. Med. Chem.*, **2008**, *16* (8), 4685–4698. https://doi.org/10.1016/j.bmc.2008.02.024.

[5] Nguyen, J. A.; Cabanero, D. C.; Rovis, T. Electrochemically Controlled Ruthenium-Catalyzed Olefin -Metathesis. *Synlett*, **2023**, *34* (12), 1477–1481. https://doi.org/10.1055/s-0042-1751469.

[6] Nadeau, E.; Ventura, D. L.; Brekan, J. A.; Davies, H. M. L. Controlling Factors for C−H Functionalization versus Cyclopropanation of Dihydronaphthalenes. *J. Org. Chem.*, **2010**, *75* (6), 1927–1939. https://doi.org/10.1021/jo902644f.

[7] Lonardi, G.; Franco, S.; Sartorello, M.; De Faveri, C.; Stivanello, M.; Licini, G.; Orlandi, M. Enantioselective Synthesis of Cyclopropanes via CuH-Catalyzed Intramolecular Hydroalkylation. *ACS Catal.*, **2024**, *14* (11), 8730–8738. https://doi.org/10.1021/acscatal.4c02119.

[8] Connolly, T.; Wang, Z.; Walker, M. A.; McDonald, I. M.; Peese, K. M. Tandem Ring-Closing Metathesis/Transfer Hydrogenation: Practical Chemoselective Hydrogenation of Alkenes. *Org. Lett.*, **2014**, *16* (17), 4444–4447. https://doi.org/10.1021/ol5019739.

[9] Sun, X.; Frimpong, K.; Tan, K. L. Synthesis of Quaternary Carbon Centers via Hydroformylation. *J. Am. Chem. Soc.*, **2010**, *132* (34), 11841–11843. https://doi.org/10.1021/ja1036226.

[10] Kawato, Y.; Kubota, A.; Ono, H.; Egami, H.; Hamashima, Y. Enantioselective Bromocyclization of Allylic Amides Catalyzed by BINAP Derivatives. *Org. Lett.*, **2015**, *17* (5), 1244–1247. https://doi.org/10.1021/acs.orglett.5b00220.

[11] Zhuo, J.; Zhang, Y.; Li, Z.; Li, C. Nickel-Catalyzed Direct Acylation of Aryl and Alkyl Bromides with Acylimidazoles. *ACS Catal.*, **2020**, *10* (6), 3895–3903. https://doi.org/10.1021/acscatal.0c00246.

[12] Greb, A.; Poh, J.; Greed, S.; Battilocchio, C.; Pasau, P.; Blakemore, D. C.; Ley, S. V. A Versatile Route to Unstable Diazo Compounds via Oxadiazolines and Their Use in Aryl–Alkyl Cross-Coupling Reactions. *Angew. Chem. Int. Ed.*, **2017**, *56* (52), 16602–16605. https://doi.org/10.1002/anie.201710445.

[13] Beligny, S.; Eibauer, S.; Maechling, S.; Blechert, S. Sequential Catalysis: A Metathesis/Dihydroxylation Sequence. *Angew. Chem. Int. Ed.*, **2006**, *45* (12), 1900–1903. https://doi.org/10.1002/anie.200503552.

[14] Kolb, H. C.; VanNieuwenhze, M. S.; Sharpless, K. B. Catalytic Asymmetric Dihydroxylation. *Chem. Rev.*, **1994**, *94* (8), 2483–2547. https://doi.org/10.1021/cr00032a009.

[15] Shved, A. S.; Ocampo, B. E.; Burlova, E. S.; Olen, C. L.; Rinehart, N. I.; Denmark, S. E. Molli: A General Purpose Python Toolkit for Combinatorial Small Molecule Library Generation, Manipulation, and Feature Extraction. *J. Chem. Inf. Model.*, **2024**. https://doi.org/10.1021/acs.jcim.4c00424.

[16] Landrum, G. RDKit: Open-Source Cheminformatics Software, 2023.

[17] Weininger, D. SMILES, a Chemical Language and Information System. 1. Introduction to Methodology and Encoding Rules. *J. Chem. Inf. Comput. Sci.*, **1988**, *28* (1), 31–36. https://doi.org/10.1021/ci00057a005.

[18] Wang, Z.-M.; Sharpless, K. B. Asymmetric Dihydroxylation of α-Substituted Styrene Derivatives. *Synlett*, **1993**, *1993* (08), 603–604. https://doi.org/10.1055/s-1993-22547.

[19] Daylight Theory: SMARTS - A Language for Describing Molecular Patterns https://www.daylight.com/dayhtml/doc/theory/theory.smarts.html (accessed Apr 10, 2023).

[20] Halgren, T. A. Merck Molecular Force Field. I. Basis, Form, Scope, Parameterization, and Performance of MMFF94. *J. Comput. Chem.*, **1996**, *17* (5–6), 490–519. https://doi.org/10.1002/(SICI)1096-987X(199604)17:5/6<490::AID-JCC1>3.0.CO;2-P.

[21] Neese, F.; Wennmohs, F.; Becker, U.; Riplinger, C. The ORCA Quantum Chemistry Program Package. *J. Chem. Phys.*, **2020**, *152* (22), 224108. https://doi.org/10.1063/5.0004608.

[22] Glendening, E. D.; Landis, C. R.; Weinhold, F. *NBO 7.0*: New Vistas in Localized and Delocalized Chemical Bonding Theory. *J. Comput. Chem.*, **2019**, *40* (25), 2234–2241. https://doi.org/10.1002/jcc.25873.

[23] Becke, A. D. Density-Functional Thermochemistry. III. The Role of Exact Exchange. *J. Chem. Phys.*, **1993**, *98*, 5648–5652. https://doi.org/10.1063/1.464913.

[24] Lee, C.; Yang, W.; Parr, R. G. Development of the Colle-Salvetti Correlation-Energy Formula into a Functional of the Electron Density. *Phys. Rev. B*, **1988**, *37* (2), 785–789. https://doi.org/10.1103/PhysRevB.37.785.

[25] Weigend, F.; Ahlrichs, R. Balanced Basis Sets of Split Valence, Triple Zeta Valence and Quadruple Zeta Valence Quality for H to Rn: Design and Assessment of Accuracy. *Phys. Chem. Chem. Phys.*, **2005**, *7* (18), 3297–3305. https://doi.org/10.1039/B508541A.

[26] Jacot-Descombes, L.; Turcani, L.; Jorner, K. Morfeus, 2024.

[27] Verloop, A.; Hoogenstraaten, W.; Tipker, J. Development and Application of New Steric Substituent Parameters in Drug Design. In *Drug Design*; Elsevier, 1976; pp 165–207. https://doi.org/10.1016/B978-0-12-060307-7.50010-9.

[28] Harper, K. C.; Bess, E. N.; Sigman, M. S. Multidimensional Steric Parameters in the Analysis of Asymmetric Catalytic Reactions. *Nat. Chem.*, **2012**, *4* (5), 366–374. https://doi.org/10.1038/nchem.1297.

[29] Brethomé, A. V.; Fletcher, S. P.; Paton, R. S. Conformational Effects on Physical-Organic Descriptors: The Case of Sterimol Steric Parameters. *ACS Catal.*, **2019**, *9* (3), 2313–2323. https://doi.org/10.1021/acscatal.8b04043.

[30] Zhao, Y. H.; Abraham, M. H.; Zissimos, A. M. Fast Calculation of van Der Waals Volume as a Sum of Atomic and Bond Contributions and Its Application to Drug Compounds. *J. Org. Chem.*, **2003**, *68* (19), 7368–7373. https://doi.org/10.1021/jo034808o.

[31] Mantina, M.; Chamberlin, A. C.; Valero, R.; Cramer, C. J.; Truhlar, D. G. Consistent van Der Waals Radii for the Whole Main Group. *J. Phys. Chem. A*, **2009**, *113* (19), 5806–5812. https://doi.org/10.1021/jp8111556.

[32] Zahrt, A. F.; Henle, J. J.; Rose, B. T.; Wang, Y.; Darrow, W. T.; Denmark, S. E. Prediction of Higher-Selectivity Catalysts by Computer-Driven Workflow and Machine Learning. *Science*, **2019**, *363* (6424). https://doi.org/10.1126/science.aau5631.

[33] Bannwarth, C.; Ehlert, S.; Grimme, S. GFN2-xTB—An Accurate and Broadly Parametrized Self-Consistent Tight-Binding Quantum Chemical Method with Multipole Electrostatics and Density-Dependent Dispersion Contributions. *J. Chem. Theory Comput.*, **2019**, *15* (3), 1652–1671. https://doi.org/10.1021/acs.jctc.8b01176.

[34] Pracht, P.; Bohle, F.; Grimme, S. Automated Exploration of the Low-Energy Chemical Space with Fast Quantum Chemical Methods. *Phys. Chem. Chem. Phys.*, **2020**, *22* (14), 7169–7192. https://doi.org/10.1039/C9CP06869D.

[35] Aprà, E.; Bylaska, E. J.; de Jong, W. A.; Govind, N.; Kowalski, K.; Straatsma, T. P.; Valiev, M.; van Dam, H. J. J.; Alexeev, Y.; Anchell, J.; et al. NWChem: Past, Present, and Future. *J. Chem. Phys.*, **2020**, *152* (18), 184102. https://doi.org/10.1063/5.0004997.

[36] Grimme, S.; Antony, J.; Ehrlich, S.; Krieg, H. A Consistent and Accurate *Ab Initio* Parametrization of Density Functional Dispersion Correction (DFT-D) for the 94 Elements H-Pu. *J. Chem. Phys.*, **2010**, *132* (15), 154104. https://doi.org/10.1063/1.3382344.

[37] Li, Y.; Evans, J. N. S. The Fukui Function: A Key Concept Linking Frontier Molecular Orbital Theory and the Hard-Soft-Acid-Base Principle. *J. Am. Chem. Soc.*, **1995**, *117* (29), 7756–7759. https://doi.org/10.1021/ja00134a021.

[38] Wiberg, K. B. Application of the Pople-Santry-Segal CNDO Method to the Cyclopropylcarbinyl and Cyclobutyl Cation and to Bicyclobutane. *Tetrahedron*, **1968**, *24* (3), 1083–1096. https://doi.org/10.1016/0040-4020(68)88057-3.

[39] Caldeweyher, E.; Bannwarth, C.; Grimme, S. Extension of the D3 Dispersion Coefficient Model. *J. Chem. Phys.*, **2017**, *147* (3), 034112. https://doi.org/10.1063/1.4993215.

[40] Tsuzuki, S.; Uchimaru, T. Accuracy of Intermolecular Interaction Energies, Particularly Those of Hetero-Atom Containing Molecules Obtained by DFT Calculations with Grimme's D2, D3 and D3BJ Dispersion Corrections. *Phys. Chem. Chem. Phys.*, **2020**, *22* (39), 22508–22519. https://doi.org/10.1039/D0CP03679J.

[41] Hagberg, A. A.; Schult, D. A.; Swart, P. J. Exploring Network Structure, Dynamics, and Function Using NetworkX. In *Proceedings of the 7th Python in Science Conference*; Varoquaux, G., Vaught, T., Millman, J., Eds.; Pasadena, CA USA, 2008; pp 11–15.

[42] Syms, C. Principal Components Analysis. In *Encyclopedia of Ecology*; Jørgensen, S. E., Fath, B. D., Eds.; Academic Press: Oxford, 2008; pp 2940–2949. https://doi.org/10.1016/B978-008045405-4.00538-3.

[43] Axen, S. D.; Huang, X.-P.; Cáceres, E. L.; Gendelev, L.; Roth, B. L.; Keiser, M. J. A Simple Representation of Three-Dimensional Molecular Structure. *J. Med. Chem.*, **2017**, *60* (17), 7393–7409. https://doi.org/10.1021/acs.jmedchem.7b00696.

[44] Molecular Modeling Software | OpenEye Scientific https://www.eyesopen.com (accessed Mar 19, 2025).

[45] Morgan, H. L. The Generation of a Unique Machine Description for Chemical Structures-A Technique Developed at Chemical Abstracts Service. *J. Chem. Doc.*, **1965**, *5* (2), 107–113. https://doi.org/10.1021/c160017a018.

[46] Bajusz, D.; Rácz, A.; Héberger, K. Why Is Tanimoto Index an Appropriate Choice for Fingerprint-Based Similarity Calculations? *J. Cheminformatics*, **2015**, *7* (1). https://doi.org/10.1186/s13321-015-0069-3.

[47] Jin, X.; Han, J. K-Means Clustering. In *Encyclopedia of Machine Learning*; Sammut, C., Webb, G. I., Eds.; Springer US: Boston, MA, 2010; pp 563–564. https://doi.org/10.1007/978-0-387-30164-8_425.

[48] Satopää, V.; Albrecht, J.; Irwin, D.; Raghavan, B. Finding a "Kneedle" in a Haystack. *IEEE ICDCS SIMPLEX Workshop*, **2011**.

[49] Pedregosa, F.; Varoquaux, G.; Gramfort, A.; Michel, V.; Thirion, B.; Grisel, O.; Blondel, M.; Prettenhofer, P.; Weiss, R.; Dubourg, V.; et al. Scikit-Learn: Machine Learning in Python. *J. Mach. Learn. Res.*, **2011**, *12* (85), 2825–2830.

[50] Wold, S.; Sjöström, M.; Eriksson, L. PLS-Regression: A Basic Tool of Chemometrics. *Chemom. Intell. Lab. Syst.*, **2001**, *58* (2), 109–130. https://doi.org/10.1016/S0169-7439(01)00155-1.

[51] Drucker, H.; Burges, C. J. C.; Kaufman, L.; Smola, A.; Vapnik, V. Support Vector Regression Machines. In *Advances in Neural Information Processing Systems*; Mozer, M. C., Jordan, M., Petsche, T., Eds.; MIT Press, 1996; Vol. 9.

[52] Hoerl, A. E.; Kennard, R. W. Ridge Regression: Biased Estimation for Nonorthogonal Problems. *Technometrics*, **1970**, *12* (1), 55–67. https://doi.org/10.1080/00401706.1970.10488634.

[53] Tibshirani, R. Regression Shrinkage and Selection via the Lasso. *J. R. Stat. Soc. Ser. B Methodol.*, **1996**, *58* (1), 267–288.

[54] Ho, T. K. Random Decision Forests. In *Proceedings of 3rd International Conference on Document Analysis and Recognition*; 1995; Vol. 1, pp 278–282 vol.1. https://doi.org/10.1109/ICDAR.1995.598994.

[55] Friedman, J. H. Greedy Function Approximation: A Gradient Boosting Machine. *Ann. Stat.*, **2001**, *29* (5), 1189–1232.

[56] Chen, T.; Guestrin, C. XGBoost: A Scalable Tree Boosting System. In *Proceedings of the 22nd ACM SIGKDD International Conference on Knowledge Discovery and Data Mining*; 2016; pp 785–794. https://doi.org/10.1145/2939672.2939785.

[57] Goldberger, J.; Hinton, G. E.; Roweis, S.; Salakhutdinov, R. R. Neighbourhood Components Analysis. In *Advances in Neural Information Processing Systems*; Saul, L., Weiss, Y., Bottou, L., Eds.; MIT Press, 2004; Vol. 17.

[58] Williams, C.; Rasmussen, C. Gaussian Processes for Regression. In *Advances in Neural Information Processing Systems*; MIT Press, 1995; Vol. 8.

[59] scikit-optimize: sequential model-based optimization in Python — scikit-optimize 0.8.1 documentation https://scikit-optimize.github.io/stable/ (accessed Mar 12, 2025).

[60] Kvålseth, T. O. Cautionary Note about R2. *Am. Stat.*, **1985**, *39* (4), 279–285. https://doi.org/10.2307/2683704.

[61] Consonni, V.; Ballabio, D.; Todeschini, R. Comments on the Definition of the Q2 Parameter for QSAR Validation. *J. Chem. Inf. Model.*, **2009**, *49* (7), 1669–1678. https://doi.org/10.1021/ci900115y.

[62] Todeschini, R.; Ballabio, D.; Grisoni, F. Beware of Unreliable Q2! A Comparative Study of Regression Metrics for Predictivity Assessment of QSAR Models. *J. Chem. Inf. Model.*, **2016**, *56* (10), 1905–1913. https://doi.org/10.1021/acs.jcim.6b00277.

[63] Klopman, G.; Kalos, A. N. Causality in Structure—Activity Studies. *J. Comput. Chem.*, **1985**, *6* (5), 492–506. https://doi.org/10.1002/jcc.540060520.

[64] *Applied Chemoinformatics: Achievements and Future Opportunities*; Engel, T., Gasteiger, J., Eds.; Wiley-VCH: Weinheim, 2018. https://doi.org/10.1002/9783527806539.

[65] Shen, M.; LeTiran, A.; Xiao, Y.; Golbraikh, A.; Kohn, H.; Tropsha, A. Quantitative Structure−Activity Relationship Analysis of Functionalized Amino Acid Anticonvulsant

Agents Using k Nearest Neighbor and Simulated Annealing PLS Methods. *J. Med. Chem.*, **2002**, *45* (13), 2811–2823. https://doi.org/10.1021/jm010488u.

[66] Tropsha, A.; Gramatica, P.; Gombar, V. K. The Importance of Being Earnest: Validation Is the Absolute Essential for Successful Application and Interpretation of QSPR Models. *QSAR Comb. Sci.*, **2003**, *22* (1), 69–77. https://doi.org/10.1002/qsar.200390007.

[67] Kubinyi, H.; Hamprecht, F. A.; Mietzner, T. Three-Dimensional Quantitative Similarity−Activity Relationships (3D QSiAR) from SEAL Similarity Matrices. *J. Med. Chem.*, **1998**, *41* (14), 2553–2564. https://doi.org/10.1021/jm970732a.

[68] Golbraikh, A.; Tropsha, A. Beware of Q2! *J. Mol. Graph. Model.*, **2002**, *20* (4), 269–276. https://doi.org/10.1016/s1093-3263(01)00123-1.

^{1}H, ^{13}C NMR Data and HPLC Traces

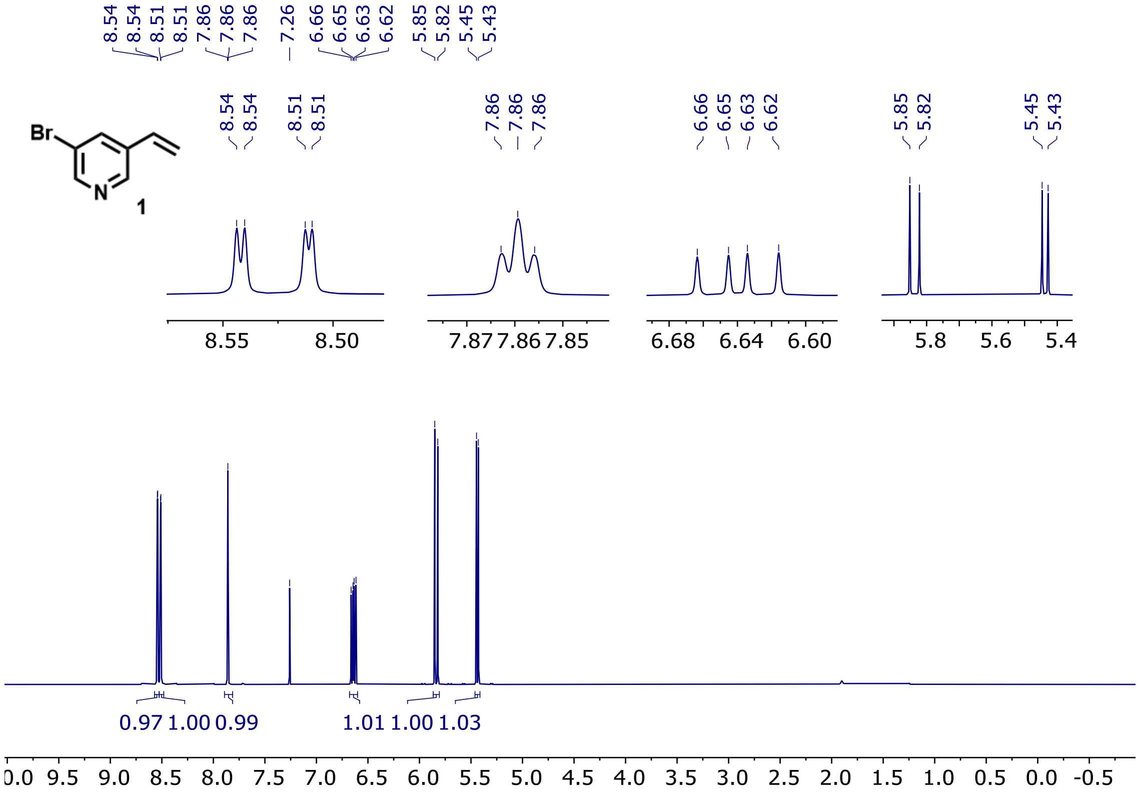

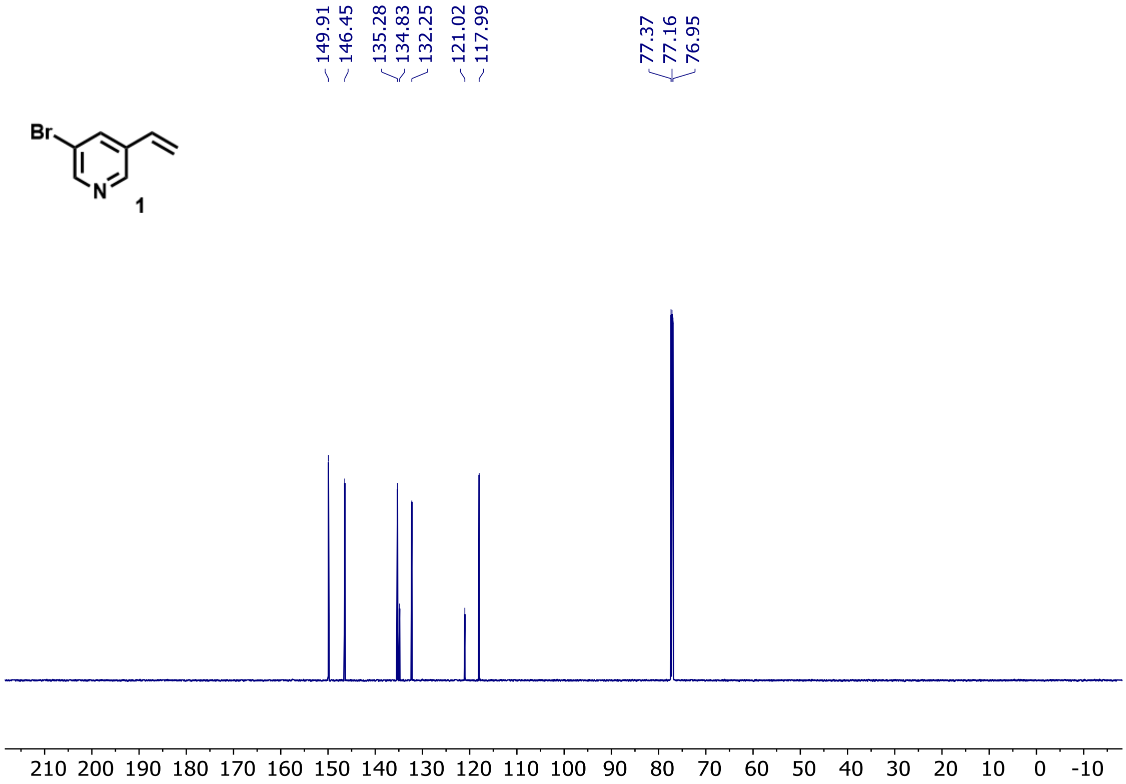
149.91
146.45
135.28
134.83
132.25
121.02
117.99
77.37
77.16
76.95
Br
N
1
210 200 190 180 170 160 150 140 130 120 110 100 90 80 70 60 50 40 30 20 10 0 -10

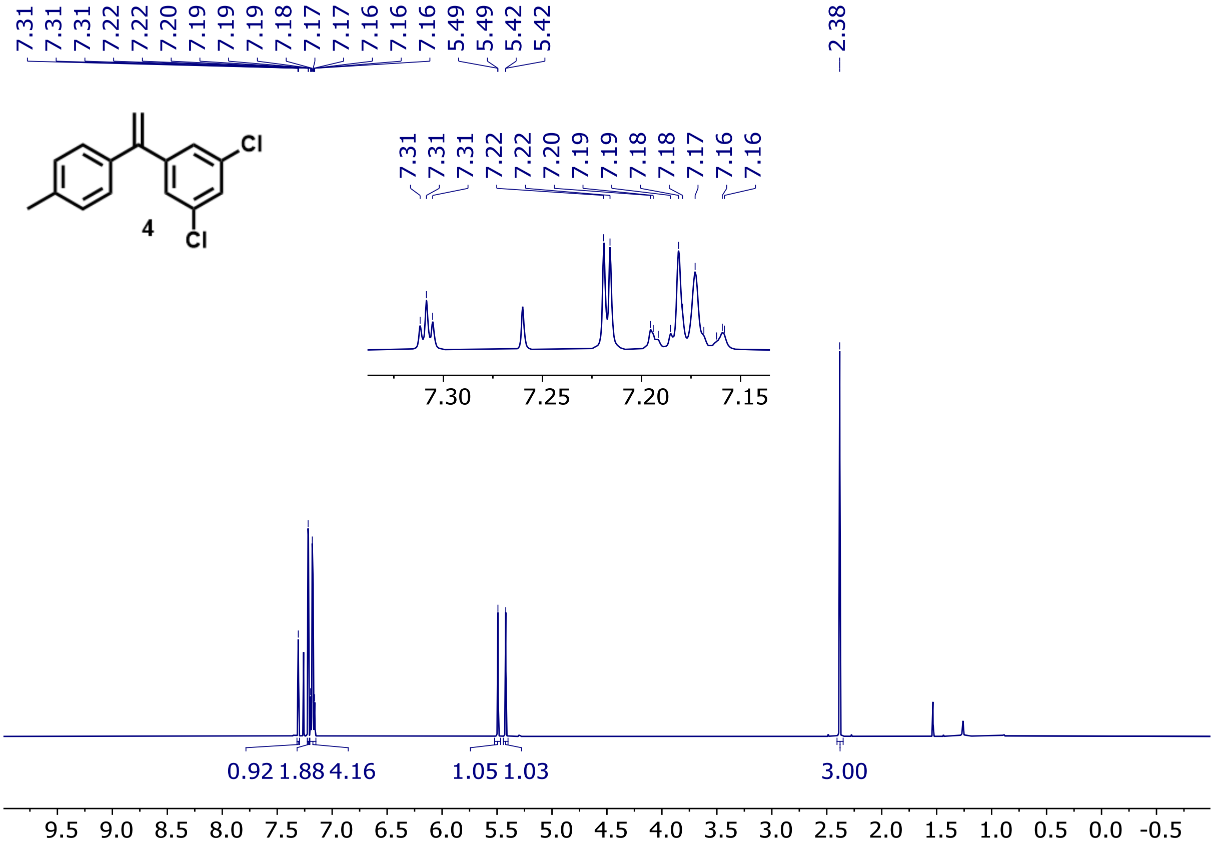
7.31
7.31
7.31
7.22
7.22
7.20
7.19
7.19
7.19
7.18
7.17
7.17
7.16
7.16
7.16
5.49
5.49
5.42
5.42
2.38
Cl
Cl
4
7.30 7.25 7.20 7.15
0.92 1.88 4.16
1.05 1.03
3.00
9.5 9.0 8.5 8.0 7.5 7.0 6.5 6.0 5.5 5.0 4.5 4.0 3.5 3.0 2.5 2.0 1.5 1.0 0.5 0.0 -0.5

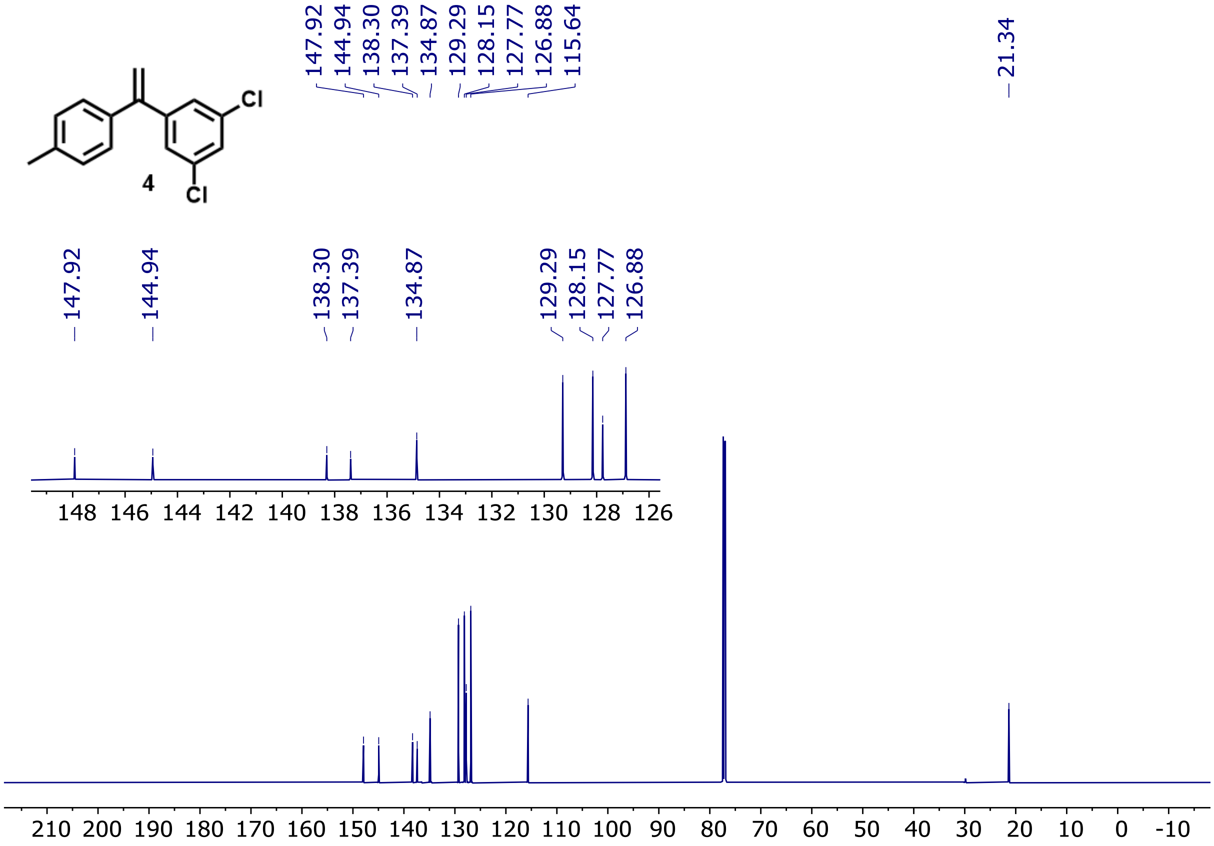
147.92
144.94
138.30
137.39
134.87
129.29
128.15
127.77
126.88
115.64
21.34
Cl
Cl
4
148 146 144 142 140 138 136 134 132 130 128 126
210 200 190 180 170 160 150 140 130 120 110 100 90 80 70 60 50 40 30 20 10 0 -10

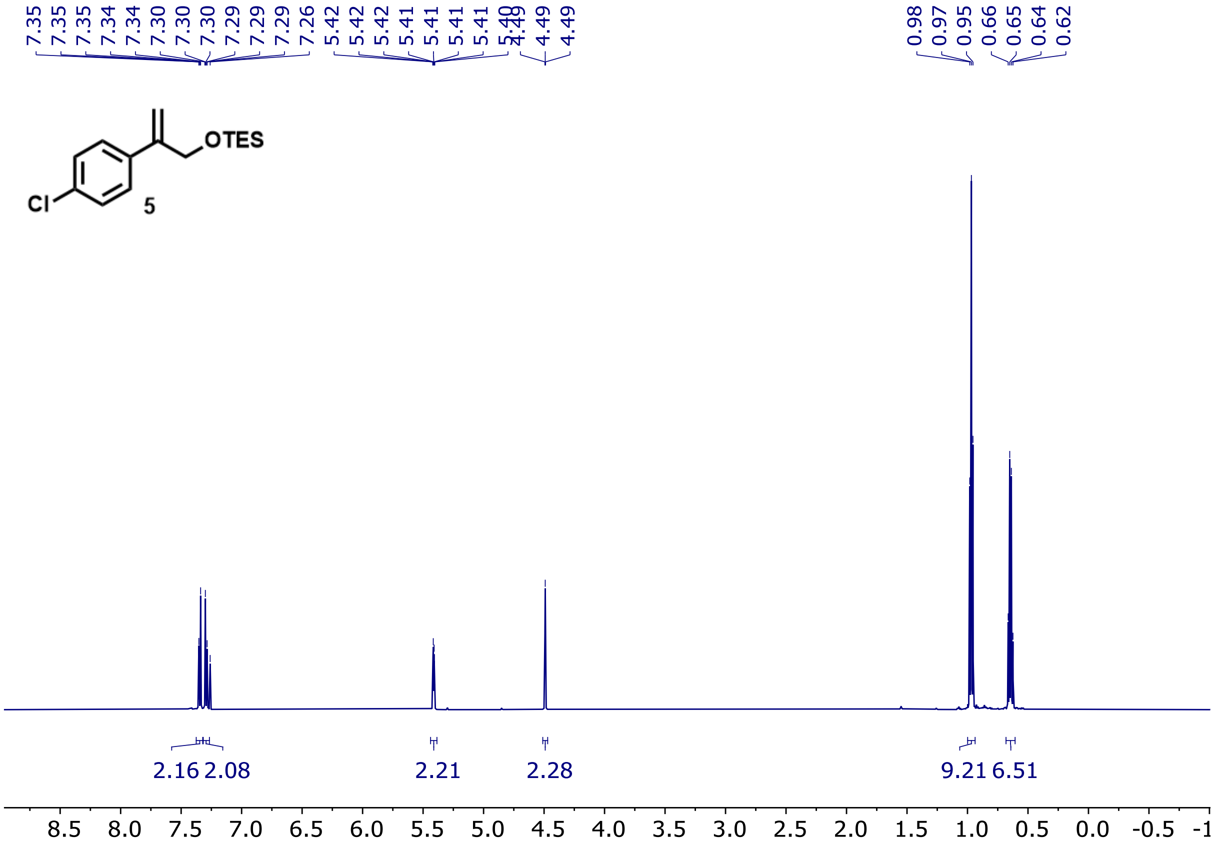
7.35
7.35
7.35
7.34
7.34
7.30
7.30
7.30
7.29
7.29
7.29
7.26
5.42
5.42
5.42
5.41
5.41
5.41
5.41
5.40
4.49
4.49
4.49
0.98
0.97
0.95
0.66
0.65
0.64
0.62
OTES
Cl
5
2.16 2.08
2.21
2.28
9.21 6.51
8.5 8.0 7.5 7.0 6.5 6.0 5.5 5.0 4.5 4.0 3.5 3.0 2.5 2.0 1.5 1.0 0.5 0.0 -0.5 -1

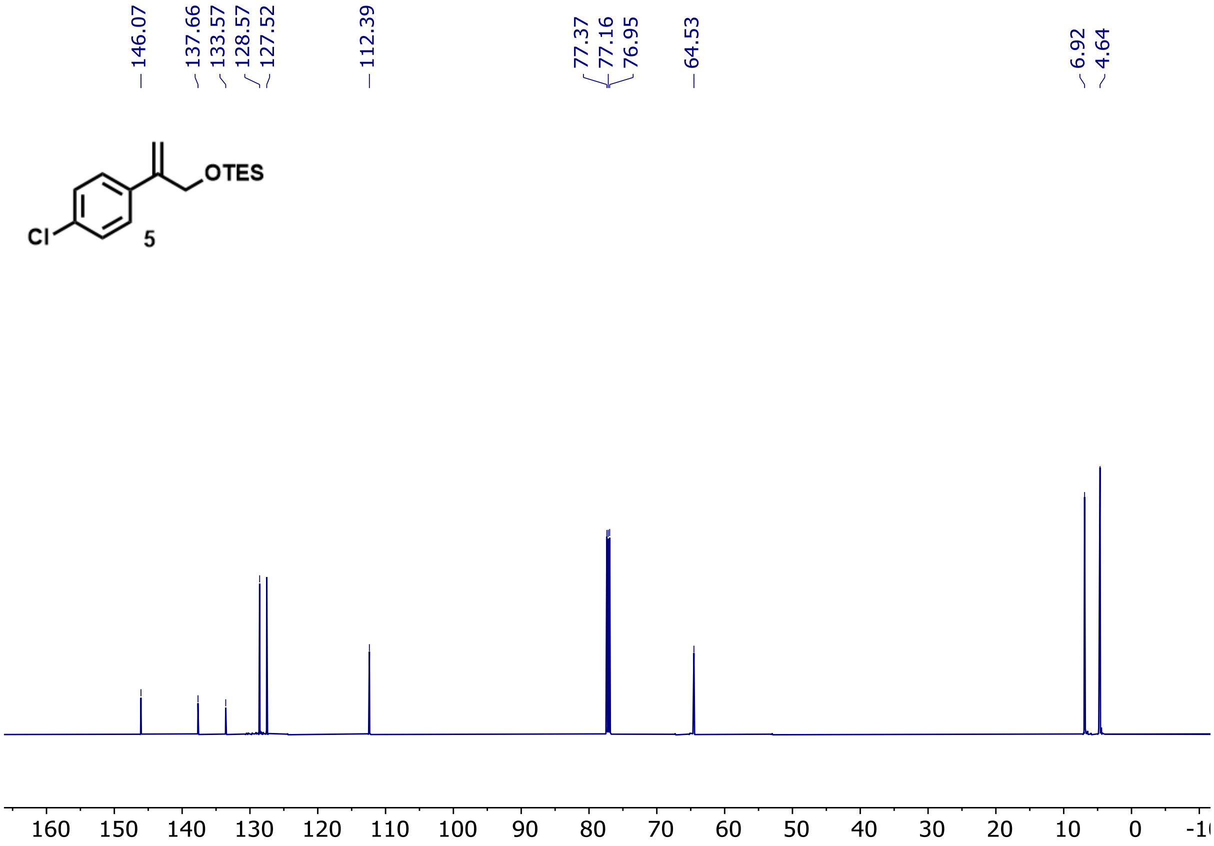
146.07
137.66
133.57
128.57
127.52
112.39
77.37
77.16
76.95
64.53
6.92
4.64
OTES
Cl
5
160 150 140 130 120 110 100 90 80 70 60 50 40 30 20 10 0 -1

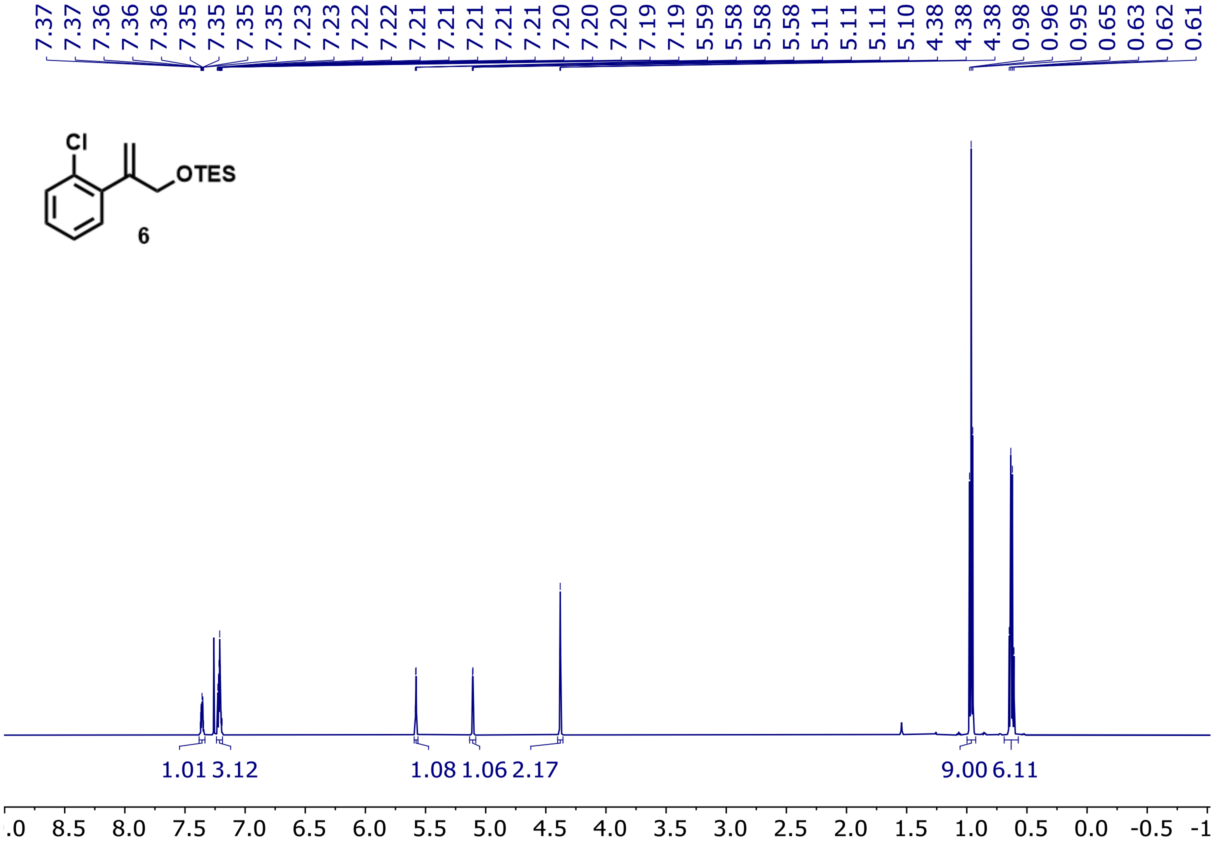
7.37 7.37 7.36 7.36 7.36 7.35 7.35 7.35 7.35 7.23 7.23 7.22 7.22 7.21 7.21 7.21 7.21 7.21 7.20 7.20 7.20 7.19 7.19 5.59 5.58 5.58 5.58 5.11 5.11 5.11 5.10 4.38 4.38 4.38 0.98 0.96 0.95 0.65 0.63 0.62 0.61
Cl
OTES
6
1.01 3.12
1.08 1.06 2.17
9.00 6.11
.0 8.5 8.0 7.5 7.0 6.5 6.0 5.5 5.0 4.5 4.0 3.5 3.0 2.5 2.0 1.5 1.0 0.5 0.0 -0.5 -1

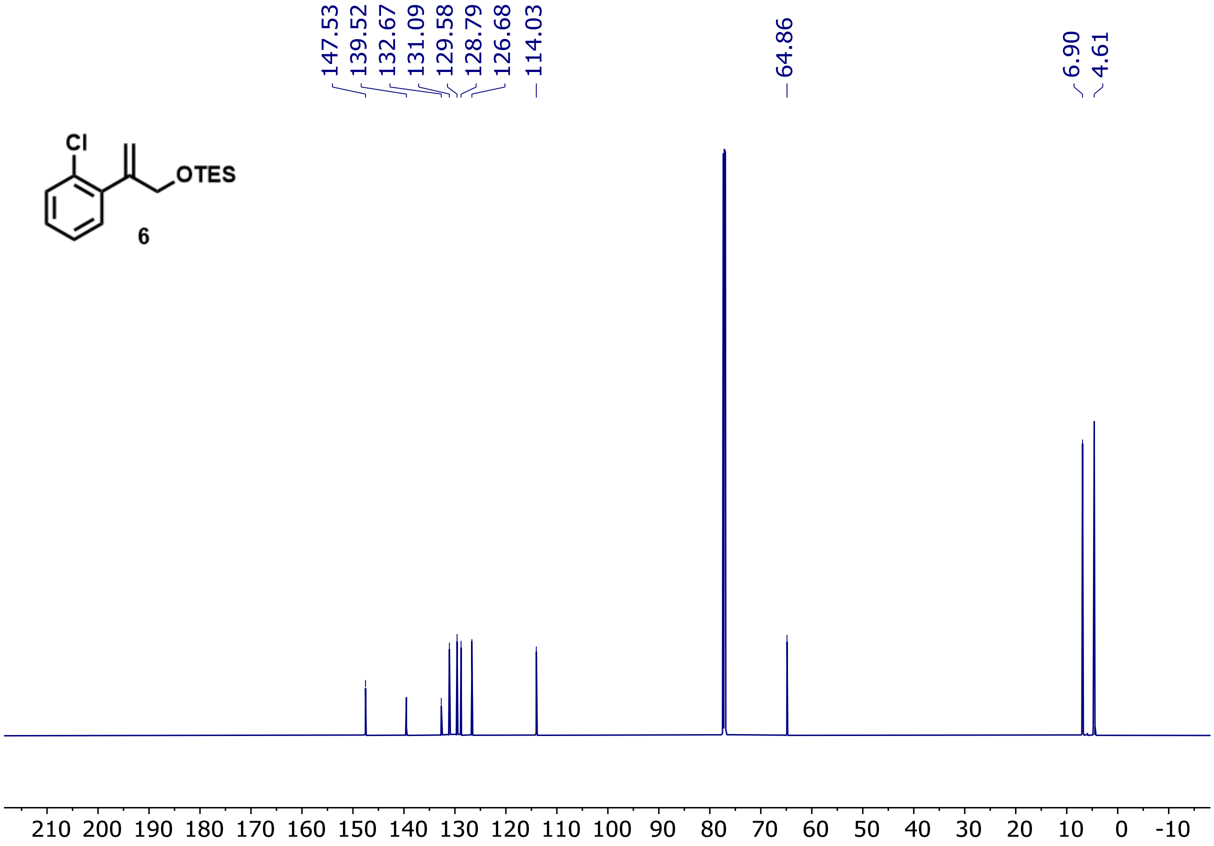
147.53
139.52
132.67
131.09
129.58
128.79
126.68
114.03
64.86
6.90
4.61
Cl
OTES
6
210 200 190 180 170 160 150 140 130 120 110 100 90 80 70 60 50 40 30 20 10 0 -10

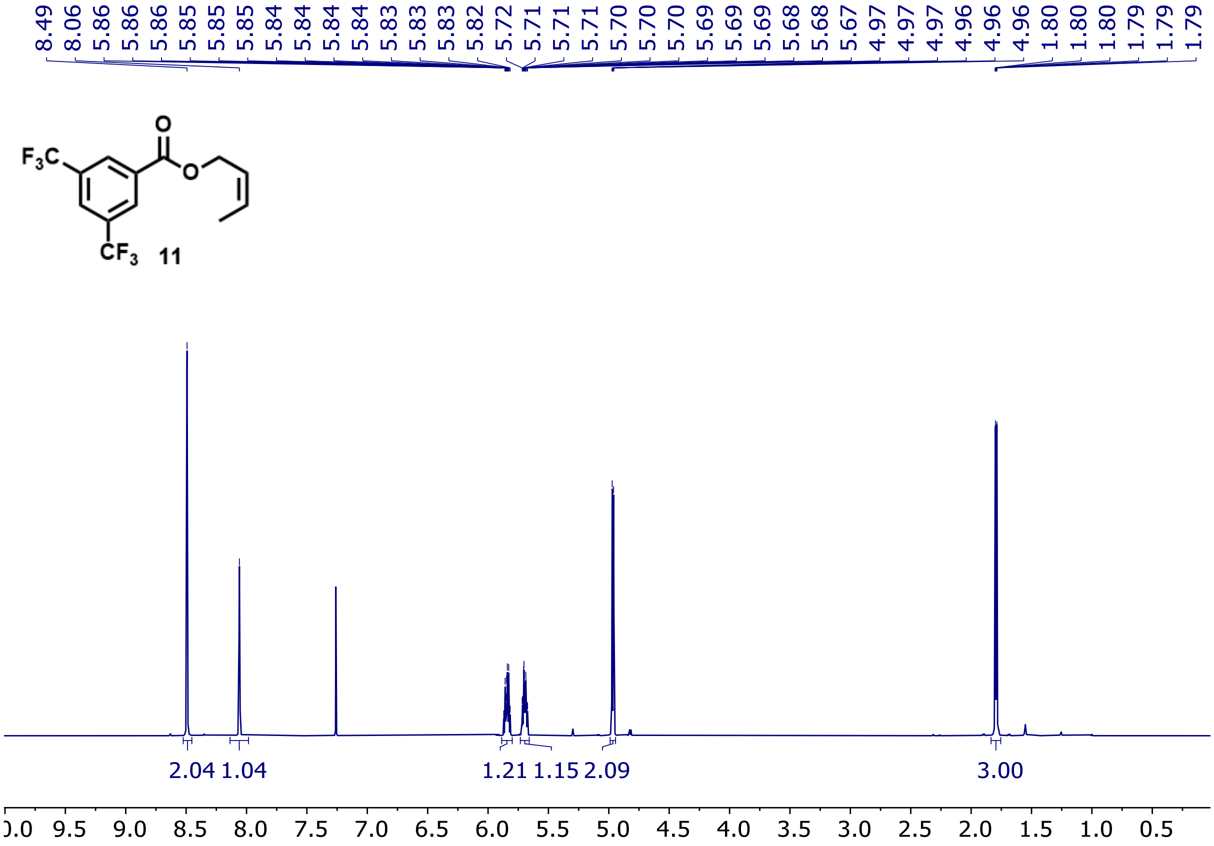
8.49
8.06
5.86
5.86
5.86
5.85
5.85
5.85
5.84
5.84
5.84
5.84
5.83
5.83
5.83
5.82
5.72
5.71
5.71
5.71
5.70
5.70
5.70
5.69
5.69
5.69
5.68
5.68
5.67
4.97
4.97
4.97
4.96
4.96
4.96
1.80
1.80
1.80
1.79
1.79
1.79
F3C
O
O
CF3
11
2.04
1.04
1.21
1.15
2.09
3.00
9.5 9.0 8.5 8.0 7.5 7.0 6.5 6.0 5.5 5.0 4.5 4.0 3.5 3.0 2.5 2.0 1.5 1.0 0.5

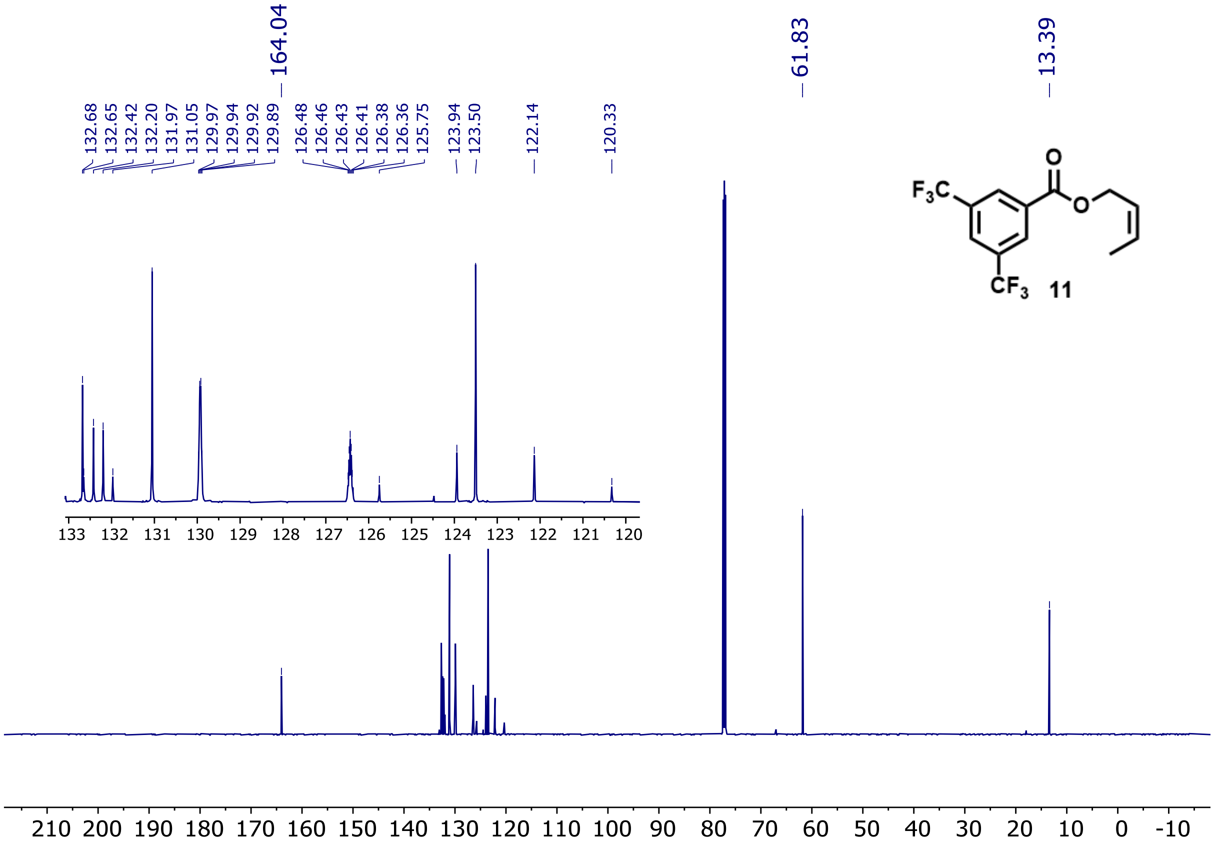
164.04
61.83
13.39
132.68
132.65
132.42
132.20
131.97
131.05
129.97
129.94
129.92
129.89
126.48
126.46
126.43
126.41
126.38
126.36
125.75
123.94
123.50
122.14
120.33
F3C
O
O
CF3
11
133 132 131 130 129 128 127 126 125 124 123 122 121 120
210 200 190 180 170 160 150 140 130 120 110 100 90 80 70 60 50 40 30 20 10 0 -10

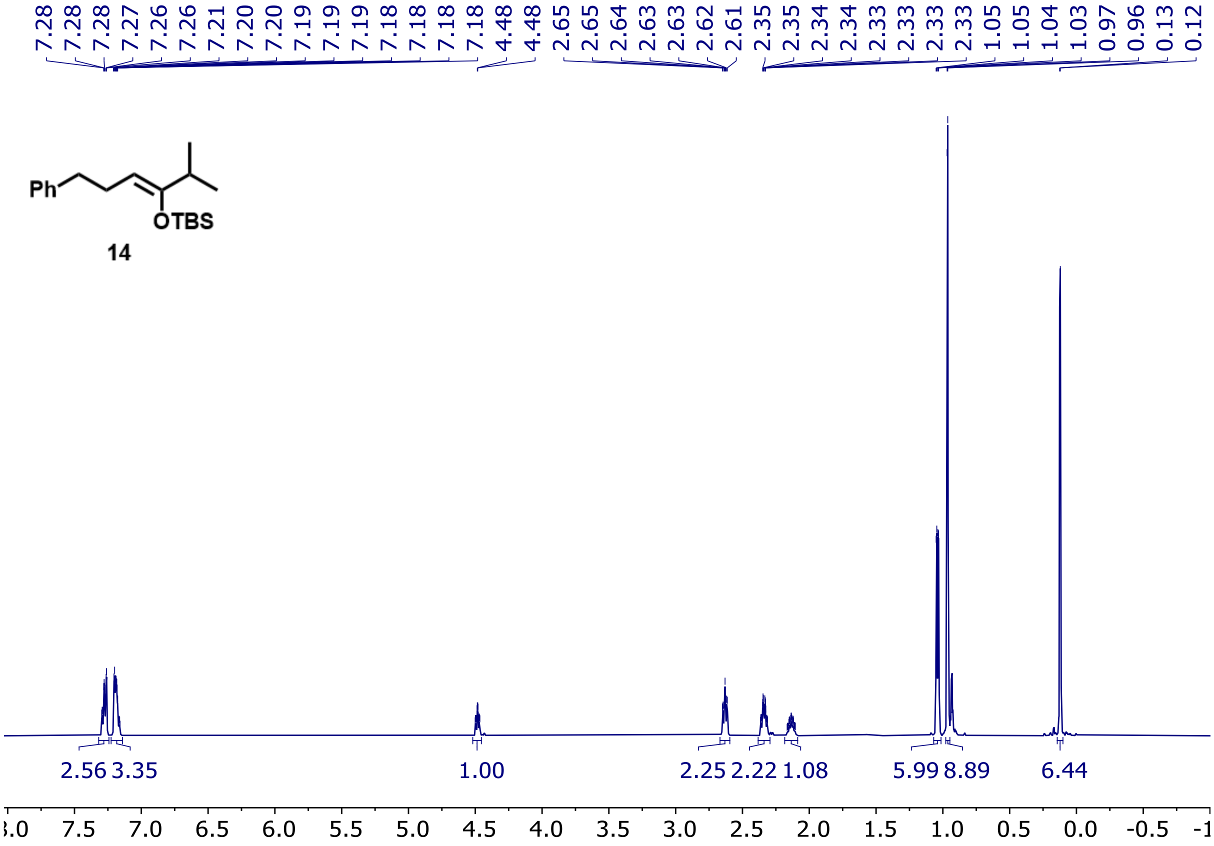
7.28 7.28 7.28 7.27 7.26 7.26 7.21 7.20 7.20 7.19 7.19 7.19 7.18 7.18 7.18 7.18 4.48 4.48 2.65 2.65 2.64 2.63 2.63 2.62 2.61 2.35 2.35 2.34 2.34 2.33 2.33 2.33 2.33 1.05 1.05 1.04 1.03 0.97 0.96 0.13 0.12
Ph
OTBS
14
2.56 3.35
1.00
2.25 2.22 1.08
5.99 8.89
6.44
8.0 7.5 7.0 6.5 6.0 5.5 5.0 4.5 4.0 3.5 3.0 2.5 2.0 1.5 1.0 0.5 0.0 -0.5 -1

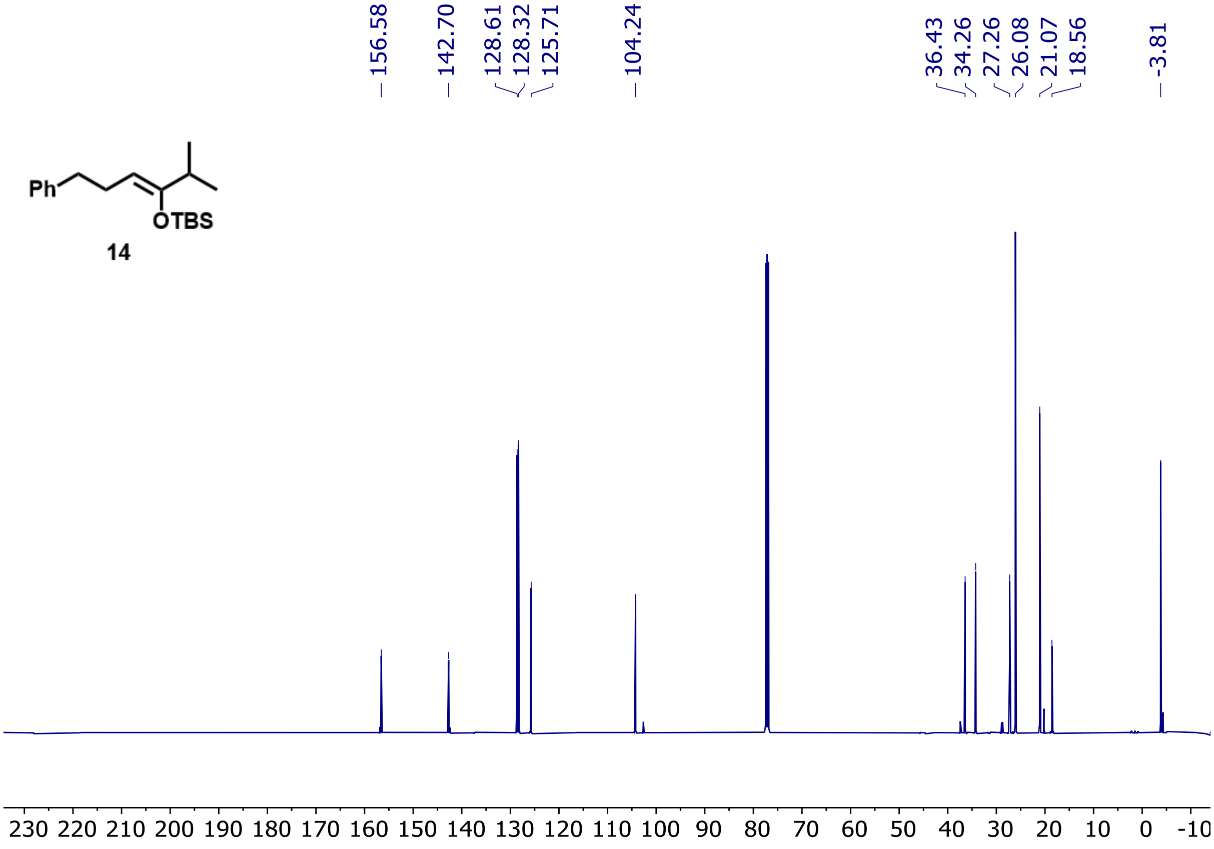
156.58
142.70
128.61 128.32 125.71
104.24
36.43 34.26 27.26 26.08 21.07 18.56
-3.81
Ph
OTBS
14
230 220 210 200 190 180 170 160 150 140 130 120 110 100 90 80 70 60 50 40 30 20 10 0 -10

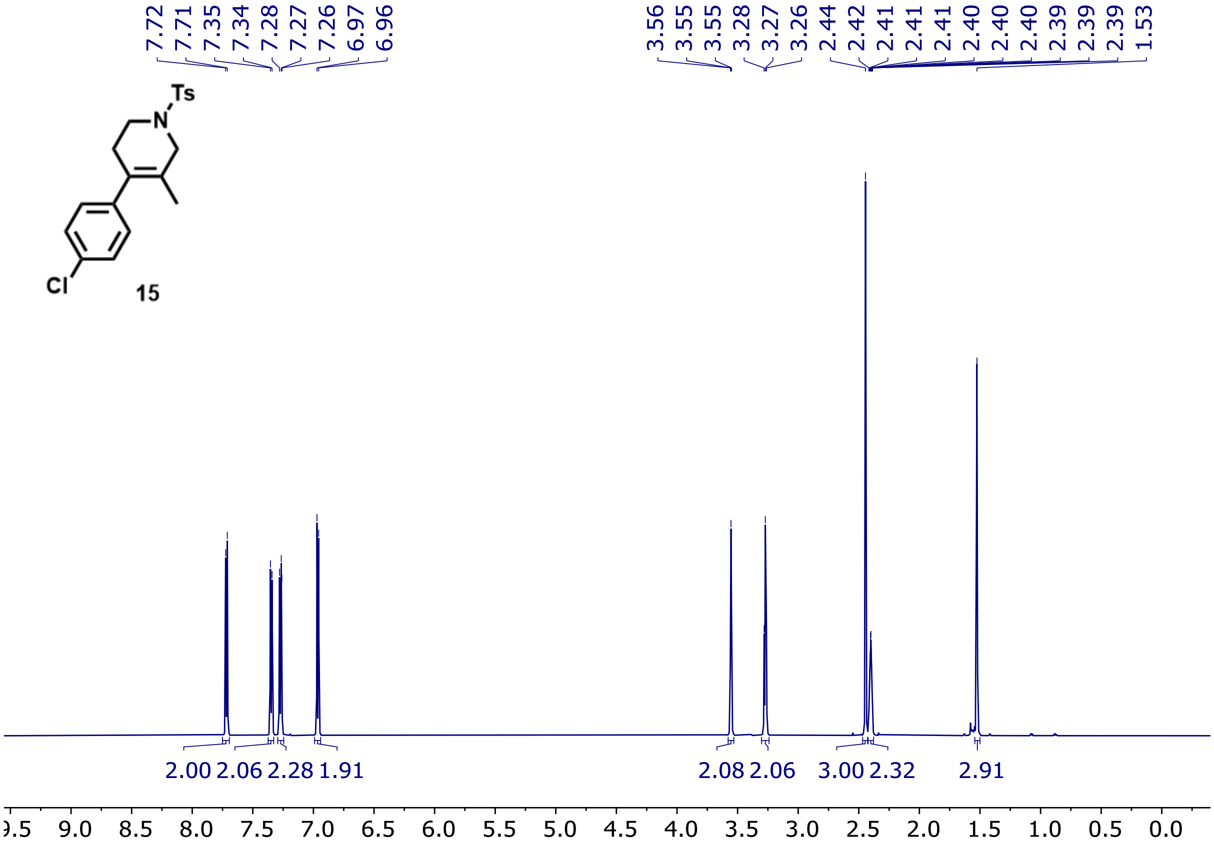
7.72
7.71
7.35
7.34
7.28
7.27
7.26
6.97
6.96
3.56
3.55
3.55
3.28
3.27
3.26
2.44
2.42
2.41
2.41
2.41
2.40
2.40
2.40
2.39
2.39
2.39
1.53
Ts
N
Cl
15
2.00 2.06 2.28 1.91
2.08 2.06 3.00 2.32 2.91
9.5 9.0 8.5 8.0 7.5 7.0 6.5 6.0 5.5 5.0 4.5 4.0 3.5 3.0 2.5 2.0 1.5 1.0 0.5 0.0

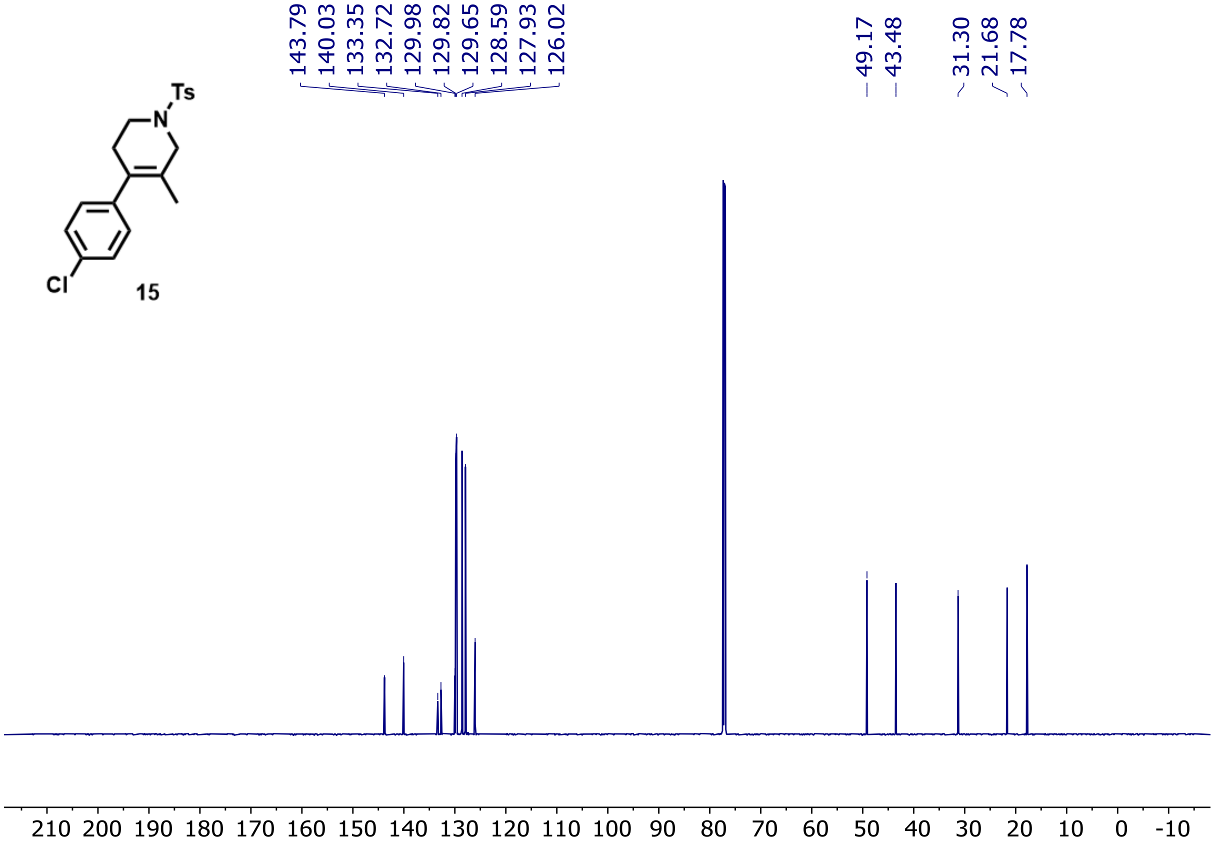
143.79
140.03
133.35
132.72
129.98
129.82
129.65
128.59
127.93
126.02
49.17
43.48
31.30
21.68
17.78
Ts
N
Cl
15
210 200 190 180 170 160 150 140 130 120 110 100 90 80 70 60 50 40 30 20 10 0 -10

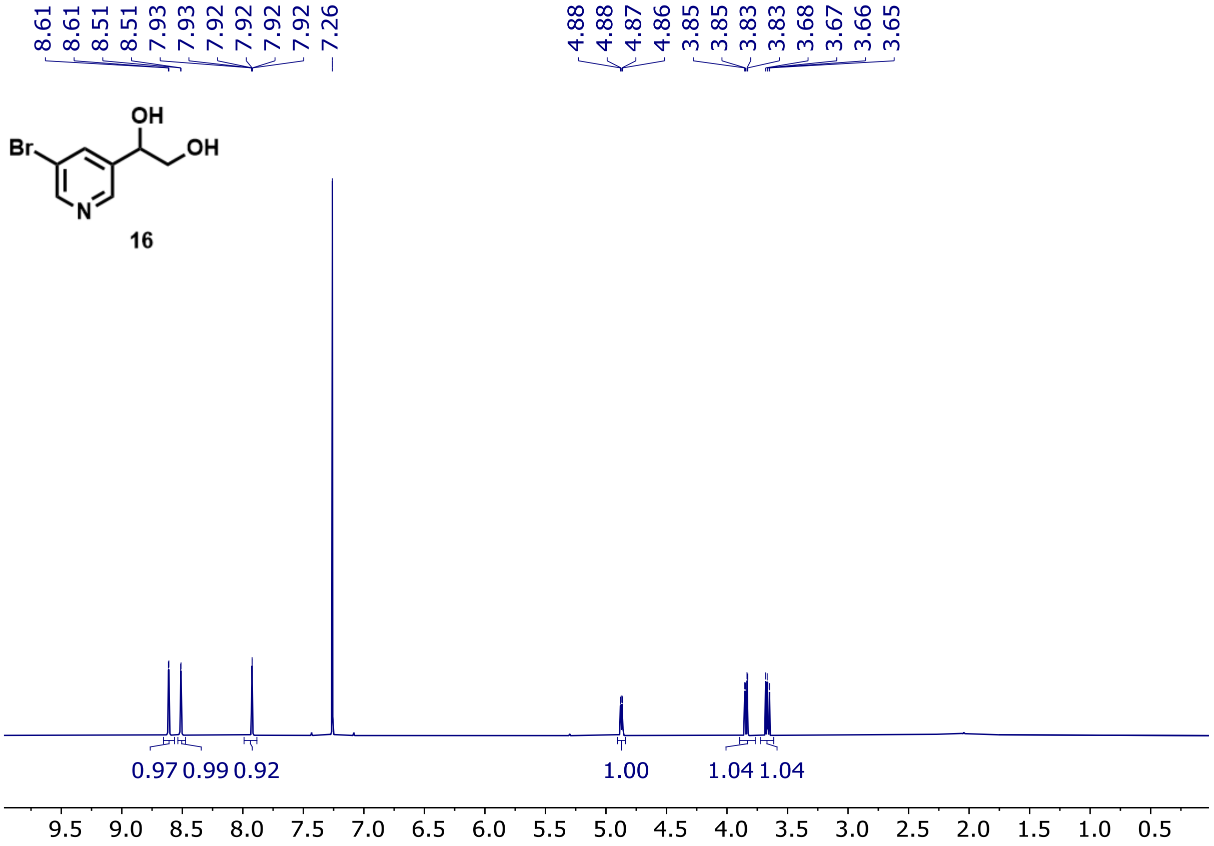
8.61
8.61
8.51
8.51
7.93
7.93
7.92
7.92
7.92
7.92
7.26
4.88
4.88
4.87
4.86
3.85
3.85
3.83
3.83
3.68
3.67
3.66
3.65
Br
OH
OH
N
16
0.97
0.99
0.92
1.00
1.04
1.04
9.5 9.0 8.5 8.0 7.5 7.0 6.5 6.0 5.5 5.0 4.5 4.0 3.5 3.0 2.5 2.0 1.5 1.0 0.5

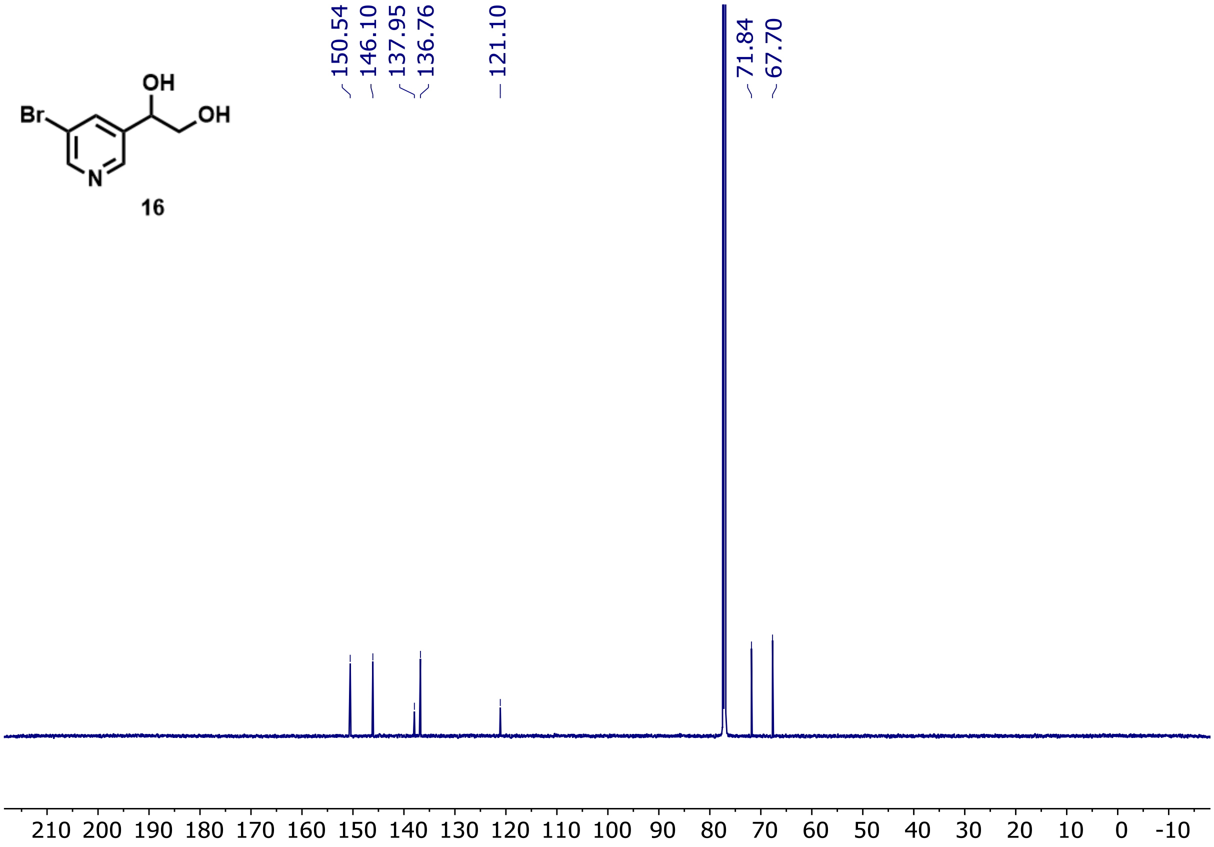
150.54
146.10
137.95
136.76
121.10
71.84
67.70
Br
OH
OH
N
16
210 200 190 180 170 160 150 140 130 120 110 100 90 80 70 60 50 40 30 20 10 0 -10

16 (racemic):

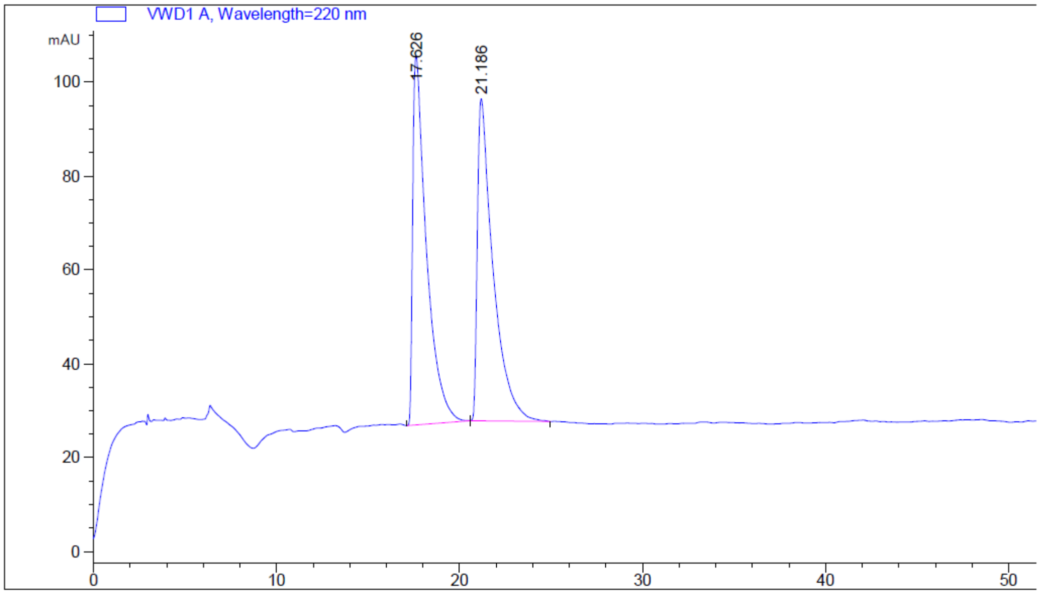

Peak #	RetTime [min]	Type	Width [min]	Area [mAU*s]	Height [mAU]	Area %
1	17.626	BB	0.7434	4101.59277	78.70188	50.0353
2	21.186	BB	0.8347	4095.80444	68.62176	49.9647

16' (*R*-isomer):

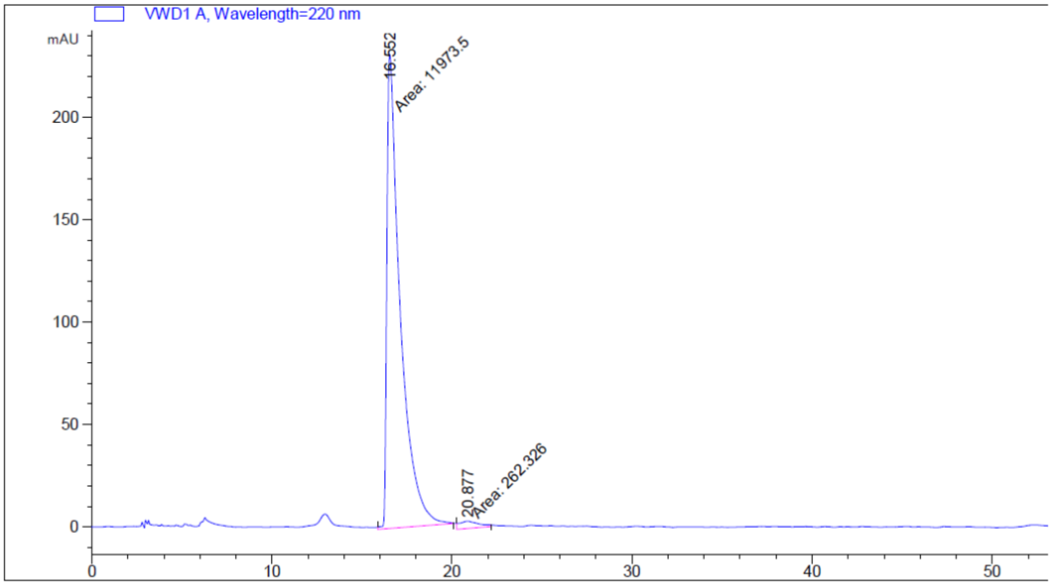

Peak #	RetTime [min]	Type	Width [min]	Area [mAU*s]	Height [mAU]	Area %
1	16.552	MM	0.8626	1.19735e4	231.34061	97.8561
2	20.877	MM	1.2259	262.32596	3.56635	2.1439

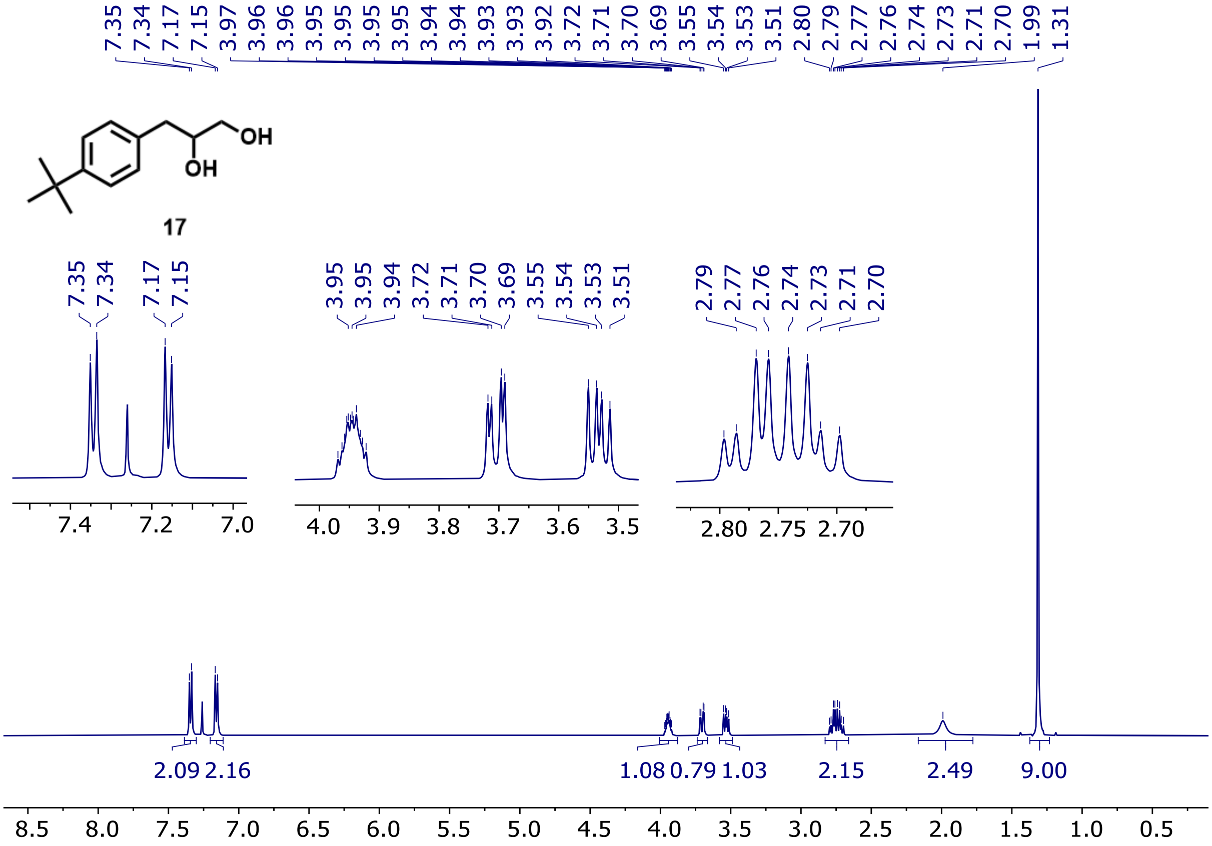
7.35
7.34
7.17
7.15
3.97
3.96
3.96
3.95
3.95
3.95
3.95
3.94
3.94
3.93
3.93
3.92
3.72
3.71
3.70
3.69
3.55
3.54
3.53
3.51
2.80
2.79
2.77
2.76
2.74
2.73
2.71
2.70
1.99
1.31
OH
OH
17
2.09
2.16
1.08
0.79
1.03
2.15
2.49
9.00
8.5 8.0 7.5 7.0 6.5 6.0 5.5 5.0 4.5 4.0 3.5 3.0 2.5 2.0 1.5 1.0 0.5

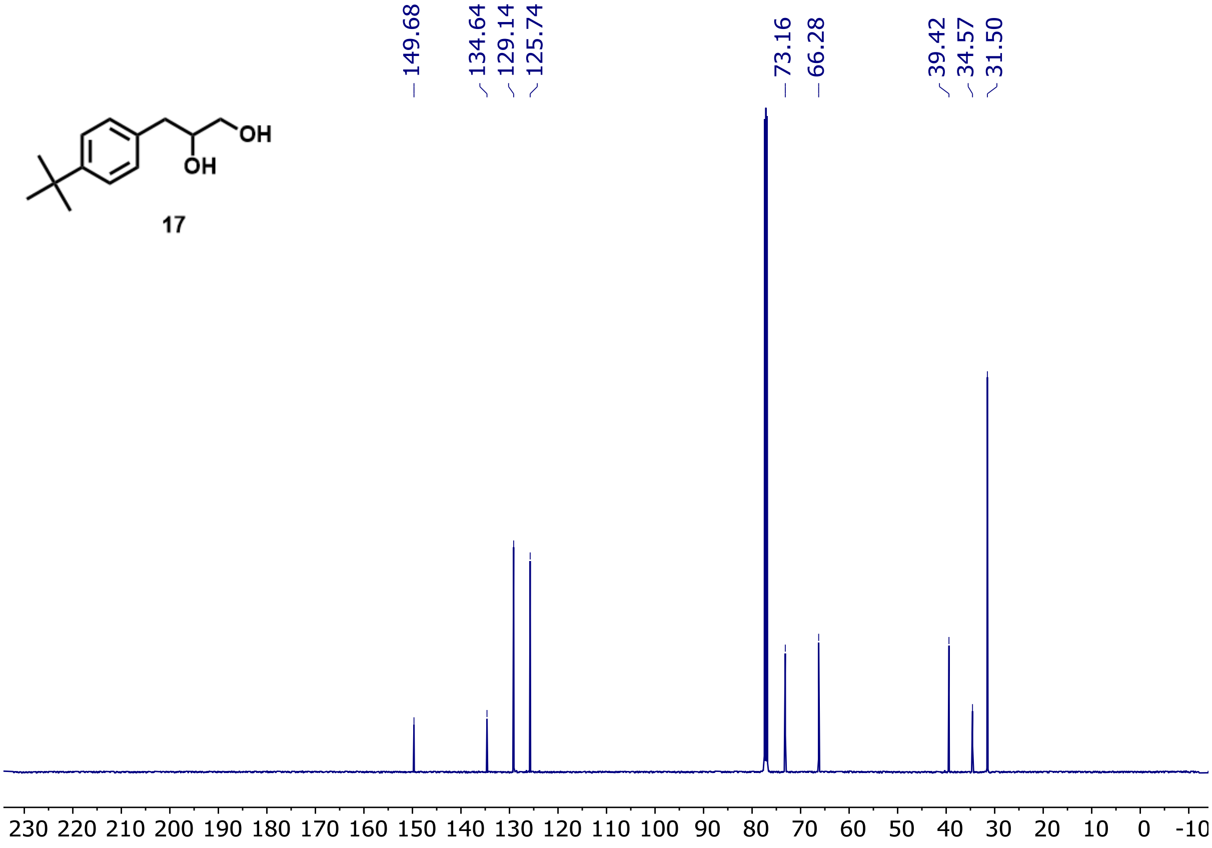
149.68
134.64
129.14
125.74
73.16
66.28
39.42
34.57
31.50
OH
OH
17
230 220 210 200 190 180 170 160 150 140 130 120 110 100 90 80 70 60 50 40 30 20 10 0 -10

17 (racemic):

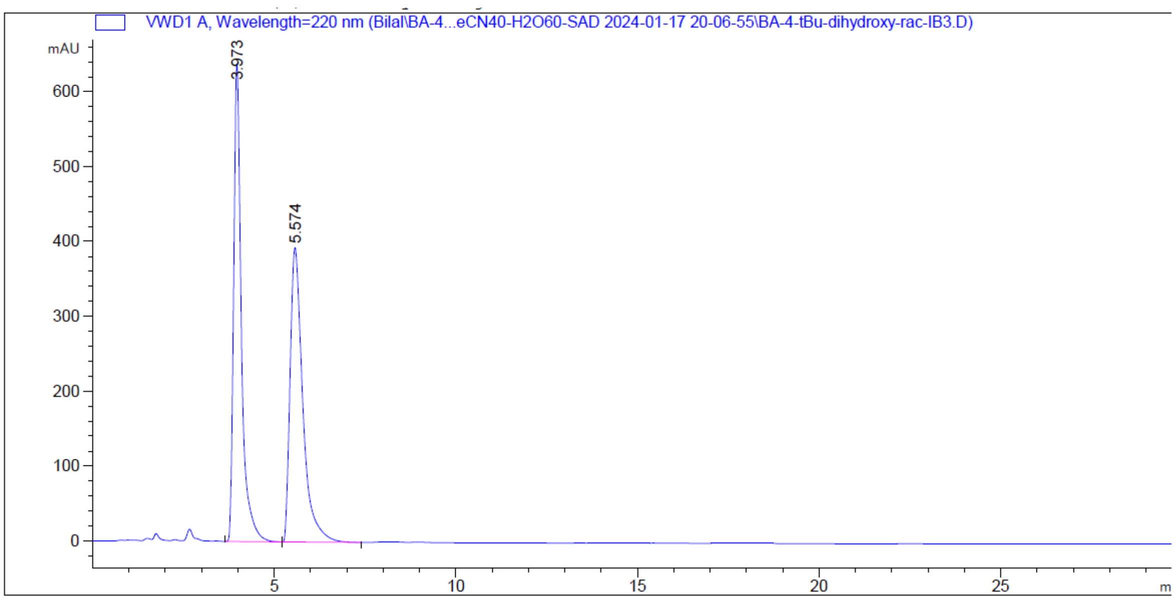

Peak #	RetTime [min]	Type	Width [min]	Area [mAU*s]	Height [mAU]	Area %
1	3.973	BB	0.2196	9497.18555	638.12018	49.9156
2	5.574	BB	0.3668	9529.28418	393.18967	50.0844

17' (*S*-isomer):

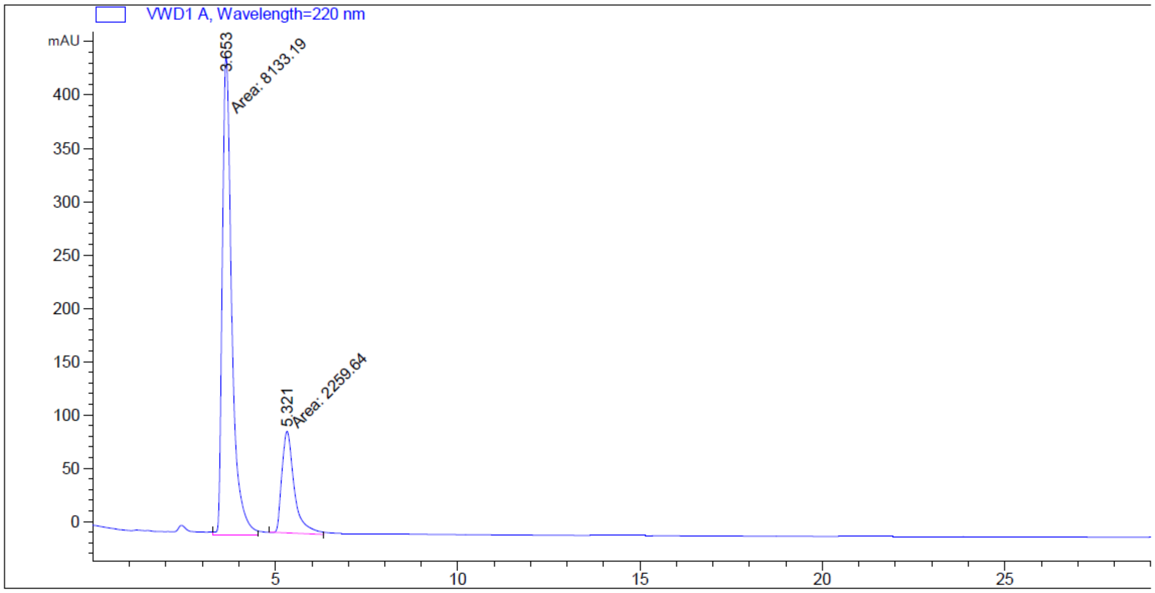

Peak #	RetTime [min]	Type	Width [min]	Area [mAU*s]	Height [mAU]	Area %
1	3.653	MM	0.3020	8133.18994	448.78531	78.2577
2	5.321	MM	0.3954	2259.64063	95.25250	21.7423

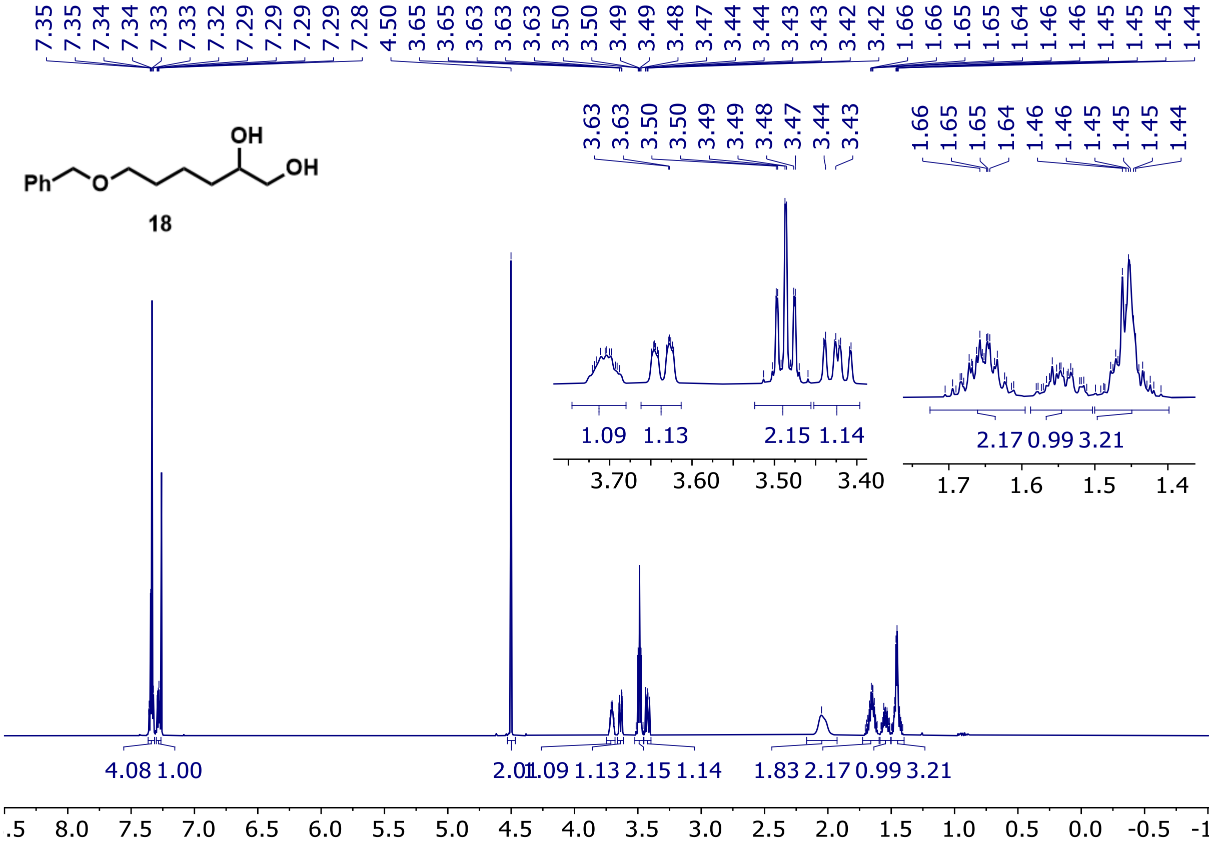
7.35 7.35 7.34 7.34 7.33 7.33 7.32 7.29 7.29 7.29 7.29 7.28 4.50 3.65 3.65 3.63 3.63 3.63 3.50 3.50 3.49 3.49 3.48 3.47 3.44 3.44 3.43 3.43 3.42 3.42 1.66 1.66 1.65 1.65 1.64 1.46 1.46 1.45 1.45 1.45 1.44
OH
Ph
O
OH
18
3.63 3.63 3.50 3.50 3.49 3.49 3.48 3.47 3.44 3.43
1.66 1.65 1.65 1.64 1.46 1.46 1.45 1.45 1.45 1.44
1.09 1.13 2.15 1.14
3.70 3.60 3.50 3.40
2.17 0.99 3.21
1.7 1.6 1.5 1.4
4.08 1.00
2.01 1.09 1.13 2.15 1.14
1.83 2.17 0.99 3.21
.5 8.0 7.5 7.0 6.5 6.0 5.5 5.0 4.5 4.0 3.5 3.0 2.5 2.0 1.5 1.0 0.5 0.0 -0.5 -1

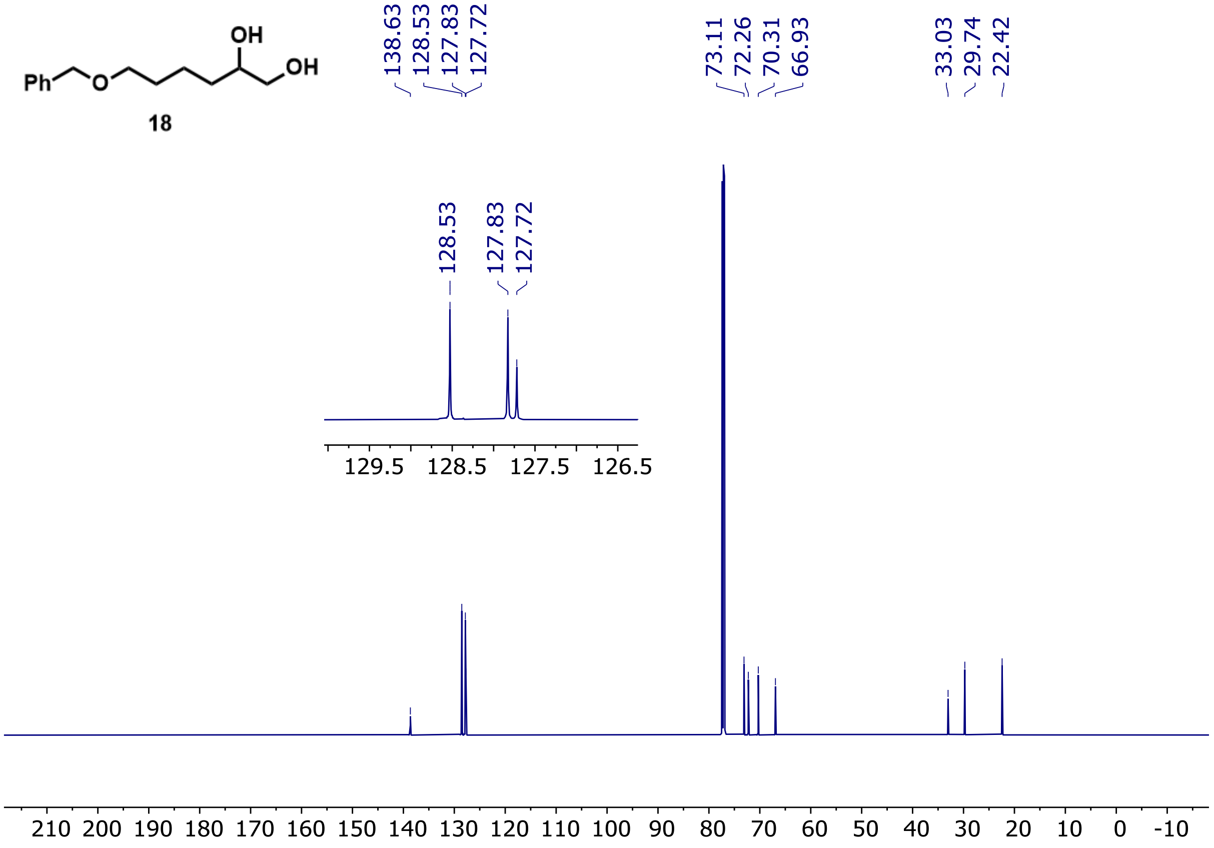
138.63 128.53 127.83 127.72
73.11 72.26 70.31 66.93
33.03 29.74 22.42
OH
Ph
O
OH
18
128.53
127.83 127.72
129.5 128.5 127.5 126.5
210 200 190 180 170 160 150 140 130 120 110 100 90 80 70 60 50 40 30 20 10 0 -10

18 (racemic):

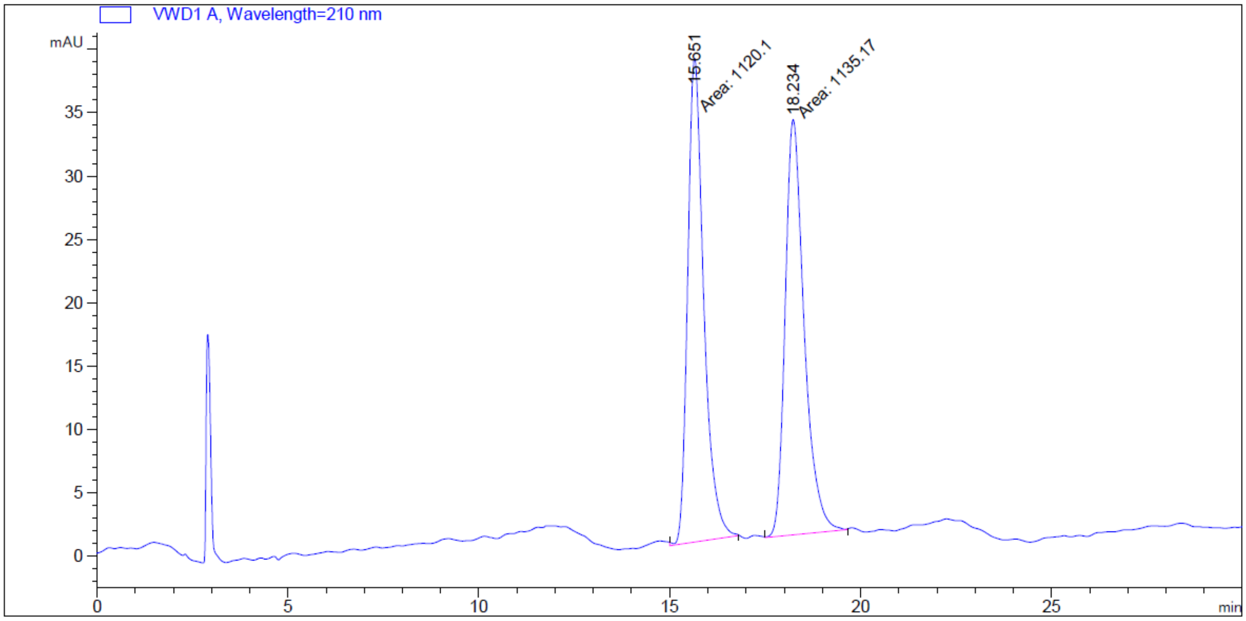

Peak #	RetTime [min]	Type	Width [min]	Area [mAU*s]	Height [mAU]	Area %
1	15.651	MM	0.4886	1120.09875	38.20772	49.6658
2	18.234	MM	0.5770	1135.17297	32.79165	50.3342

18 (*R*-isomer):

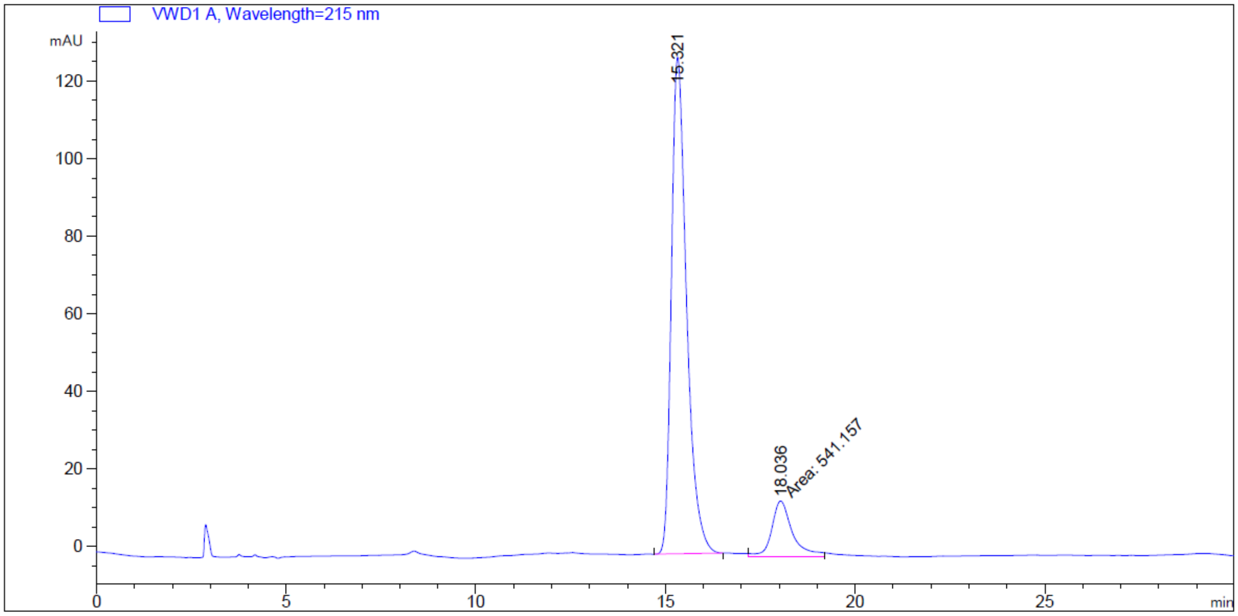

Peak #	RetTime [min]	Type	Width [min]	Area [mAU*s]	Height [mAU]	Area %
1	15.321	BB	0.4243	3571.40552	128.09727	86.8414
2	18.036	MM	0.6299	541.15735	14.31909	13.1586

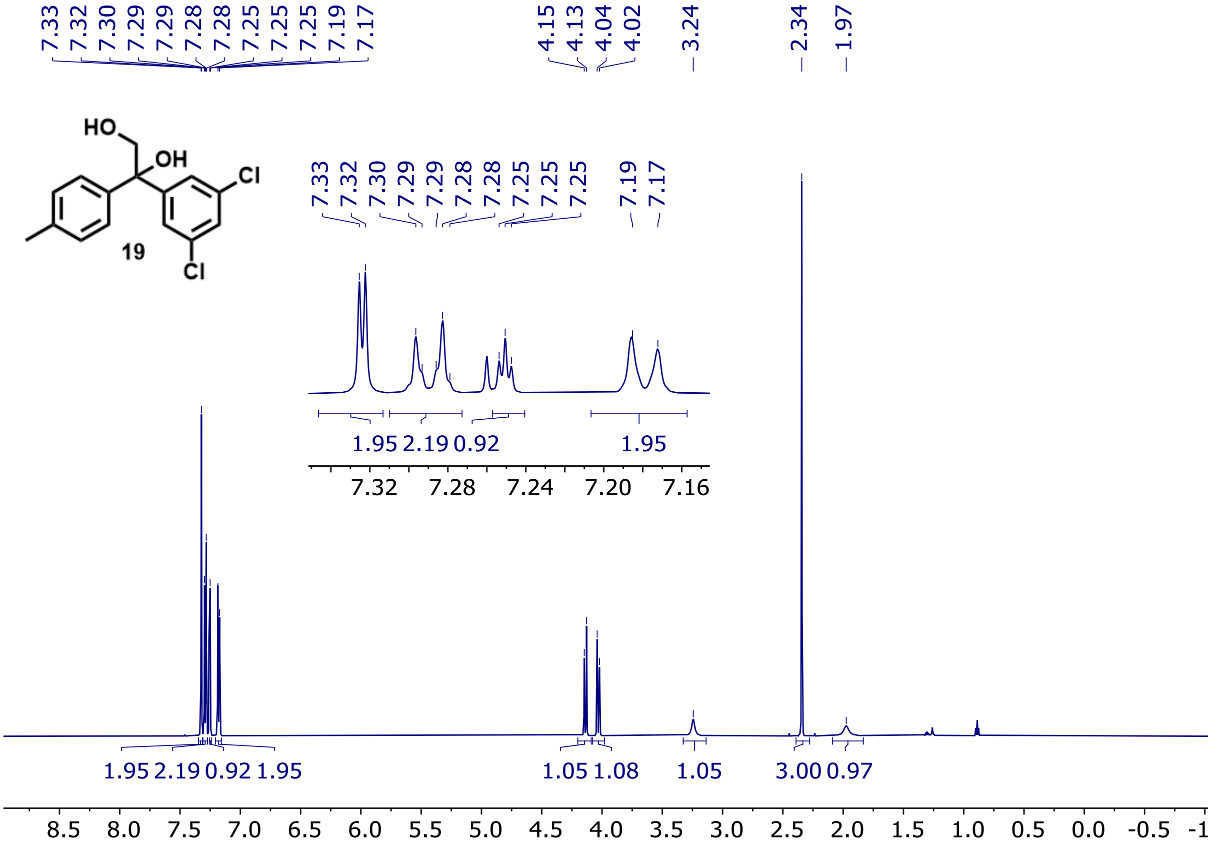
7.33 7.32 7.30 7.29 7.29 7.28 7.28 7.25 7.25 7.25 7.19 7.17
4.15 4.13 4.04 4.02
3.24
2.34
1.97
HO
OH
Cl
Cl
19
1.95 2.19 0.92 1.95
7.32 7.28 7.24 7.20 7.16
1.95 2.19 0.92 1.95
1.05 1.08
1.05
3.00 0.97
8.5 8.0 7.5 7.0 6.5 6.0 5.5 5.0 4.5 4.0 3.5 3.0 2.5 2.0 1.5 1.0 0.5 0.0 -0.5 -1

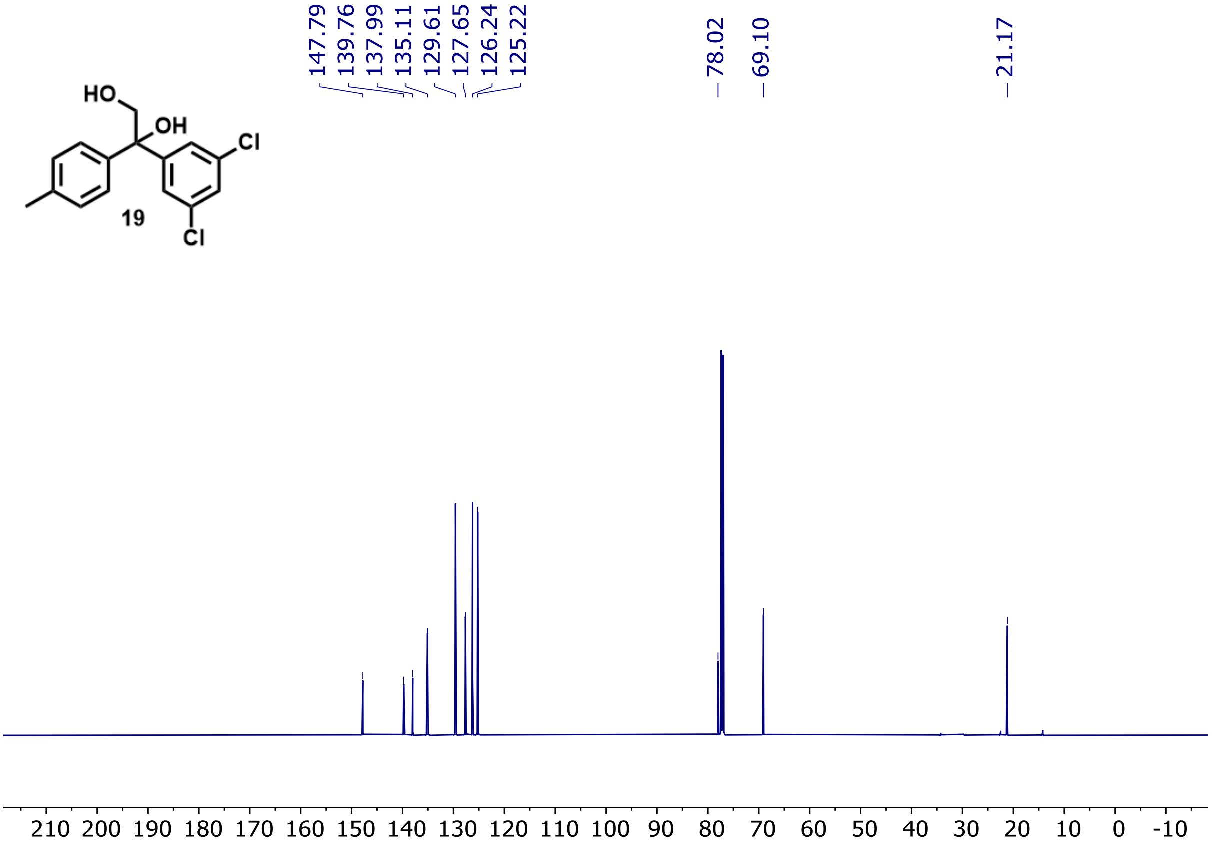
147.79 139.76 137.99 135.11 129.61 127.65 126.24 125.22
78.02
69.10
21.17
HO
OH
Cl
Cl
19
210 200 190 180 170 160 150 140 130 120 110 100 90 80 70 60 50 40 30 20 10 0 -10

19 (racemic):

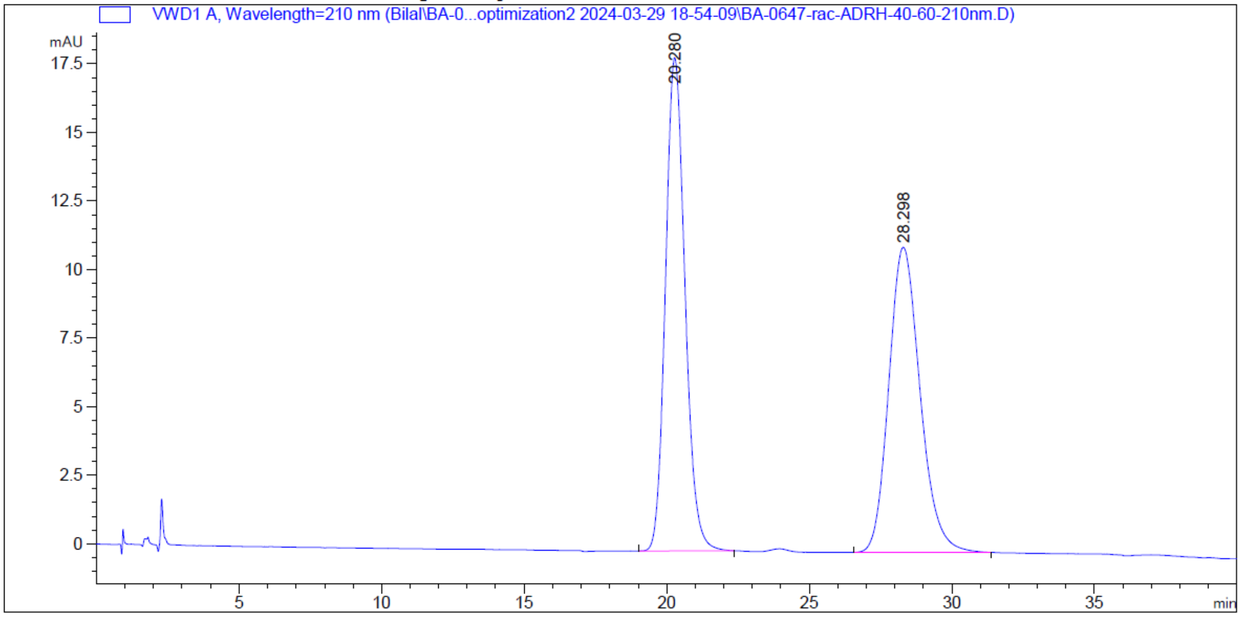

Peak #	RetTime [min]	Type	Width [min]	Area [mAU*s]	Height [mAU]	Area %
1	20.280	BB	0.7317	854.94342	17.98114	50.2069
2	28.298	BB	1.1353	847.89539	11.12292	49.7931

19' (*R*-isomer):

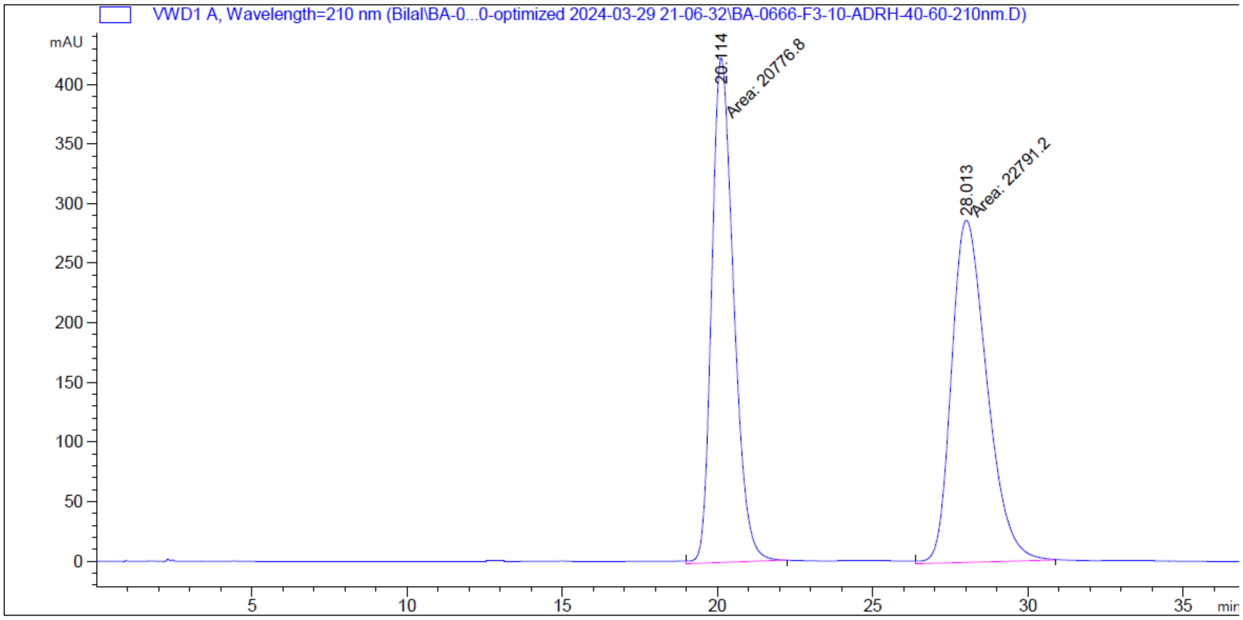

Peak #	RetTime [min]	Type	Width [min]	Area [mAU*s]	Height [mAU]	Area %
1	20.114	MM	0.8181	2.07768e4	423.28607	47.6882
2	28.013	MM	1.3231	2.27912e4	287.09952	52.3118

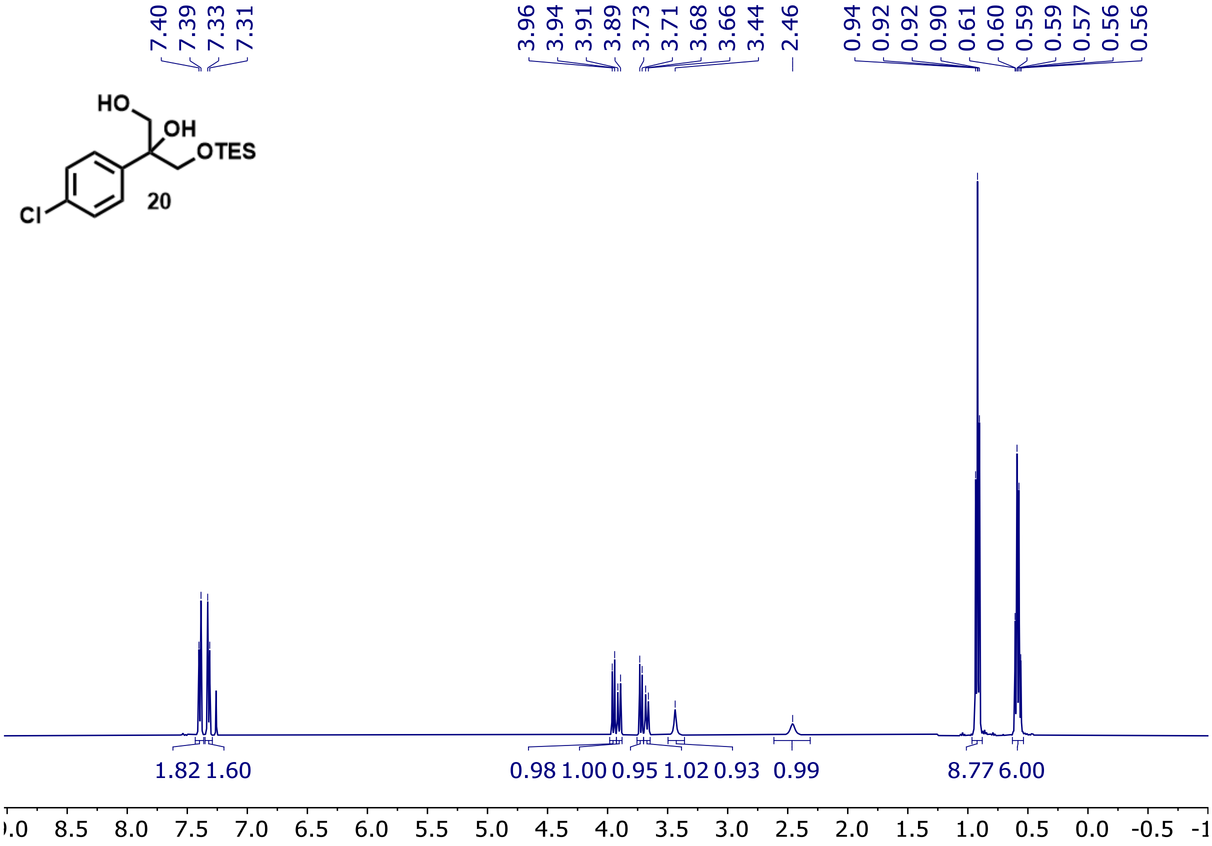
7.40
7.39
7.33
7.31
3.96
3.94
3.91
3.89
3.73
3.71
3.68
3.66
3.44
2.46
0.94
0.92
0.92
0.90
0.61
0.60
0.59
0.59
0.57
0.56
0.56
HO
OH
OTES
Cl
20
1.82 1.60
0.98 1.00 0.95 1.02 0.93 0.99
8.77 6.00
8.5 8.0 7.5 7.0 6.5 6.0 5.5 5.0 4.5 4.0 3.5 3.0 2.5 2.0 1.5 1.0 0.5 0.0 -0.5

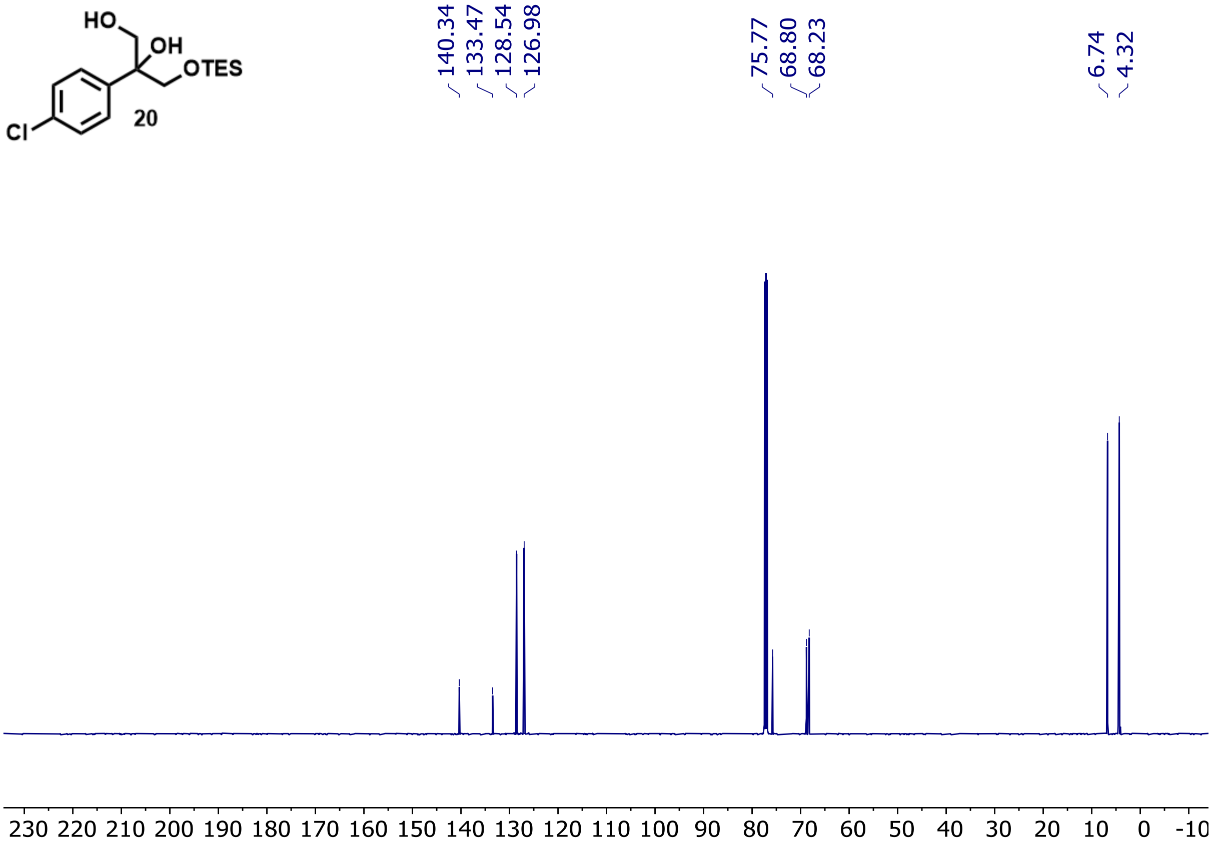
HO
OH
OTES
Cl
20
140.34
133.47
128.54
126.98
75.77
68.80
68.23
6.74
4.32
230 220 210 200 190 180 170 160 150 140 130 120 110 100 90 80 70 60 50 40 30 20 10 0 -10

20 (Racemic):

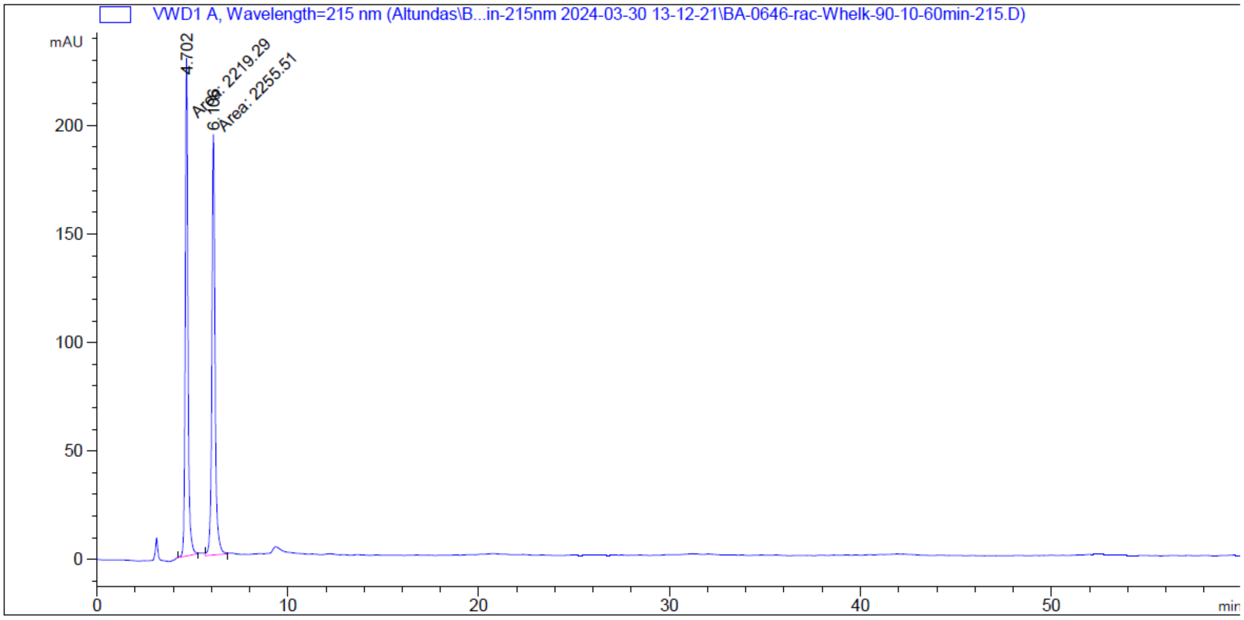

Peak #	RetTime [min]	Type	Width [min]	Area [mAU*s]	Height [mAU]	Area %
1	4.702	MM	0.1611	2219.29199	229.54710	49.5953
2	6.106	MM	0.1940	2255.51489	193.76390	50.4047

20' (*S*-isomer):

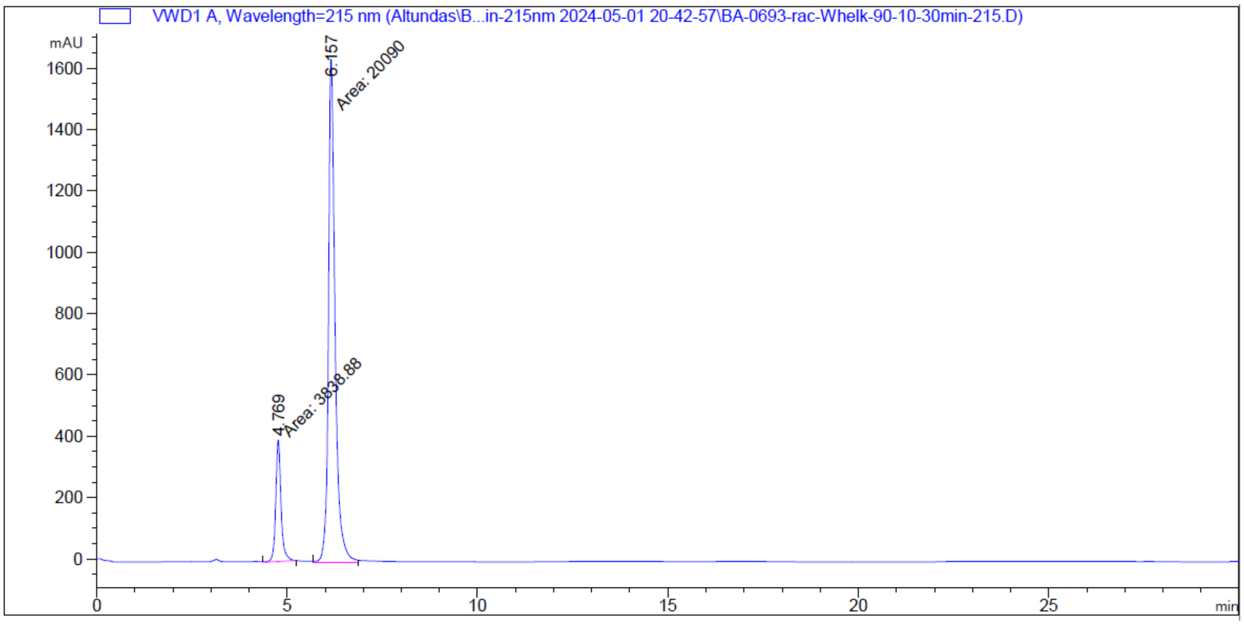

Peak #	RetTime [min]	Type	Width [min]	Area [mAU*s]	Height [mAU]	Area %
1	4.769	MM	0.1614	3838.87573	396.32620	16.0428
2	6.157	MM	0.2039	2.00900e4	1642.32251	83.9572

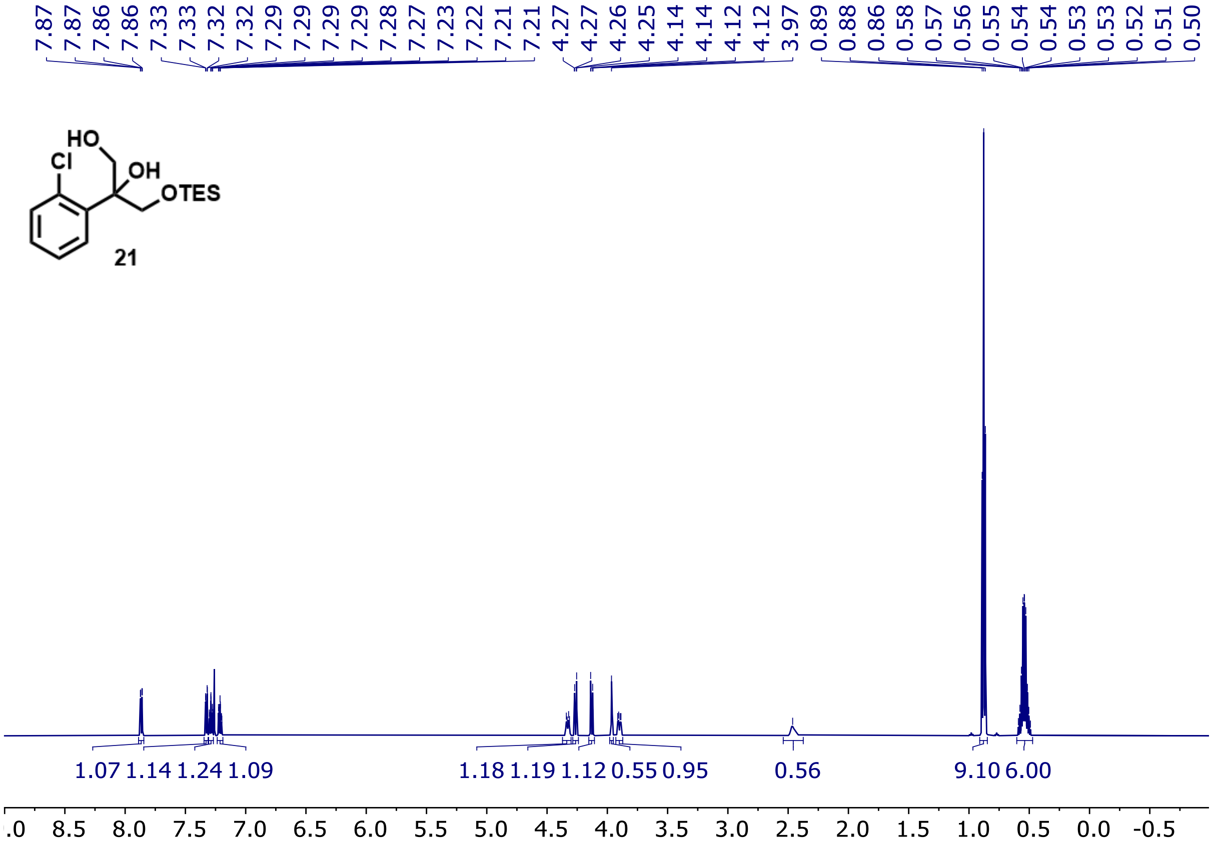
7.87
7.87
7.86
7.86
7.33
7.33
7.32
7.32
7.29
7.29
7.29
7.29
7.28
7.27
7.23
7.22
7.21
7.21
4.27
4.27
4.26
4.25
4.14
4.14
4.12
4.12
3.97
0.89
0.88
0.86
0.58
0.57
0.56
0.55
0.54
0.54
0.53
0.53
0.52
0.51
0.50
HO
Cl
OH
OTES
21
1.07
1.14
1.24
1.09
1.18
1.19
1.12
0.55
0.95
0.56
9.10
6.00
.0 8.5 8.0 7.5 7.0 6.5 6.0 5.5 5.0 4.5 4.0 3.5 3.0 2.5 2.0 1.5 1.0 0.5 0.0 -0.5

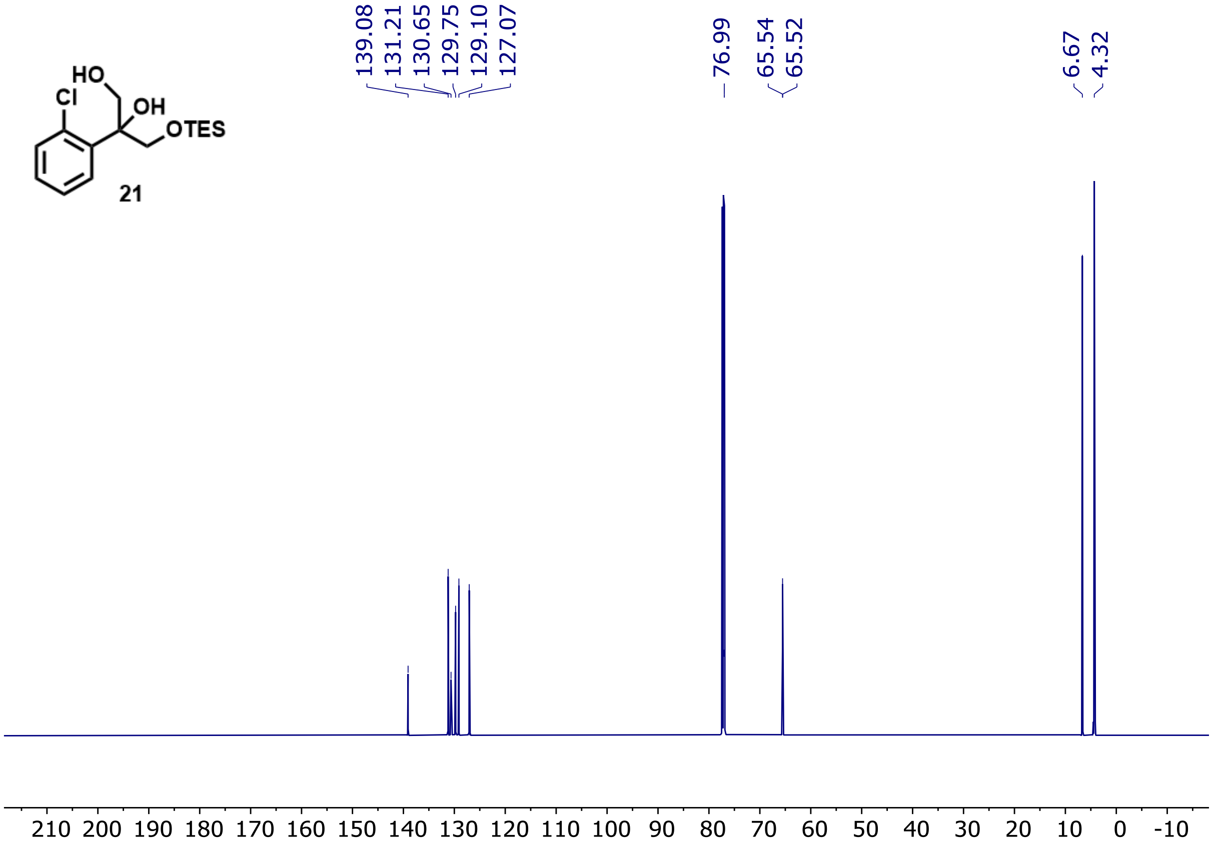
139.08
131.21
130.65
129.75
129.10
127.07
76.99
65.54
65.52
6.67
4.32
HO
Cl
OH
OTES
21
210 200 190 180 170 160 150 140 130 120 110 100 90 80 70 60 50 40 30 20 10 0 -10

21 (Racemic):

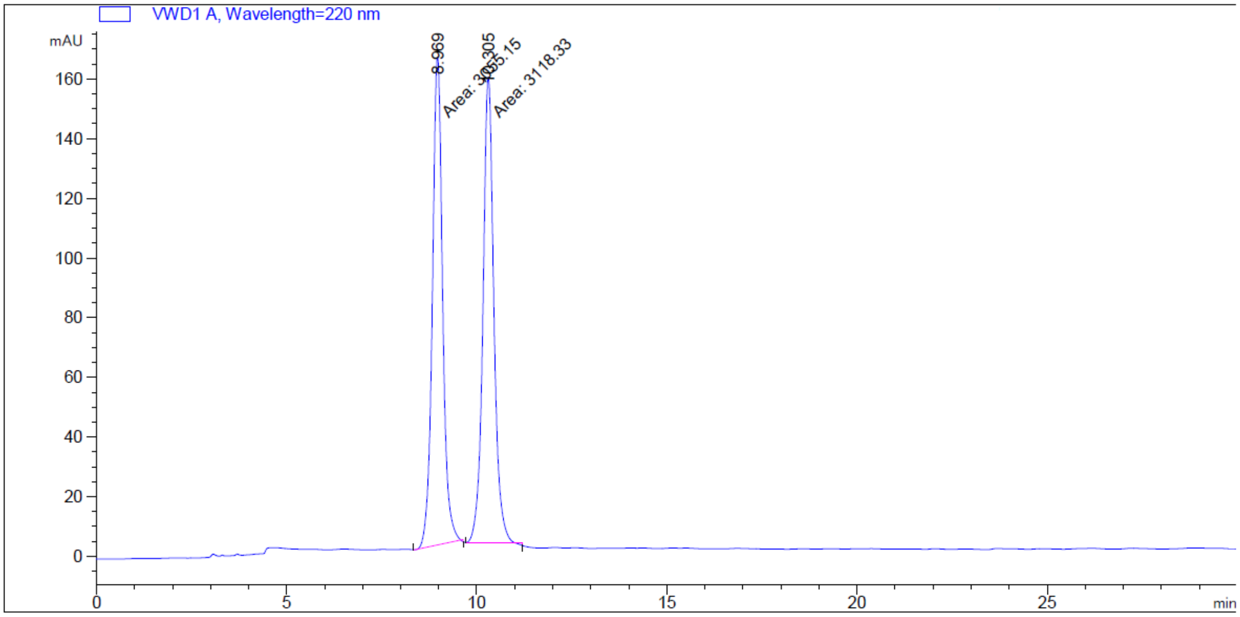

Peak #	RetTime [min]	Type	Width [min]	Area [mAU*s]	Height [mAU]	Area %
1	8.969	MM	0.3115	3055.15234	163.49059	49.4883
2	10.305	MM	0.3325	3118.33008	156.32895	50.5117

21' (*S*-isomer):

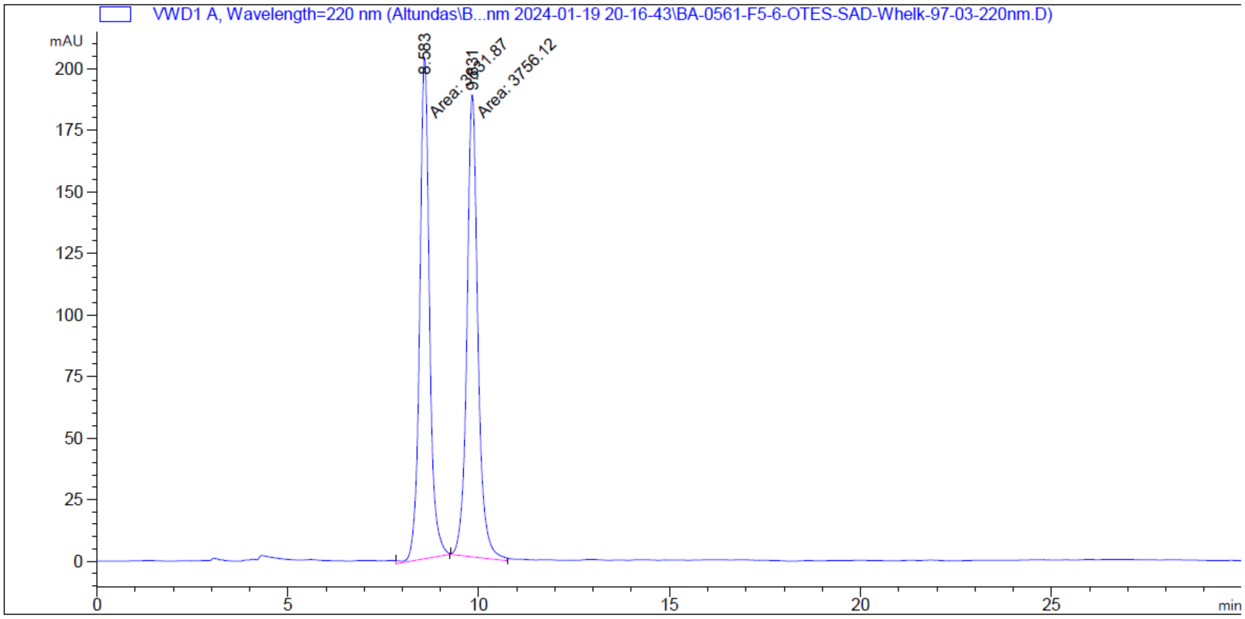

Peak #	RetTime [min]	Type	Width [min]	Area [mAU*s]	Height [mAU]	Area %
1	8.583	MM	0.2974	3631.86914	203.50970	49.1591
2	9.831	MM	0.3338	3756.11890	187.56024	50.8409

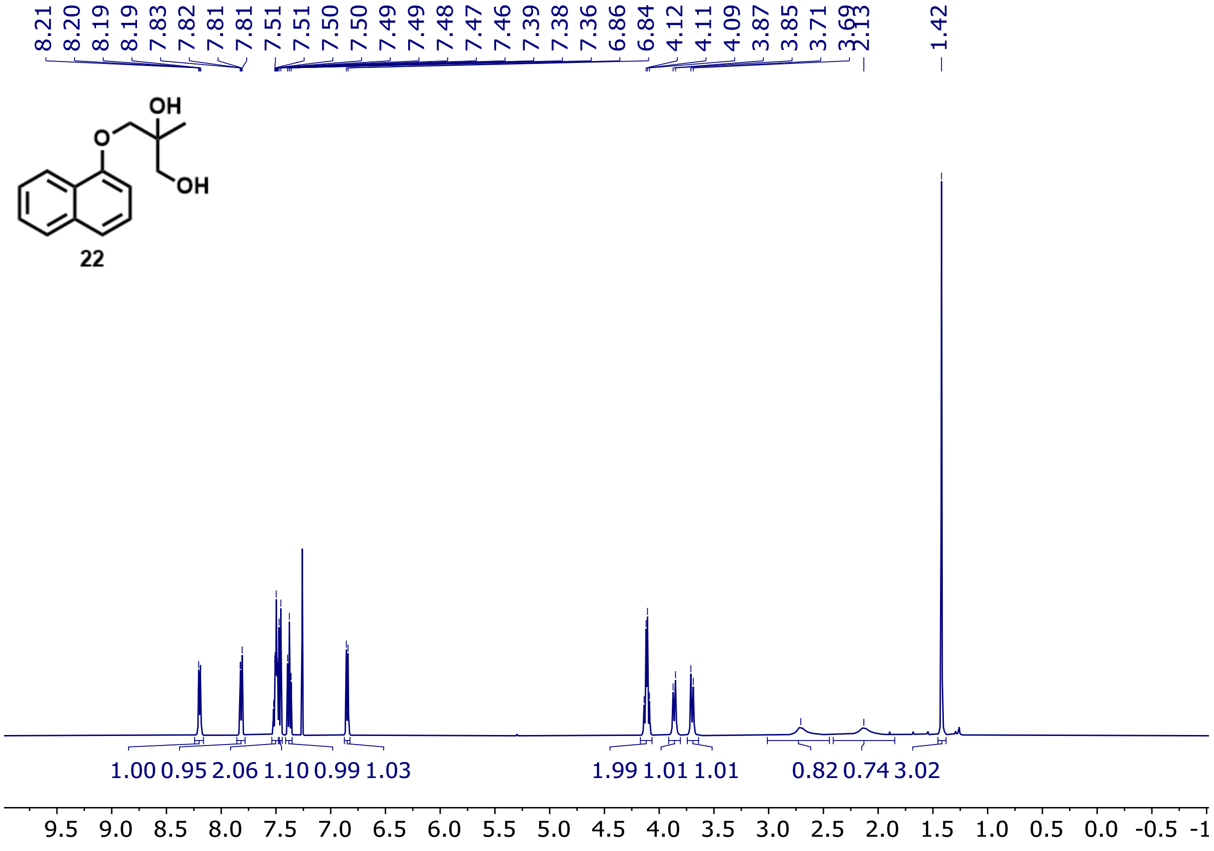
8.21 8.20 8.19 8.19 7.83 7.82 7.81 7.81 7.51 7.51 7.50 7.50 7.49 7.49 7.48 7.47 7.46 7.39 7.38 7.36 6.86 6.84 4.12 4.11 4.09 3.87 3.85 3.71 3.69 2.13 1.42
OH
O
OH
22
1.00 0.95 2.06 1.10 0.99 1.03
1.99 1.01 1.01
0.82 0.74 3.02
9.5 9.0 8.5 8.0 7.5 7.0 6.5 6.0 5.5 5.0 4.5 4.0 3.5 3.0 2.5 2.0 1.5 1.0 0.5 0.0 -0.5 -1

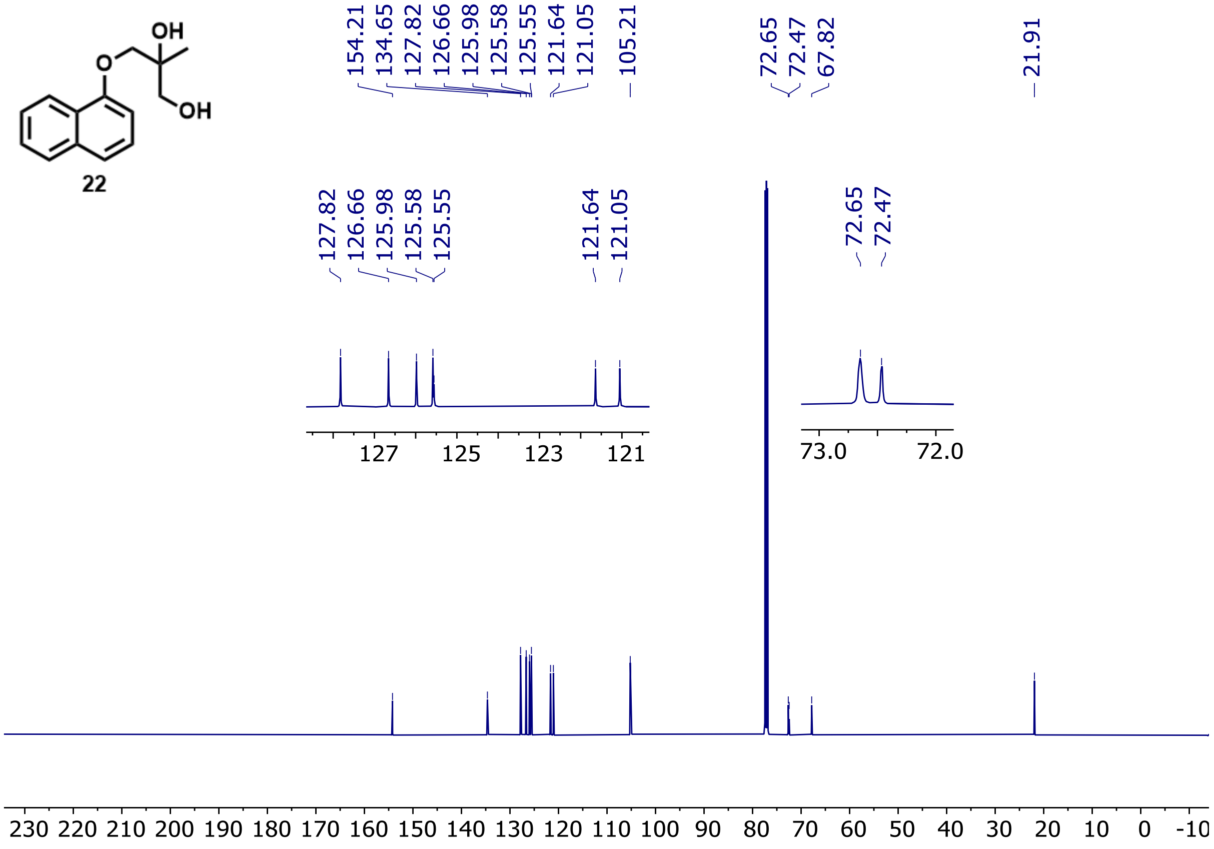
OH
O
OH
22
154.21 134.65 127.82 126.66 125.98 125.58 125.55 121.64 121.05 105.21
72.65 72.47 67.82
21.91
127.82 126.66 125.98 125.58 125.55
121.64 121.05
72.65 72.47
127 125 123 121
73.0 72.0
230 220 210 200 190 180 170 160 150 140 130 120 110 100 90 80 70 60 50 40 30 20 10 0 -10

22 (racemic):

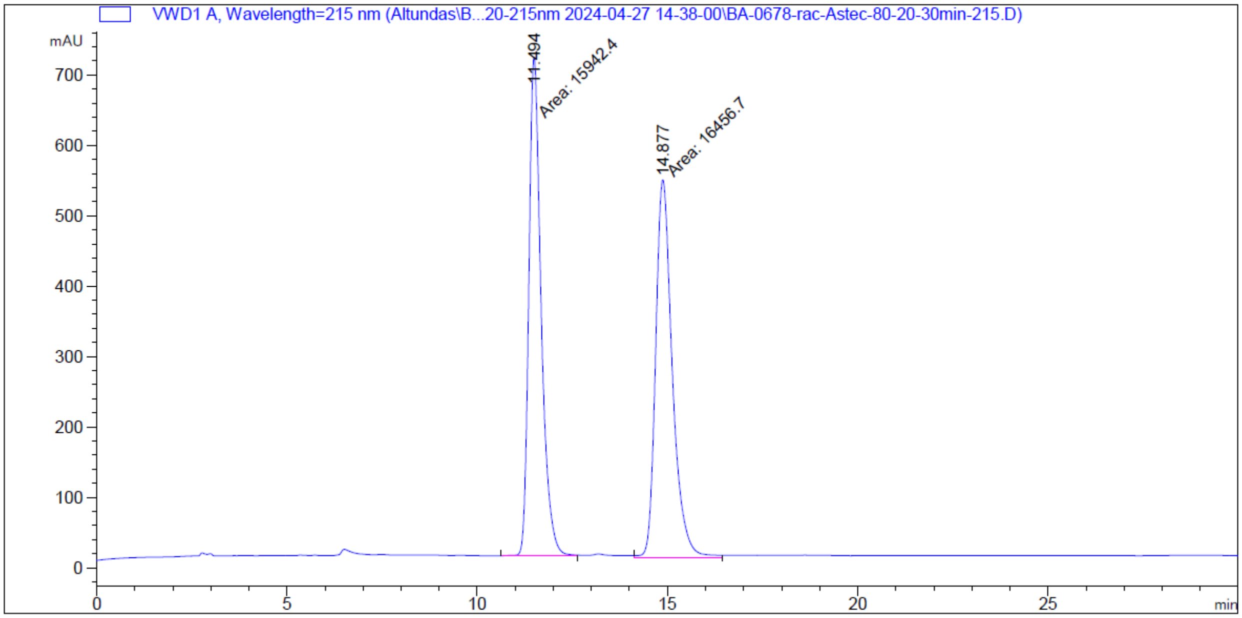

Peak #	RetTime [min]	Type	Width [min]	Area [mAU*s]	Height [mAU]	Area %
1	11.494	MM	0.3751	1.59424e4	708.39362	49.2063
2	14.877	MM	0.5104	1.64567e4	537.33362	50.7937

22' (*S*-isomer):

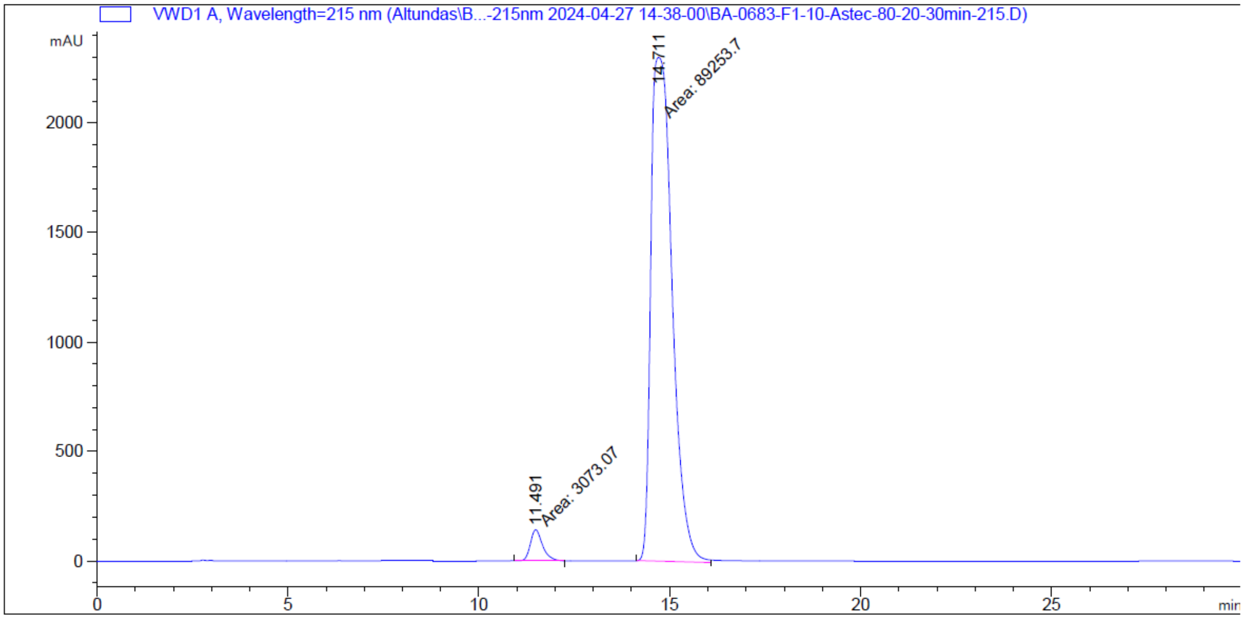

Peak #	RetTime [min]	Type	Width [min]	Area [mAU*s]	Height [mAU]	Area %
1	11.491	MM	0.3637	3073.07300	140.83028	3.3285
2	14.711	MM	0.6466	8.92537e4	2300.46167	96.6715

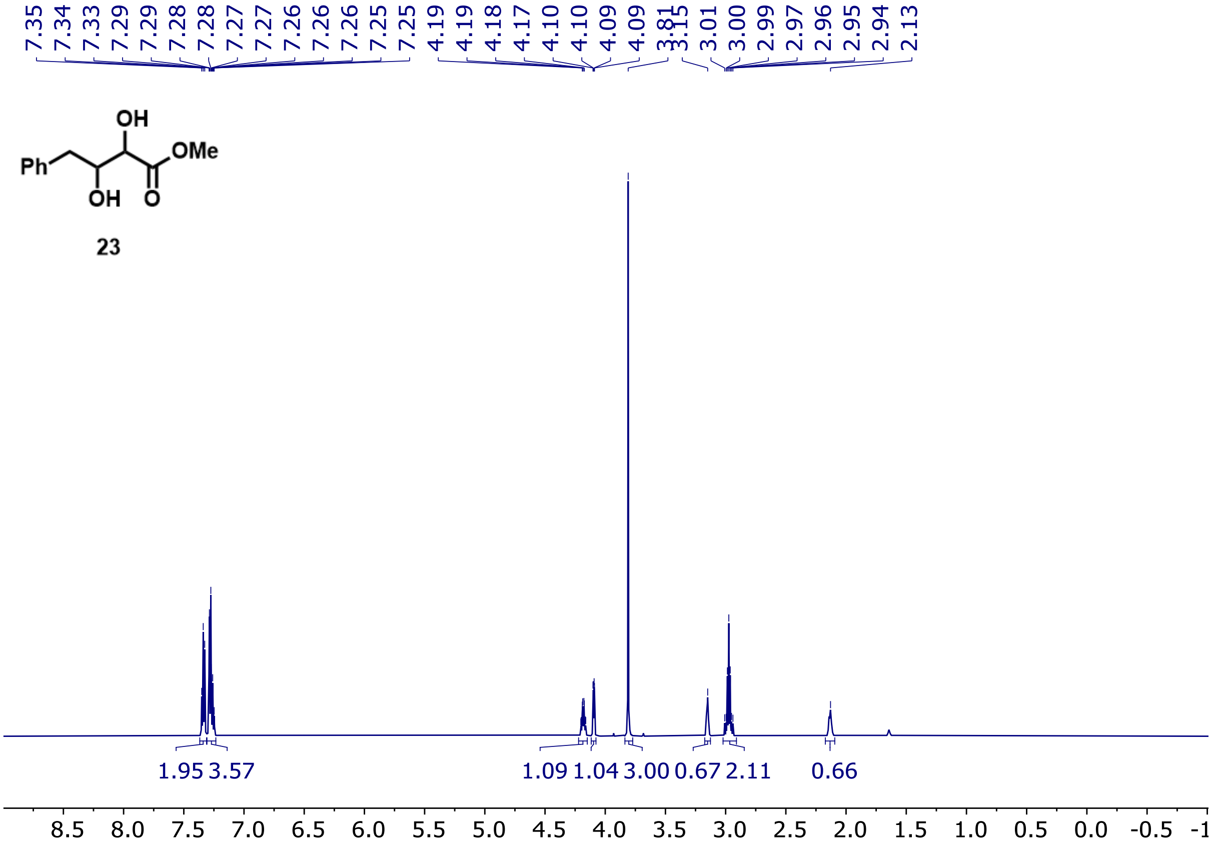
7.35 7.34 7.33 7.29 7.29 7.28 7.28 7.27 7.27 7.26 7.26 7.26 7.25 7.25 4.19 4.19 4.18 4.17 4.10 4.10 4.09 4.09 3.81 3.15 3.01 3.00 2.99 2.97 2.96 2.95 2.94 2.13
OH
OMe
Ph
OH
O
23
1.95 3.57
1.09 1.04 3.00 0.67 2.11
0.66
8.5 8.0 7.5 7.0 6.5 6.0 5.5 5.0 4.5 4.0 3.5 3.0 2.5 2.0 1.5 1.0 0.5 0.0 -0.5 -1

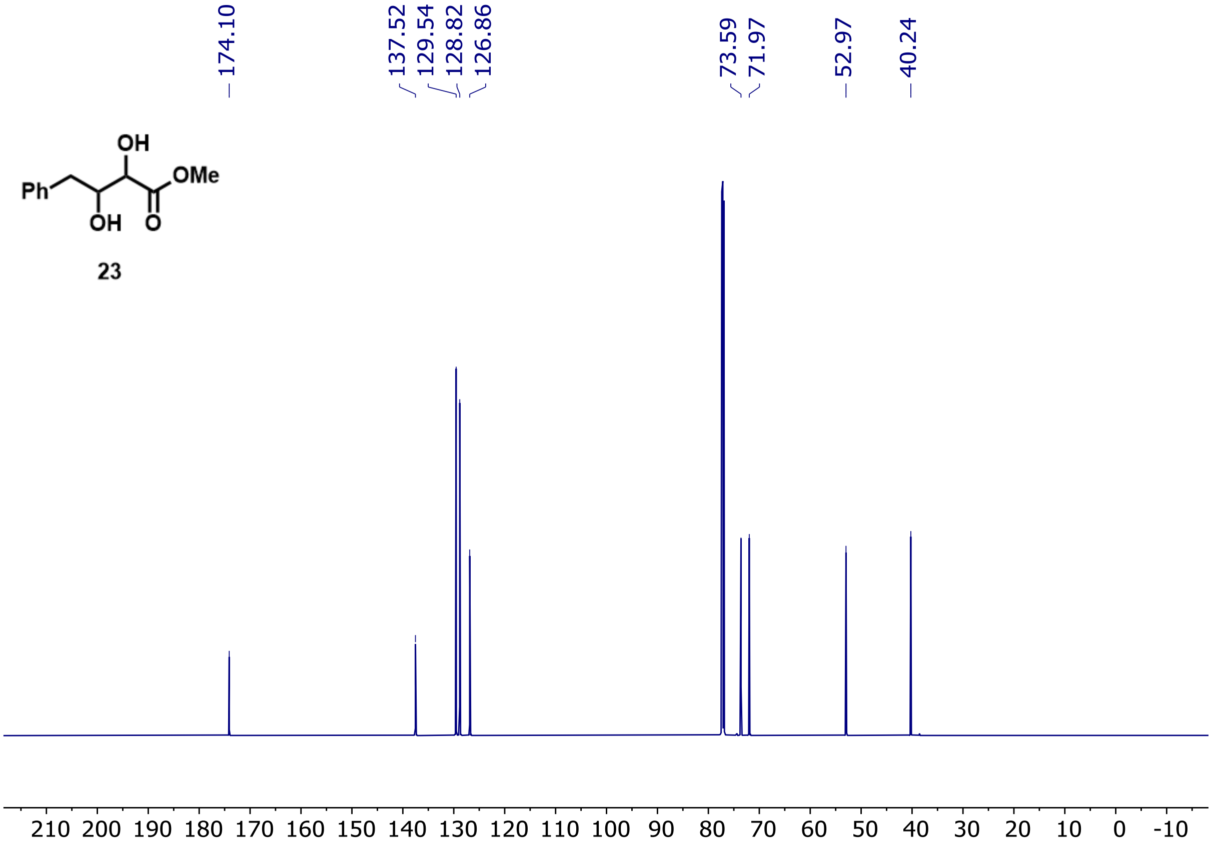
174.10
137.52 129.54 128.82 126.86
73.59 71.97
52.97
40.24
OH
OMe
Ph
OH
O
23
210 200 190 180 170 160 150 140 130 120 110 100 90 80 70 60 50 40 30 20 10 0 -10

23 (racemic):

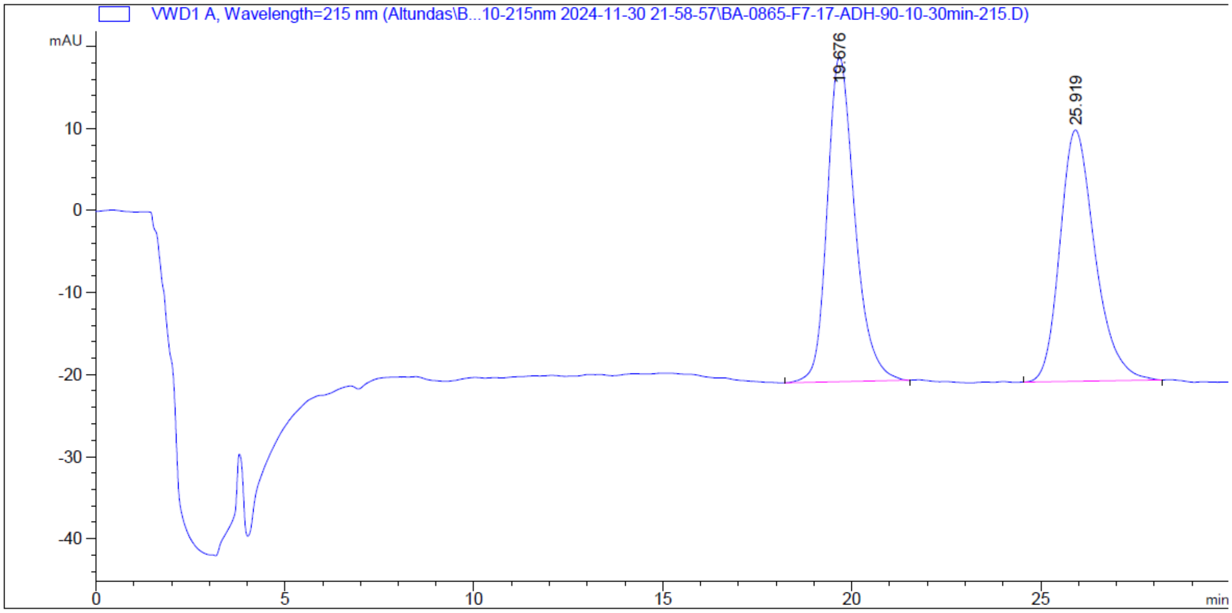

Peak #	RetTime [min]	Type	Width [min]	Area [mAU*s]	Height [mAU]	Area %
1	19.676	BB	0.7749	2006.71289	39.58652	50.3920
2	25.919	BB	0.9794	1975.49146	30.63620	49.6080

23' (RS-isomer):

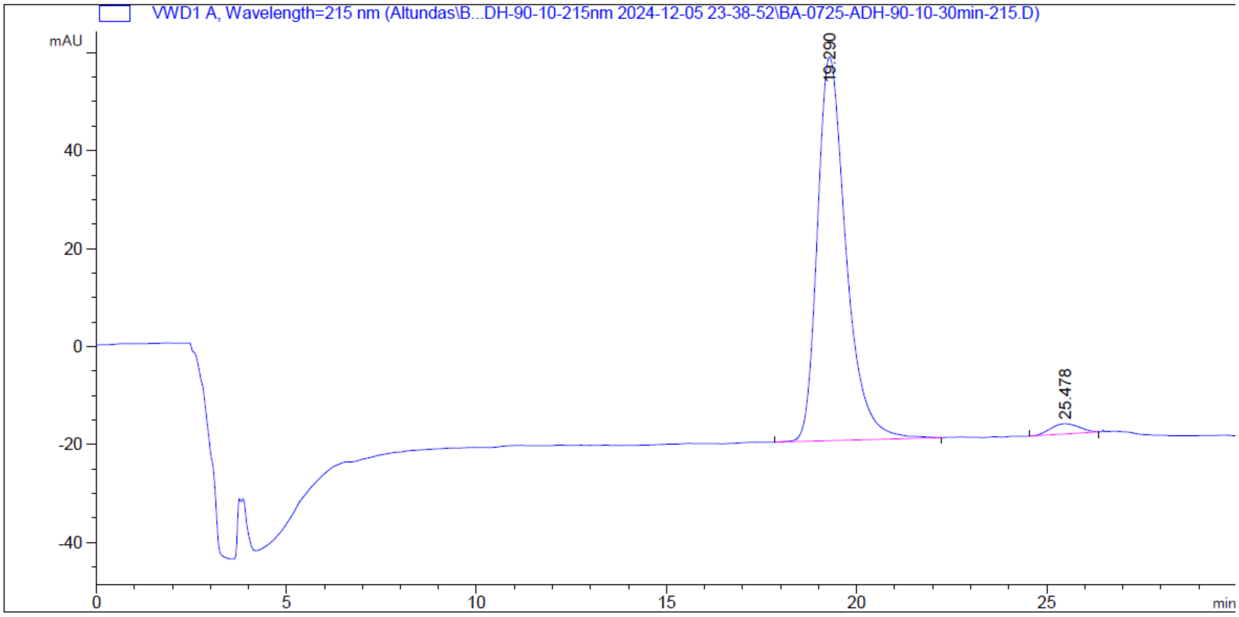

Peak #	RetTime [min]	Type	Width [min]	Area [mAU*s]	Height [mAU]	Area %
1	19.290	BB	0.7962	4104.09912	78.32807	97.4691
2	25.478	BB	0.6207	106.56991	2.04131	2.5309

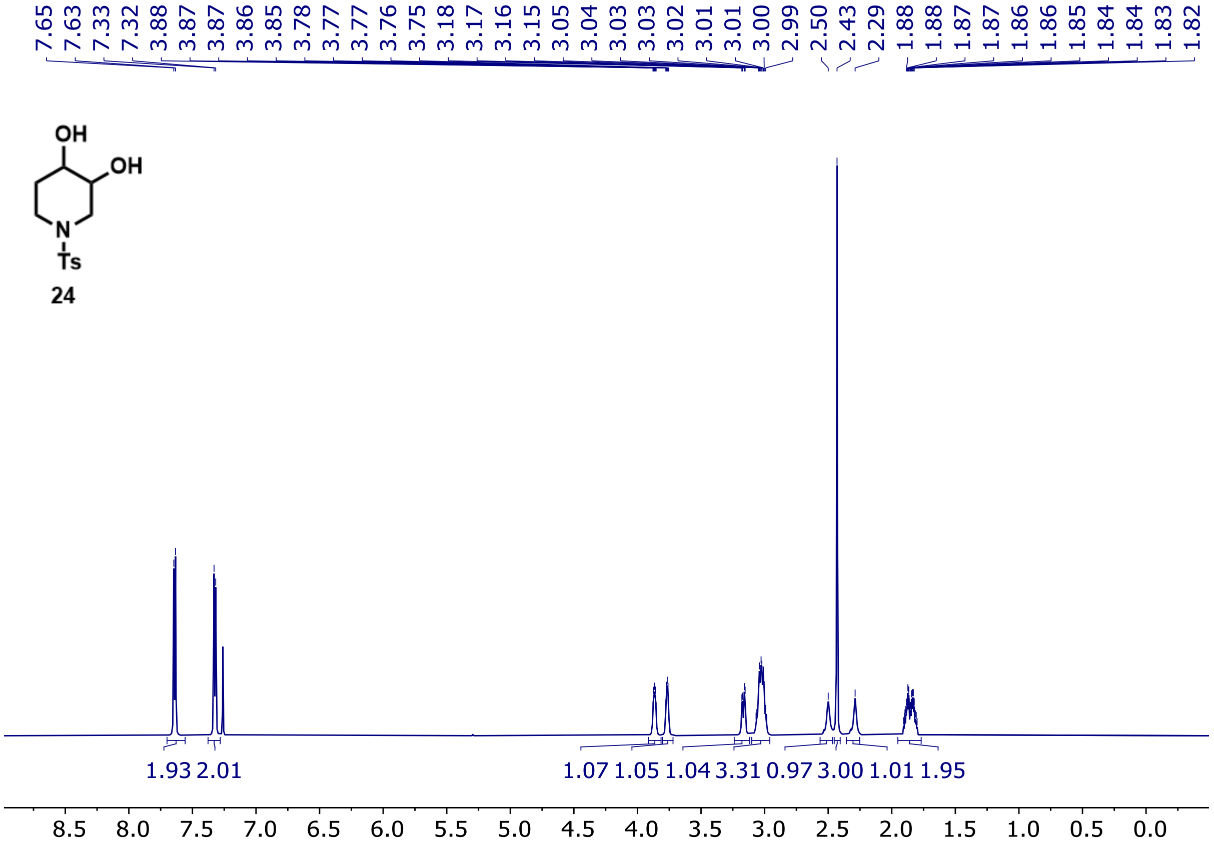
7.65 7.63 7.33 7.32 3.88 3.87 3.87 3.86 3.85 3.78 3.77 3.77 3.76 3.75 3.18 3.17 3.16 3.15 3.05 3.04 3.03 3.03 3.02 3.01 3.01 3.00 2.99 2.50 2.43 2.29 1.88 1.88 1.87 1.87 1.86 1.86 1.85 1.84 1.84 1.83 1.82
OH
OH
N
Ts
24
1.93 2.01
1.07 1.05 1.04 3.31 0.97 3.00 1.01 1.95
8.5 8.0 7.5 7.0 6.5 6.0 5.5 5.0 4.5 4.0 3.5 3.0 2.5 2.0 1.5 1.0 0.5 0.0

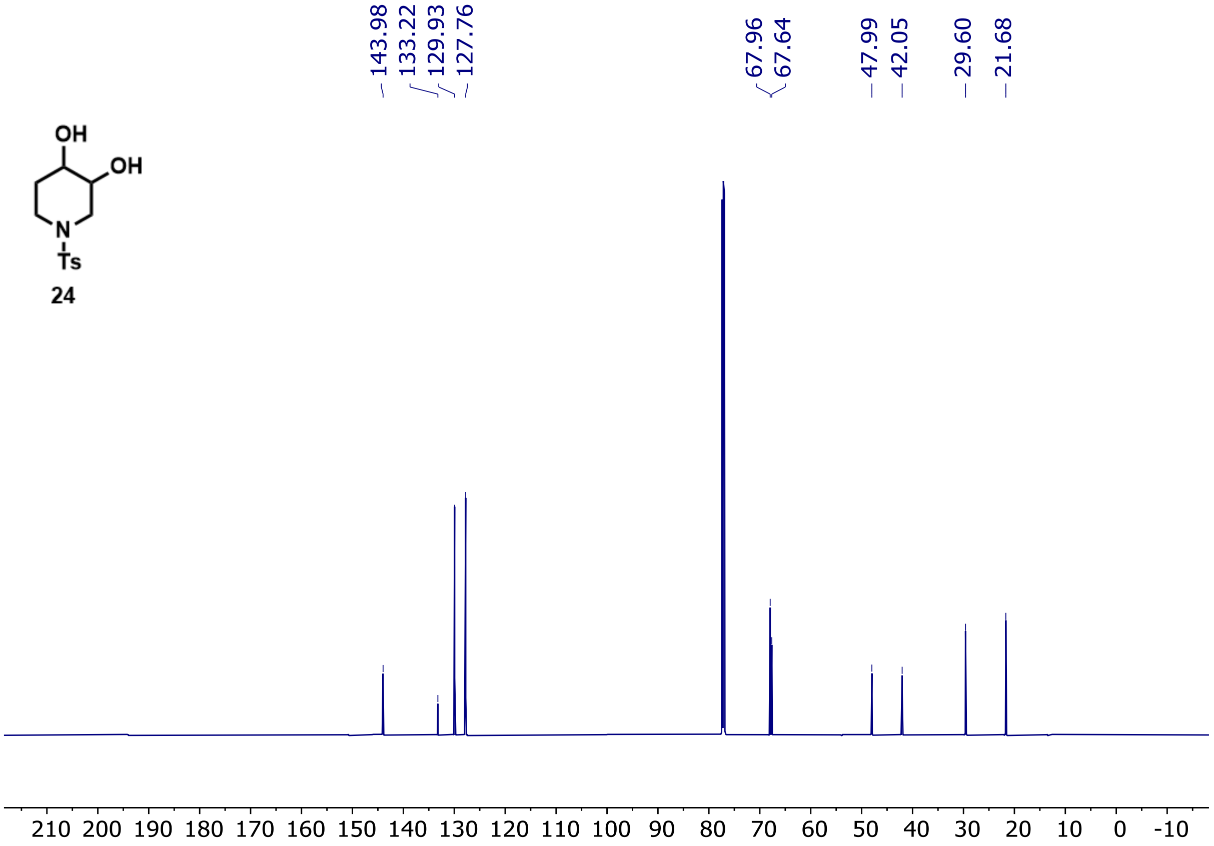
143.98 133.22 129.93 127.76
67.96 67.64
47.99 42.05
29.60 21.68
OH
OH
N
Ts
24
210 200 190 180 170 160 150 140 130 120 110 100 90 80 70 60 50 40 30 20 10 0 -10

24 (racemic):

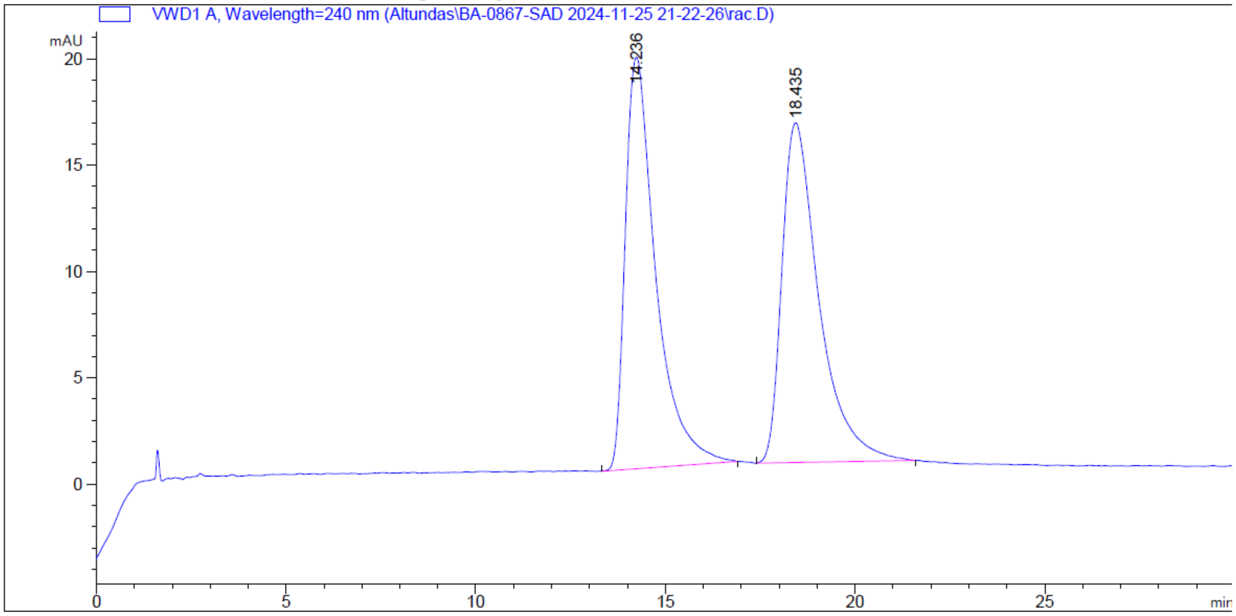

Peak #	RetTime [min]	Type	Width [min]	Area [mAU*s]	Height [mAU]	Area %
1	14.236	BB	0.8215	1080.05457	19.35264	49.9394
2	18.435	BB	0.9826	1082.67639	15.98130	50.0606

24' (*3S*, *4R* – isomer):

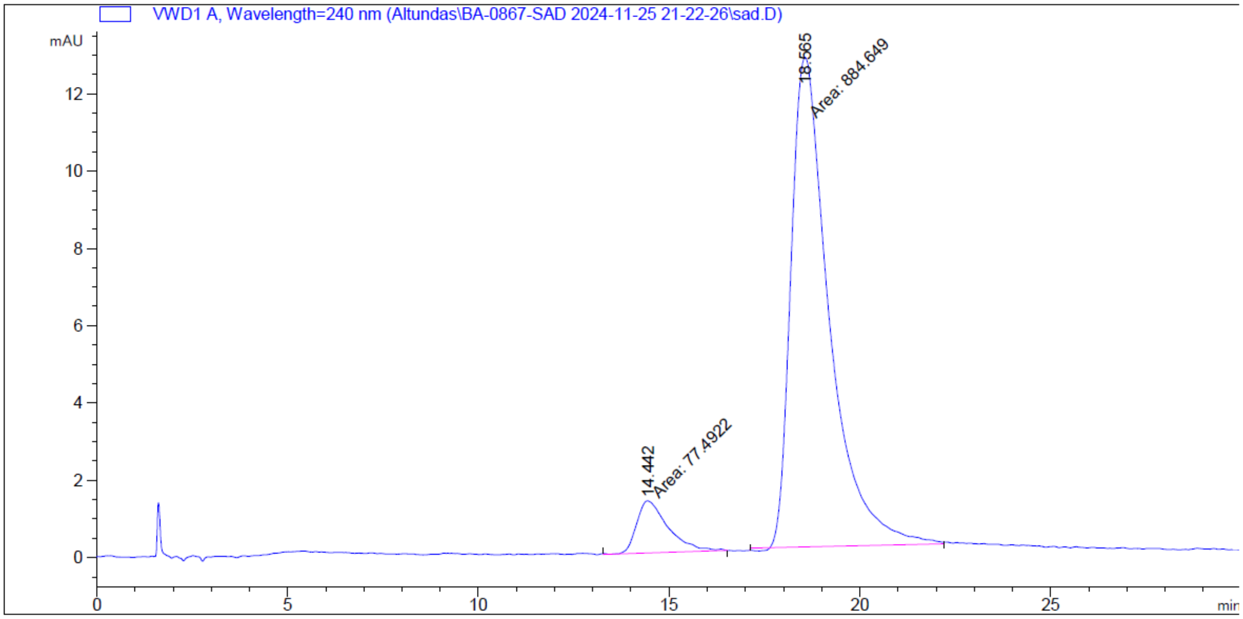

Peak #	RetTime [min]	Type	Width [min]	Area [mAU*s]	Height [mAU]	Area %
1	14.442	MM	0.9549	77.49216	1.35249	8.0541
2	18.565	MM	1.1636	884.64874	12.67147	91.9459

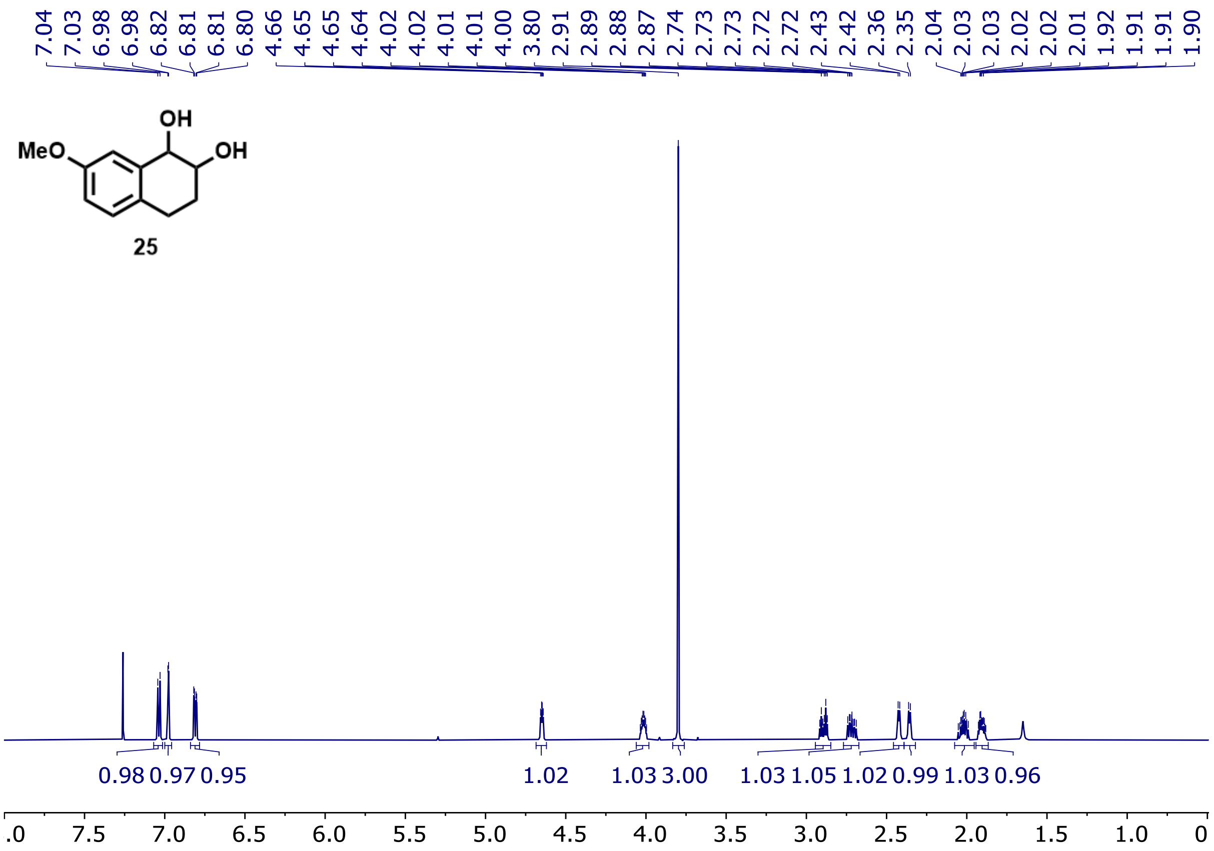
7.04 7.03 6.98 6.98 6.82 6.81 6.81 6.80 4.66 4.65 4.65 4.64 4.02 4.02 4.01 4.01 4.00 3.80 2.91 2.89 2.88 2.87 2.74 2.73 2.73 2.72 2.72 2.43 2.42 2.36 2.35 2.04 2.03 2.03 2.02 2.02 2.01 1.92 1.91 1.91 1.90
OH
MeO
OH
25
0.98 0.97 0.95 1.02 1.03 3.00 1.03 1.05 1.02 0.99 1.03 0.96
.0 7.5 7.0 6.5 6.0 5.5 5.0 4.5 4.0 3.5 3.0 2.5 2.0 1.5 1.0 0

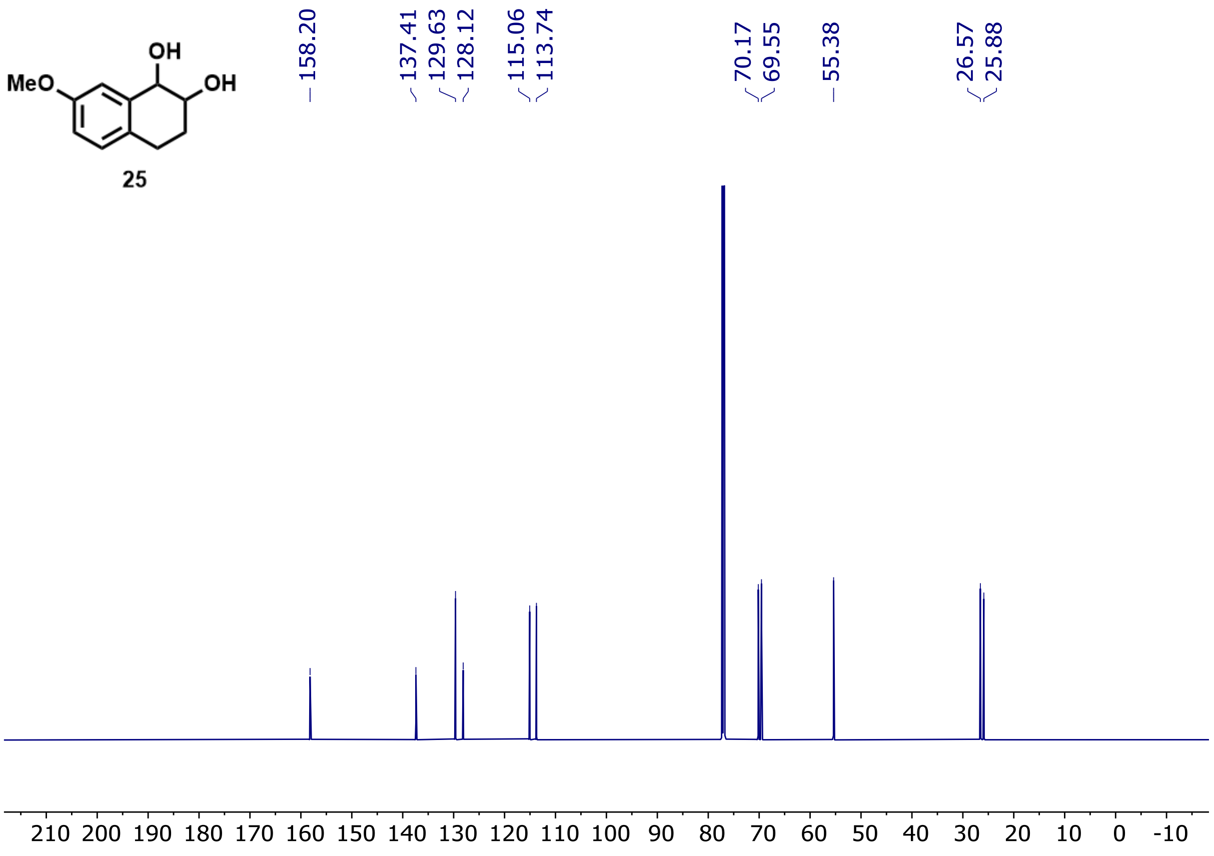
158.20 137.41 129.63 128.12 115.06 113.74 70.17 69.55 55.38 26.57 25.88
OH
MeO
OH
25
210 200 190 180 170 160 150 140 130 120 110 100 90 80 70 60 50 40 30 20 10 0 -10

25 (racemic):

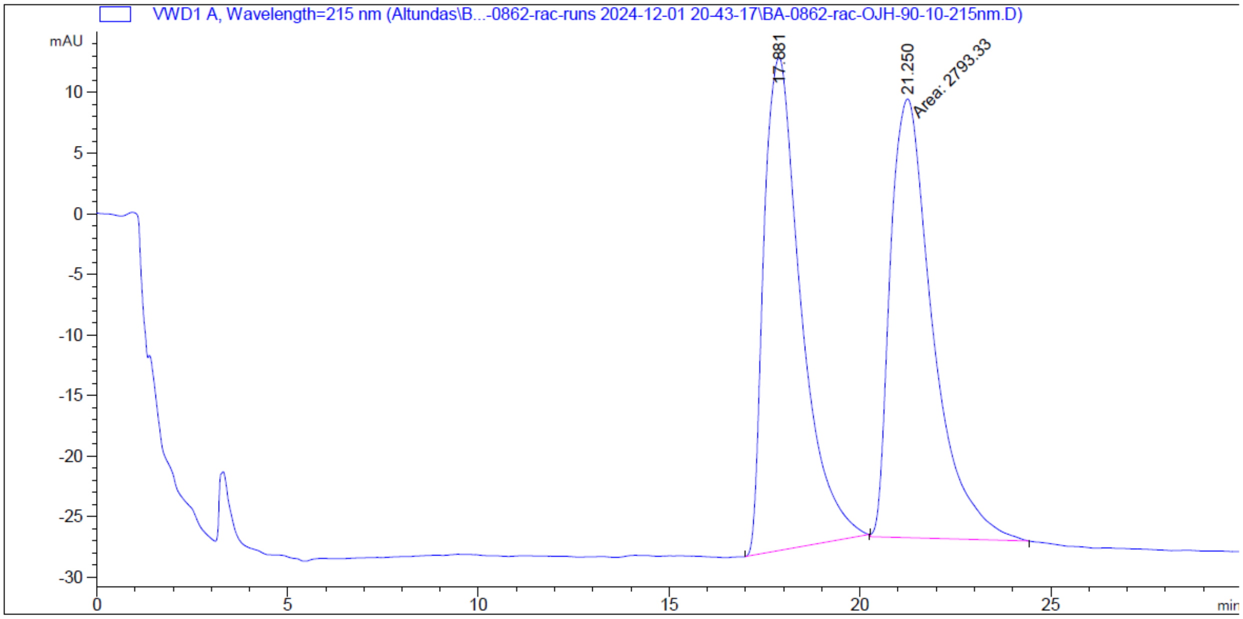

Peak #	RetTime [min]	Type	Width [min]	Area [mAU*s]	Height [mAU]	Area %
1	17.881	BB	1.0223	2747.91650	40.69059	49.5902
2	21.250	MM	1.2860	2793.32983	36.20069	50.4098

25' (*1R*, *2S* – isomer):

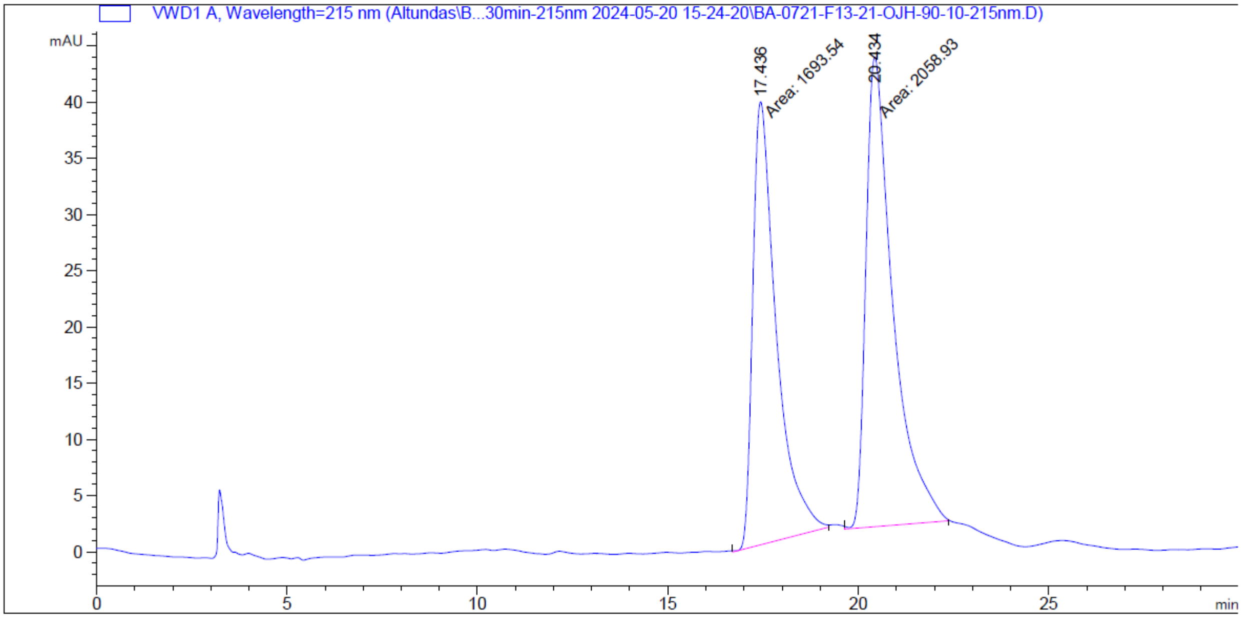

Peak #	RetTime [min]	Type	Width [min]	Area [mAU*s]	Height [mAU]	Area %
1	17.436	MM	0.7164	1693.54285	39.40011	45.1314
2	20.434	MM	0.8218	2058.92773	41.75536	54.8686

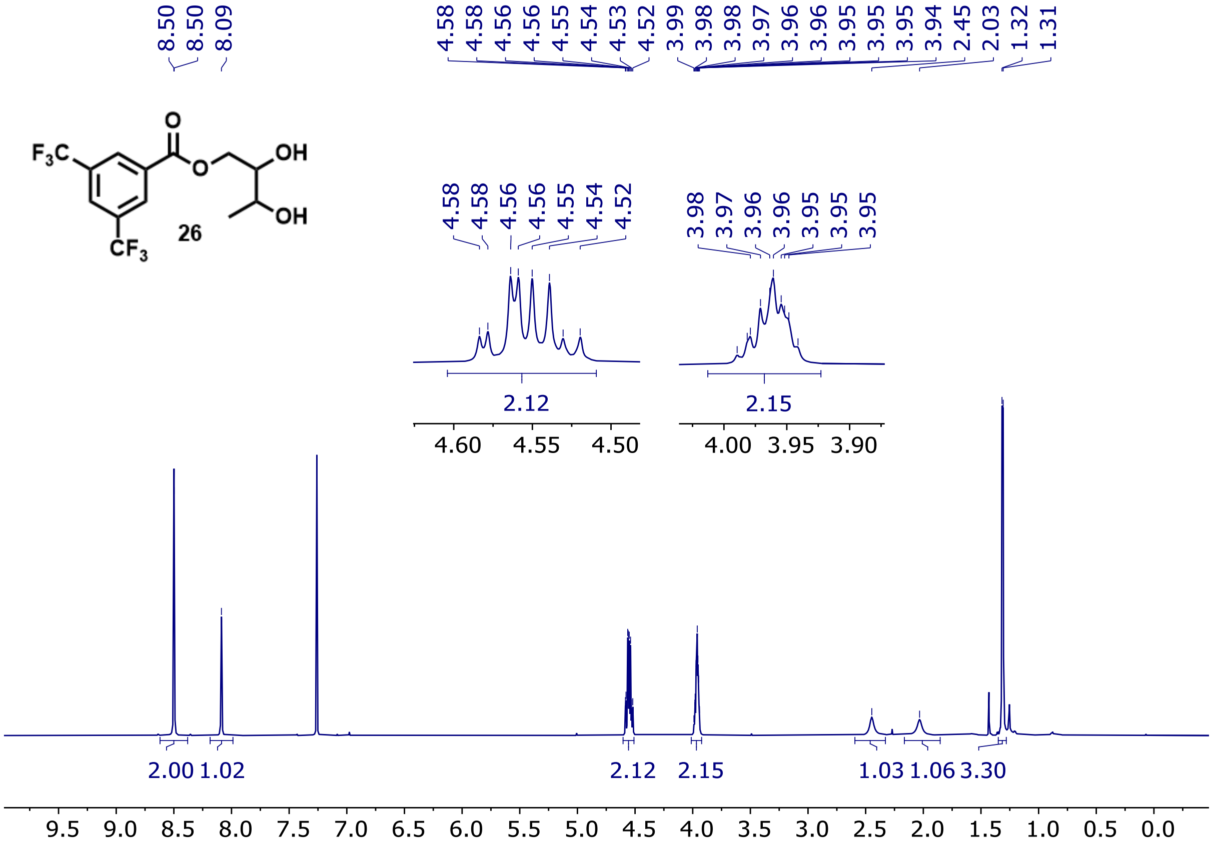
8.50 8.50 8.09
4.58 4.58 4.56 4.56 4.55 4.54 4.53 4.52 3.99 3.98 3.98 3.97 3.96 3.96 3.95 3.95 3.95 3.94 2.45 2.03 1.32 1.31
F3C
O
O
OH
OH
CF3
26
4.58 4.58 4.56 4.56 4.55 4.54 4.52
3.98 3.97 3.96 3.96 3.95 3.95 3.95
2.12
2.15
4.60 4.55 4.50
4.00 3.95 3.90
2.00 1.02 2.12 2.15 1.03 1.06 3.30
9.5 9.0 8.5 8.0 7.5 7.0 6.5 6.0 5.5 5.0 4.5 4.0 3.5 3.0 2.5 2.0 1.5 1.0 0.5 0.0

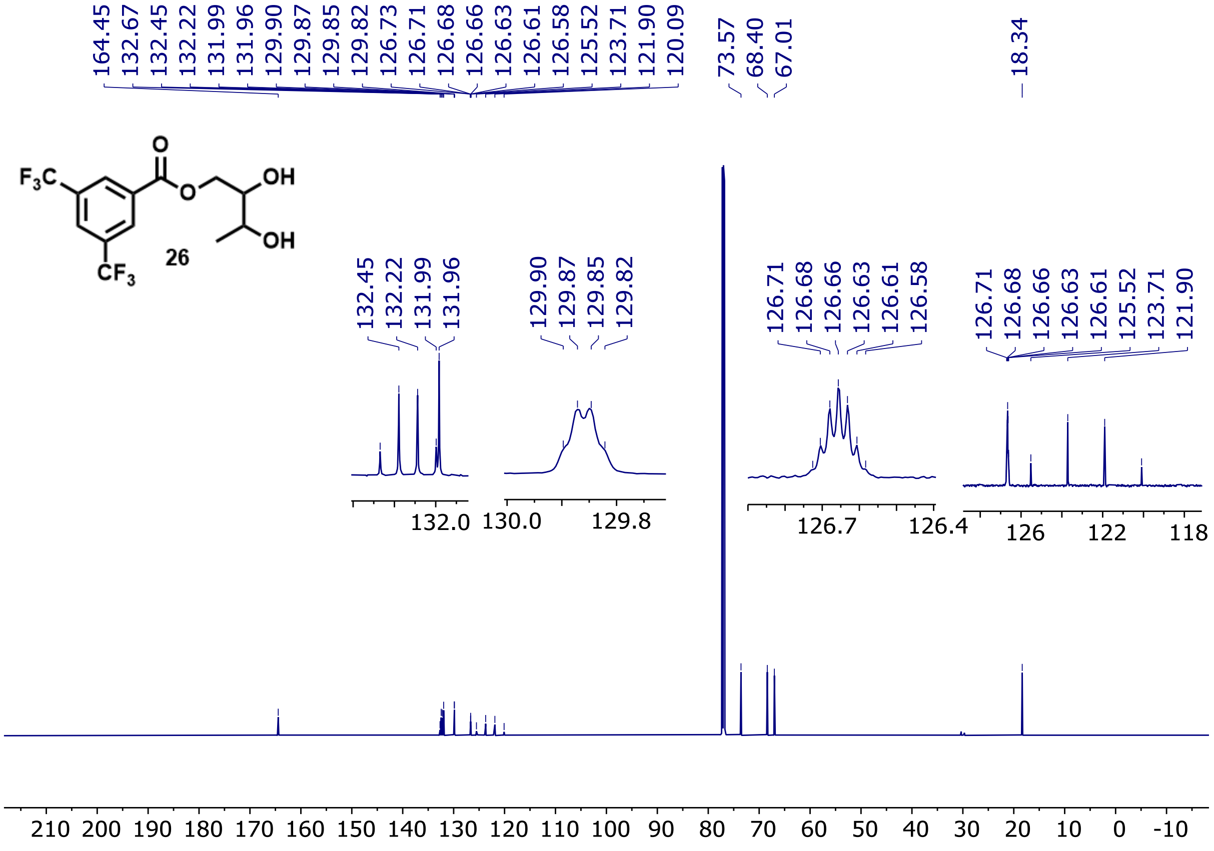
164.45 132.67 132.45 132.22 131.99 131.96 129.90 129.87 129.85 129.82 126.73 126.71 126.68 126.66 126.63 126.61 126.58 125.52 123.71 121.90 120.09
73.57 68.40 67.01
18.34
F3C
O
O
OH
OH
CF3
26
132.45 132.22 131.99 131.96
129.90 129.87 129.85 129.82
126.71 126.68 126.66 126.63 126.61 126.58
126.71 126.68 126.66 126.63 126.61 125.52 123.71 121.90
132.0 130.0 129.8
126.7 126.4
126 122 118
210 200 190 180 170 160 150 140 130 120 110 100 90 80 70 60 50 40 30 20 10 0 -10

26 (racemic):

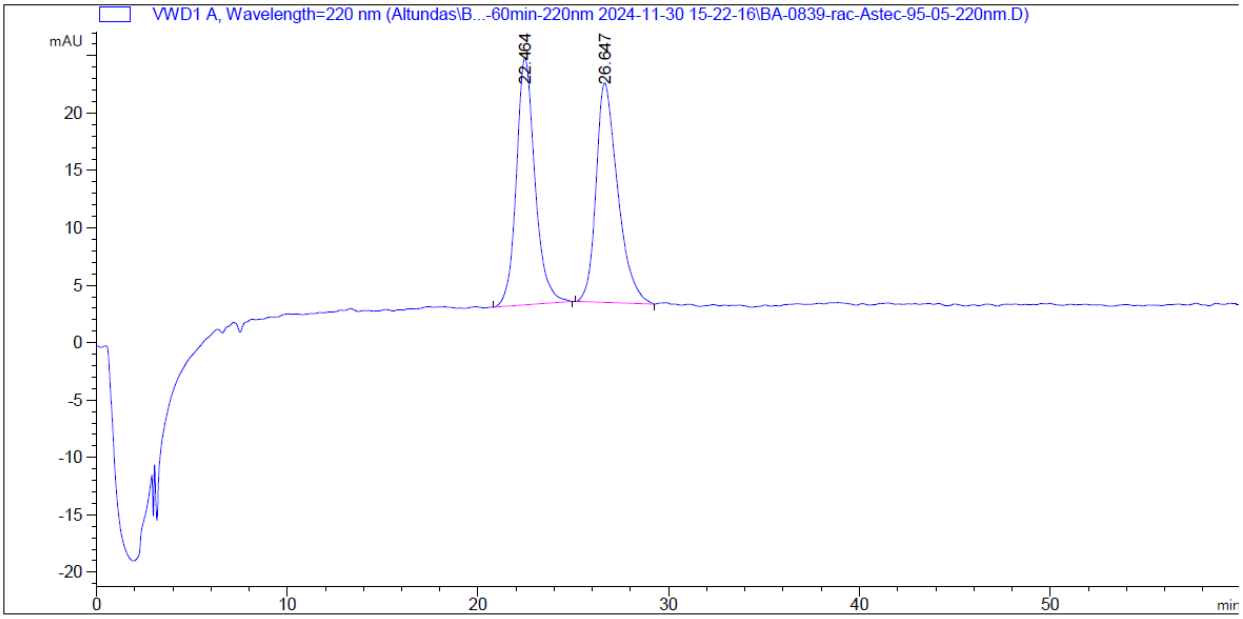

Peak #	RetTime [min]	Type	Width [min]	Area [mAU*s]	Height [mAU]	Area %
1	22.464	BB	1.0280	1460.09009	21.54436	49.1486
2	26.647	BB	1.1245	1510.67712	19.11266	50.8514

26' (*2R*, *3S*-isomer):

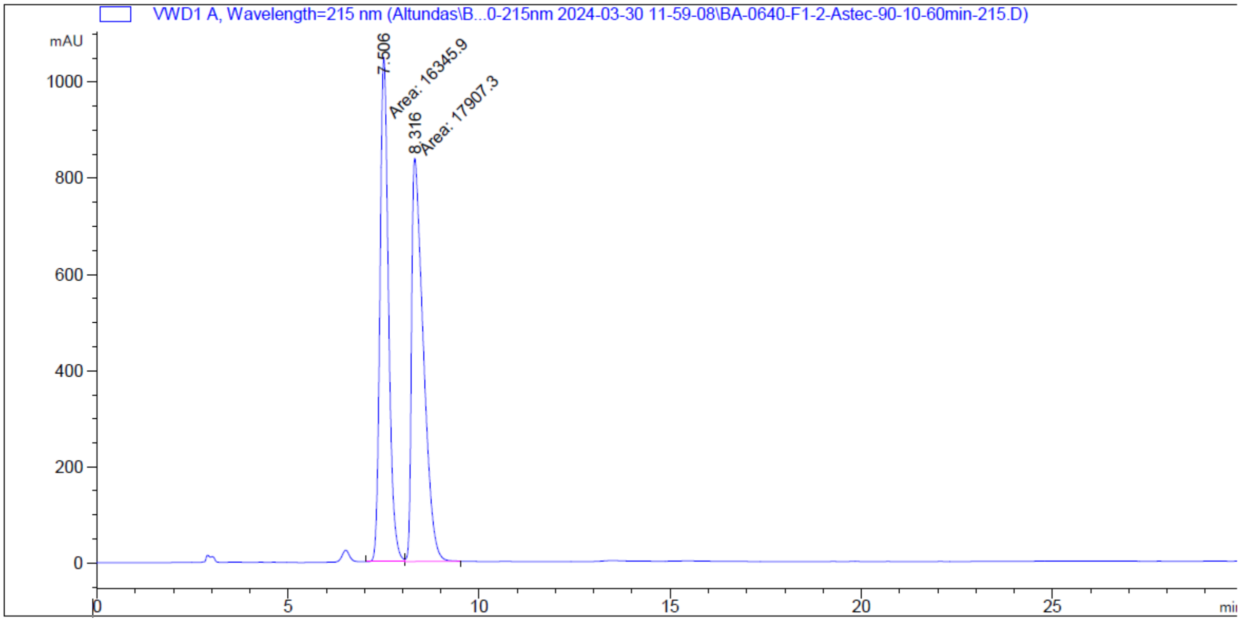

Peak #	RetTime [min]	Type	Width [min]	Area [mAU*s]	Height [mAU]	Area %
1	7.506	MF	0.2599	1.63459e4	1048.11902	47.7208
2	8.316	FM	0.3560	1.79073e4	838.37433	52.2792

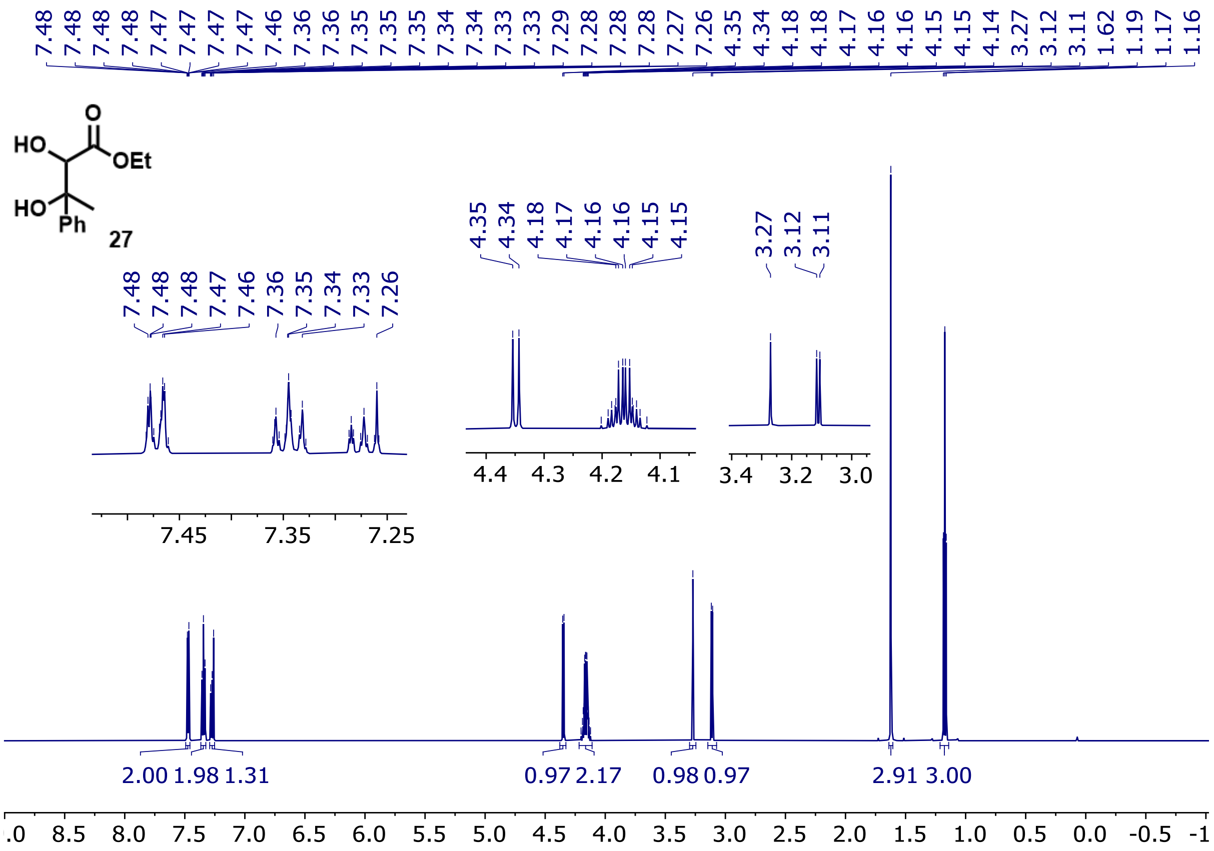
7.48 7.48 7.48 7.48 7.47 7.47 7.47 7.47 7.46 7.36 7.36 7.35 7.35 7.35 7.34 7.34 7.33 7.33 7.29 7.28 7.28 7.28 7.27 7.26 4.35 4.34 4.18 4.18 4.17 4.16 4.16 4.15 4.15 4.14 3.27 3.12 3.11 1.62 1.19 1.17 1.16
HO
O
OEt
HO
Ph
27
7.48 7.48 7.48 7.47 7.46 7.36 7.35 7.34 7.33 7.26
7.45 7.35 7.25
4.35 4.34 4.18 4.17 4.16 4.16 4.15 4.15
4.4 4.3 4.2 4.1
3.27 3.12 3.11
3.4 3.2 3.0
2.00 1.98 1.31
0.97 2.17
0.98 0.97
2.91 3.00
.0 8.5 8.0 7.5 7.0 6.5 6.0 5.5 5.0 4.5 4.0 3.5 3.0 2.5 2.0 1.5 1.0 0.5 0.0 -0.5 -1

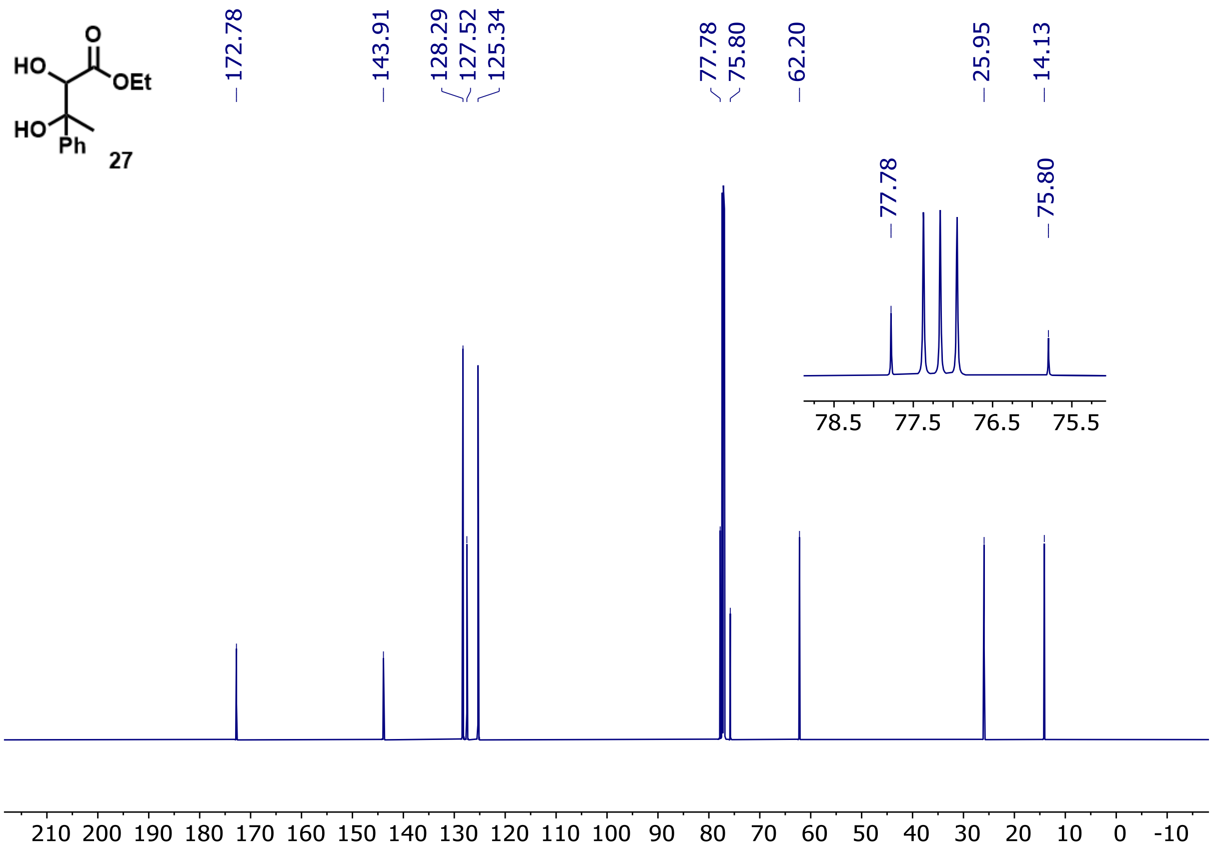
172.78
143.91
128.29 127.52 125.34
77.78 75.80
62.20
25.95
14.13
HO
O
OEt
HO
Ph
27
77.78
75.80
78.5 77.5 76.5 75.5
210 200 190 180 170 160 150 140 130 120 110 100 90 80 70 60 50 40 30 20 10 0 -10

27 (racemic):

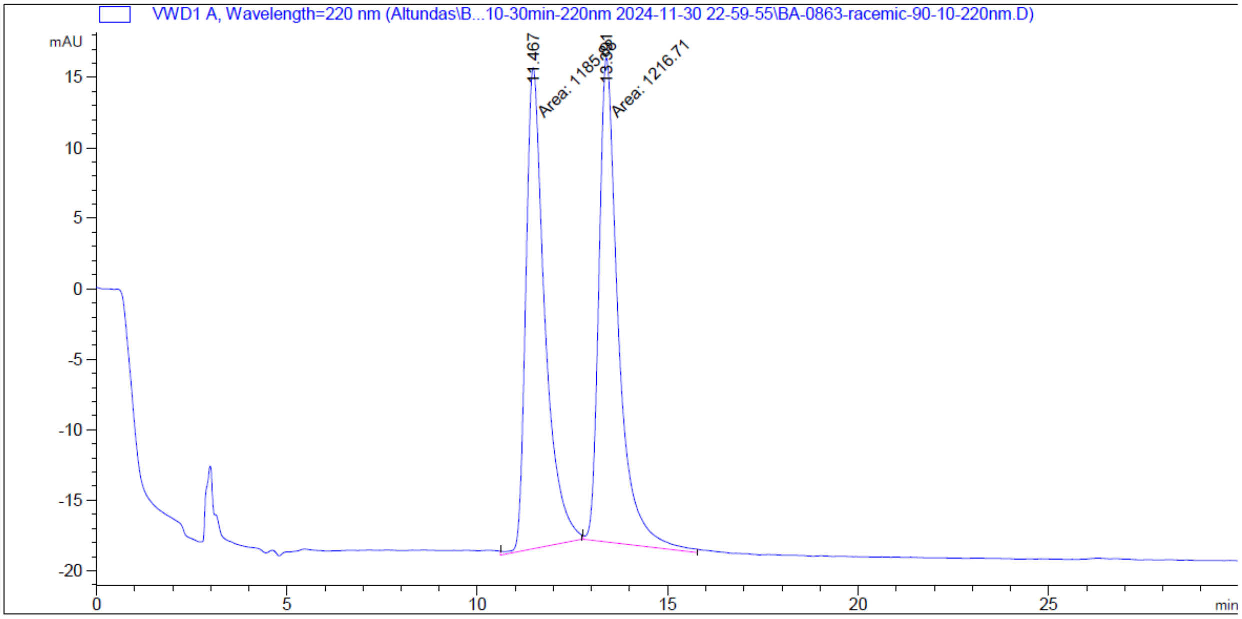

Peak #	RetTime [min]	Type	Width [min]	Area [mAU*s]	Height [mAU]	Area %
1	11.467	MM	0.5790	1185.88123	34.13375	49.3584
2	13.391	MM	0.5904	1216.71057	34.34635	50.6416

27' (*2S*, *3R* – isomer):

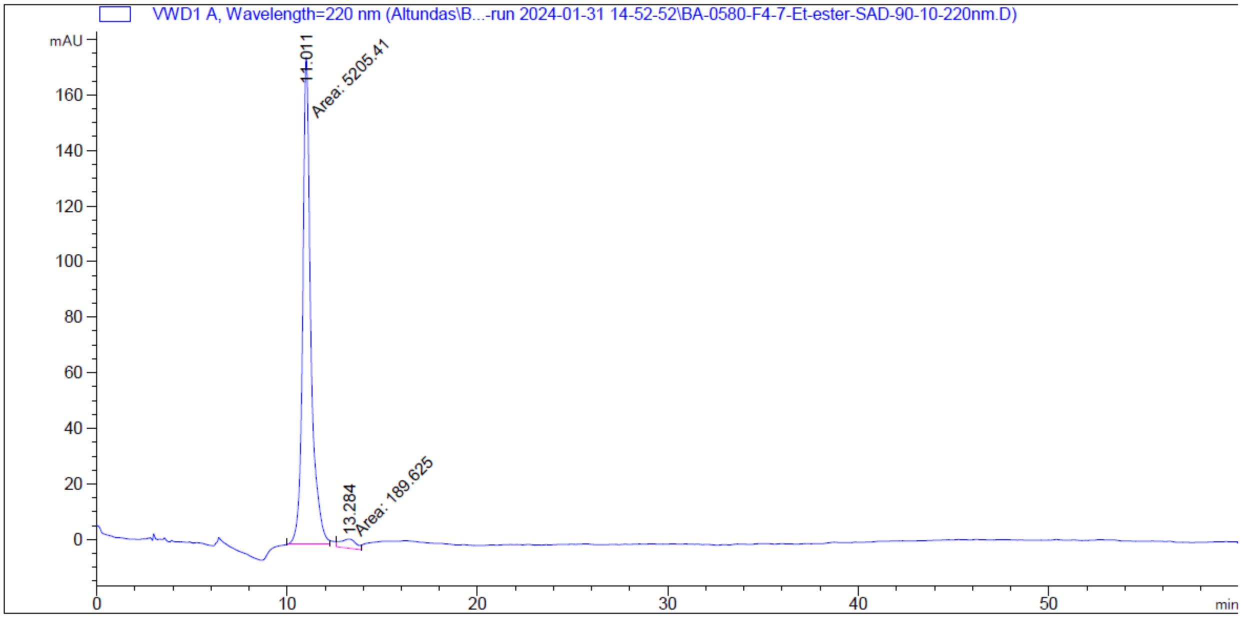

Peak #	RetTime [min]	Type	Width [min]	Area [mAU*s]	Height [mAU]	Area %
1	11.011	MM	0.4955	5205.41016	175.07376	96.4852
2	13.284	MM	0.9547	189.62486	3.31032	3.5148

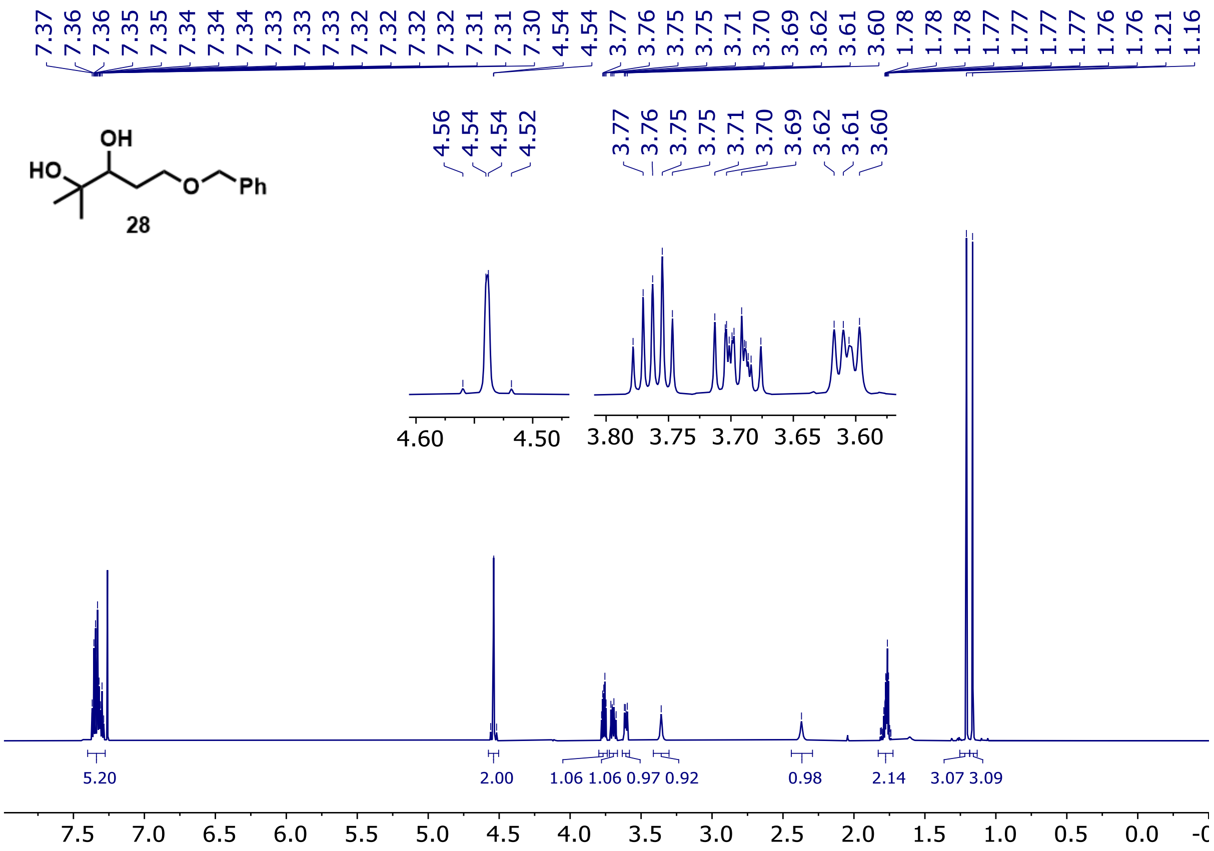
7.37 7.36 7.36 7.35 7.35 7.34 7.34 7.34 7.33 7.33 7.33 7.32 7.32 7.32 7.32 7.31 7.31 7.30 4.54 4.54 3.77 3.76 3.75 3.75 3.71 3.70 3.69 3.62 3.61 3.60 1.78 1.78 1.78 1.77 1.77 1.77 1.77 1.76 1.76 1.21 1.16
4.56 4.54 4.54 4.52
3.77 3.76 3.75 3.75 3.71 3.70 3.69 3.62 3.61 3.60
OH
HO
O
Ph
28
4.60 4.50
3.80 3.75 3.70 3.65 3.60
5.20 2.00 1.06 1.06 0.97 0.92 0.98 2.14 3.07 3.09
7.5 7.0 6.5 6.0 5.5 5.0 4.5 4.0 3.5 3.0 2.5 2.0 1.5 1.0 0.5 0.0

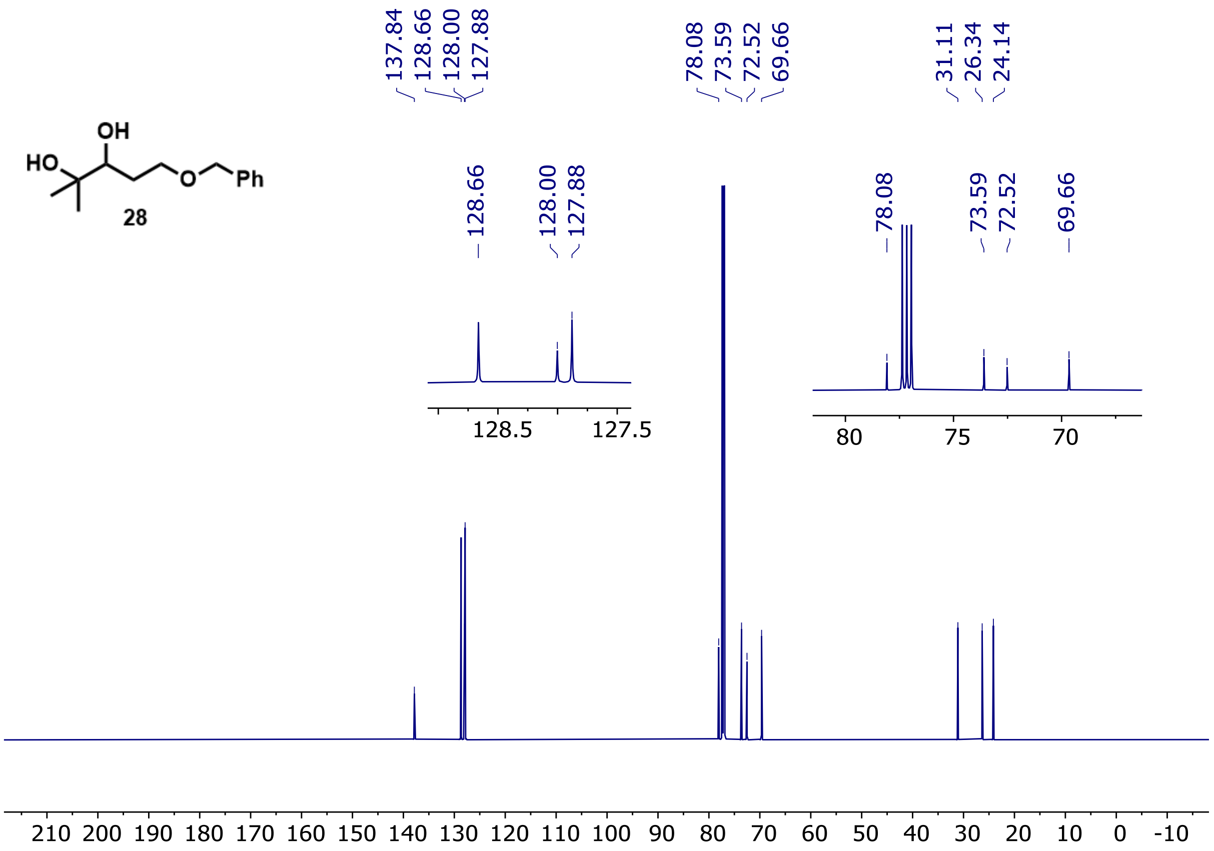
137.84 128.66 128.00 127.88
78.08 73.59 72.52 69.66
31.11 26.34 24.14
OH
HO
O
Ph
28
128.66 128.00 127.88
128.5 127.5
78.08 73.59 72.52 69.66
80 75 70
210 200 190 180 170 160 150 140 130 120 110 100 90 80 70 60 50 40 30 20 10 0 -10

28 (racemic):

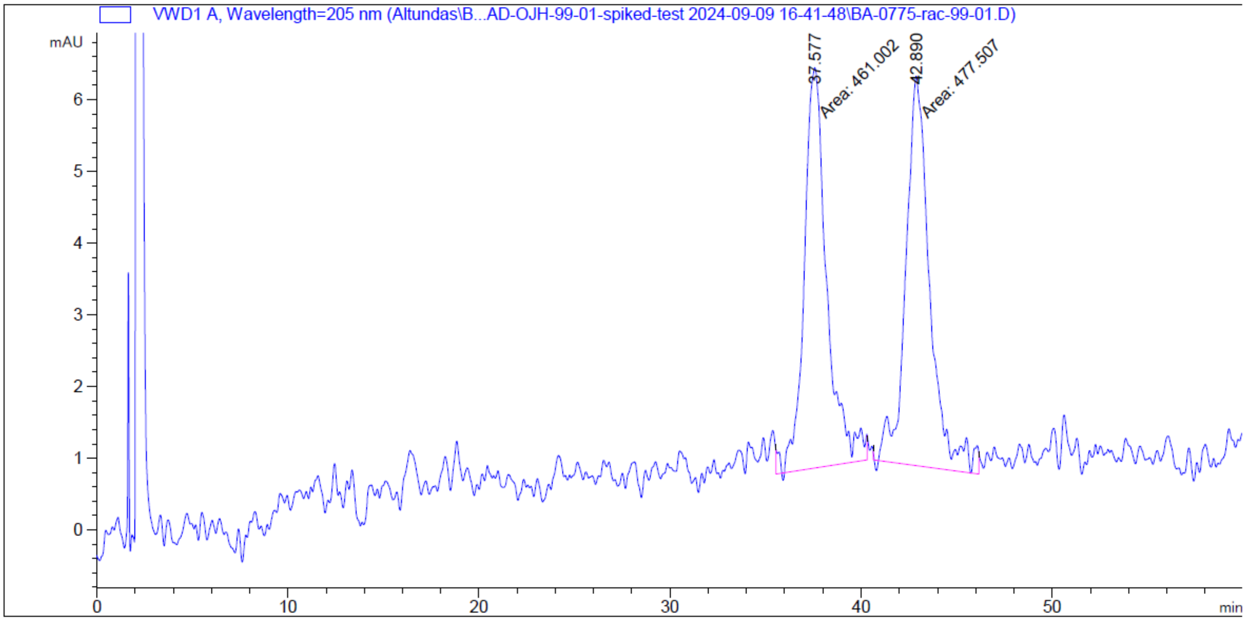

Peak #	RetTime [min]	Type	Width [min]	Area [mAU*s]	Height [mAU]	Area %
1	37.577	MM	1.3750	461.00189	5.58789	49.1207
2	42.890	MM	1.4647	477.50696	5.43353	50.8793

28' (*R*-isomer):

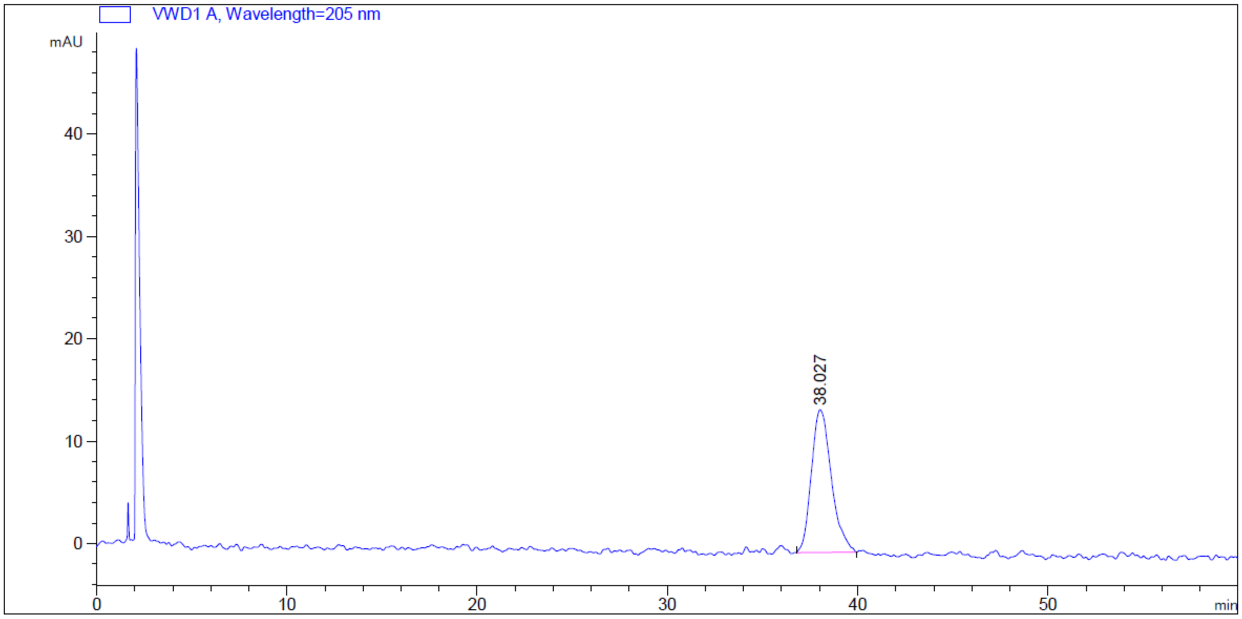

Peak #	RetTime [min]	Type	Width [min]	Area [mAU*s]	Height [mAU]	Area %
1	38.027	BB	1.0419	1018.66907	13.93926	100.0000

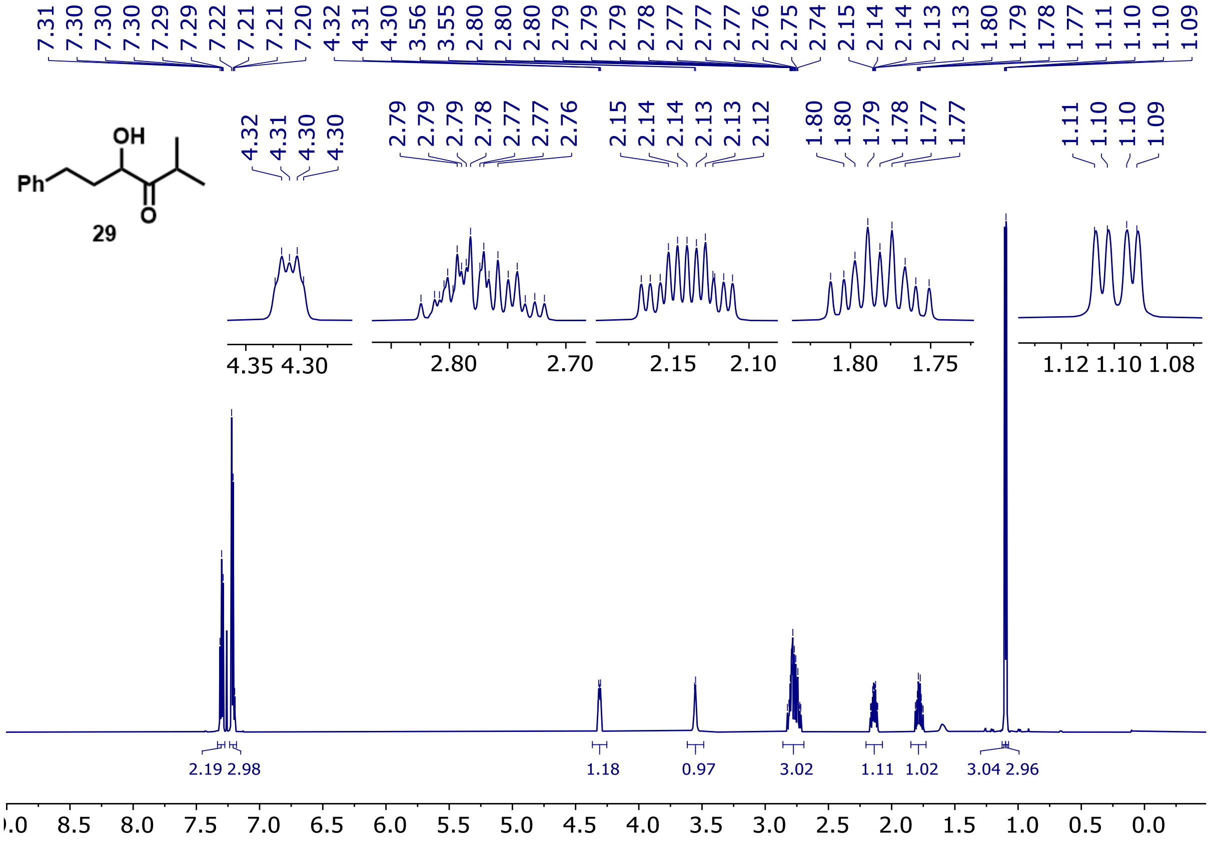
7.31 7.30 7.30 7.30 7.29 7.29 7.22 7.21 7.21 7.20 4.32 4.31 4.30 3.56 3.55 2.80 2.80 2.80 2.79 2.79 2.79 2.78 2.77 2.77 2.77 2.76 2.75 2.74 2.15 2.14 2.14 2.13 2.13 1.80 1.79 1.78 1.77 1.11 1.10 1.10 1.09
OH
Ph
O
29
2.19 2.98 1.18 0.97 3.02 1.11 1.02 3.04 2.96

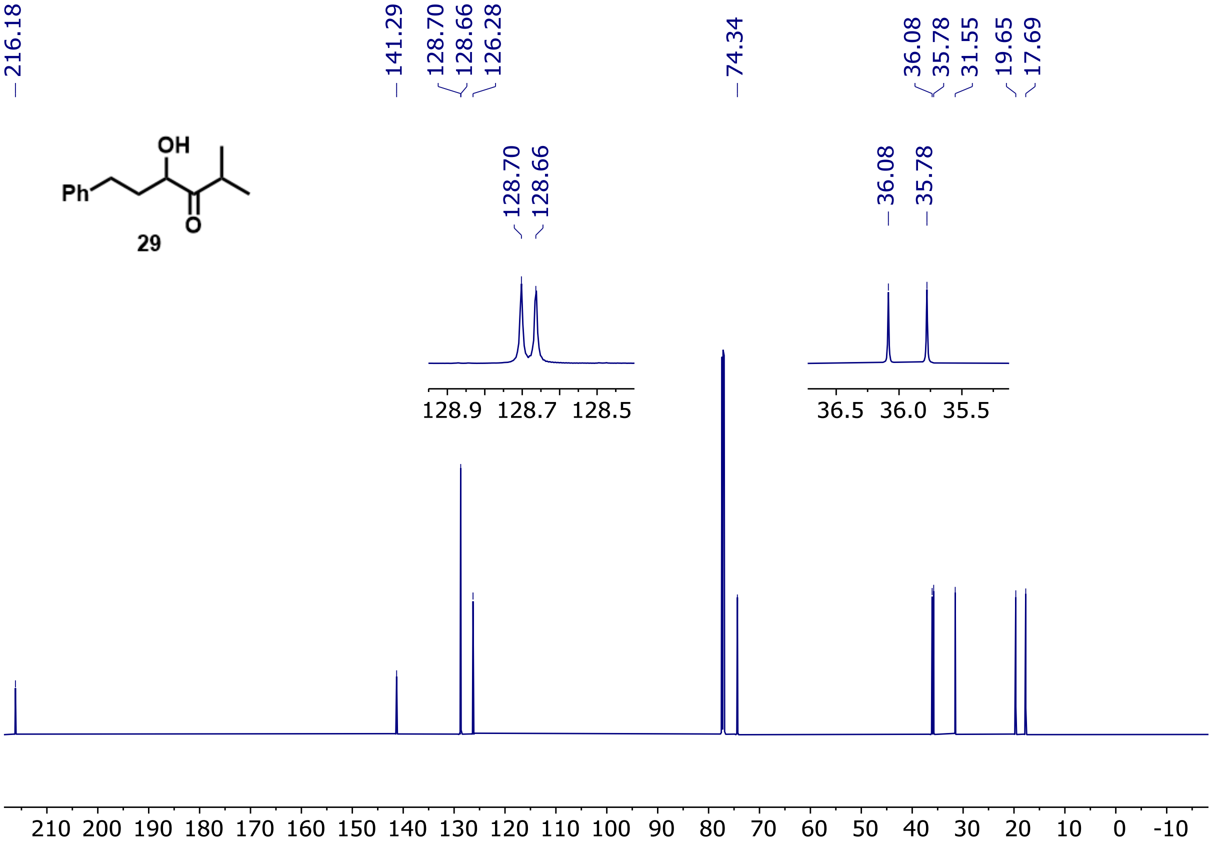
216.18 141.29 128.70 128.66 126.28 74.34 36.08 35.78 31.55 19.65 17.69
OH
Ph
O
29

29 (racemic):

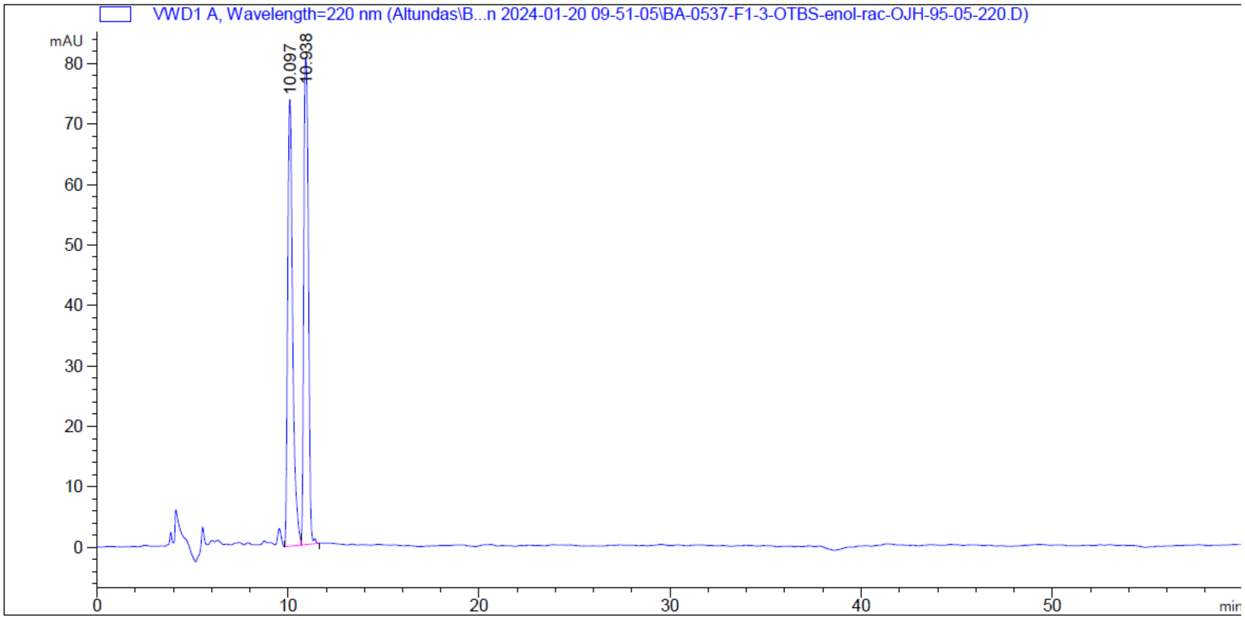

Peak #	RetTime [min]	Type	Width [min]	Area [mAU*s]	Height [mAU]	Area %
1	10.097	BV	0.2882	1354.55188	73.87106	50.0047
2	10.938	VB R	0.2762	1354.29797	80.55033	49.9953

29' (*S*-isomer):

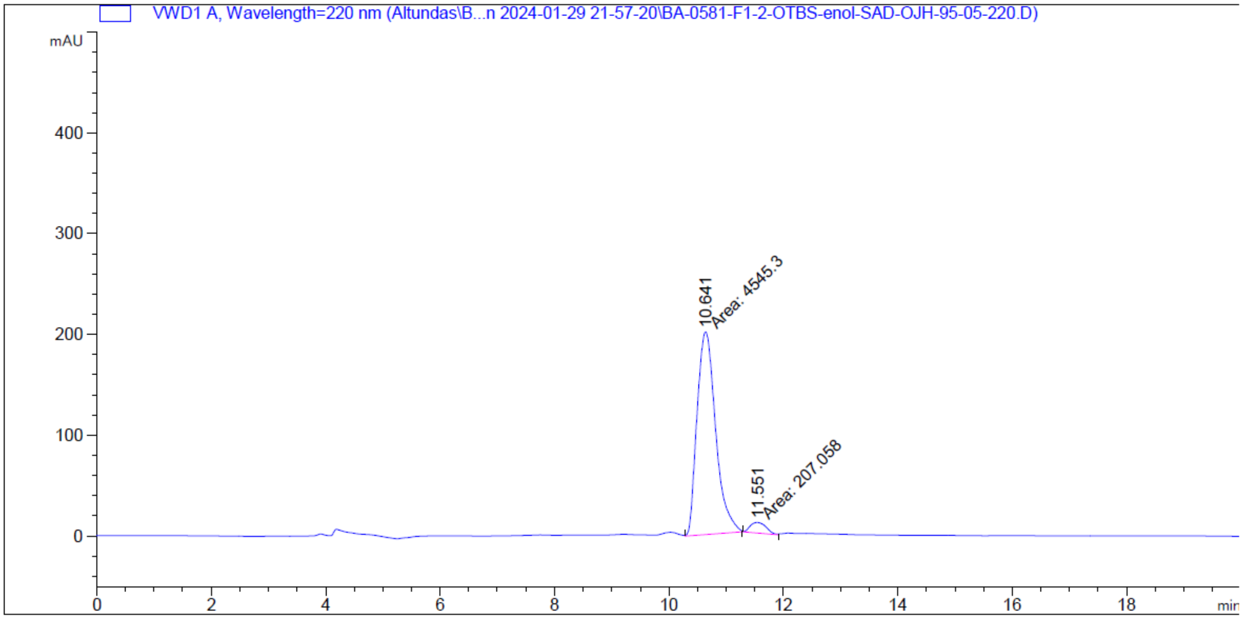

Peak #	RetTime [min]	Type	Width [min]	Area [mAU*s]	Height [mAU]	Area %
1	10.641	MM	0.3766	4545.30225	201.14586	95.6430
2	11.551	MM	0.3289	207.05818	10.49361	4.3570

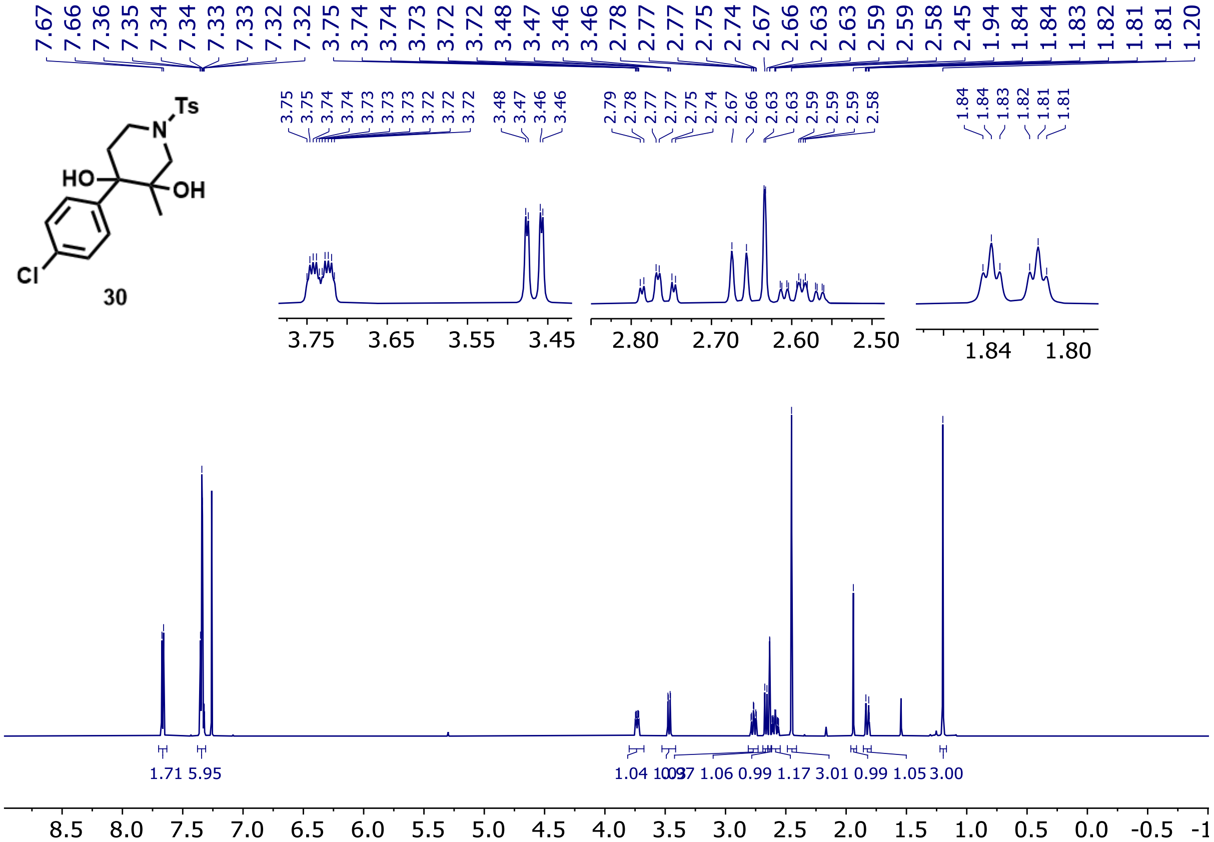
7.67
7.66
7.36
7.35
7.34
7.34
7.33
7.33
7.32
7.32
3.75
3.74
3.74
3.73
3.72
3.72
3.48
3.47
3.46
3.46
2.78
2.77
2.77
2.75
2.74
2.67
2.66
2.63
2.63
2.59
2.59
2.58
2.45
1.94
1.84
1.84
1.83
1.82
1.81
1.81
1.20
Ts
N
HO
OH
Cl
30
3.75 3.65 3.55 3.45
2.80 2.70 2.60 2.50
1.84 1.80
1.71 5.95
1.04 1.03 0.97 1.06 0.99 1.17 3.01 0.99 1.05 3.00
8.5 8.0 7.5 7.0 6.5 6.0 5.5 5.0 4.5 4.0 3.5 3.0 2.5 2.0 1.5 1.0 0.5 0.0 -0.5 -1

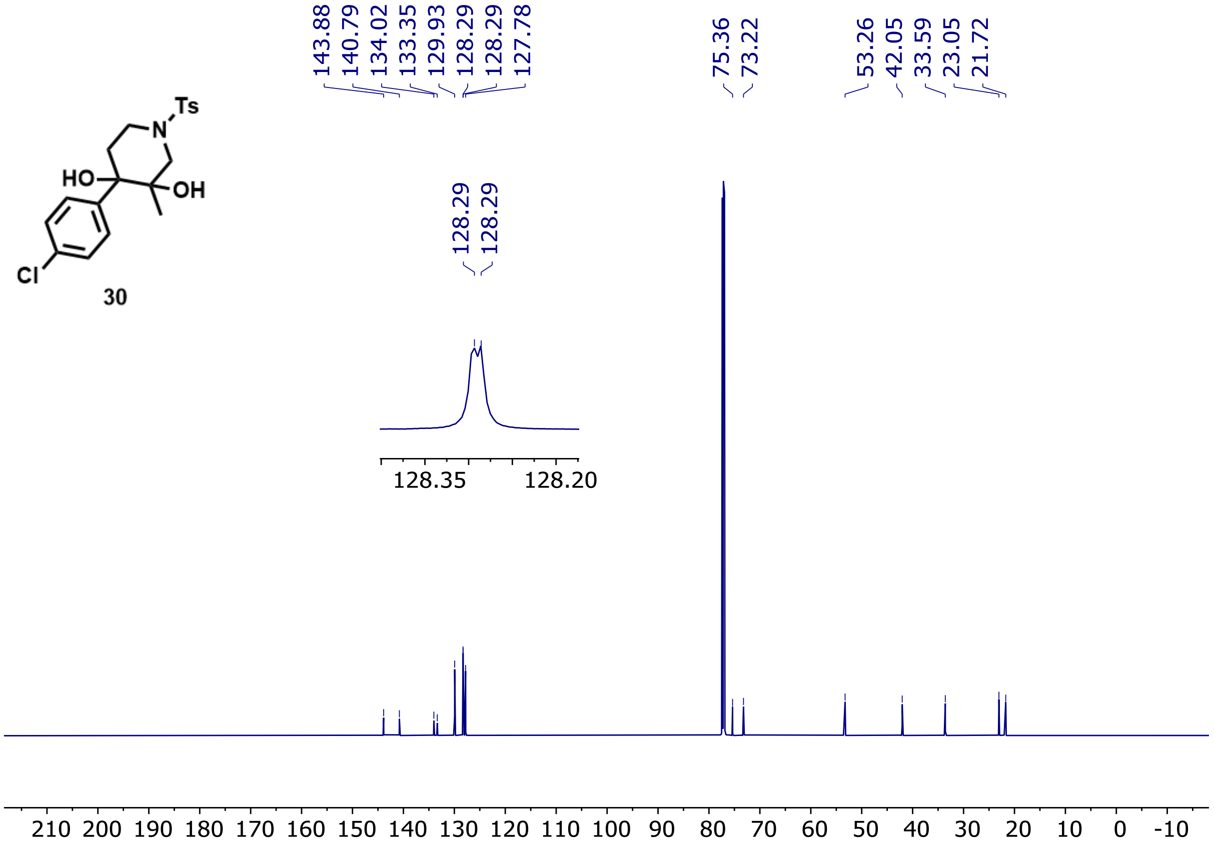
143.88
140.79
134.02
133.35
129.93
128.29
128.29
127.78
75.36
73.22
53.26
42.05
33.59
23.05
21.72
Ts
N
HO
OH
Cl
30
128.29
128.29
128.35 128.20
210 200 190 180 170 160 150 140 130 120 110 100 90 80 70 60 50 40 30 20 10 0 -10

30 (racemic):

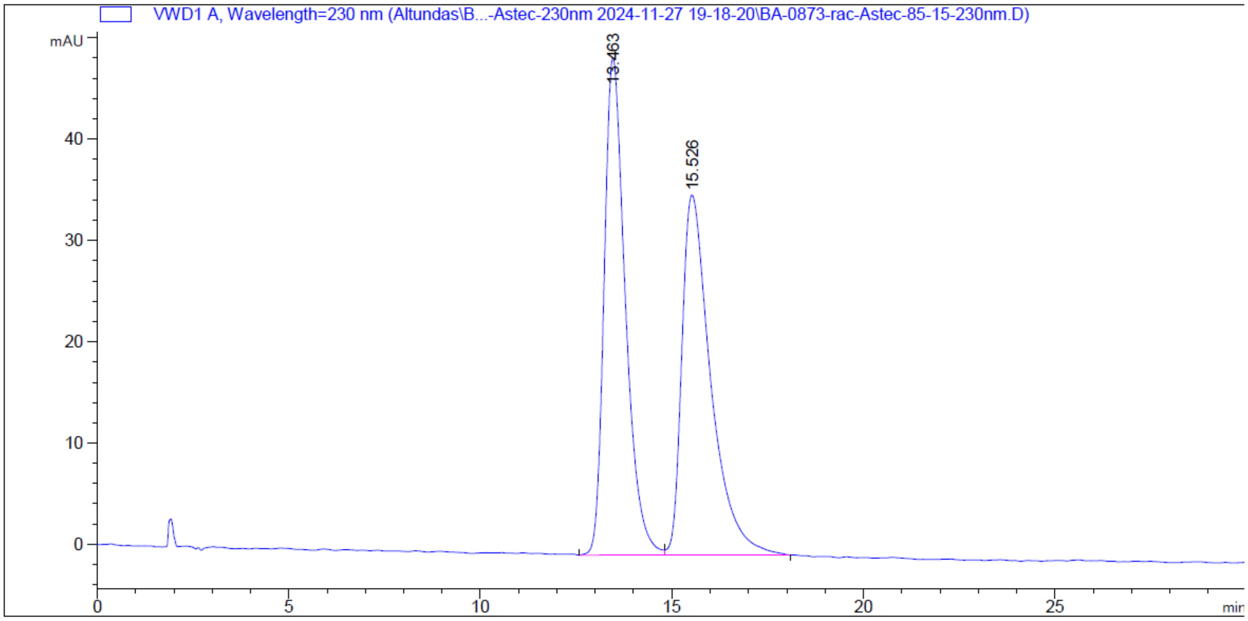

Peak #	RetTime [min]	Type	Width [min]	Area [mAU*s]	Height [mAU]	Area %
1	13.463	BV	0.5821	1891.35132	49.08054	50.3881
2	15.526	VB	0.7716	1862.21265	35.53481	49.6119

30' (*3R*, *4R*-isomer):

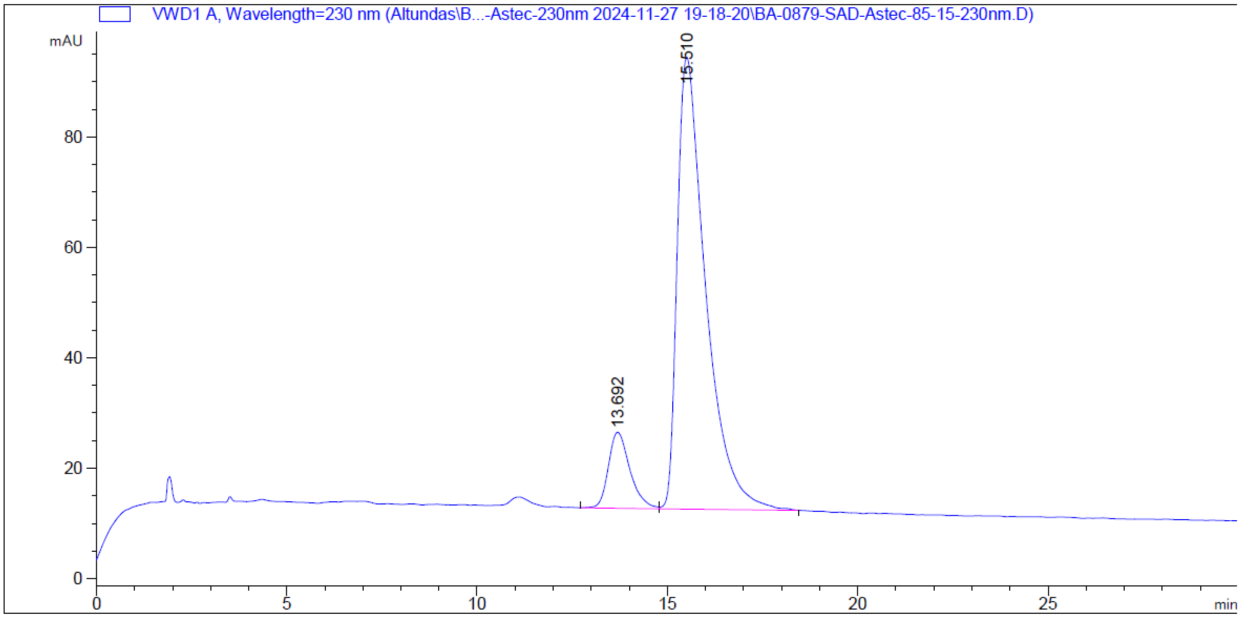

Peak #	RetTime [min]	Type	Width [min]	Area [mAU*s]	Height [mAU]	Area %
1	13.692	BV	0.5921	539.72504	13.74461	11.2689
2	15.510	VB	0.7505	4249.76367	81.76799	88.7311
